# Human Spermatogenic Failure Purges Deleterious Mutation Load from the Autosomes and Both Sex Chromosomes, including the Gene *DMRT1*

**DOI:** 10.1371/journal.pgen.1003349

**Published:** 2013-03-21

**Authors:** Alexandra M. Lopes, Kenneth I. Aston, Emma Thompson, Filipa Carvalho, João Gonçalves, Ni Huang, Rune Matthiesen, Michiel J. Noordam, Inés Quintela, Avinash Ramu, Catarina Seabra, Amy B. Wilfert, Juncheng Dai, Jonathan M. Downie, Susana Fernandes, Xuejiang Guo, Jiahao Sha, António Amorim, Alberto Barros, Angel Carracedo, Zhibin Hu, Matthew E. Hurles, Sergey Moskovtsev, Carole Ober, Darius A. Paduch, Joshua D. Schiffman, Peter N. Schlegel, Mário Sousa, Douglas T. Carrell, Donald F. Conrad

**Affiliations:** 1 Institute of Molecular Pathology and Immunology of the University of Porto (IPATIMUP), Porto, Portugal; 2 Andrology and IVF Laboratories, Department of Surgery, University of Utah School of Medicine, Salt Lake City, Utah, United States of America; 3 Department of Human Genetics, University of Chicago, Chicago, Illinois, United States of America; 4 Department of Genetics, Faculty of Medicine, University of Porto, Porto, Portugal; 5 Department of Human Genetics, National Institute of Health Dr. Ricardo Jorge, Lisbon, Portugal; 6 Department of Genetics, Washington University School of Medicine, St. Louis, Missouri, United States of America; 7 Genomics Medicine Group, National Genotyping Center, University of Santiago de Compostela, Santiago de Compostela, Spain; 8 Department of Epidemiology and Biostatistics and Key Laboratory of Modern Toxicology of Ministry of Education, School of Public Health, Nanjing Medical University, Nanjing, China; 9 Department of Oncological Sciences, Huntsman Cancer Institute, University of Utah School of Medicine, Salt Lake City, Utah, United States of America; 10 State Key Laboratory of Reproductive Medicine, Nanjing Medical University, Nanjing, China; 11 Department of Histology and Embryology, Nanjing Medical University, Nanjing, China; 12 Faculty of Sciences, University of Porto, Porto, Portugal; 13 Centre for Reproductive Genetics Alberto Barros, Porto, Portugal; 14 Galician Foundation of Genomic Medicine and University of Santiago de Compostela, CIBERER, Santiago de Compostela, Spain; 15 Genome Mutation and Genetic Disease Group, Wellcome Trust Sanger Institute, Cambridge, United Kingdom; 16 CReATe Fertility Center, University of Toronto, Toronto, Canada; 17 Department of Obstetrics and Gynaecology, University of Toronto, Toronto, Canada; 18 Department of Obstetrics and Gynecology, University of Chicago, Chicago, Illinois, United States of America; 19 Department of Urology, Weill Cornell Medical College, New York-Presbyterian Hospital, New York, New York, United States of America; 20 Center for Children's Cancer Research (C3R), Huntsman Cancer Institute, University of Utah School of Medicine, Salt Lake City, Utah, United States of America; 21 Division of Pediatric Hematology/Oncology, Huntsman Cancer Institute, University of Utah School of Medicine, Salt Lake City, Utah, United States of America; 22 Laboratory of Cell Biology, UMIB, ICBAS, University of Porto, Porto, Portugal; 23 Department of Physiology, University of Utah School of Medicine, Salt Lake City, Utah, United States of America; 24 Department of Obstetrics and Gynecology, University of Utah School of Medicine, Salt Lake City, Utah, United States of America; 25 Department of Pathology and Immunology, Washington University School of Medicine, St. Louis, Missouri, United States of America; University of Leicester, United Kingdom

## Abstract

Gonadal failure, along with early pregnancy loss and perinatal death, may be an important filter that limits the propagation of harmful mutations in the human population. We hypothesized that men with spermatogenic impairment, a disease with unknown genetic architecture and a common cause of male infertility, are enriched for rare deleterious mutations compared to men with normal spermatogenesis. After assaying genomewide SNPs and CNVs in 323 Caucasian men with idiopathic spermatogenic impairment and more than 1,100 controls, we estimate that each rare autosomal deletion detected in our study multiplicatively changes a man's risk of disease by 10% (OR 1.10 [1.04–1.16], p<2×10^−3^), rare X-linked CNVs by 29%, (OR 1.29 [1.11–1.50], p<1×10^−3^), and rare Y-linked duplications by 88% (OR 1.88 [1.13–3.13], p<0.03). By contrasting the properties of our case-specific CNVs with those of CNV callsets from cases of autism, schizophrenia, bipolar disorder, and intellectual disability, we propose that the CNV burden in spermatogenic impairment is distinct from the burden of large, dominant mutations described for neurodevelopmental disorders. We identified two patients with deletions of *DMRT1*, a gene on chromosome 9p24.3 orthologous to the putative sex determination locus of the avian ZW chromosome system. In an independent sample of Han Chinese men, we identified 3 more *DMRT1* deletions in 979 cases of idiopathic azoospermia and none in 1,734 controls, and found none in an additional 4,519 controls from public databases. The combined results indicate that *DMRT1* loss-of-function mutations are a risk factor and potential genetic cause of human spermatogenic failure (frequency of 0.38% in 1306 cases and 0% in 7,754 controls, p = 6.2×10^−5^). Our study identifies other recurrent CNVs as potential causes of idiopathic azoospermia and generates hypotheses for directing future studies on the genetic basis of male infertility and IVF outcomes.

## Author Summary

Infertility is a disease that prevents the transmission of DNA from one generation to the next, and consequently it has been difficult to study the genetics of infertility using classical human genetics methods. Now, new technologies for screening entire genomes for rare and patient-specific mutations are revolutionizing our understanding of reproductively lethal diseases. Here, we apply techniques for variation discovery to study a condition called azoospermia, the failure to produce sperm. Large deletions of the Y chromosome are the primary known genetic risk factor for azoospermia, and genetic testing for these deletions is part of the standard treatment for this condition. We have screened over 300 men with azoospermia for rare deletions and duplications, and find an enrichment of these mutations throughout the genome compared to unaffected men. Our results indicate that sperm production is affected by mutations beyond the Y chromosome and will motivate whole-genome analyses of larger numbers of men with impaired spermatogenesis. Our finding of an enrichment of rare deleterious mutations in men with poor sperm production also raises the possibility that the slightly increased rate of birth defects reported in children conceived by *in vitro* fertilization may have a genetic basis.

## Introduction

Male infertility is a multifaceted disorder affecting nearly 5% of men of reproductive age. In spite of its prevalence and a considerable research effort over the past several decades, the underlying cause of male infertility is uncharacterized in up to half of all cases [1]. Some degree of spermatogenic impairment is present for most male infertility patients, and, in its most severe form, manifests as azoospermia, the lack of detectable spermatozoa in semen, or oligozoospermia, defined by the World Health Organization as less than 15 million sperm/mL of semen. Spermatogenesis is a complex multistep process that requires germ cells to (a) maintain a stable progenitor population through frequent mitotic divisions, (b) reduce ploidy of the spermatogonial progenitors from diploid to haploid through meiotic divisions, and (c) assume highly specialized sperm morphology and function through spermiogenesis. These steps involve the expression of thousands of genes and carefully orchestrated interactions between germ cells and somatic cells within the seminiferous tubules [2]. It is likely that a large proportion of idiopathic cases of spermatogenic failure are of uncharacterized genetic origin, but measuring the heritability of infertility phenotypes has been challenging.

Known genetic causes of non-obstructive azoospermia (NOA) include deletions in the azoospermia factor (AZF) regions of the Y chromosome [3], Klinefelter's syndrome [4], and other cytogenetically visible chromosome aneuploidies and translocations [5]. Beyond these well-established causes, which are observed in 25–30% of cases, the genetic architecture of spermatogenic impairment is currently unknown. One might expect *a priori* that rare or *de novo*, large effect mutations will be the central players in genetic infertility, and indeed other primary infertility phenotypes like disorders of gonadal development, isolated gonadotropin-releasing hormone deficiency, and globozoospermia, a disorder of sperm morphology and function, appear to be caused by essentially Mendelian mutations operating in a monogenic or oligogenic fashion [6], [7], [8]. Similarly, recurrent mutations of the AZF region on the Y chromosome are either completely penetrant (AZFa, AZFb/c) or highly penetrant (AZFc) risk factors for azoospermia. Our working model at the start of this study was that additional “AZF-like” loci existed in the genome, either on the Y chromosome or elsewhere, and that, much like recent progress in the analysis of developmental disorders of childhood, a large number of causal point mutations and submicroscopic deletions could be revealed in idiopathic cases by the appropriate use of genomic technology.

In this paper, we employ oligonucleotide SNP arrays as discovery technology to conduct a whole-genome screen for two rare genetic features in men with spermatogenic failure. First, we extract and analyze the probe intensity data to find rare copy number variants (CNVs). A growing number of CNVs have been associated with a host of complex disease states [9] including neurological disorders [10], [11], [12], [13], several autoimmune diseases [14], [15], type 2 diabetes [16], cardiovascular disease [17], and cancer [18], [19], [20], [21]. Now, a role for CNVs in male infertility is beginning to emerge [22], [23], [24], [25].

As a second approach to identify rare genetic variants, we use a population genetics modeling framework to identify large homozygous-by-descent (HBD) chromosome segments that may harbor recessive disease alleles. When applied to consanguineous families, so-called “HBD-mapping” has been an unequivocal success in identifying the location of causal variants for simple recessive monogenic diseases [26]. HBD analysis can also be used to screen for the location of rare variants in common disease case-control studies of unrelated individuals, using either a single-locus association testing framework or by testing for an autozygosity burden, frequently referred to as “inbreeding depression”: an enrichment of size or predicted functional impact of HBD regions aggregated across the genome. This approach has produced results for a growing list of common diseases, including schizophrenia [27], Alzheimer's disease [28], breast and prostate cancer [29].

In this study, we screened three cohorts of men with idiopathic spermatogenic failure in an attempt to identify rare, potentially causal mutations, and to better understand the genetic architecture of the disease (Table 1). We found a genomewide enrichment of large, rare CNVs in men with spermatogenic failure compared to normozoospermic or unphenotyped men (controls). We also identify a number of cases with unusual patterns of homozygosity, possibly the result of recent consanguineous matings. Our results show that spermatogenic output is a phenotype of the entire genome, not just the Y chromosome, place spermatogenic failure firmly among the list of diseases that feature a genomewide burden of rare deleterious mutations and provide a powerful organizing principle for understanding male infertility.

**Table 1:** Case and control cohorts used in the study.

Cases					Controls			
Center	Phenotype	Ethnicity	N	Analyses	Center	Ethnicity	N	Analyses
Utah	Azoo/Oligo	Caucasian	83	C,A	Utah	Caucasian	62	C,A
Weill Cornell	Azoo	Caucasian	420	C,R,A	UKNBS	Caucasian	974	C
Porto	Azoo/Oligo	Caucasian	162	C,A	Spain	Caucasian	622	A
WUSTL	Azoo/Oligo	Caucasian	61	C,A	WUSTL	Caucasian	100	C,A
Nanjing	Azoo	Han Chinese	979	R	Nanjing	Han Chinese	1734	R

‘N’, number of individuals in the cohort after excluding ethnic outliers and samples with poor data quality. ‘Analyses’, describes whether the cohort was included in primary CNV analyses (‘C’), replication CNV analyses (‘R’), and autozygosity analyses (‘A’). Note that due to small sample sizes, the 17 Weill Cornell samples with SNP array data were merged with Porto samples and the combined set treated as a single cohort for the primary CNV analyses. Thus the total number of cases with whole-genome array data are 83+17+162+61 = 323. Many more samples were sourced from Cornell for replication analysis. Full details of each cohort are available in Text S1.

## Results

First, we attempted to find evidence for undiscovered dominant causes of spermatogenic failure by studying the genomewide distribution of CNVs in our primary cohort from Utah: 35 men with idiopathic non-obstructive azoospermia, 48 men with severe oligozoospermia, and 62 controls with normal semen analysis. All cases had previously tested negative when screened for canonical Y chromosome deletions. Samples were assayed with an Illumina 370K oligonucleotide array that provides both SNP and CNV content. There was no detectable difference in the average number of CNVs called per sample among the three groups (mean = 20, azoospermic; 19.5, oligozospermic; 20, normozoospermic), however, the majority of variants (61% on average) in any one sample were common polymorphisms.

### Rare CNV burden is a feature of spermatogenic failure

When restricting our analysis to CNVs with a call frequency of less than 5%, a subset likely to be enriched for pathogenic events, we observed pronounced differences among groups (Table S1). Azoospermic and oligozoospermic men have nearly twice the amount of deleted sequence genomewide when compared to controls (p = 1.7×10^−4^, Wilcoxon rank sum test), and a nonsignificant 12% increase in the number of deletions per genome. When examining the even more restricted set of rare CNVs larger than 100 kb (Dataset S1), these associations are more pronounced: the rate of deletions in cases was twice that of controls (1.12 vs. 0.55, p = 9.7×10^−4^) and the amount of deleted sequence 2.6 times greater in cases (p = 8.8×10^−4^).

In order to replicate these initial findings, we assayed two additional cohorts – one group of 61 Caucasian men with severe spermatogenic impairment and 100 ethnicity-matched, unphenotyped controls, both collected at Washington University in St. Louis (WUSTL), and a larger case cohort of 179 Caucasian men with idiopathic azoospermia, primarily from medical practices in Porto, Portugal, matched to an unphenotyped control set of 974 Caucasian men collected by the UK National Blood Service (NBS, [30]). Although using different array platforms (Text S1), we observed replication of our initial association (Table S2 and Table S3); in the WUSTL cohort a 20% increase in the rate (p<0.05) and in the Porto cohort a 31% increase in rate (p<5×10^−3^). We excluded several artifactual explanations for this burden effect, including specific batch phenomena or population structure (Text S1, Figures S1, S2, S3, S4, S5). To better characterize these genomewide signals, we set out to search for clustering of pathogenic mutations on specific chromosomes.

**Figure 1:**
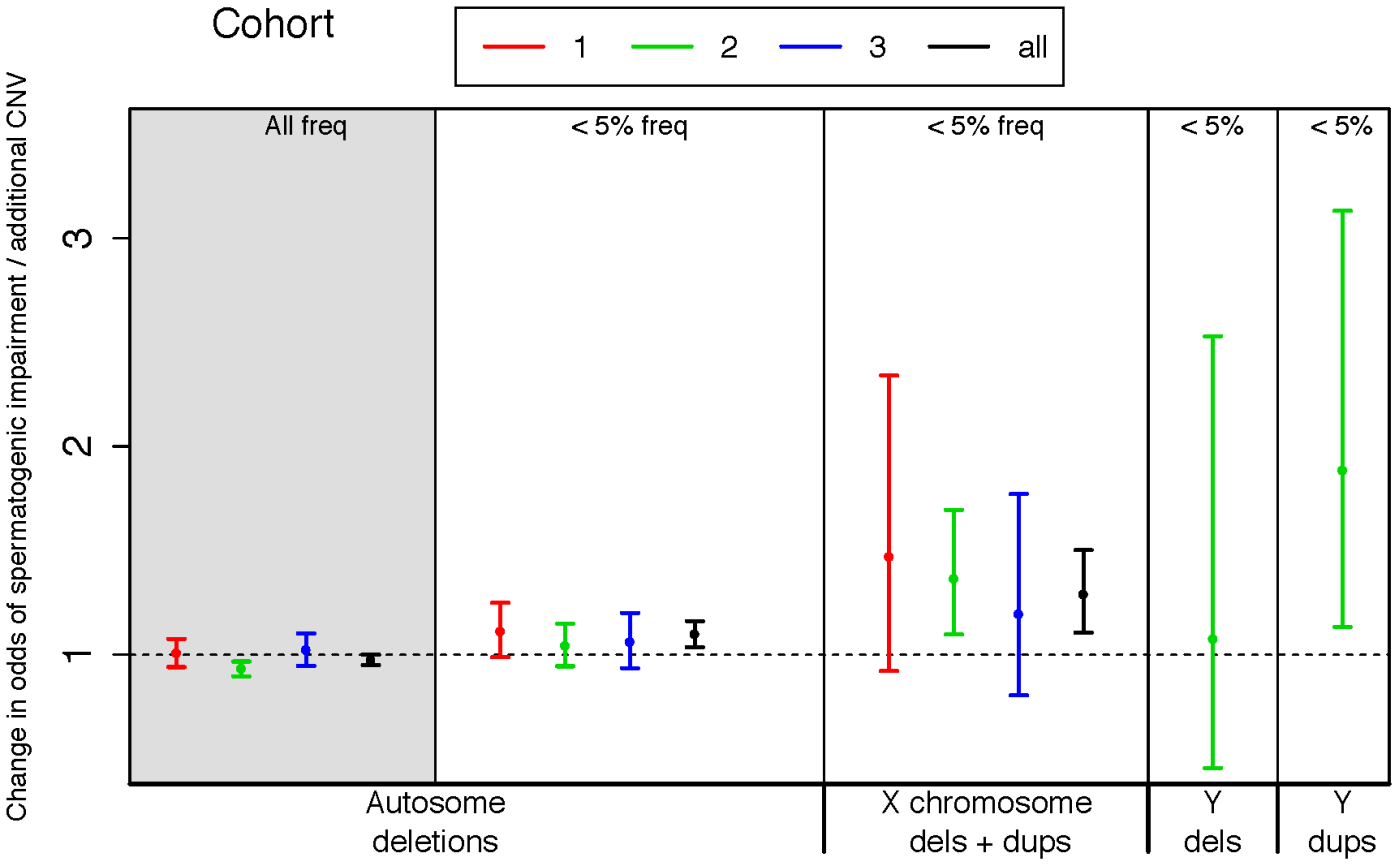
Rare variant burden in cases of spermatogenic impairment. We used logistic regression to estimate the influence of copy number variants (CNVs) on the odds of being diagnosed with impaired spermatogenesis in three case-control cohorts. The estimated odds of spermatogenic impairment is equal to, or slightly lower than, one when considering autosomal deletions of all frequencies (leftmost panel, shaded grey). However, when considering only autosomal deletions with call frequencies less than 5%, we observed a progressively increasing risk conferred by events on the autosomes, the X and the Y chromosomes. A very small number of Y-linked calls were made in cohorts 1 and 3 due to array design, thus we have only plotted Y-linked rates for cohort 2. Samples with Y-linked AZF deletions were excluded from the study. The odds ratio estimated from fitting a logistic regression model of total CNV count to disease status is plotted separately for each cohort, as well as the combined set of all cohorts (black points). Cohort 1 = Utah (Illumina 370K), 2 = Porto and Weill Cornell (Affymetrix 6.0), 3 = WUSTL (Illumina OmniExpress), All = meta-analysis of all three cohorts. Sample sizes used in CNV analysis are n = 83 cases and 62 controls for cohort 1, n = 183 cases and 974 controls for cohort 2, and n = 61 cases and 100 controls for cohort 3.

We focused first on the Y chromosome as it is the location of most known mutations modulating human spermatogenesis (Figure 1, Figure S6). Y-linked microdeletions of the AZFa, AZFb, and AZFc regions are well-established causes of spermatogenic impairment, and thus we excluded from this study cases with AZF microdeletions visible by STS PCR. In the array data, we found no significant difference in the frequency of rare Y deletions between case and controls groups; however rare duplications were more abundant in Porto cases compared to the NBS controls (a 3-fold enrichment in Porto cohort, p = 1.9×10^−3^). We could classify the majority (>90%) of our samples to major Y haplogroups using SNP genotypes (Text S1), and, as expected, most of these samples fall into the two most common European haplogroups: I (22%) and R (70%). The observed duplication burden was not an artifact of differences in major Y haplogroup frequency between cases and controls, as association was essentially unchanged when only considering samples with haplogroup R1 (p = 3.3×10^−3^). Due to low probe coverage, only one Y-linked duplication was called in the Utah cohorts (in a control individual) and two in the WUSTL cohort (both in cases), so this burden of Y duplications was not replicated.

Next we turned to the X chromosome, which is highly enriched for genes transcribed in spermatogonia [31]. In the Utah cohorts there were 71 gains and losses with a frequency of less than 5% on the X chromosome, cumulatively producing three times as much aneuploid sequence in azoospermic and oligozoospermic men compared with normozoospermic men (89 kb/person azoo, 45 kb/person oligo, 27 kb/person normozoospermic men, all cases versus controls p<0.03). This burden was strongly replicated in the Porto samples, which displayed a 1.6 fold enrichment of rare CNV on the X (p = 5×10^−4^) and the WUSTL samples (31% of cases with a rare X-linked CNV versus 16% of controls, p = 0.02 by permutation).

The genome-wide signal of CNV burden was not driven solely by sex chromosome events: considering only autosomal mutations in Utah samples there was an enrichment of aneuploid sequence in large deletions in azoospermic men (268 kb/person) and oligozoospermic men (308 kb/person) compared to control men (189 kb/person, p = 9.8×10^−3^), and an enrichment in the rate of deletions in all cases when considering just events >100 kb (1.9 fold enrichment, p = 6×10^−3^). In the Porto cohort, there was modest evidence for a higher rate of rare deletions of all sizes in azoospermic men (1.27 fold enrichment, not significant) as well as an increase in total amount of deleted sequence (345 kb/case vs. 258 kb/control, p<0.003).

In order to cleanly summarize our findings across all cohorts, we fit logistic regression models for each cohort, regressing case status onto CNV count for different classes of CNV. We also fit a linear mixed-effects logistic regression model to the total dataset for each CNV class, treating cohort as a random factor (Figure 1). In each regression model we controlled for population structure by including eigenvectors from a genomewide principal components analysis (Methods). On the basis of the combined analysis, we estimate that each rare autosomal deletion multiplicatively changes the odds of spermatogenic impairment by 10% (OR 1.10 [1.04–1.16], p<2×10^−3^), each rare X-linked CNV (gain or loss) by 29%, (OR 1.29 [1.11–1.50], p<1×10^−3^) and each rare Y-linked duplication by 88% (OR 1.88 [1.13–3.13], p<0.03).

### Locus-specific analyses

Deletions of the AZF regions of the Y chromosome are often mediated by non-allelic homologous recombination (NAHR) between segmental duplications and are the most common known cause of spermatogenic failure. Because of their prognostic power and high rate of recurrence in the population, screening for AZF deletions is a standard part of the clinical workup for azoospermia. It would be of high clinical value if additional azoospermia susceptibility loci with significant recurrence rates could be identified.

We screened all cohorts for large (>100 kb) rearrangements flanked by homologous segmental duplications capable of generating recurrent events by NAHR [32]. There was no significant enrichment of gains or losses in cases across these hotspot regions when considered as an aggregate. Due to small sample sizes we found no single-locus associations, at these hotspot loci, or elsewhere, that met the strict criteria of genomewide significance in both the discovery and replication cohorts. Many of our single-cohort associations from one platform lack adequate probe coverage on other platforms for robust replication (Text S1). However, several loci were significant on joint analysis of all cohorts.

**Figure 2:**
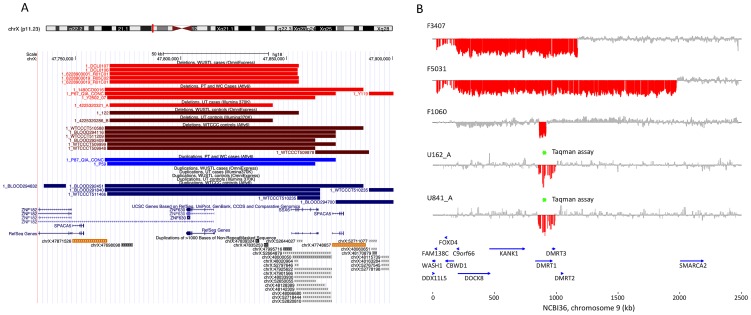
Discovery of recurrent deletions in azoospermia. (A) A recurrent microdeletion on Xp11.23 (47765109–47871527 bp, hg18) is a strong candidate risk factor for spermatogenic failure. The location of deletions (red shades) and duplications (blue shades) in cases and controls are plotted separately for each cohort. CNVs at this locus appear to arise due to non-allelic homologous recombination between two nearly identical (>99.5% homology) 16 kb segmental duplications that contain the sperm acrosome gene *SPACA5*. Also within the CNV region are the genes *ZNF630* and the cancer-testis antigen *SSX6*. We identified 9 deletions of this locus spread across all patient cohorts (3 in PT, 1 in UT, 5 in WUSTL) compared to 8 in the pooled 1124 controls (2.8% frequency versus 0.7%, odds ratio = 3.96, p = 0.005, Fisher exact test). After analysis of an additional 403 cases and 2121 controls, the association is still significant (combined data: 1.6% frequency in cases, 0.55% in controls, OR 3.0, 95% CI = [1.31–6.62], p = 0.007). (B) We identified two patients with deletion of *DMRT1*, a gene on 9p24.3 that is orthologous to the putative sex determination locus of the avian ZW chromosome system [36]. Both men were diagnosed as azoospermic. We validated these deletion calls with a qPCR assay (green star, Figure S9). We screened Affymetrix 6.0 data from an independent Han Chinese case-control study of NOA and identified an additional 3 deletions of *DMRT1* coding sequence in 979 cases and none in 1734 controls. Finally, we observed no coding deletions of *DMRT1* in the two largest control SNP array datasets in the Database of Genomic Variants, consisting of 4519 samples [42], [43]. The combined results indicate that deletion of *DMRT1* is a highly penetrant genetic cause of human spermatogenic failure (frequency of 0.38% in 1306 cases and 0% in 7754 controls, combined p = 6.2×10^−5^). Patient IDs are indicated next to each plot (U162_A, U841_A = Utah cohort patients; F3407, F5031, F1060 = Nanjing cohort patients).

The best candidate for a novel locus generating NAHR-mediated infertility risk mutations is a 100 kb segment on chromosome Xp11.23 flanked by two nearly identical (>99.5% homology) 16 kb segmental duplications containing the sperm acrosome gene *SPACA5* (Figure 2a, Figure S7). We identified 9 deletions of this locus spread across all patient cohorts (3 in PT, 1 in UT, 5 in WUSTL) compared to 8 in the pooled 1124 controls (2.8% frequency versus 0.7%, odds ratio = 3.96, p = 0.005, Fisher exact test). We genotyped the deletion by +/− PCR in an additional cohort of 403 men with idiopathic NOA from Weill Cornell, and observed an additional 3 deletions (Figure S8, Text S1). In a prior case-control study of intellectual disability, investigators using qPCR estimated the allele frequency of this deletion to be 0.47% (10/2121) in a large Caucasian male control cohort [33]. Combining these data, we estimate the allele frequency of the deletion to be 1.6% in Caucasian cases, compared to 0.55% in Caucasian controls (OR 3.0, 95% CI 1.31–6.62, p = 0.007). The deleted region contains the X-linked cancer-testis (CT-X) antigen gene *SSX6*; the CT-X antigen family is a highly duplicated gene family on the X chromosome comprising 10% of all X-linked genes and is expressed specifically in testis. After controlling for differences in coverage across the array platforms used in this study, we find a significant enrichment of rare deletions of CT-X genes in all cases (p = 0.02); this finding did not extend to duplications or CT antigen genes on the autosomes (Table 2).

**Table 2:** X-linked cancer-testis antigens deleted in case and control samples.

GENE	START**	STOP	PT/WC	UTAH	WUSTL	CASE COUNT	CONTROL COUNT
SSX6†	47852031	47865013	3	1	5	9	8
SSX1	47999740	48011823	0	1	0	1	2
SSX3	48090806	48101086	0	0	0	0	1
GAGE10	49047068	49063255	0	0	0	0	1
NXF2B	101501974	101613388	1	0	0	1	1
CT47*	119895375	119898693	1	1	0	2	1
CT45*	134674850	134684654	9	0	0	9	21
SPANXA1/A2* †	140499461	140500526	0	0	0	0	6
MAGEA11†	148575476	148604507	0	1	0	1	0
MAGEA9†	148671401	148677206	0	0	0	0	1
MAGEA8†	148770653	148775266	1	0	0	1	0
Unique Samples						24 (7.3%)	42 (3.7%)***

* Gene or gene family is annotated multiple times on the reference genome; coordinates for the first copy are given.

** Gene coordinates are based on NCBI36.

*** Frequency difference between cases and controls, p<0.05.

† Patient-specific deletions of these genes were reported in a study of X-linked CNVs in over 250 azoospermia cases and 300 normospermic controls [58].

When analyzing all cohorts jointly, our strongest association (genomewide corrected p-value <0.002) is to both gains and losses involving a 200 kb tandem repeat on Yq11.22, *DYZ19* (Figure S6, Figure S9), a human-specific array of 125 bp repeats first discovered as a novel band of heterochromatin in the Y chromosome sequencing project [34]. Tandem repeat arrays are often highly unstable sequence elements that can mutate by both replication-based and recombination-based (e.g. NAHR) mechanisms. In our data there were 9 gains and 11 losses at *DYZ19* in 323 cases (combined frequency 6.1%), compared to 3 gains and 12 losses in 1136 controls (combined frequency 1.3%). While this finding may ultimately require painstaking technical work to conclusively validate, we have several reasons to believe the association is real. First, we have previously shown that it is possible to identify real copy number changes at VNTR loci using short oligonucleotide arrays [35]; second, copy number changes at this locus were identified by multiple platforms in the current study; third, the association is nominally significant in both the Utah and Porto cohorts; fourth the locus is within the AZFb/c region. The direction of copy number changes does appear to track with haplogroup – while 12/13 duplications occur on the R1 background, 14/15 deletions for which haplogroup could be determined occur on I or J background. Haplogroup assignments for the carriers of these CNVs were confirmed by standard short tandem repeat analysis (Text S1). The strong association between haplogroup and direction of copy number change is noteworthy; it may indicate that DYZ19 CNVs are merely correlated with other functional changes on these chromosomes, or perhaps the structure of these chromosomes predisposes them to recurrent gains (R1) or losses (I/J).

The gene *DMRT1* is widely believed to be the sex-determination factor in avians, analogous to *SRY* in therians, and may play the same or similar role in all species that are based upon the ZW sex chromosome system [36]. *DMRT1* encodes a transcription factor that can activate or repress target genes in Sertoli cells and premeiotic germ cells through sequence-specific binding [37]. In humans, *DMRT1* is located on 9p24.3 in a small cluster with the related genes *DMRT2* and *DMRT3*. Large terminal deletions of 9p are a known cause of syndromic XY sex-reversal, and although the role of the *DMRT* genes in the 9p deletion syndrome phenotype has not yet been defined, mouse experiments have shown that homozygous deletion of *DMRT1* causes severe testicular hypoplasia [38], [39], [40].

We found two, perhaps identical, 132 kb deletions spanning *DMRT1* in the Utah cohort in men with azoospermia, and a 1.8 Mb terminal duplication of 9p, spanning these genes, was seen in a single normozoospermic control from Utah (Figure 2b). All three of these rearrangements were validated by TaqMan assay (Figure S10, Text S1). Both men were recruited into the study in Salt Lake City, UT between 2002 and 2004. They self-reported their ancestry as Caucasian, and in both cases this assumption was clearly verified by principal components analysis of their genetic data (Figure S2). There was no evidence that the two deletion carriers were closely related upon comparison of their whole-genome SNP genotypes. Testis biopsies were performed on both men; these indicated apparent Sertoli cell only syndrome in the first and spermatocytic arrest in the second. Both men exhibited apparently normal male habitus and virilization with no phenotypic similarities to 9p deletion syndrome.

We obtained Affymetrix 6.0 array data from a previously published genomewide association study of idiopathic NOA in Han Chinese [41] comprised of 979 cases and 1734 controls (Text S1). After processing these samples with our CNV calling pipeline, we observed an additional 3 deletions of *DMRT1* exonic sequence in cases (0.3%) and none in controls (Figure 2B, Figure S11). From these combined array data we estimate a frequency of *DMRT1* exonic deletion of 0.38% (5/1306) in cases and 0% (0/2858) in controls (OR = Infinity, [2.0-Inf], p = 0.003). We obtained the two largest control SNP array datasets in the Database of Genomic Variants (DGV), representing CNV calls from 4519 samples typed with platforms of equal or higher probe density to the ones used here [42], [43]. None of these samples contained CNV of any sort affecting *DMRT1*. Finally, we screened an additional set of 233 idiopathic NOA cases from Weill Cornell, and 135 controls with the TaqMan validation assay and identified an additional 3 deletions (2 in cases, 1 in controls, Text S1, Figure S12). As this qPCR assay interrogates intronic sequence, the functional consequences of these 3 deletions are unclear. Our array data have revealed some of the smallest coding deletions of *DMRT1* reported to date in humans, and should help to clarify the critical regions of 9p involved in testicular development and function.

Notably, using a bespoke reanalysis of the intensity data, we did not see evidence for CNVs involving the gene *PRDM9*, a recently characterized zinc finger methyltransferase that appears to control the location of recombination hotspots in a diversity of mammalian species. Heterozygosity of *PRDM9* zinc finger copy number has been shown to cause sterility in male hybrids of *Mus m. domesticus* and *Mus m. musculus* due to meiotic arrest [44].

### Functional impact

The identification of functional or physical annotations enriched in case-associated CNVs can be a powerful step in constructing models to classify pathogenic variants. We searched for significant case-specific aggregation of CNVs in several classes of functional sequence, including 195 genes previously shown to result in spermatogenic defects when mutated in the mouse [45], all protein and non-protein coding genes, and 525 testis genes that are differentially expressed during human spermatogenesis (Text S1). Deletion of X- or Y-linked exonic sequence conferred the strongest risk (OR = 1.87 [1.30–2.68], p<1×10^−3^). Very similar risk was associated with deletion of exonic sequence from testis genes differentially expressed during spermatogenesis, despite the fact that only 15% of these genes are located on the sex chromosomes (OR = 1.85 [1.01–3.39], p<0.05). Deletion of any exonic sequence was also associated with disease (OR = 1.25 [1.07–1.46], p<5×10^−3^). Deletion of miRNAs was not associated, nor was deletion of the 195 mouse spermatogenic genes [45], which were very rarely deleted in either cases or controls.

**Figure 3:**
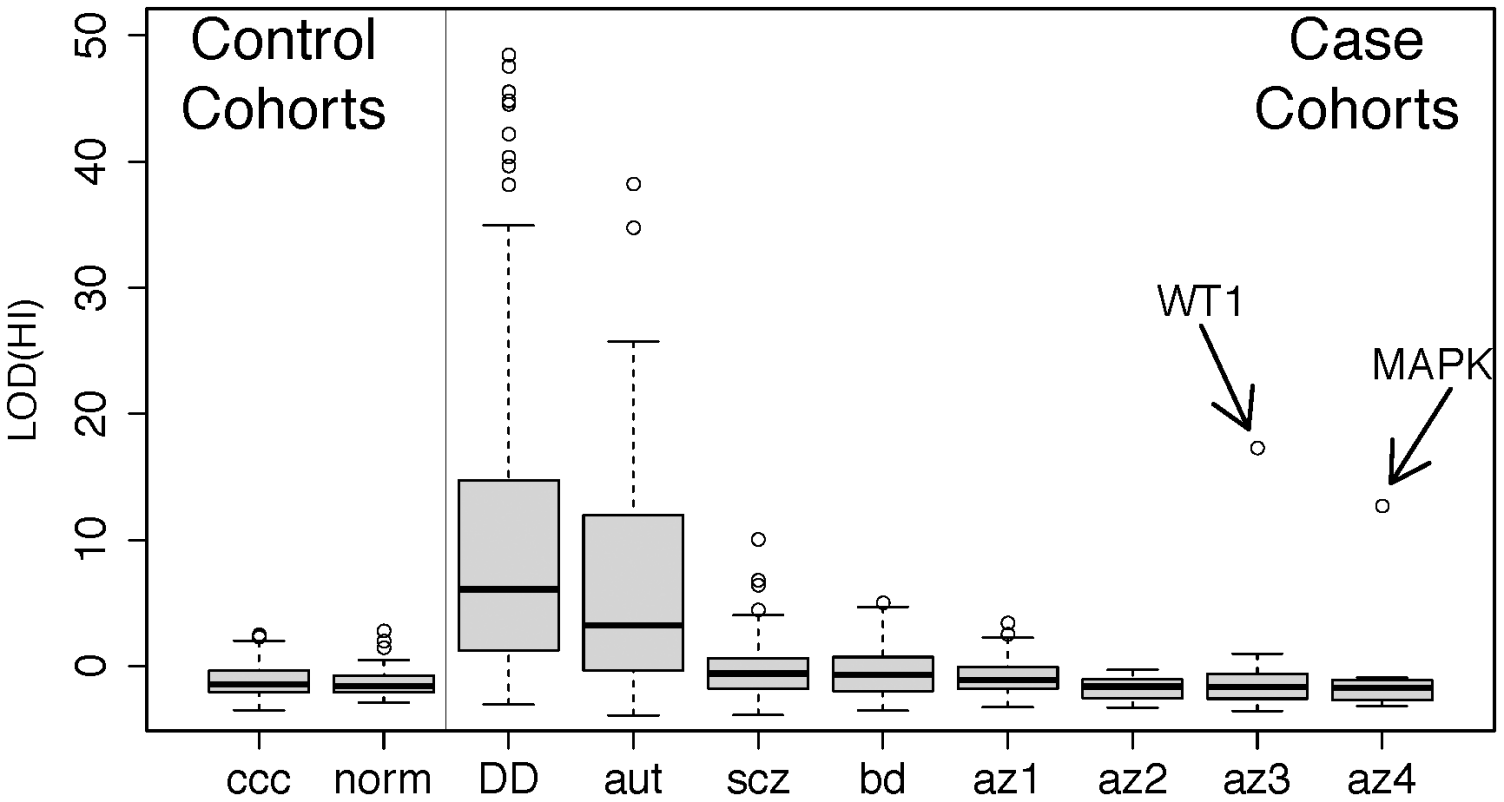
Disruption of predicted haploinsufficient genes is infrequent in spermatogenic failure. We obtained lists of rare deletions, left panel, from the Utah and WTCCC control cohorts and, right panel, from cohorts of developmental delay (DECIPHER) [66], autism [67], schizophrenia [68], bipolar disorder [66], [68], and spermatogenic impairment (this study). We used a published method for assessing the likelihood that each deletion disrupts a haploinsufficient gene [47], summarized as a LOD score, and ordered each cohort by the median LOD(HI) within cases and controls separately. While the CNVS from DECIPHER (p<1×10^−15^), autism (p<1×10^−15^), schizophrenia (p<1×10^−4^) and bipolar disorder (p<0.002) show significant enrichment of high LOD (HI) scores compared to controls, the infertility cohorts have score distributions indistinguishable from controls. Two outlier deletions from the infertility cohort are annotated; one is a deletion of *WT1*, a key gene in gonadal differentiation, and the other is a 1 Mb deletion involving several genes including *MAPK1* and the cancer-testis antigen *PRAME*. Further review of clinical data from the *WT1* carrier showed signs of cryptorchidism. Abbreviation of azoospermia cohorts: az1, Utah cohort, az2, WUSTL, az3 Porto, az4, Weill-Cornell. Note that for additional detail we have split the cohort referred to as “Porto” in the main text into two subgroups, az3 and az4, defined by the clinical group that ascertained the cases.

We hypothesized that at least some of the functional impact of CNV burden on fertility was a result of disruption of haploinsufficient (HI) genes, as has been demonstrated for neuropsychiatric and developmental disease [46]. For each singleton deletion in our collections we used a recently described modeling framework to calculate the probability that the deletion is pathogenic due to dominant disruption of a haploinsufficient gene [47]. Much to our surprise, HI scores from deletions in infertility cases were much smaller than those from cases of autism and developmental disorders and in fact indistinguishable from controls (mean HI score −1.16 in controls, −1.02 in all spermatogenic impairment cases, p = 0.49 by Wilcoxon rank sum test; Figure 3). Likewise there was no enrichment of large rearrangements within 45 known genomic disorder regions in cases [46]. In contrast to previously described diseases that feature CNV burden, spermatogenic impairment may be more likely to result from large effect recessive mutations, or perhaps the additive effect of deleterious mutations across many loci. We sought to uncover support for recessive mutation load in our cases by assessing the impact of inbreeding, or elevated rates of homozygosity, on disease risk by applying a population genetic approach to the SNP genotype data from our samples [48].

### HBD analyses

The major genetic side effect of consanguineous mating is a genome-wide increase in the probability that both paternal and maternal alleles are homozygous-by-descent. This probability is often summarized as the inbreeding coefficient, *F*, and can be estimated from analysis of pedigree structure or by direct observation of genomewide SNP genotypes.

Due to differences in demographic history and culture, the extent of background homozygosity in the genome is expected to vary when comparing diverse populations throughout the globe. The haplotype modeling algorithms implemented in the software package BEAGLE estimate the background patterns of linkage disequilibrium and homozygosity across a set of samples, allowing population-specific information to be used to assess the evidence that any given section of a genome is likely to be homozygous-by-descent (HBD). During the course of our study we concluded that standard PCA-based approaches to stratification are insufficient to correct for population structure during the analysis of inbreeding, even when using population genetic methods like BEAGLE (Text S1, Figure S13). The problem comes not from spurious identification of HBD, but from spurious association of HBD with disease status when case and controls are sampled from groups with different levels of background relatedness. For instance, in a recent survey of 17 Caucasian cohorts, estimates of the average inbreeding coefficient, *F*, varied from 0.09% to 0.61%, with UK-based cohorts showing the lowest *F* and the one Portuguese cohort showing the highest [27]. While PCA-based methods traditionally detect and correct for differences in allele frequencies among groups, we believe that they do not detect differences in inbreeding that can be readily incorporated into a case-control testing framework. In the following section, we use data from 622 healthy adults from Spain, who we believe form a more appropriate control group for the Porto case cohort (Methods, Text S1, Figure S13).

**Figure 4:**
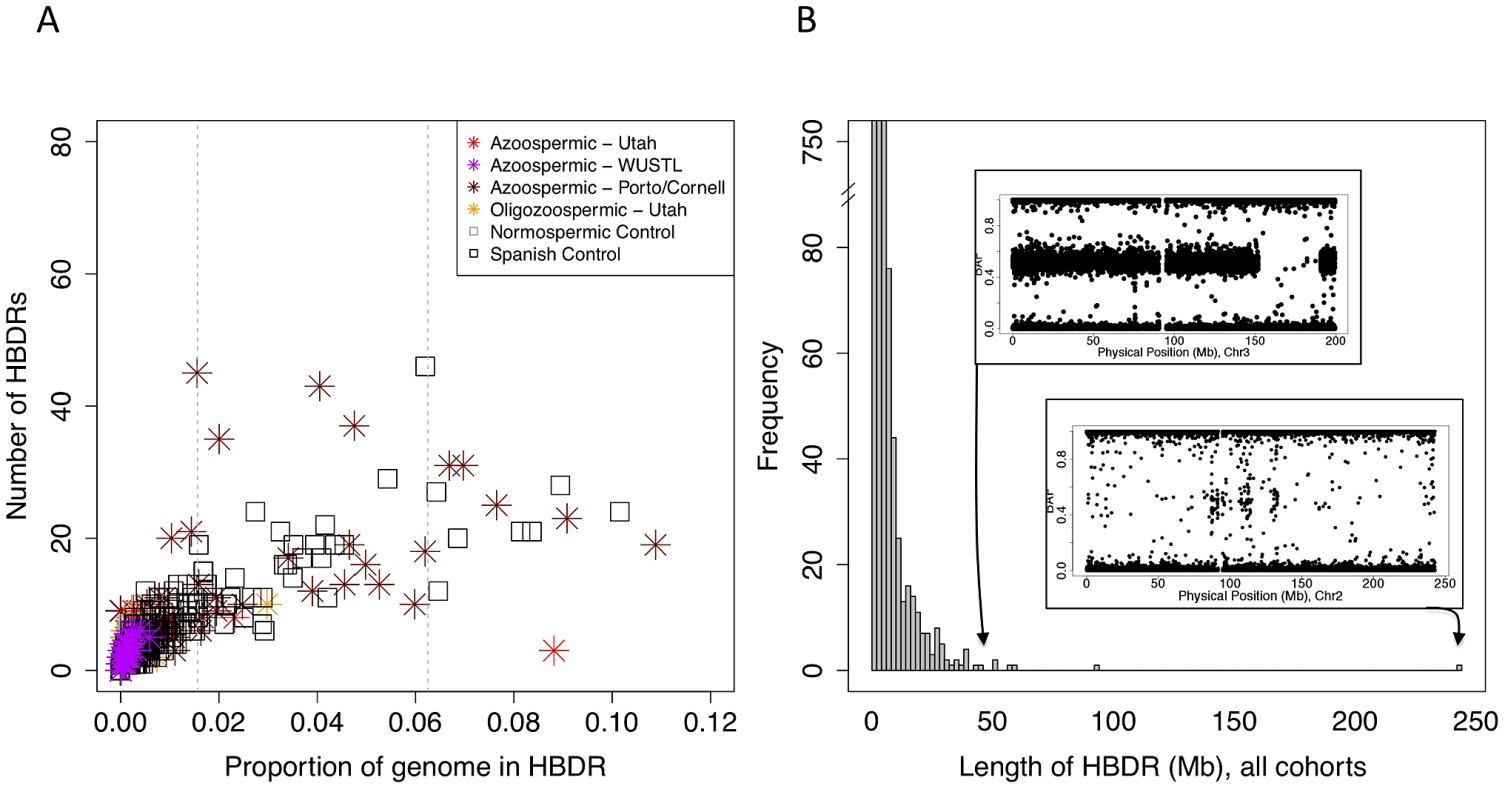
Patterns of homozygosity in men with low sperm count. (A) Distribution of the number of HBD regions (HBDRs), and the proportion of genome contained in these putative HBD regions, plotted for each sample in this study. Replication case and control cohorts are indicated in the legend. (B) Length distribution of HBDRs detected in all samples combined. Inset, two panels showing probe level intensity data corresponding to the two largest HBDRs detected. BAF: b-allele frequency, calculated as B/(A+B) where A and B are the approximate copy numbers for the A and B allele, respectively. The largest HBDR detected corresponds to a case of uniparental disomy of chromosome 2 (UPD2) detected in an azoospermic man from the Utah cohort.

**Figure 5:**
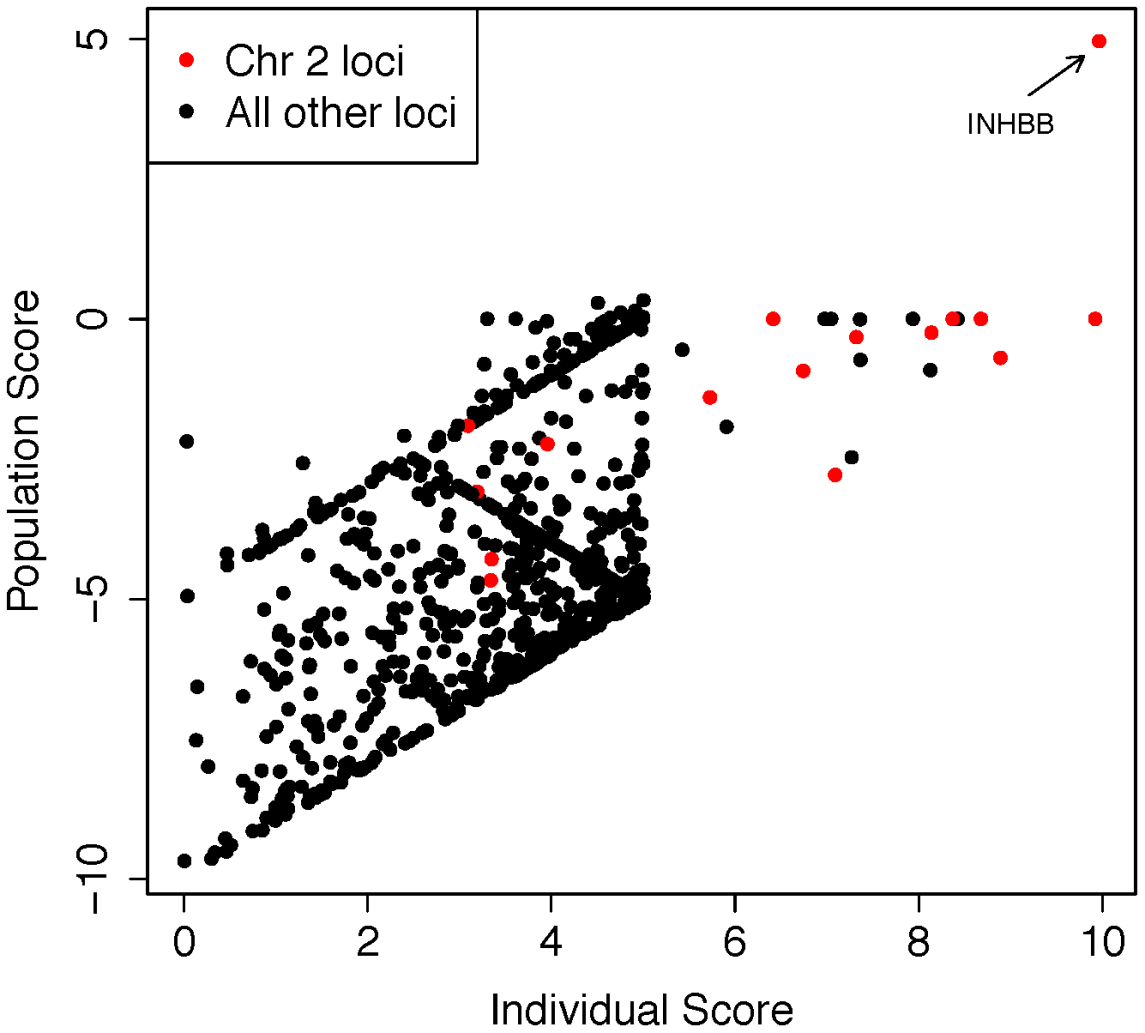
Analysis of exome sequencing data identifies a candidate azoospermia mutation in the case of UPD2. We performed whole-exome sequencing on the case of UPD2 in an attempt to identify a potential genetic cause for this man's azoospermia. We constructed a scoring method to rank order the exome variants in two dimensions: (i) within the set of variants seen in this single exome, the “Individual Score” and (ii) across a large set of exome sequences, the “Population Score”. For each exome variant, the Individual Score, P_ind,_, was constructed by summing normalized predictions of functional impact from 5 commonly used annotation algorithms: PhyloP, PolyPhen2, SIFT, GERP, and LRT. This score was then multiplied by the ploidy of the mutant allele (e.g. 1× for a heterozygous genotype and 2× for a homozygous genotype) creating a final Individual Score ranging from 0–10. We also calculated the Individual Score for all variation in the 1000 genomes Phase I sequencing data. To construct the “Population Score” for each variant in the UPD individual, P_pop_, we identified the maximum Individual Score variant in the corresponding gene, P_max_, within the 1000 genomes data, and defined P_pop_ = P_ind_−P_max_. The purpose of the Population Score is to scale the importance of each Individual Score by the extent of pathogenic variation that exists in the population at each gene. Only sites with minor allele frequencies less than 10% in both the 1000 genomes data and the Exome Variant Server (http://evs.gs.washington.edu/EVS/) were considered in the analysis. When examining the joint distribution of P_pop_ and P_ind_ for the UPD2 individual, we saw an enrichment of large scores for variants on chromosome 2, as expected. The most extreme variant on both scales was a homozygous nonsense mutation in the gene *INHBB*, the implications of which we discuss in Figure 6.

**Figure 6:**
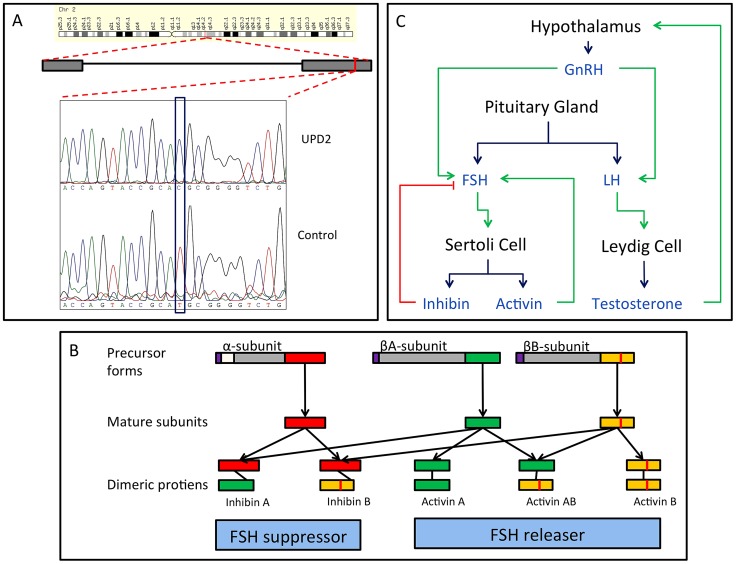
Homozygous missense mutation of *INHBB* identified in the case of UPD2. (A) We validated this candidate by Sanger sequencing in the UPD2 case and control individuals. Mutant and reference nucleotides are highlighted within the blue box, confirming the homozygous T to C nucleotide change observed at chr2:12,1107,305 bp (hg19) of the UPD2 individual. Grey boxes represent the exons of the gene and the red line indicates the location of the observed mutation within the gene. (B) *INHBB* encodes for the protein, Inhibin βB, which along with inhibin α and inhibin βA, combine combinatorially to form the inhibins and activins. Each protein expressed by *INHA*, *INHBA*, *INHBB* consists of an N-terminal signal peptide (purple), a propeptide (grey), and a subunit chain (green, red or yellow). The mutation identified here results in a M370T change of the inhibin βB subunit chain (location indicated by a vertical red line throughout the diagram). The various inhibin subunits dimerize via disulfide bonds (locations indicated by black lines between subunits). As the βB subunit participates in multiple complexes with antagonistic functions, the functional consequences of loss-of-function or gain-of-function mutations in this protein may be difficult to predict. (C) The role of inhibins and activins in the hypothalamic-pituitary testicular axis. These complexes have diverse functions in the body, but are most well known for their ability to stimulate and inhibit follicle stimulating hormone (FSH) production, a process critical for spermatogenesis. Blue arrows connect hormones to the cell or gland by which they are secreted. Green arrows indicate stimulatory interactions, and red lines indicate inhibitory interactions.

Analyzing each cohort separately, BEAGLE identified 5343 chromosome segments likely to represent HBD regions (HBDRs) across all samples. We excluded low-level admixture as a spurious source of HBD (Figure S3). Only three of these segments were identified as apparent artifacts induced by large heterozygous deletions (287 kb, 817 kb, and 877 kb in size) and were removed before subsequent analyses. As expected, the distribution of HBD across all samples was L-shaped, with the majority of HBDRs shorter than 1 Mb and a few intermediate and very large events observed (Figure 4b). The largest HBDR identified spanned all of chromosome 2 in an azoospermic individual, indicative of uniparental isodisomy of the entire chromosome. Clinical reports of UPD2 are extremely rare – there are 7 previous reports of UPD2 that have been ascertained through association with an autosomal recessive disorder [49]. In each of these cases a recessive disorder that lead to clinical presentation was identified. There is currently no proof of imprinted genes on chromosome 2 from either mouse or human data. We performed whole exome sequencing on this individual, and using a simple scoring scheme based on functional annotation and population genetic data, identified a homozygous missense mutation of the *INHBB* gene as the most unusual damaging homozygous lesion in the genome of this individual (Figure 5, Text S1). The biology of the *INHBB* gene product strongly implicates this mutation as a causal factor but without additional functional or epidemiological evidence such a conclusion is speculative (Figure 6).

Setting aside this case of UPD2, we found only modest evidence for an enrichment of homozygosity in men with spermatogenic impairment (Figure 4a, Table 3). Our hypothesis was that, if a large percentage of cases of azoospermia were attributable to large-effect autosomal recessive Mendelian mutations, we would see a corresponding increase in the proportion of cases with large values of *F*. The average inbreeding coefficient was numerically higher in each case cohort compared to its matched control cohort (Table 3). We used a logistic regression mixed model framework to test for association between autozygosity and disease, while controlling for population structure, fitting models that treated autozygosity as both a categorical variable (e.g. inbreeding coefficient >6.25%, yes or no) and a continuous variable (*F*, Methods). While the estimated effect of inbreeding on disease risk was positive in every model that we tested, the corresponding odds ratios did not differ significantly from 1 in any version (Table 3). There were fewer than 10 HBD regions shared by 2 or more cases, supporting the model that spermatogenic efficiency has a polygenic basis. We also tested for case-specific aggregation of HBD segments using the same association framework as that used for CNVs. We did not identify any significant patterns. Based on published analyses of small-effect recessive risk mutations in other complex diseases, we believe our current sample size would be underpowered to detect association between very old inbreeding (e.g. due to shared ancestors 15 generations ago). It is possible that large cohorts, consisting of over 10,000 cases, may be needed to accurately estimate the relationship between low-level variation in inbreeding (*F* values smaller than 0.1) and azoospermia risk, as well as map specific risk alleles [27], [50].

**Table 3:** Summary of inbreeding coefficient estimates across cohorts, and association testing.

			*F*>0.5%	*F*>1.6%	*F*>6.25%	All *F*
Cohort	Type	Average *F*	# samples	# samples	# samples	# samples
Porto	Case	0.0069	39	21	5	175
Spain	Control	0.0042	112	41	8	622
Utah	Case	0.0020	5	3	1	84
Utah	Control	0.0014	0	0	0	59
WUSTL	Case	0.0027	6	0	0	70
WUSTL	Control	0.0020	1	0	0	99
Effect			OR 1.25 (95% CI = [0.81–1.92])	OR 1.62 (95% CI = [0.88–2.98])	OR 1.18 (95% CI = [0.34–4.03])	β = 8.23 (95% CI = [1.92–14.54])
p value			0.31	0.12	0.794	0.19

For each case and control group we present the average the estimated inbreeding coefficient and the number of individuals with inbreeding coefficients above a specified threshold. The last column indicates the total number of individuals in each group. The bottom two rows indicate the results of an association test between inbreeding and case/control status using either a categorical variable as a definition of inbreeding status (*F*>0.5%, *F*>1.6%, and *F*>6.25%) or using the inbreeding coefficient as a continuous variable (“All *F*”).

## Discussion

We report here the largest whole genome study to date investigating the role of rare variants in infertility, examining data from 323 cases of male infertility and 1,136 controls. These data demonstrate that rare CNVs are a major risk factor for spermatogenic impairment, and while confirming the central role of the Y chromosome in modulating spermatogenic output, our risk estimates for autosomal and X-linked CNVs indicate that this phenotype is influenced by rare variation across the entire genome. The controls from two of the cohorts were unphenotyped, and given the estimated prevalence of azoospermia (1%), we may have underestimated the risk associated with these large rearrangements.

We observed 5 deletions of *DMRT1* coding sequence in cases and none in over 7,000 controls. These deletions ranged in size from 54 kb to over 2 Mb (Table 4). *DMRT1* is situated in a region of chromosome 9p that has been identified as a source of syndromic and non-syndromic forms of XY gonadal dysgenesis (GD). The deletions of this region that are associated with syndromic forms of GD are usually 4–10 Mb in size, while isolated GD has been reported for deletions smaller than 1 Mb [40], [51], [52]. Despite frequent involvement of *DMRT1* in these putative causal mutations, there is variability in both the phenotypic outcome affiliated with each deletion and the extent of *DMRT1* coding sequence contained therein. At least two cases of GD have been linked to deletions near but not overlapping *DMRT1* – one 700 kb mutation 30 kb distal to *DMRT1* in a case of complete XY GD that was inherited from an apparently normal mother, and a second 260 kb *de novo* deletion about 250 kb distal to *DMRT1* [39], [40]. Both of these deletions overlapped the genes *KANK1* and *DOCK8*. On the other hand, two smaller deletions, one a 25 kb deletion of *DMRT1* exons 1 and 2, and one a 35 kb deletion of exons 3 and 4, have been observed in patients with complete GD and bilateral ovotesticular disorder of sexual development, respectively [51], [52]. Based on the clinical records of patients in our current study, there is no chance that our *DMRT1* deletion carriers could represent misdiagnosis of a condition as severe as complete XY GD, which presents with the appearance of female genitalia. Indeed, two of our *DMRT1* deletion carriers were subject to testicular biopsies. Our observations here suggest that hemizygous deletion of *DMRT1* is a lesion that shows variable expressivity that may depend on the sequence of the undeleted *DMRT1* allele, variation in other sequences on chromosome 9p, and the state of other factors in the pathways regulating testicular development and function. Strictly speaking, statements that hemizygous deletions of *DMRT1* are “sufficient” to cause GD or spermatogenic failure need to be qualified at this point until we gain a better understanding of the effects of genetic background. For instance, in most studies of *DMRT1* deletion, the undeleted *DMRT1* allele is rarely sequenced. Is the mode of action dominant or recessive?

**Table 4:** *DMRT1* deletions detected by array in the current study.

Case	Start (bp)	End (bp)	Platform	DMRT1 Exons	Phenotype
U841_A	845091	994958	Illumina 370K	3,4,5	Azoo - SCOS
U162_A	853635	994958	Illumina 370K	3,4,5	Azoo - MA
F1060	861888	916779	Affymetrix 6	3,4	Azoo
F5031	30911	1972069	Affymetrix 6	All	Azoo
F3407	30911	1170987	Affymetrix 6	All	Azoo

‘DMRT1 Exons’ – exons contained within each deletion, numbered from the 5′ to 3′ position. SCOS – Sertoli Cell Only Syndrome; MA – maturation arrest. Deletion coordinates given with respect to NCBI36.

Deletions of the Y chromosome have long been appreciated as a cause of azoospermia, and we have now shown here that Y-linked duplications are also significant risk factors for spermatogenic failure. The precise definition of the duplication sensitive sequences awaits further investigation. Historically, Y duplications have been much less studied than Y deletions, as +/− STS PCR is the standard assay for assessing Y chromosome copy number variation in both the clinical and research setting. Quantitative PCR methods for measuring Y chromosome gene dosage have been described in the literature, and applied almost exclusively to studying the phenotypic effects of duplication of genes in the AZFc region [53]. Results of these investigations are conflicting, with studies of Europeans reporting no association between AZFc partial duplication and spermatogenic impairment [54], while reproducible associations have been reported in east Asian cohorts [55], [56]. Notably, we identified some duplications on the Y chromosome greater than 2.5 Mb in size, all spanning the AZFc locus (Figure S6), in 8/179 cases (those typed on Affymetrix 6.0), compared to 13/972 controls (OR 3.45 [1.21–9.12], p<0.01). Rearrangements of this size on the autosomes confer staggering risk for other forms of disease; for example, by one recent estimate CNVs larger than 3 Mb have an OR of 47.7 for intellectual disability and/or developmental delay [46]. Our results suggest that Y chromosome structure may be more dosage sensitive than previously appreciated, and we speculate that some genes and non-coding sequences of the Y chromosome may be under stabilizing selection for copy number [57].

Three recent studies have used array-based approaches to characterize CNVs in men with azoospermia. Our finding of an X-linked CNV burden in men with spermatogenic failure has been replicated and described elsewhere [58]. In a second study, Tuttelmann *et al.* evaluated 89 severe oligozoospermic, 37 azoospermic, and 100 normozoospermic control men using Agilent 244K and 400K arrays and identified a number of CNVs potentially involved in male infertility [24]. Third, Stouffs *et al.* assayed nine azoospermic men and twenty control samples using the 244K array and followed-up CNVs of interest by q-PCR in up to 130 additional controls [25]. Using the criterion of at least 51% reciprocal overlap, we have identified a number of CNVs in the current study that overlap with case-specific CNVs in the Tuttelmann and Stouffs studies. The majority of these CNVs appear to be relatively common polymorphisms and not case-specific in our larger dataset; however several noteworthy CNVs overlap between studies and are absent, or are present at a very low frequency in controls. For example, Tuttelmann *et al.* identified a private duplication on Xq22.2 in an oligozoospermic man [24], and we identified an overlapping duplication in an oligozoospermic man from the present study (ChrX:103065826–103205985, NCBI36). These duplications alter the copy number of a small number of testis-specific or testis-expressed variants of histone 2B (H2BFWT, H2BFXP, H2BFM). No CNVs in this region were identified in more than 1600 controls. Tuttelmann *et al.* also identified an azoospermic man with a deletion and another with a duplication on 8q24.3, encompassing the genes *PLEC1* and *MIR661* [24]. We identified an oligozoospermic man with a duplication of the same region, affecting the same functional elements (chr8:145064091–145118650, NCBI36). CNVs of this locus are very rare, with a frequency of about 0.005% in our controls and 0.0025% in controls used for a recent study of developmental delay [46]. It is important to note that new variants will frequently be discovered whenever a discovery technology such as array CGH is applied to a new sample set, and the observation that a variant is patient-specific is not in itself remarkable, especially when one is investigating very small sample sizes.

Our observation of low deletion HI scores in cases raises a number of considerations for future studies of the genetics of spermatogenic impairment. We interpret low HI scores in cases as evidence against a widespread role for dominant, highly penetrant deletions in spermatogenic failure. It is possible that our case recruitment, which pre-screened for normal karyotype, may have removed all large HI score events; however our identification of two large HI deletions of *WT1* and *MAPK1* indicate otherwise (Figure 3). A second concern is that the data used to train the haploinsufficiency prediction algorithm is in part based on features of deletions known to cause dominant pediatric disease, and that an analogous approach trained on fertility phenotypes may lead to different conclusions. There are few examples of dominant loss-of-function mutations causing isolated infertility in humans and only 5 of the >200 mouse infertility mutants described in a previous review showed a phenotype in heterozygous form [45], so fitting a model of a dominant infertility mutation may be challenging in the short term. Nonetheless, developing disease-specific pathogenicity scores for infertility phenotypes should be a priority.

Despite the differences between the genetic signatures of spermatogenic impairment and severe developmental disease noted above, there are connections in their epidemiology. Recent results estimate a 9.9% rate of birth defects in children conceived by intracytoplasmic sperm injection (ICSI), the technology typically employed for assisting cases of severe male factor infertility, which is an OR of 1.77 compared to unassisted reproduction [59]. Among several possible explanations for this finding, our data raise the possibility that mutations that compromise gonadal function may act pleiotropically to disrupt development in other tissues. A better understanding of the genetic basis of male infertility is urgently needed in order to improve risk assessment for couples considering assisted reproduction.

Clinical genomics is a paradigm in need of robust applications, and our finding of a large CNV burden in cases suggest that some infertility mutations may have the high penetrance required for clinical utility. Indeed some mutation screens are already used clinically in the management of male infertility. Although the presence of azoospermia can be easily assessed using a standard laboratory test, many men with azoospermia will have sperm production within the testis and be candidates for testicular sperm retrieval. We have already identified that the specific AZF deletion (a, b or b/c) has a dramatic effect on the prognosis of sperm retrieval (vs. AZFc-deleted males) [60]. In the present study, we have identified deletion of *DMRT1* coding sequence as a genetic event that appears highly predictive of spermatogenic failure. In depth characterization of carriers is now needed to understand how this mutation affects the prognosis of sperm retrieval. Similar whole genome tests may provide critical prognostic information that can help to characterize the chance of successful treatment for couples with non-obstructive azoospermia, avoiding expensive and needlessly invasive interventions, while potentially providing guidance for new therapeutic interventions.

## Methods

### Ethics statement

All DNA samples used in this study were derived from peripheral blood lymphocytes collected from individuals giving IRB-approved informed consent. The following IRBs were involved: INSA Ethics Committee and Hospital Authority (Portugal), University of Utah IRB, and Washington University in St. Louis IRB (#201107177). All samples of genomic DNA to be analysed in this study i) belong to DNA banks that have been established throughout the years; ii) are coded; and iii) each individual has signed a declaration of informed consent before donating his genomic DNA for analysis, authorizing molecular studies to be performed with this material.

### Patient cohorts

All cases were deemed idiopathic following a standard clinical workup, which included screening for Y chromosome deletions. Controls from the Utah cohort were men with normal semen analysis, remaining controls were not phenotyped on semen quality. Full details of the source and diagnosis of samples in this study are available in Supplemental Methods. When using SNP arrays, CNV analysis is more sensitive to experimental noise than SNP genotyping, and we used different sample QC metrics to inform CNV and SNP stages of our project. As a result, we have slightly larger sample sizes for the HBD analyses than for the CNV analyses.

### Population structure

The individuals studied here were sourced from diverse geographic locations (Table 1, Text S1). All primary samples (e.g. 323 cases and 1133 control samples subjected to whole-genome genetic analysis) were of self-reported Caucasian ancestry, but it was necessary to take additional steps to control for population structure in all aspects of the analysis. First, genetic ancestry of each sample was assessed by principal components analysis and ethnicity outliers were removed (Figure S2, Figure S3). Second, eigenvectors generated by this principal components analysis were used as covariates in both CNV association and inbreeding coefficient association analyses. For analyses focusing on the Y chromosome, we performed analyses conditioning on Y haplogroup to provide the most stringent possible correction for population structure with available data. Lastly, we conducted alternate association analyses with the Porto case cohort using a smaller, but more geographically proximal Spanish control cohort (Figure S5).

### Identification of CNVs, regions of homozygosity-by-descent

Three array platforms were used for CNV discovery: Illumina 370K (Utah), Illumina OmniExpress (Washington University), and Affymetrix 6.0 (Porto, Cornell, Nanjing). Full details of sample processing and array experiments are available in Supplemental Methods. Three CNV calling algorithms were used to generate CNV maps for each individual typed with Illumina technology: GADA, a sparse Bayesian learning approach [61]; PennCNV, a Hidden Markov Model (HMM)-based method originally designed for the Illumina platform [62]; and QuantiSNP 2.0, another HMM-based method for Illumina [63]. CNVs called by 2 of 3 algorithms were retained for analysis. CNV calling for Affymetrix 6.0 was performed with Birdsuite [64]. Due to the complexity of calling CNVs on the sex chromosomes, for all array datasets we implemented a bespoke normalization and calling procedure that used only the GADA algorithm to call CNVs from the X and Y chromosomes. For full details of CNV calling see Supplemental Methods.

Regions of homozygosity-by-descent (HBD) were identified using BEAGLE 3.0 [48]. SNPs with no-call rates >5% were removed prior to HBD analysis. As BEAGLE uses a model for background linkage disequilibrium that is fit from the data, cases and controls from each cohort were analyzed simultaneously and separately to assess cohort-specific biases in calling HBD. Prior to downstream analysis, we identified and removed a small number of reported HBD regions that corresponded to rare, large hemizygous deletions.

Inbreeding coefficients for each individual were calculated from their HBD data using the formula:
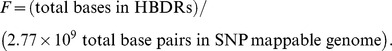



### CNV and HBD association analyses

Due to differences in array content, CNV frequencies were determined on a per-platform basis. All CNV calls made on a given platform, in both cases and controls, were combined into CNV regions using a threshold of 50% reciprocal overlap to defined two events as the same ([35]). We defined the CNV frequency as the proportion of all samples (cases and controls) containing that CNV.

We constructed several statistical tests to measure differences between cases and controls. We used Mann-Whitney U tests to test for differences in the total amount of aneuploid sequence per genome. We used standard logistic regression to test for CNV load on chromosome compartments (e.g. the autosomes, X chromosome) and a small number of functional features (genes, miRNA, etc). To control for population structure these models included the first 10 principal components from PCA analysis of the SNP genotype data from all cohorts (Figure S2). We used a permutation strategy for genomewide, locus-by-locus testing for association at all genes and in 500 kb non-overlapping genomic windows. The permutation strategy, implemented with the software package PLINK, calculates nominal and genomewide p-values by permuting case-control labels [65]. To present consistent summaries of CNV burden for the entire study (all cohorts combined), we used linear mixed-effects logistic regression, treating cohort as a random factor and compared these to effect size estimates for each cohort separately using standard logistic regression (Figure 1). The mixed effects modeling framework controls for SNP platform as each case-control cohort was typed on a different platform; a similar use of mixed-effect modeling was recently described in a meta-analysis of schizophrenia SNP data [27].

Analogous tests were conducted on HBD segments from the original discovery cohort and the combined primary and replication datasets.

### Validation assay

We performed validation and replication analyses of *DMRT1* deletions with and assay based on Taqman PCR. Copy number was assessed using a pre-designed assay #Hs06833797_cn within the *DMRT1* gene against an RNase P reference (assay # 4403326; both assays from Applied Biosystems, Carlsbad, CA, USA) according to manufacturer's recommendations.

## Supporting Information

Dataset S1 Images of the normalized intensity data for all CNV calls in the Utah case-control cohort >100 kb in size. For each CNV, we have plotted the Log R Ratio (vertical lines) and B Allele Frequency (black points) of all probes within the CNV, as well as an equal number of probes 5′ and 3′ to the edges of the CNV. The Log R Ratio for probes within “gain” CNV calls are colored green, within “loss” CNV calls are colored red, and outside of a CNV call are colored grey. The sample ID and number of probes in the CNV call are listed above each image.
(PDF)

Figure S1 QC of Affymetrix callsets. Summary plots of array QC for the case samples and NBS control samples. There is an expected inverse correlation between the noise in the data (measured by the median absolute deviation (MAD) of the probe intensities) and the number of calls made in a particular experiment. We fit a linear model to these parameters separately for cases (A) and NBS controls (C), and samples >4 MADs from the fitted model were removed (circled dots). We also implemented an analogous QC step using the ratio of deletions/duplication calls per sample and number of calls per sample, separately for cases (B) and NBS controls (D), . In all plots, arrays are colored by their spatial autocorrelation function (a measure of “waviness”). Distributions of post-QC statistics are highly similar between cases and controls.
(TIF)

Figure S2 Principal components analysis (PCA) of population structure in all case cohorts post-QC. For each cohort, samples were analyzed together with HapMap samples using the EIGENSOFT package [69]. (A) Utah (B) WUSTL batch 1, (C) WUSTL batch 2, (D) Porto. Eigenvector loadings for cases and controls (A,B,C) or cases (D) are plotted as red crosses, while HapMap samples are plotted as other colored symbols described in each legend.
(TIF)

Figure S3 Analysis of population structure in the Porto cohort after sample QC. Based on the results of PCA analysis in Figure S2D, which indicate that the Portuguese population may have subtle differences in allele frequencies from northern European populations, we further investigated the possibility of population structure as a confounder. No significant correlation was observed between the estimated amount of African ancestry in each Porto case and (A) the total number of deletion calls or (B) the total number of rare deletions (here defined as <5% frequency). In both cases smaller (more negative) eigenvector loadings (x-axis) indicate a larger degree of African admixture. We segmented the genome of each sample into regions with 0, 1 or 2 chromosomes of African ancestry using the program HapMix [70]. (C) The percent ancestry inferred by HapMix in this way correlated well with PCA based ancestry estimates. (D) The extent of African ancestry in each case, as estimated by EIGENSTRAT, was uncorrelated (R = 0.002) with the fraction of the genome contained in a homozygous-by-descent region (a rough measure of the inbreeding coefficient), indicating that variation in distant African ancestry was not a major confounder of the HBD analyses.
(TIF)

Figure S4 Analysis of batch effects in Porto cases. The Porto arrays were run over a period of several months. Here we plot the total number of CNV calls per array, as a function of run order (sample number 1 = first array run, sample number 162 = last array run). Each dot is colored based on run date. No obvious outlier batches are visible. There was a small but insignificant trend for fewer CNV calls on later run dates (least-squares regression line is plotted).
(TIF)

Figure S5 CNV burden statistics using a Spanish control cohort. In the primary CNV analyses described in the main text, we use a Caucasian population from the United Kingdom as a control group for the Porto azoospermia cohort. Here, we address the effect of using a control group that is more closely matched on genetic ancestry. We performed the same burden analyses depicted in Figure 1 of the main text, this time using a much smaller control cohort of 368 Caucasian men ascertained in Spain. We used logistic regression to estimate the influence of copy number variants (CNVs) on the odds of being diagnosed with impaired spermatogenesis in three case-control cohorts. Eigenvectors from a principal components analysis were used as covariates as before. The odds ratio estimated from fitting a logistic regression model of total CNV count to disease status is plotted separately for each cohort, as well as the combined set of all cohorts (black points). Cohort 1 = Utah (Illumina 370K), 2 = Porto and Weill Cornell (Affymetrix 6.0), 3 = WUSTL (Illumina OmniExpress). Sample sizes used in CNV analysis are n = 83 cases and n = 62 controls for cohort 1, n = 179 cases and 368 controls for cohort 2, and n = 61 cases and 100 controls for cohort 3. Conclusion: While the direction of burden effect for rare autosomal deletions, X-linked deletions, and Y duplications was the same as seen with the analysis using UK controls, only the rare autosomal deletion burden model shows statistical evidence for an odds ratio greater than 1.
(TIF)

Figure S6 CNVs on the Y chromosome. (A) Our strongest statistical association involved gains and losses of a 200 kb tandem repeat termed *DYZ19*, approximately 500 kb distal to palindrome P4. Here are plotted 6 deletions from the UT cohort, which were evenly distributed between azoospermic and oligozoospermic men. (B) In the Weill-Cornell cohort, a small group of azoospermic individuals ascertained at a tertiary care clinic, we identified a number of classical AZF deletions, as well as duplications of AZFc. Next to each CNV is listed the sample ID and Y haplogroup of the sample inferred from SNP data (Methods). These data demonstrate that existing SNP platforms can cleanly identify Y chromosome rearrangements involving both gain and loss of sequence, and will facilitate investigation of the full spectrum of Y chromosome variation in future studies of male infertility. Notably, we observed complex patterns of copy number change in some samples that highlight the challenge of interpreting array data mapped to a single reference Y chromosome (haplogroup R1). In both panels, for each individual, deviations of probe log_2_ ratios from 0 are depicted by grey lines or black dots, and probes spanning CNV calls are colored as either red (losses) or green (gains).
(TIF)

Figure S7 CNV calls made in all array datasets at the Xp11 rearrangement hotspot. This plot is the same as Figure 2a, with the addition of tracks containing deletion and duplication calls from a Spanish male control cohort assayed on the Affymetrix 6.0 platform.
(TIF)

Figure S8 Left, example STS PCR validation of Xp11 in one case from Cornell (F10) and two controls. Right, the same assay, run in multiplex (Xp11 and B-globin reactions in the same tube) for 5 WUSTL case carriers and two controls. Note the presence of the smaller beta-globin band in the two control individuals in lanes 7 and 8. The primer sequences for the Xp11 deletion assay and a control locus are given in the Text S1.
(TIF)

Figure S9 CNV calls made at the DYZ19 tandem repeat locus. CNV calls made on the Utah, Porto, and WUSTL case cohorts, and the Utah, NBS (WTCCC), WUSTL and Spanish control cohorts.
(TIF)

Figure S10 qPCR validation of DMRT1 deletions in the Utah Cohort. (A) Histogram of mean probe intensities from Illumina 370K array spanning the DMRT1/DMRT3 deletion locus (chr9:845901–994958 bp). N = 148 samples are plotted. (B) Taqman validation results for the *DMRT1* locus from 30 of the samples screened by 370K array in panel A (y-axis), including the two deletion carriers (red points) and one duplication carrier (green point) identified from Illumina 370K intensity data (x-axis).
(TIF)

Figure S11 CNVs on chromosome 9p in the Nanjing cohort. Affymetrix 6.0 data was generated on a cohort of 979 idiopathic NOA cases and ethnicity-matched controls 1734 controls recruited primarily from the cities of Nanjing and Wuhan, China. CNVs were called using the identical pipeline as the other cohorts. Plotted above are all of the deletions (red) and duplications (green) observed in these cases (top) and controls (bottom).
(TIF)

Figure S12 Detection of additional intronic *DMRT1* deletions using the TaqMan validation assay. As described in the supplemental methods, we screened 5 plates of DNA from Weill Cornell cases and 2 plates of Caucasian male controls using the *DMRT1* TaqMan validation assay. Each sample was assayed in quadruplicate (although some samples were assayed in duplicate or triplicate if insufficient DNA was available). We applied extremely stringent calling criteria to these data, excluding samples with (1) low DNA content as defined by picogreen assay (2) high standard deviation (>0.3) of delta CT measurements across replicates (3) low copy number confidence scores generated by CopyCaller software. Each point in the panels above represents the average delta CT for the control locus (VIC) and *DMRT1* (FAM) for a single sample. Red dots indicate deletion carrier calls. We detected 2 deletions in 233 case samples, a frequency of 0.86%, and 1 deletion in 135 controls (0.74%). As this assay is targeting intronic sequence, and we have not cloned the breakpoints the functional consequences of each deletion is unclear.
(TIF)

Figure S13 Controlling for population structure while testing for association between inbreeding and infertility. As described in Figure S2 and in Text S1, we used principal components analysis to assess population structure in our case and control cohorts. We use the BEAGLE software package to define homozygous-by-descent regions (HBDRs) of each sample in our study, and used these HBDRs to estimate corresponding inbreeding coefficients. We tested for association between inbreeding coefficient and the probability of azoospermia using a linear model. On the left, we show that there is strong association when analyzing data from the Porto cases and NBS Wellcome Trust case-control consortium controls (CCC controls) in a simple model that does not account for population structure (“No EV”). When including the first 10 eigenvectors from a PCA analysis (“EV”), the point estimate of association remains somewhat inflated but is no longer significant. On the right, we show the same analysis performed on the Porto cohort with a more closely matched control population from Spain, with clearly smaller confidence intervals and smaller point estimates of effect size. In order to be as conservative as possible, we report results of inbreeding analysis using the Spanish control cohort in the main text.
(TIF)

Table S1 The results of CNV burden tests performed on the Utah cohort. Each subtable contains summary statistics for a different subset of CNVs, selected by size and/or frequency.
(XLSX)

Table S2 The results of CNV burden tests performed on the Porto cohort. Each subtable contains summary statistics for a different subset of CNVs, selected by size and/or frequency.
(XLSX)

Table S3 The results of CNV burden tests performed on the Washington University cohort. Each subtable contains summary statistics for a different subset of CNVs, selected by size and/or frequency.
(XLSX)

Text S1 A description of patient cohorts, supplementary methods and analyses.
(DOCX)
