# Supplementary material for: Human Spermatogenic Failure Purges Deleterious Mutation Load from the Autosomes and Both Sex Chromosomes, including the Gene DMRT1
Source: PLoS Genet. 2013 Mar 21;9(3):e1003349. doi: 10.1371/journal.pgen.1003349 (PMC3605256; doi:10.1371/journal.pgen.1003349)
Supplement: Dataset S1 — Images of the normalized intensity data for all CNV calls in the Utah case-control cohort >100 kb in size. For each CNV, we have plotted the Log R Ratio (vertical lines) and B Allele Frequency (black points) of all probes within the CNV, as well as an equal number of probes 5′ and 3′ to the edges of the CNV. The Log R Ratio for probes within “gain” CNV calls are colored green, within “loss” CNV calls are colored red, and outside of a CNV call are colored grey. The sample ID and number of probes in the CNV call are listed above each image. (PDF) [file pgen.1003349.s001.pdf]

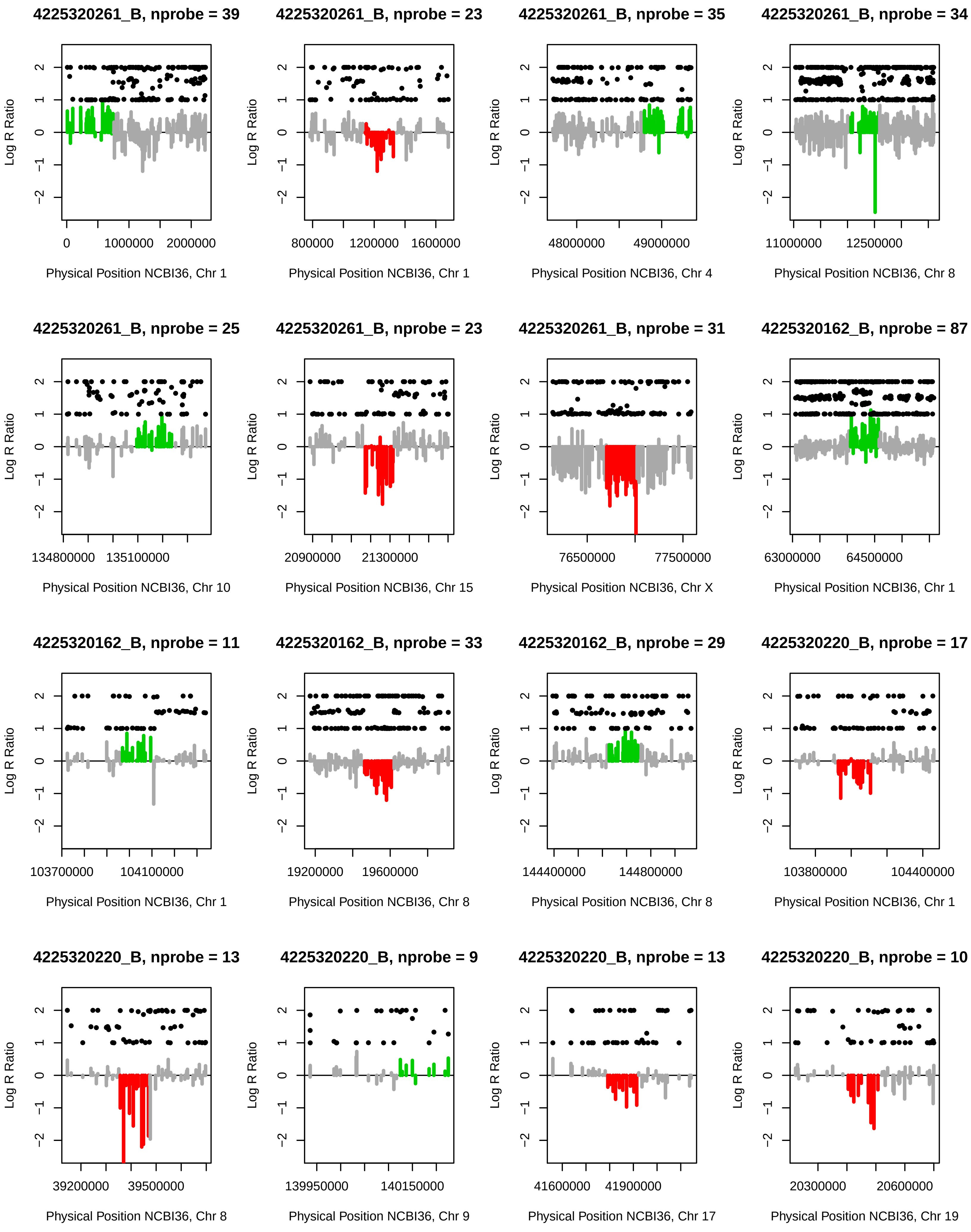
4225320261_B, nprobe = 39
Log R Ratio
2
1
0
−1
−2
0
1000000
2000000
Physical Position NCBI36, Chr 1
4225320261_B, nprobe = 23
800000
1200000
1600000
Physical Position NCBI36, Chr 1
4225320261_B, nprobe = 35
48000000
49000000
Physical Position NCBI36, Chr 4
4225320261_B, nprobe = 34
11000000
12500000
Physical Position NCBI36, Chr 8
4225320261_B, nprobe = 25
134800000
135100000
Physical Position NCBI36, Chr 10
4225320261_B, nprobe = 23
20900000
21300000
Physical Position NCBI36, Chr 15
4225320261_B, nprobe = 31
76500000
77500000
Physical Position NCBI36, Chr X
4225320162_B, nprobe = 87
63000000
64500000
Physical Position NCBI36, Chr 1
4225320162_B, nprobe = 11
103700000
104100000
Physical Position NCBI36, Chr 1
4225320162_B, nprobe = 33
19200000
19600000
Physical Position NCBI36, Chr 8
4225320162_B, nprobe = 29
144400000
144800000
Physical Position NCBI36, Chr 8
4225320220_B, nprobe = 17
103800000
104400000
Physical Position NCBI36, Chr 1
4225320220_B, nprobe = 13
39200000
39500000
Physical Position NCBI36, Chr 8
4225320220_B, nprobe = 9
139950000
140150000
Physical Position NCBI36, Chr 9
4225320220_B, nprobe = 13
41600000
41900000
Physical Position NCBI36, Chr 17
4225320220_B, nprobe = 10
20300000
20600000
Physical Position NCBI36, Chr 19

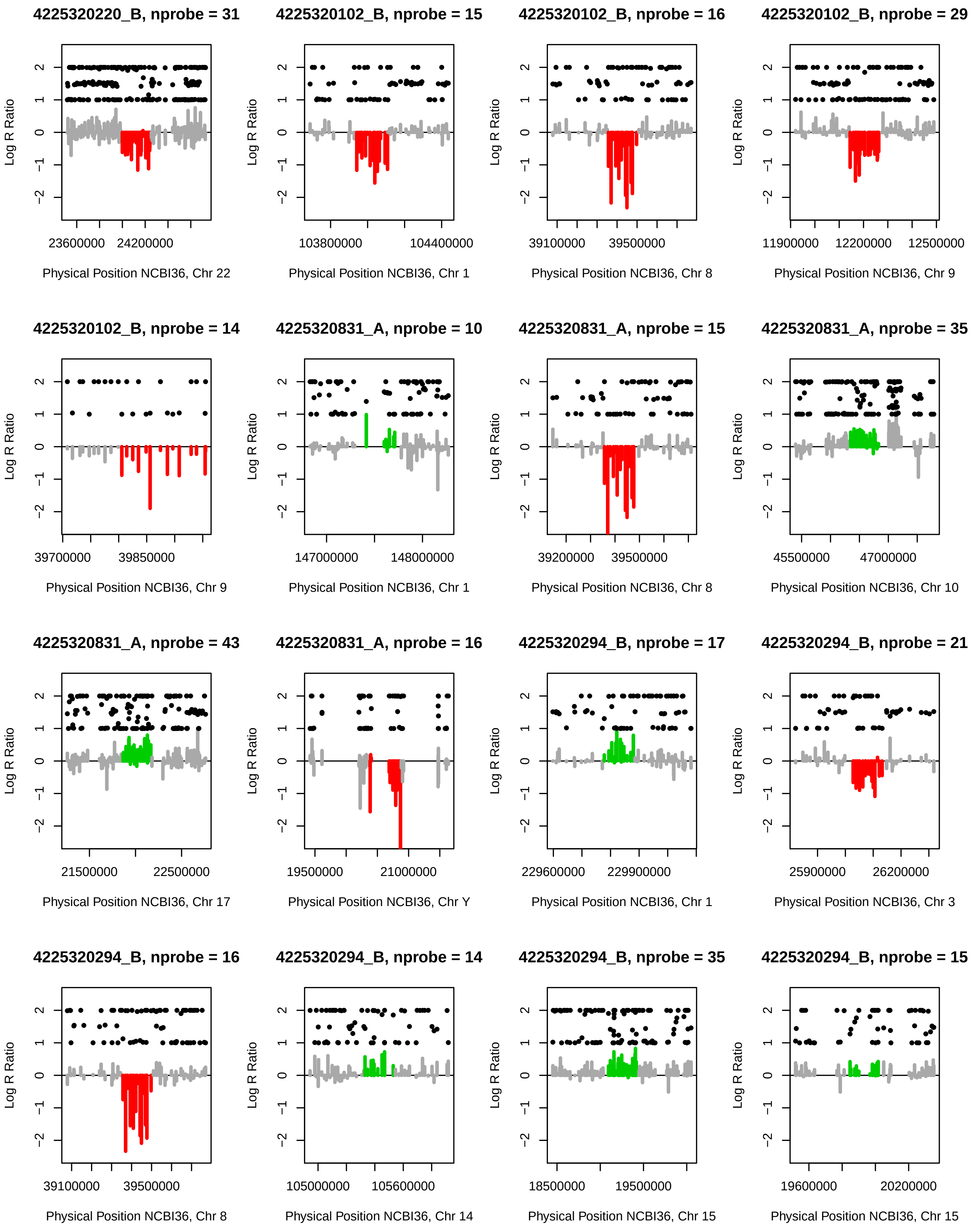

4225320220_B, nprobe = 31
Log R Ratio
Physical Position NCBI36, Chr 22
4225320102_B, nprobe = 15
Physical Position NCBI36, Chr 1
4225320102_B, nprobe = 16
Physical Position NCBI36, Chr 8
4225320102_B, nprobe = 29
Physical Position NCBI36, Chr 9
4225320102_B, nprobe = 14
Physical Position NCBI36, Chr 9
4225320831_A, nprobe = 10
Physical Position NCBI36, Chr 1
4225320831_A, nprobe = 15
Physical Position NCBI36, Chr 8
4225320831_A, nprobe = 35
Physical Position NCBI36, Chr 10
4225320831_A, nprobe = 43
Physical Position NCBI36, Chr 17
4225320831_A, nprobe = 16
Physical Position NCBI36, Chr Y
4225320294_B, nprobe = 17
Physical Position NCBI36, Chr 1
4225320294_B, nprobe = 21
Physical Position NCBI36, Chr 3
4225320294_B, nprobe = 16
Physical Position NCBI36, Chr 8
4225320294_B, nprobe = 14
Physical Position NCBI36, Chr 14
4225320294_B, nprobe = 35
Physical Position NCBI36, Chr 15
4225320294_B, nprobe = 15
Physical Position NCBI36, Chr 15

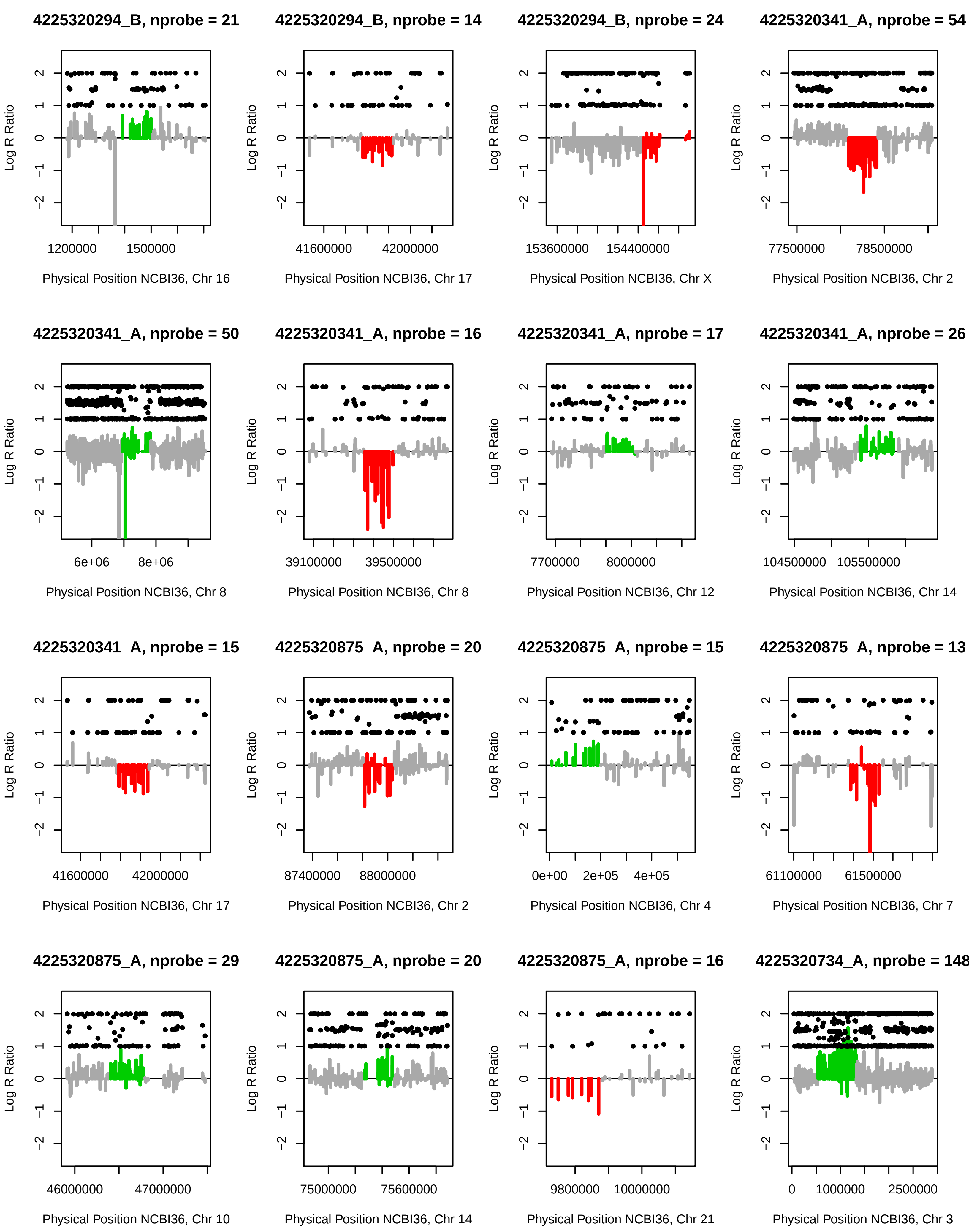

4225320294_B, nprobe = 21
Log R Ratio
2
1
0
−1
−2
1200000
1500000
Physical Position NCBI36, Chr 16
4225320294_B, nprobe = 14
Log R Ratio
41600000
42000000
Physical Position NCBI36, Chr 17
4225320294_B, nprobe = 24
Log R Ratio
153600000
154400000
Physical Position NCBI36, Chr X
4225320341_A, nprobe = 54
Log R Ratio
77500000
78500000
Physical Position NCBI36, Chr 2
4225320341_A, nprobe = 50
Log R Ratio
6e+06
8e+06
Physical Position NCBI36, Chr 8
4225320341_A, nprobe = 16
Log R Ratio
39100000
39500000
Physical Position NCBI36, Chr 8
4225320341_A, nprobe = 17
Log R Ratio
7700000
8000000
Physical Position NCBI36, Chr 12
4225320341_A, nprobe = 26
Log R Ratio
104500000
105500000
Physical Position NCBI36, Chr 14
4225320341_A, nprobe = 15
Log R Ratio
41600000
42000000
Physical Position NCBI36, Chr 17
4225320875_A, nprobe = 20
Log R Ratio
87400000
88000000
Physical Position NCBI36, Chr 2
4225320875_A, nprobe = 15
Log R Ratio
0e+00
2e+05
4e+05
Physical Position NCBI36, Chr 4
4225320875_A, nprobe = 13
Log R Ratio
61100000
61500000
Physical Position NCBI36, Chr 7
4225320875_A, nprobe = 29
Log R Ratio
46000000
47000000
Physical Position NCBI36, Chr 10
4225320875_A, nprobe = 20
Log R Ratio
75000000
75600000
Physical Position NCBI36, Chr 14
4225320875_A, nprobe = 16
Log R Ratio
9800000
10000000
Physical Position NCBI36, Chr 21
4225320734_A, nprobe = 148
Log R Ratio
0
1000000
2500000
Physical Position NCBI36, Chr 3

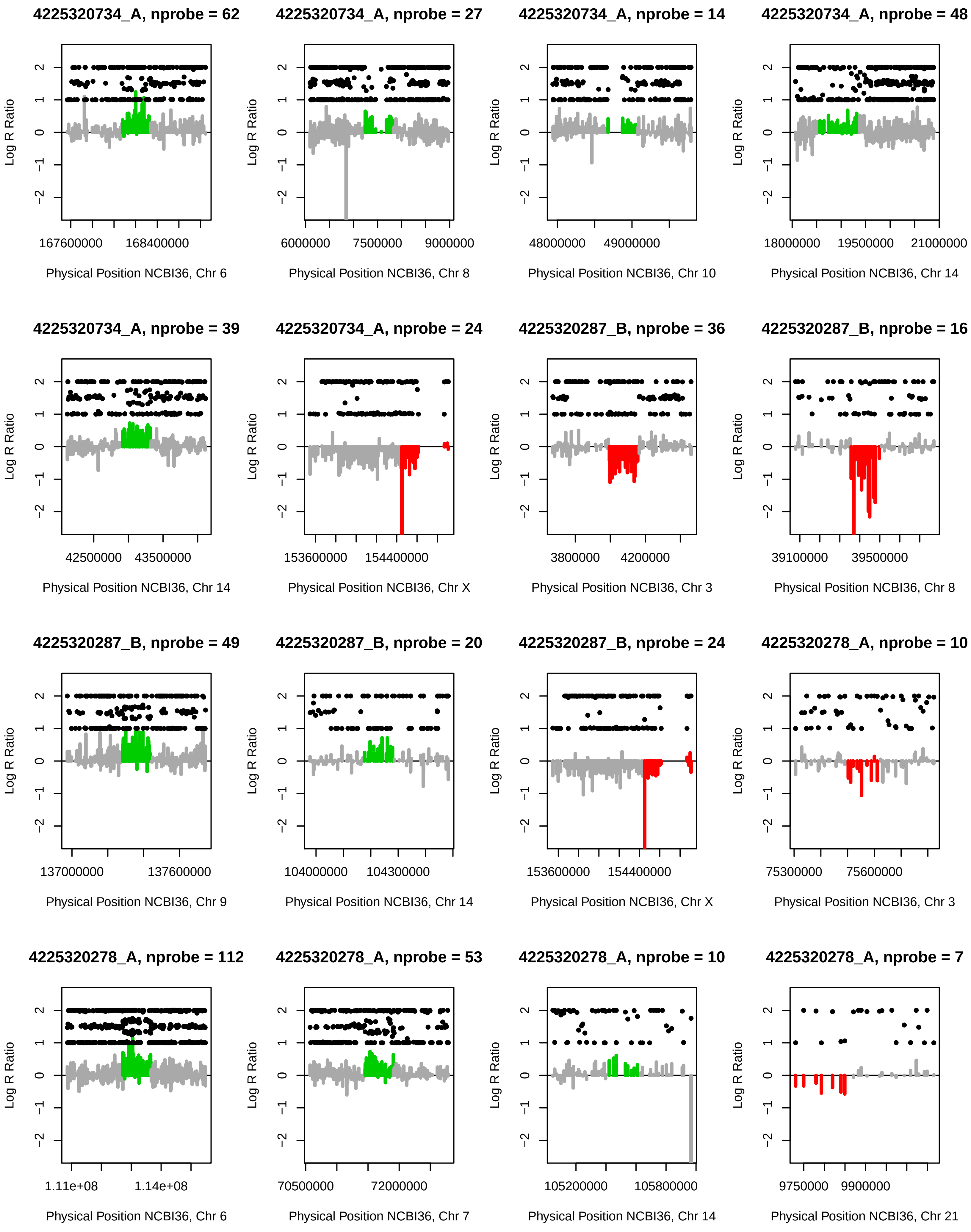
4225320734_A, nprobe = 62
Log R Ratio
Physical Position NCBI36, Chr 6
4225320734_A, nprobe = 27
Log R Ratio
Physical Position NCBI36, Chr 8
4225320734_A, nprobe = 14
Log R Ratio
Physical Position NCBI36, Chr 10
4225320734_A, nprobe = 48
Log R Ratio
Physical Position NCBI36, Chr 14
4225320734_A, nprobe = 39
Log R Ratio
Physical Position NCBI36, Chr 14
4225320734_A, nprobe = 24
Log R Ratio
Physical Position NCBI36, Chr X
4225320287_B, nprobe = 36
Log R Ratio
Physical Position NCBI36, Chr 3
4225320287_B, nprobe = 16
Log R Ratio
Physical Position NCBI36, Chr 8
4225320287_B, nprobe = 49
Log R Ratio
Physical Position NCBI36, Chr 9
4225320287_B, nprobe = 20
Log R Ratio
Physical Position NCBI36, Chr 14
4225320287_B, nprobe = 24
Log R Ratio
Physical Position NCBI36, Chr X
4225320278_A, nprobe = 10
Log R Ratio
Physical Position NCBI36, Chr 3
4225320278_A, nprobe = 112
Log R Ratio
Physical Position NCBI36, Chr 6
4225320278_A, nprobe = 53
Log R Ratio
Physical Position NCBI36, Chr 7
4225320278_A, nprobe = 10
Log R Ratio
Physical Position NCBI36, Chr 14
4225320278_A, nprobe = 7
Log R Ratio
Physical Position NCBI36, Chr 21

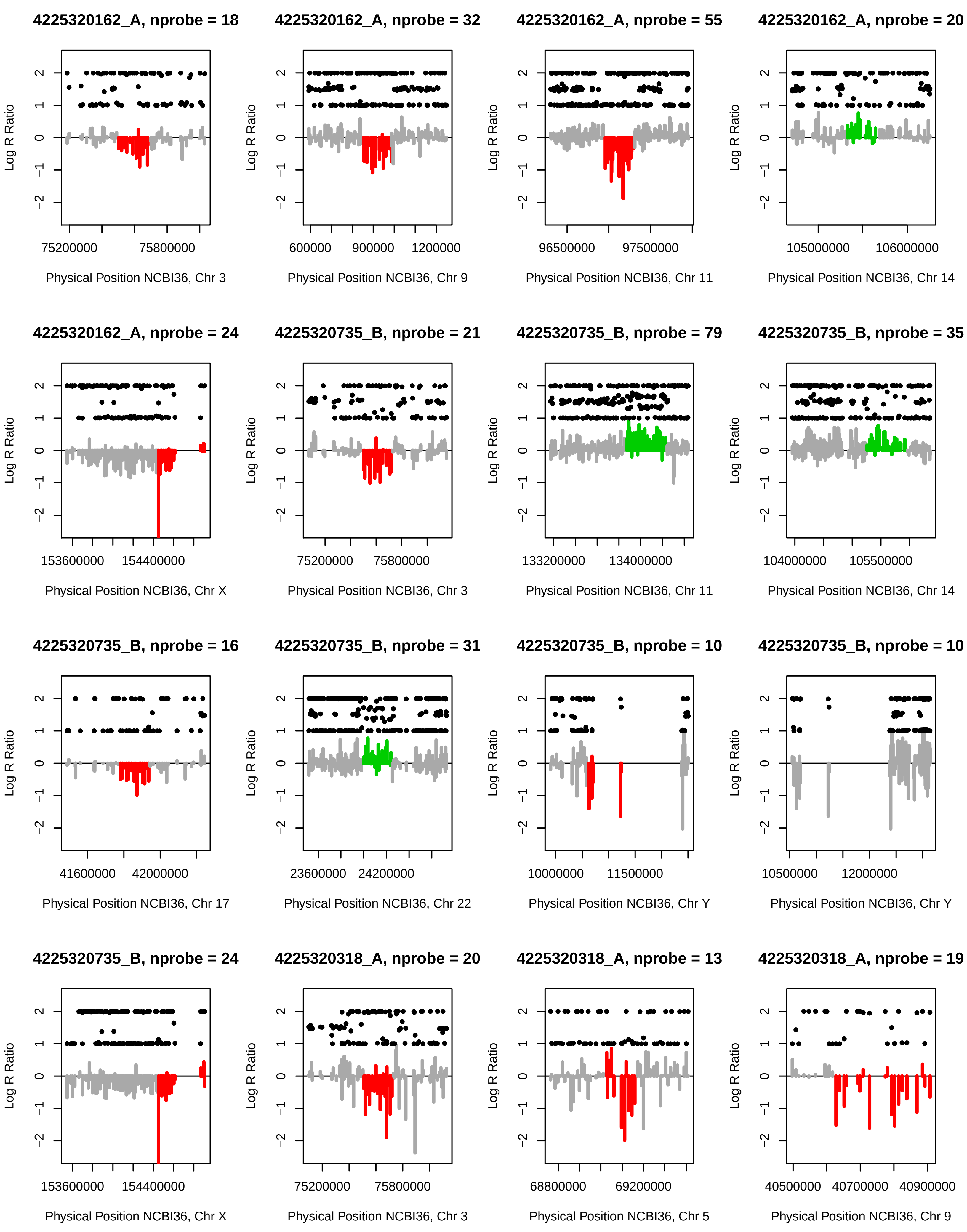
4225320162_A, nprobe = 18
Log R Ratio
Physical Position NCBI36, Chr 3
4225320162_A, nprobe = 32
Physical Position NCBI36, Chr 9
4225320162_A, nprobe = 55
Physical Position NCBI36, Chr 11
4225320162_A, nprobe = 20
Physical Position NCBI36, Chr 14
4225320162_A, nprobe = 24
Physical Position NCBI36, Chr X
4225320735_B, nprobe = 21
Physical Position NCBI36, Chr 3
4225320735_B, nprobe = 79
Physical Position NCBI36, Chr 11
4225320735_B, nprobe = 35
Physical Position NCBI36, Chr 14
4225320735_B, nprobe = 16
Physical Position NCBI36, Chr 17
4225320735_B, nprobe = 31
Physical Position NCBI36, Chr 22
4225320735_B, nprobe = 10
Physical Position NCBI36, Chr Y
4225320735_B, nprobe = 10
Physical Position NCBI36, Chr Y
4225320735_B, nprobe = 24
Physical Position NCBI36, Chr X
4225320318_A, nprobe = 20
Physical Position NCBI36, Chr 3
4225320318_A, nprobe = 13
Physical Position NCBI36, Chr 5
4225320318_A, nprobe = 19
Physical Position NCBI36, Chr 9

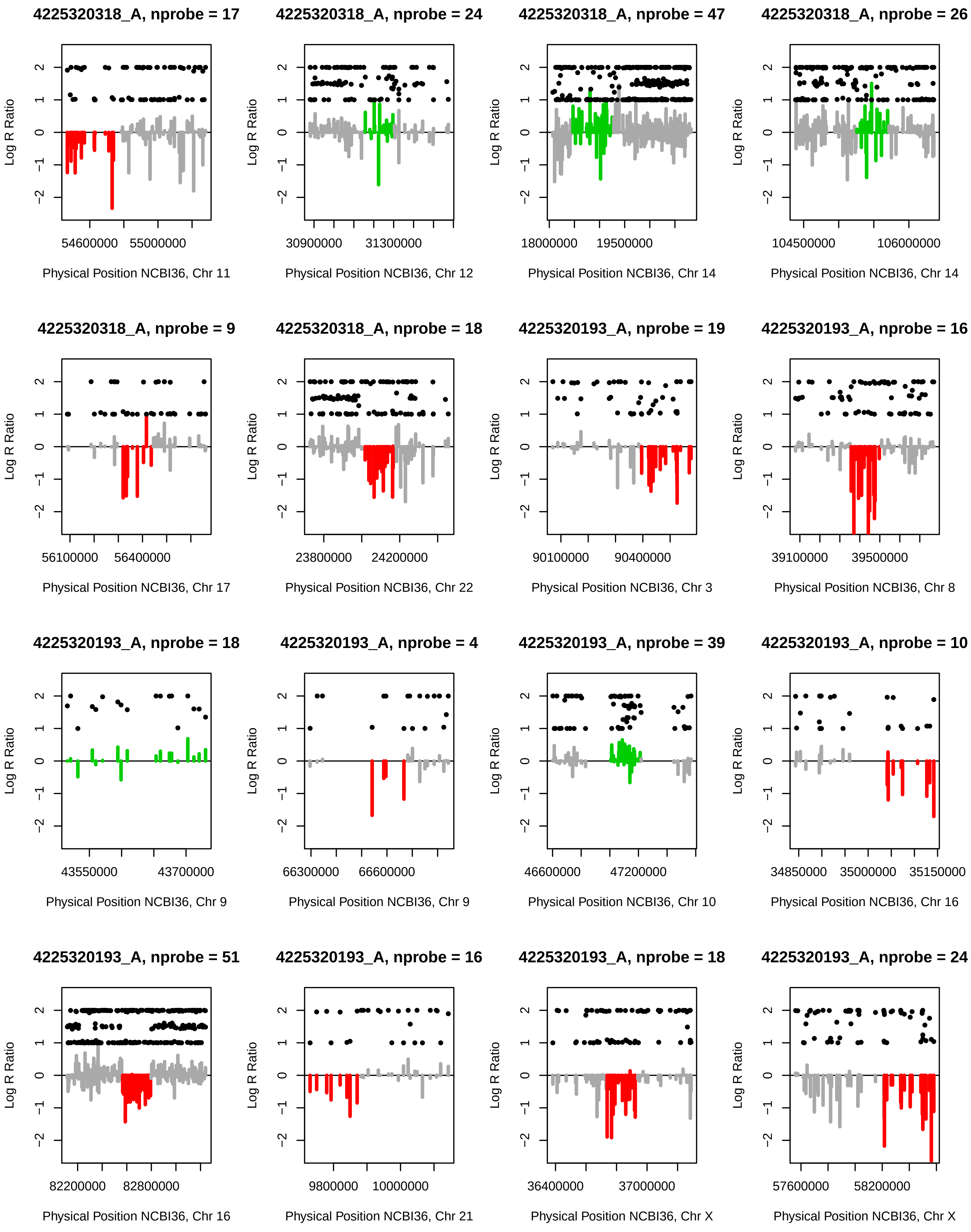
4225320318_A, nprobe = 17
Log R Ratio
54600000
55000000
Physical Position NCBI36, Chr 11
4225320318_A, nprobe = 24
Log R Ratio
30900000
31300000
Physical Position NCBI36, Chr 12
4225320318_A, nprobe = 47
Log R Ratio
18000000
19500000
Physical Position NCBI36, Chr 14
4225320318_A, nprobe = 26
Log R Ratio
104500000
106000000
Physical Position NCBI36, Chr 14
4225320318_A, nprobe = 9
Log R Ratio
56100000
56400000
Physical Position NCBI36, Chr 17
4225320318_A, nprobe = 18
Log R Ratio
23800000
24200000
Physical Position NCBI36, Chr 22
4225320193_A, nprobe = 19
Log R Ratio
90100000
90400000
Physical Position NCBI36, Chr 3
4225320193_A, nprobe = 16
Log R Ratio
39100000
39500000
Physical Position NCBI36, Chr 8
4225320193_A, nprobe = 18
Log R Ratio
43550000
43700000
Physical Position NCBI36, Chr 9
4225320193_A, nprobe = 4
Log R Ratio
66300000
66600000
Physical Position NCBI36, Chr 9
4225320193_A, nprobe = 39
Log R Ratio
46600000
47200000
Physical Position NCBI36, Chr 10
4225320193_A, nprobe = 10
Log R Ratio
34850000
35000000
35150000
Physical Position NCBI36, Chr 16
4225320193_A, nprobe = 51
Log R Ratio
82200000
82800000
Physical Position NCBI36, Chr 16
4225320193_A, nprobe = 16
Log R Ratio
9800000
10000000
Physical Position NCBI36, Chr 21
4225320193_A, nprobe = 18
Log R Ratio
36400000
37000000
Physical Position NCBI36, Chr X
4225320193_A, nprobe = 24
Log R Ratio
57600000
58200000
Physical Position NCBI36, Chr X

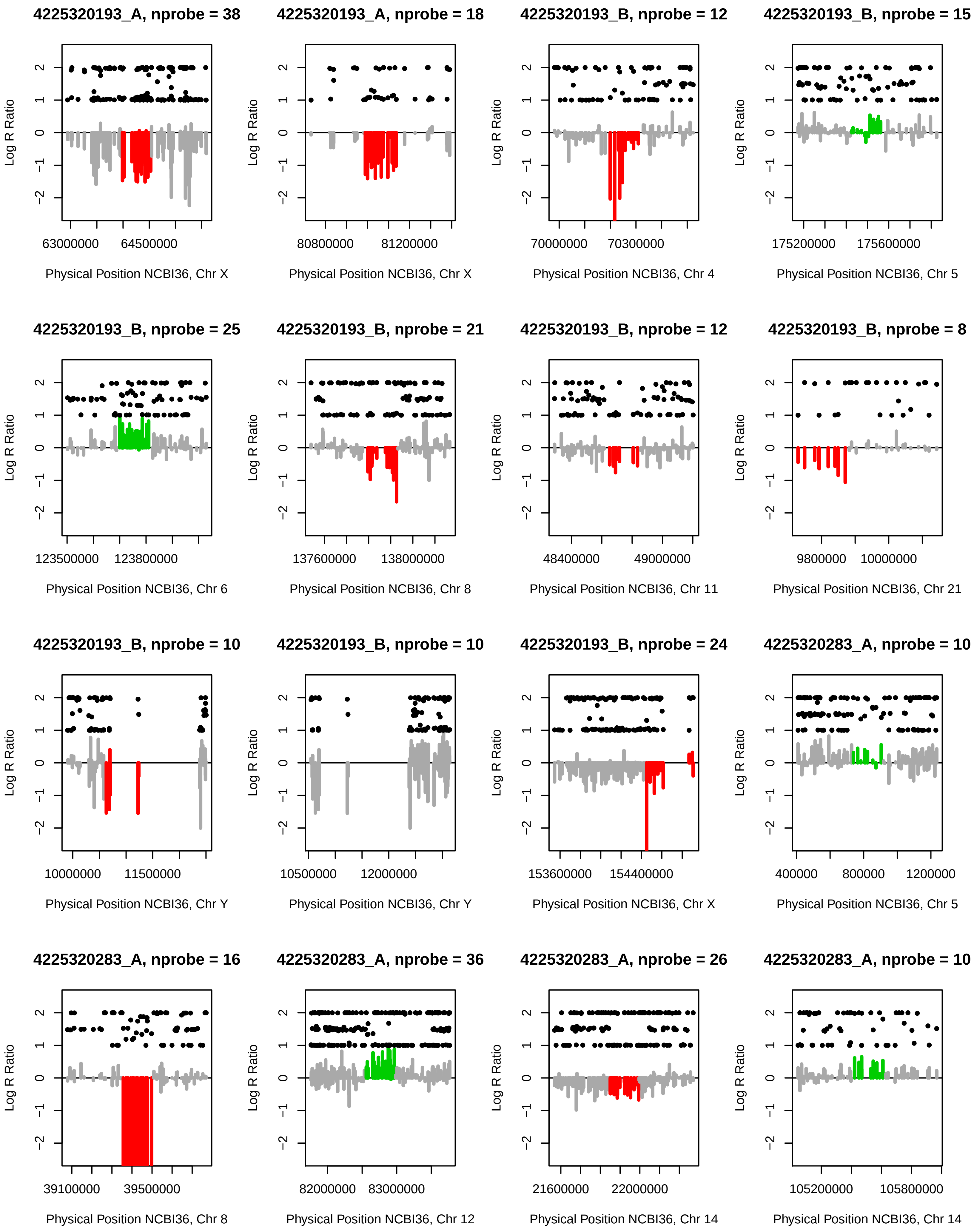

4225320193_A, nprobe = 38
Log R Ratio
Physical Position NCBI36, Chr X
4225320193_A, nprobe = 18
Log R Ratio
Physical Position NCBI36, Chr X
4225320193_B, nprobe = 12
Log R Ratio
Physical Position NCBI36, Chr 4
4225320193_B, nprobe = 15
Log R Ratio
Physical Position NCBI36, Chr 5
4225320193_B, nprobe = 25
Log R Ratio
Physical Position NCBI36, Chr 6
4225320193_B, nprobe = 21
Log R Ratio
Physical Position NCBI36, Chr 8
4225320193_B, nprobe = 12
Log R Ratio
Physical Position NCBI36, Chr 11
4225320193_B, nprobe = 8
Log R Ratio
Physical Position NCBI36, Chr 21
4225320193_B, nprobe = 10
Log R Ratio
Physical Position NCBI36, Chr Y
4225320193_B, nprobe = 10
Log R Ratio
Physical Position NCBI36, Chr Y
4225320193_B, nprobe = 24
Log R Ratio
Physical Position NCBI36, Chr X
4225320283_A, nprobe = 10
Log R Ratio
Physical Position NCBI36, Chr 5
4225320283_A, nprobe = 16
Log R Ratio
Physical Position NCBI36, Chr 8
4225320283_A, nprobe = 36
Log R Ratio
Physical Position NCBI36, Chr 12
4225320283_A, nprobe = 26
Log R Ratio
Physical Position NCBI36, Chr 14
4225320283_A, nprobe = 10
Log R Ratio
Physical Position NCBI36, Chr 14

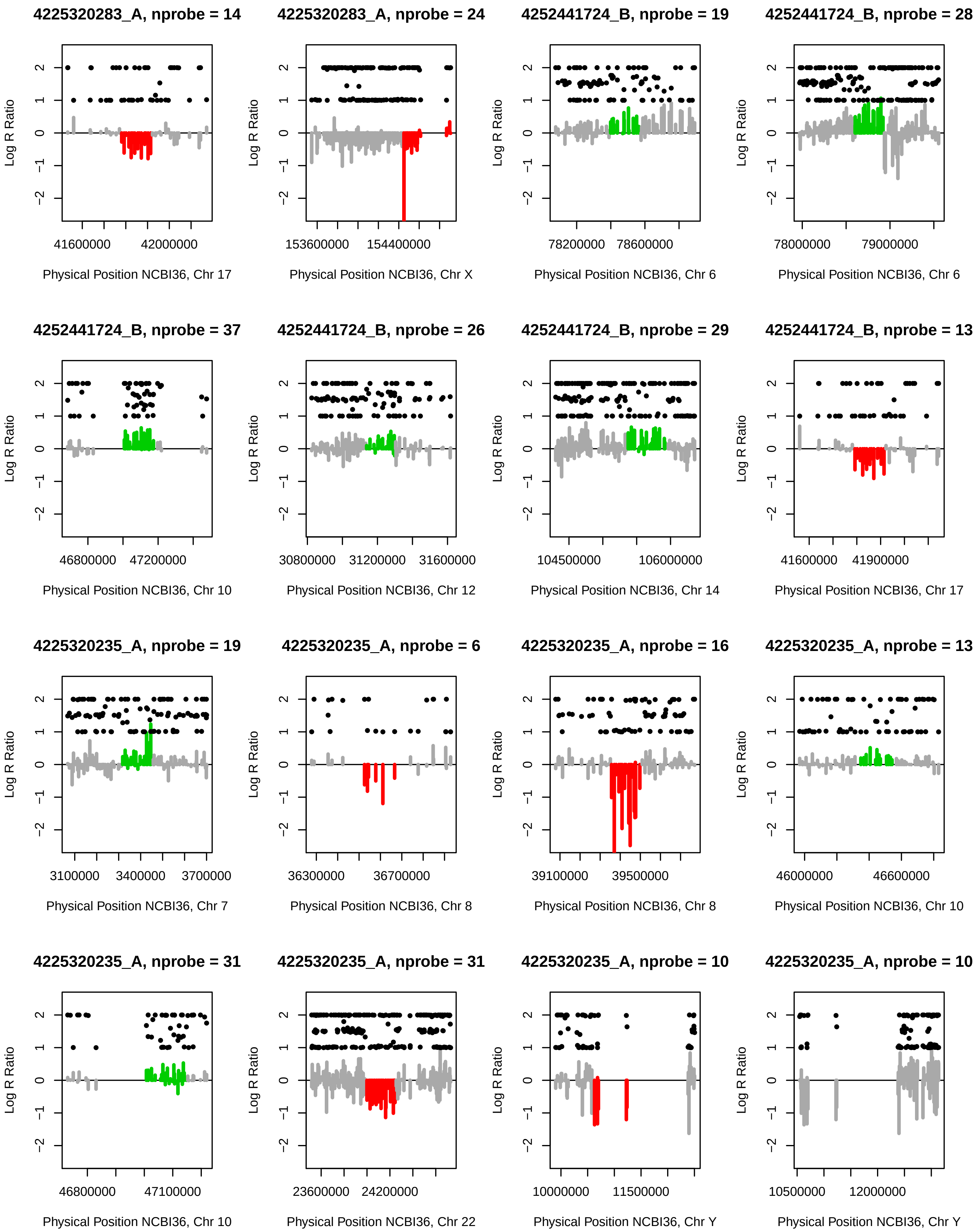

4225320283_A, nprobe = 14
Log R Ratio
41600000 42000000
Physical Position NCBI36, Chr 17
4225320283_A, nprobe = 24
153600000 154400000
Physical Position NCBI36, Chr X
4252441724_B, nprobe = 19
78200000 78600000
Physical Position NCBI36, Chr 6
4252441724_B, nprobe = 28
78000000 79000000
Physical Position NCBI36, Chr 6
4252441724_B, nprobe = 37
46800000 47200000
Physical Position NCBI36, Chr 10
4252441724_B, nprobe = 26
30800000 31200000 31600000
Physical Position NCBI36, Chr 12
4252441724_B, nprobe = 29
104500000 106000000
Physical Position NCBI36, Chr 14
4252441724_B, nprobe = 13
41600000 41900000
Physical Position NCBI36, Chr 17
4225320235_A, nprobe = 19
3100000 3400000 3700000
Physical Position NCBI36, Chr 7
4225320235_A, nprobe = 6
36300000 36700000
Physical Position NCBI36, Chr 8
4225320235_A, nprobe = 16
39100000 39500000
Physical Position NCBI36, Chr 8
4225320235_A, nprobe = 13
46000000 46600000
Physical Position NCBI36, Chr 10
4225320235_A, nprobe = 31
46800000 47100000
Physical Position NCBI36, Chr 10
4225320235_A, nprobe = 31
23600000 24200000
Physical Position NCBI36, Chr 22
4225320235_A, nprobe = 10
10000000 11500000
Physical Position NCBI36, Chr Y
4225320235_A, nprobe = 10
10500000 12000000
Physical Position NCBI36, Chr Y

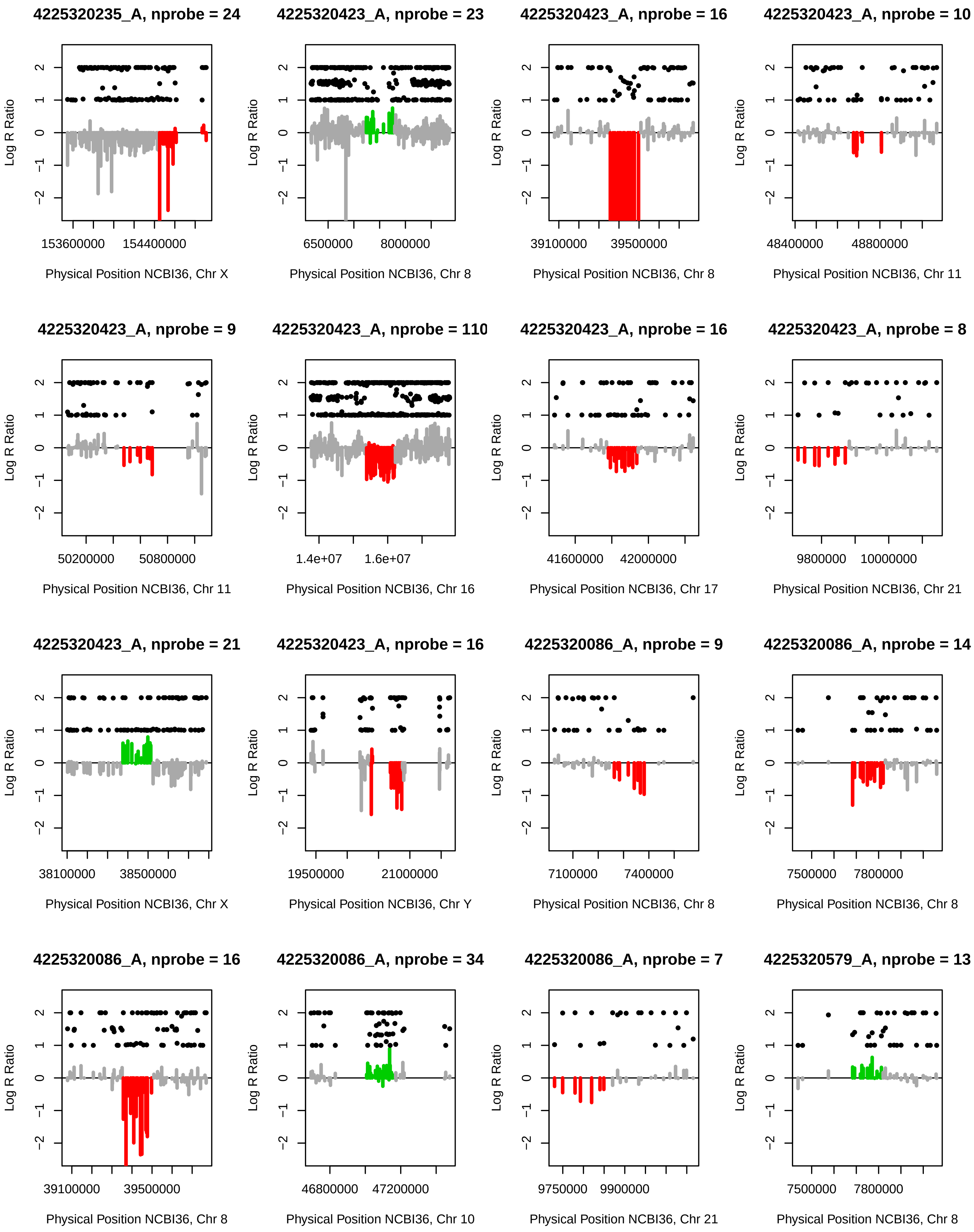
4225320235_A, nprobe = 24
Log R Ratio
153600000
154400000
Physical Position NCBI36, Chr X
4225320423_A, nprobe = 23
6500000
8000000
Physical Position NCBI36, Chr 8
4225320423_A, nprobe = 16
39100000
39500000
Physical Position NCBI36, Chr 8
4225320423_A, nprobe = 10
48400000
48800000
Physical Position NCBI36, Chr 11
4225320423_A, nprobe = 9
50200000
50800000
Physical Position NCBI36, Chr 11
4225320423_A, nprobe = 110
1.4e+07
1.6e+07
Physical Position NCBI36, Chr 16
4225320423_A, nprobe = 16
41600000
42000000
Physical Position NCBI36, Chr 17
4225320423_A, nprobe = 8
9800000
10000000
Physical Position NCBI36, Chr 21
4225320423_A, nprobe = 21
38100000
38500000
Physical Position NCBI36, Chr X
4225320423_A, nprobe = 16
19500000
21000000
Physical Position NCBI36, Chr Y
4225320086_A, nprobe = 9
7100000
7400000
Physical Position NCBI36, Chr 8
4225320086_A, nprobe = 14
7500000
7800000
Physical Position NCBI36, Chr 8
4225320086_A, nprobe = 16
39100000
39500000
Physical Position NCBI36, Chr 8
4225320086_A, nprobe = 34
46800000
47200000
Physical Position NCBI36, Chr 10
4225320086_A, nprobe = 7
9750000
9900000
Physical Position NCBI36, Chr 21
4225320579_A, nprobe = 13
7500000
7800000
Physical Position NCBI36, Chr 8

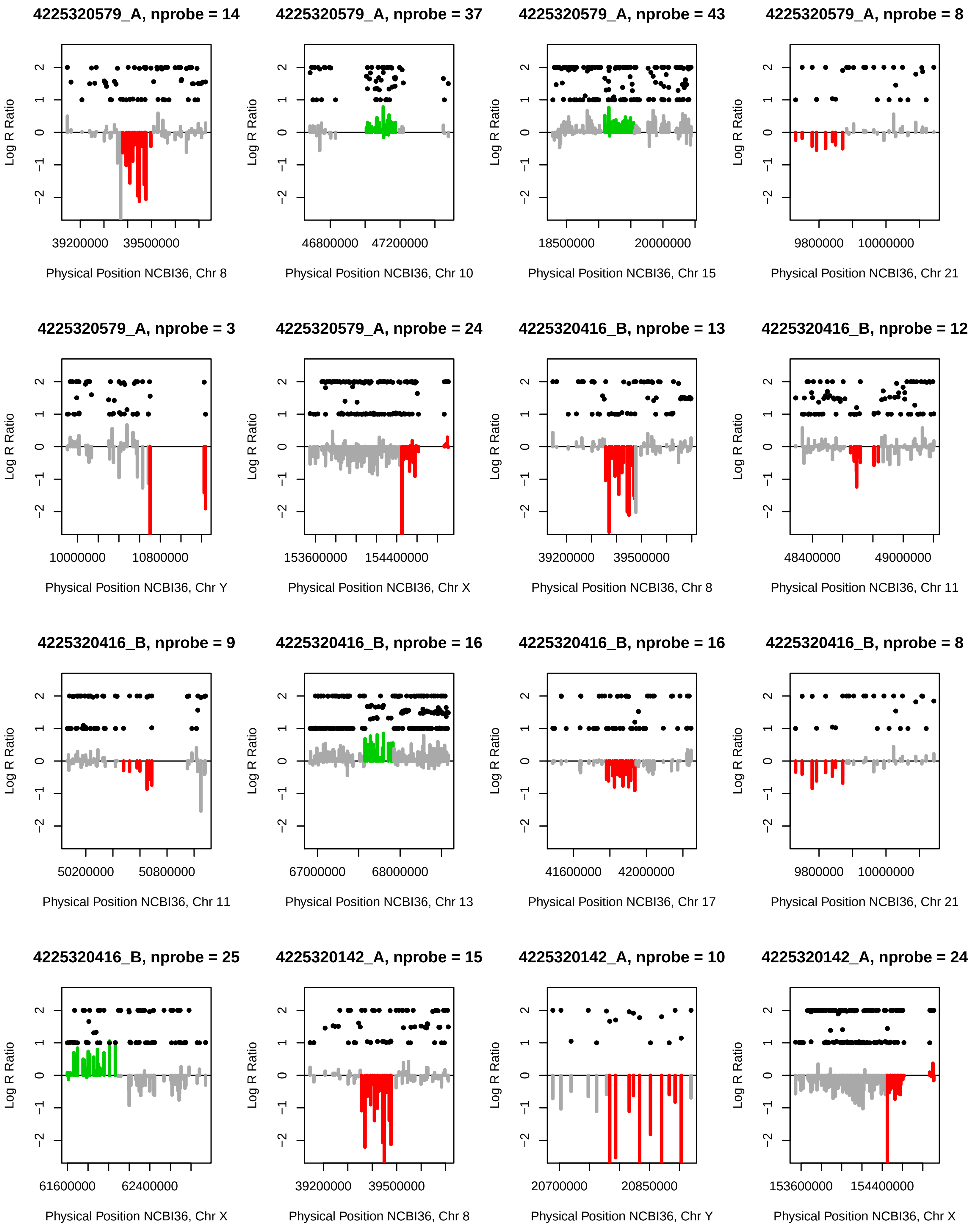
4225320579_A, nprobe = 14
Log R Ratio
Physical Position NCBI36, Chr 8
39200000
39500000
4225320579_A, nprobe = 37
Physical Position NCBI36, Chr 10
46800000
47200000
4225320579_A, nprobe = 43
Physical Position NCBI36, Chr 15
18500000
20000000
4225320579_A, nprobe = 8
Physical Position NCBI36, Chr 21
9800000
10000000
4225320579_A, nprobe = 3
Physical Position NCBI36, Chr Y
10000000
10800000
4225320579_A, nprobe = 24
Physical Position NCBI36, Chr X
153600000
154400000
4225320416_B, nprobe = 13
Physical Position NCBI36, Chr 8
39200000
39500000
4225320416_B, nprobe = 12
Physical Position NCBI36, Chr 11
48400000
49000000
4225320416_B, nprobe = 9
Physical Position NCBI36, Chr 11
50200000
50800000
4225320416_B, nprobe = 16
Physical Position NCBI36, Chr 13
67000000
68000000
4225320416_B, nprobe = 16
Physical Position NCBI36, Chr 17
41600000
42000000
4225320416_B, nprobe = 8
Physical Position NCBI36, Chr 21
9800000
10000000
4225320416_B, nprobe = 25
Physical Position NCBI36, Chr X
61600000
62400000
4225320142_A, nprobe = 15
Physical Position NCBI36, Chr 8
39200000
39500000
4225320142_A, nprobe = 10
Physical Position NCBI36, Chr Y
20700000
20850000
4225320142_A, nprobe = 24
Physical Position NCBI36, Chr X
153600000
154400000

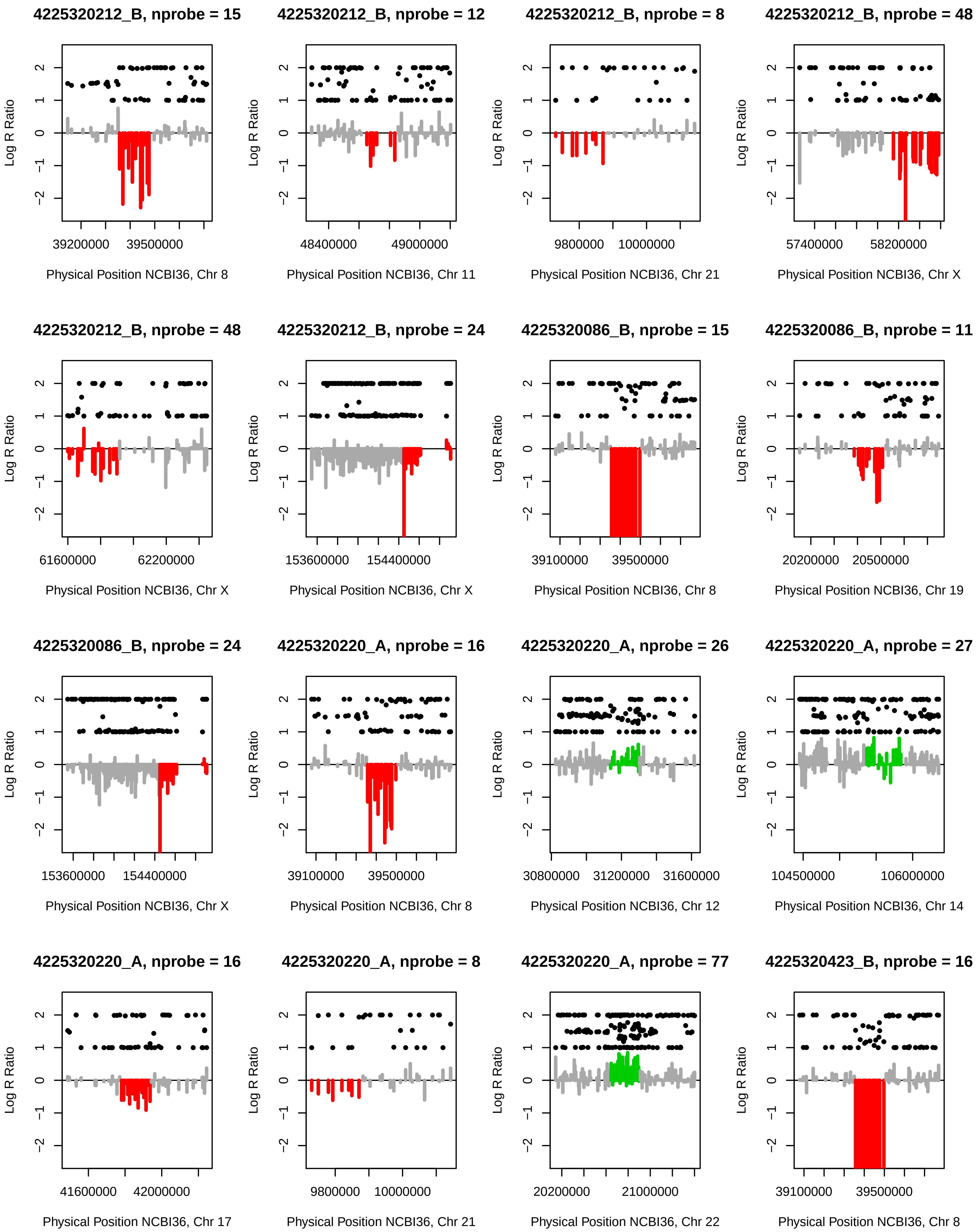
4225320212_B, nprobe = 15
Log R Ratio
39200000 39500000
Physical Position NCBI36, Chr 8
4225320212_B, nprobe = 12
Log R Ratio
48400000 49000000
Physical Position NCBI36, Chr 11
4225320212_B, nprobe = 8
Log R Ratio
9800000 10000000
Physical Position NCBI36, Chr 21
4225320212_B, nprobe = 48
Log R Ratio
57400000 58200000
Physical Position NCBI36, Chr X
4225320212_B, nprobe = 48
Log R Ratio
61600000 62200000
Physical Position NCBI36, Chr X
4225320212_B, nprobe = 24
Log R Ratio
153600000 154400000
Physical Position NCBI36, Chr X
4225320086_B, nprobe = 15
Log R Ratio
39100000 39500000
Physical Position NCBI36, Chr 8
4225320086_B, nprobe = 11
Log R Ratio
20200000 20500000
Physical Position NCBI36, Chr 19
4225320086_B, nprobe = 24
Log R Ratio
153600000 154400000
Physical Position NCBI36, Chr X
4225320220_A, nprobe = 16
Log R Ratio
39100000 39500000
Physical Position NCBI36, Chr 8
4225320220_A, nprobe = 26
Log R Ratio
30800000 31200000 31600000
Physical Position NCBI36, Chr 12
4225320220_A, nprobe = 27
Log R Ratio
104500000 106000000
Physical Position NCBI36, Chr 14
4225320220_A, nprobe = 16
Log R Ratio
41600000 42000000
Physical Position NCBI36, Chr 17
4225320220_A, nprobe = 8
Log R Ratio
9800000 10000000
Physical Position NCBI36, Chr 21
4225320220_A, nprobe = 77
Log R Ratio
20200000 21000000
Physical Position NCBI36, Chr 22
4225320423_B, nprobe = 16
Log R Ratio
39100000 39500000
Physical Position NCBI36, Chr 8

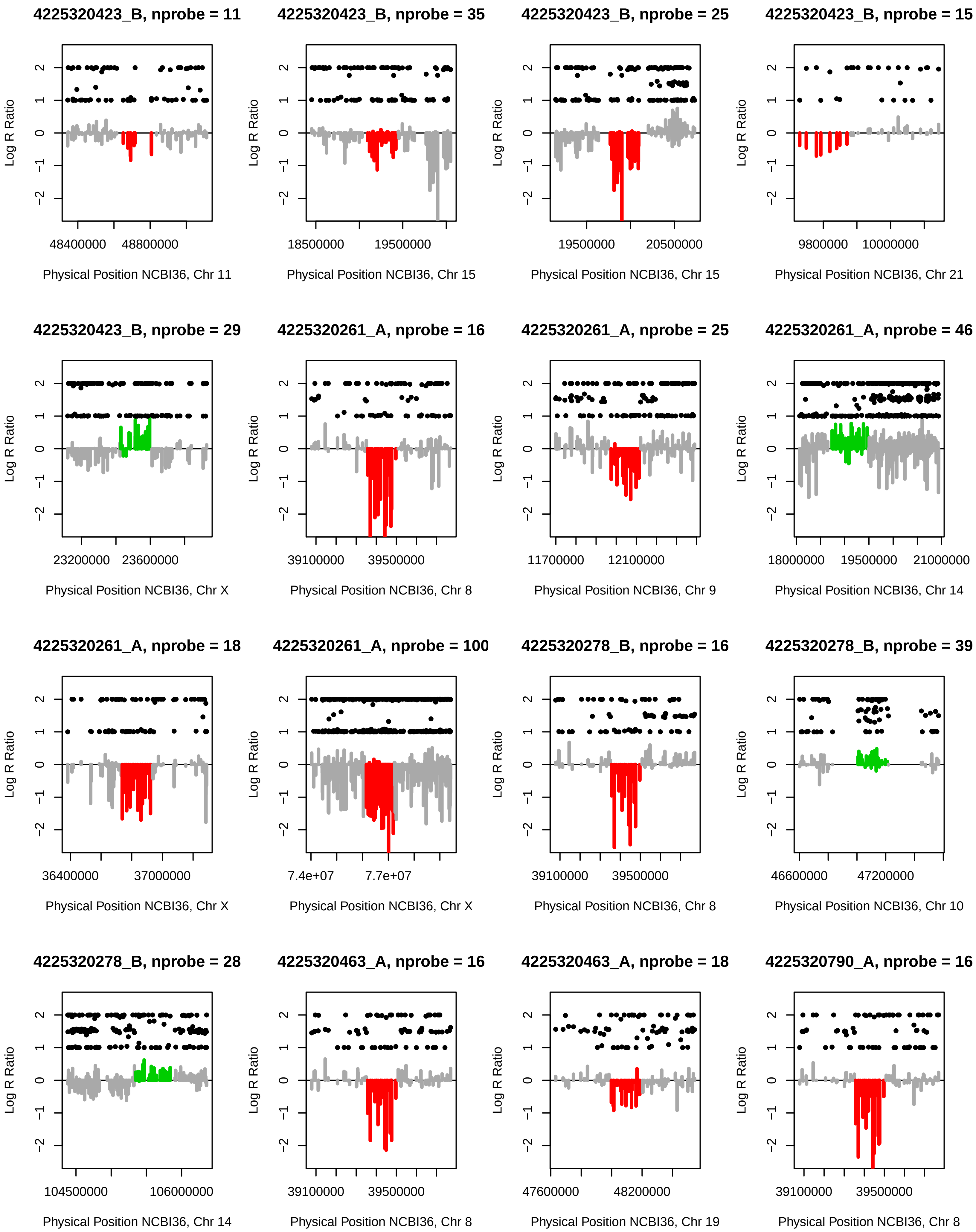
4225320423_B, nprobe = 11
Log R Ratio
48400000
48800000
Physical Position NCBI36, Chr 11
4225320423_B, nprobe = 35
Log R Ratio
18500000
19500000
Physical Position NCBI36, Chr 15
4225320423_B, nprobe = 25
Log R Ratio
19500000
20500000
Physical Position NCBI36, Chr 15
4225320423_B, nprobe = 15
Log R Ratio
9800000
10000000
Physical Position NCBI36, Chr 21
4225320423_B, nprobe = 29
Log R Ratio
23200000
23600000
Physical Position NCBI36, Chr X
4225320261_A, nprobe = 16
Log R Ratio
39100000
39500000
Physical Position NCBI36, Chr 8
4225320261_A, nprobe = 25
Log R Ratio
11700000
12100000
Physical Position NCBI36, Chr 9
4225320261_A, nprobe = 46
Log R Ratio
18000000
19500000
21000000
Physical Position NCBI36, Chr 14
4225320261_A, nprobe = 18
Log R Ratio
36400000
37000000
Physical Position NCBI36, Chr X
4225320261_A, nprobe = 100
Log R Ratio
7.4e+07
7.7e+07
Physical Position NCBI36, Chr X
4225320278_B, nprobe = 16
Log R Ratio
39100000
39500000
Physical Position NCBI36, Chr 8
4225320278_B, nprobe = 39
Log R Ratio
46600000
47200000
Physical Position NCBI36, Chr 10
4225320278_B, nprobe = 28
Log R Ratio
104500000
106000000
Physical Position NCBI36, Chr 14
4225320463_A, nprobe = 16
Log R Ratio
39100000
39500000
Physical Position NCBI36, Chr 8
4225320463_A, nprobe = 18
Log R Ratio
47600000
48200000
Physical Position NCBI36, Chr 19
4225320790_A, nprobe = 16
Log R Ratio
39100000
39500000
Physical Position NCBI36, Chr 8

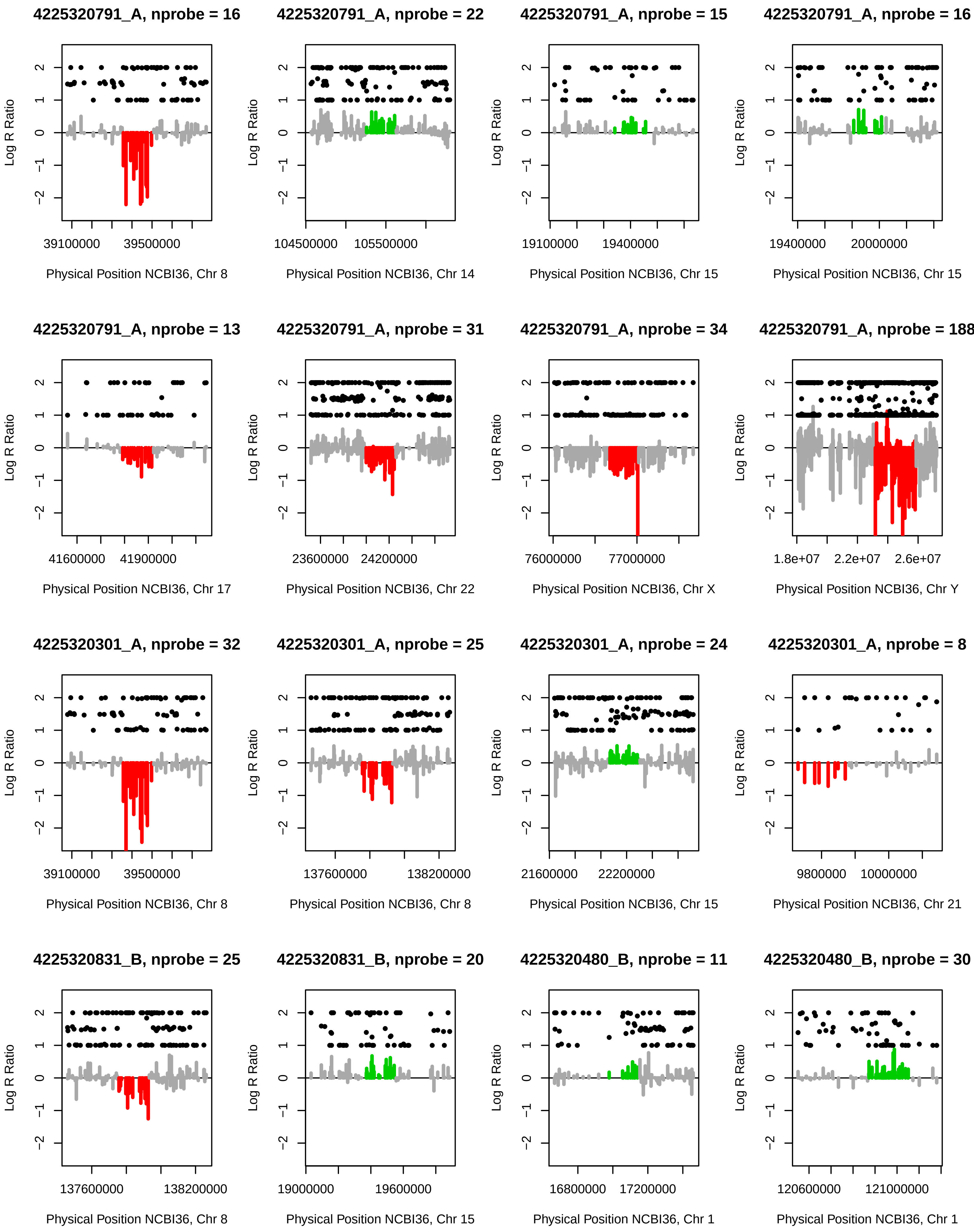
4225320791_A, nprobe = 16
Log R Ratio
39100000
39500000
Physical Position NCBI36, Chr 8
4225320791_A, nprobe = 22
Log R Ratio
104500000
105500000
Physical Position NCBI36, Chr 14
4225320791_A, nprobe = 15
Log R Ratio
19100000
19400000
Physical Position NCBI36, Chr 15
4225320791_A, nprobe = 16
Log R Ratio
19400000
20000000
Physical Position NCBI36, Chr 15
4225320791_A, nprobe = 13
Log R Ratio
41600000
41900000
Physical Position NCBI36, Chr 17
4225320791_A, nprobe = 31
Log R Ratio
23600000
24200000
Physical Position NCBI36, Chr 22
4225320791_A, nprobe = 34
Log R Ratio
76000000
77000000
Physical Position NCBI36, Chr X
4225320791_A, nprobe = 188
Log R Ratio
1.8e+07
2.2e+07
2.6e+07
Physical Position NCBI36, Chr Y
4225320301_A, nprobe = 32
Log R Ratio
39100000
39500000
Physical Position NCBI36, Chr 8
4225320301_A, nprobe = 25
Log R Ratio
137600000
138200000
Physical Position NCBI36, Chr 8
4225320301_A, nprobe = 24
Log R Ratio
21600000
22200000
Physical Position NCBI36, Chr 15
4225320301_A, nprobe = 8
Log R Ratio
9800000
10000000
Physical Position NCBI36, Chr 21
4225320831_B, nprobe = 25
Log R Ratio
137600000
138200000
Physical Position NCBI36, Chr 8
4225320831_B, nprobe = 20
Log R Ratio
19000000
19600000
Physical Position NCBI36, Chr 15
4225320480_B, nprobe = 11
Log R Ratio
16800000
17200000
Physical Position NCBI36, Chr 1
4225320480_B, nprobe = 30
Log R Ratio
120600000
121000000
Physical Position NCBI36, Chr 1

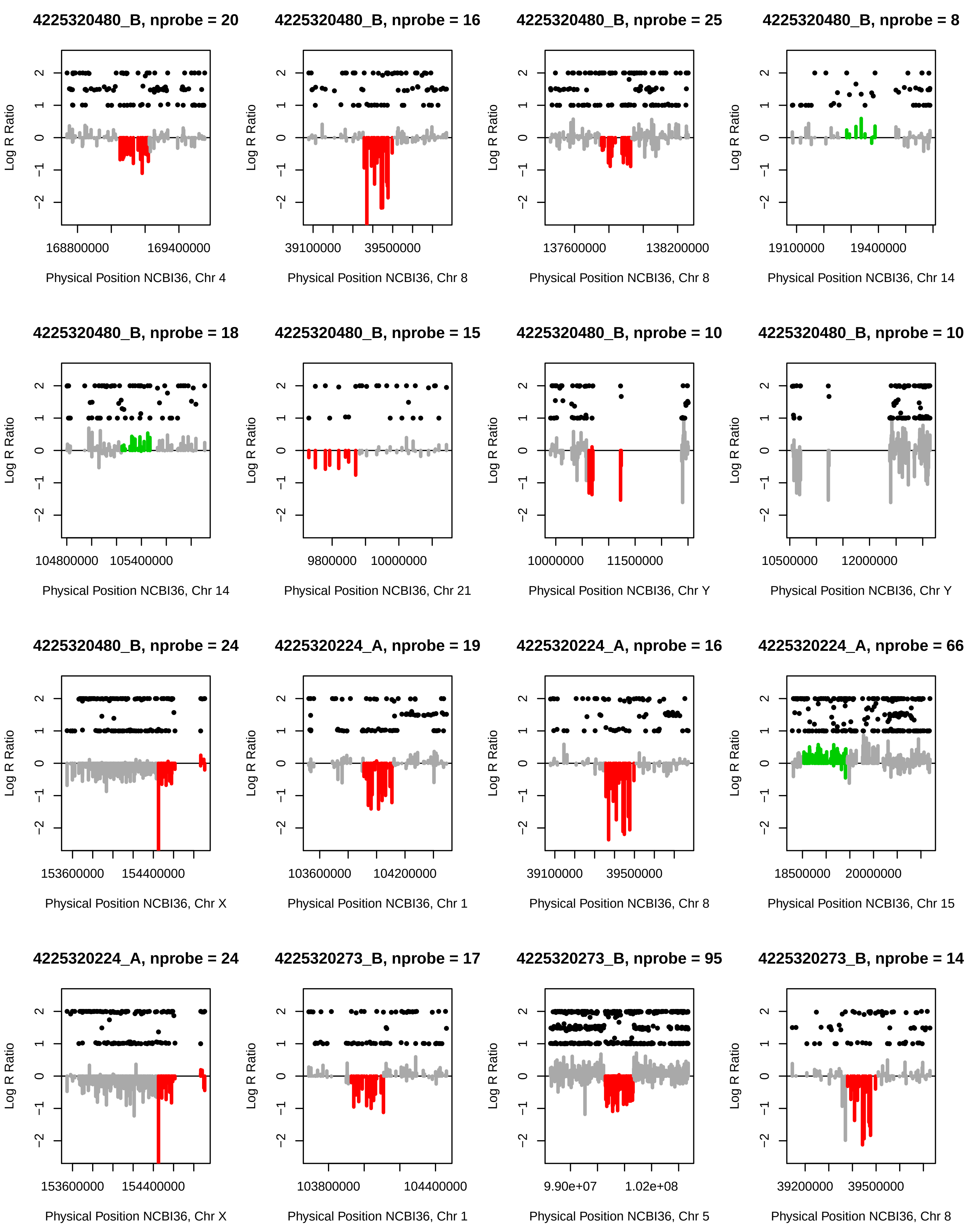

4225320480_B, nprobe = 20
Log R Ratio
168800000 169400000
Physical Position NCBI36, Chr 4
4225320480_B, nprobe = 16
Log R Ratio
39100000 39500000
Physical Position NCBI36, Chr 8
4225320480_B, nprobe = 25
Log R Ratio
137600000 138200000
Physical Position NCBI36, Chr 8
4225320480_B, nprobe = 8
Log R Ratio
19100000 19400000
Physical Position NCBI36, Chr 14
4225320480_B, nprobe = 18
Log R Ratio
104800000 105400000
Physical Position NCBI36, Chr 14
4225320480_B, nprobe = 15
Log R Ratio
9800000 10000000
Physical Position NCBI36, Chr 21
4225320480_B, nprobe = 10
Log R Ratio
10000000 11500000
Physical Position NCBI36, Chr Y
4225320480_B, nprobe = 10
Log R Ratio
10500000 12000000
Physical Position NCBI36, Chr Y
4225320480_B, nprobe = 24
Log R Ratio
153600000 154400000
Physical Position NCBI36, Chr X
4225320224_A, nprobe = 19
Log R Ratio
103600000 104200000
Physical Position NCBI36, Chr 1
4225320224_A, nprobe = 16
Log R Ratio
39100000 39500000
Physical Position NCBI36, Chr 8
4225320224_A, nprobe = 66
Log R Ratio
18500000 20000000
Physical Position NCBI36, Chr 15
4225320224_A, nprobe = 24
Log R Ratio
153600000 154400000
Physical Position NCBI36, Chr X
4225320273_B, nprobe = 17
Log R Ratio
103800000 104400000
Physical Position NCBI36, Chr 1
4225320273_B, nprobe = 95
Log R Ratio
9.90e+07 1.02e+08
Physical Position NCBI36, Chr 5
4225320273_B, nprobe = 14
Log R Ratio
39200000 39500000
Physical Position NCBI36, Chr 8

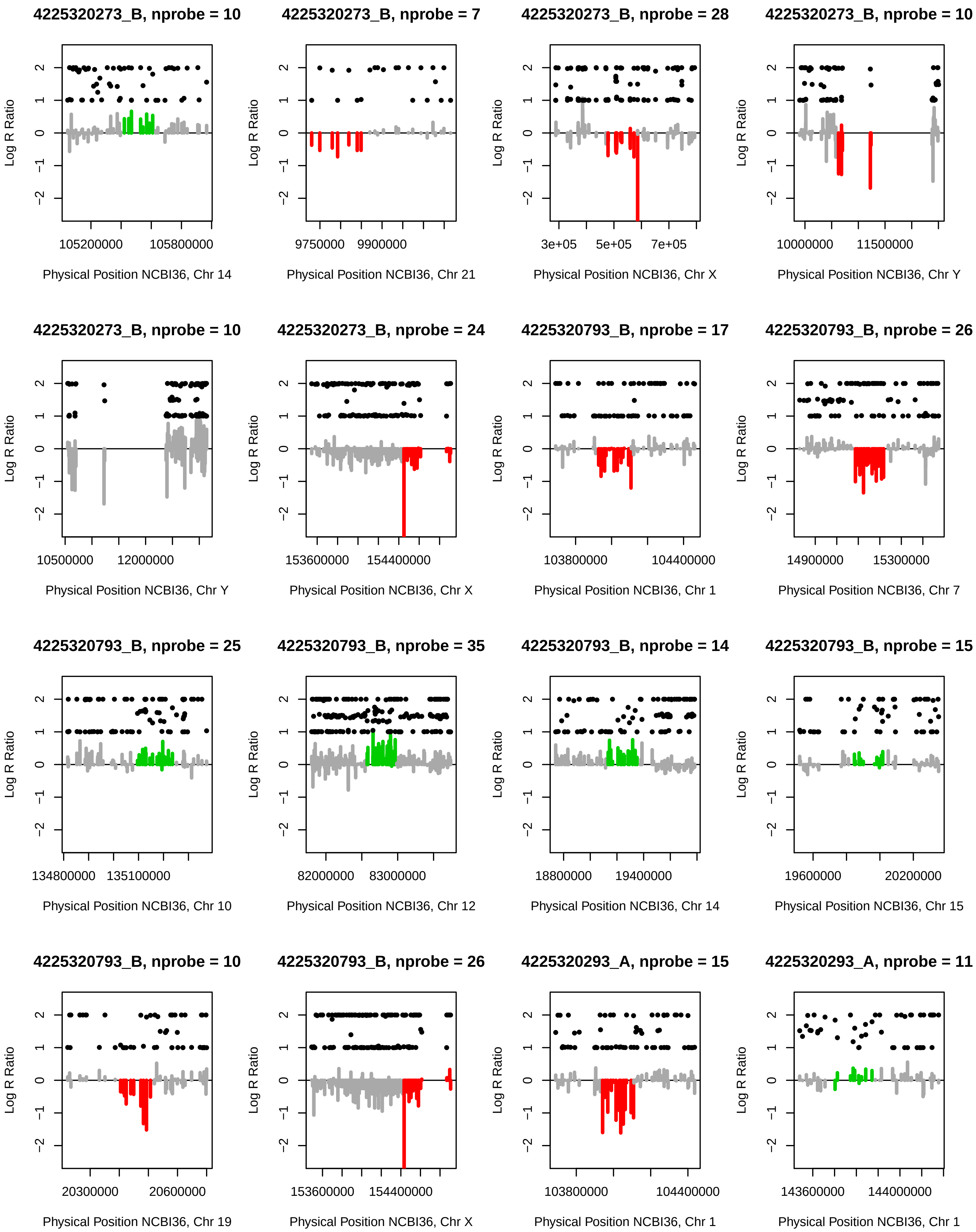

4225320273_B, nprobe = 10
Log R Ratio
105200000
105800000
Physical Position NCBI36, Chr 14
4225320273_B, nprobe = 7
Log R Ratio
9750000
9900000
Physical Position NCBI36, Chr 21
4225320273_B, nprobe = 28
Log R Ratio
3e+05
5e+05
7e+05
Physical Position NCBI36, Chr X
4225320273_B, nprobe = 10
Log R Ratio
10000000
11500000
Physical Position NCBI36, Chr Y
4225320273_B, nprobe = 10
Log R Ratio
10500000
12000000
Physical Position NCBI36, Chr Y
4225320273_B, nprobe = 24
Log R Ratio
153600000
154400000
Physical Position NCBI36, Chr X
4225320793_B, nprobe = 17
Log R Ratio
103800000
104400000
Physical Position NCBI36, Chr 1
4225320793_B, nprobe = 26
Log R Ratio
14900000
15300000
Physical Position NCBI36, Chr 7
4225320793_B, nprobe = 25
Log R Ratio
134800000
135100000
Physical Position NCBI36, Chr 10
4225320793_B, nprobe = 35
Log R Ratio
82000000
83000000
Physical Position NCBI36, Chr 12
4225320793_B, nprobe = 14
Log R Ratio
18800000
19400000
Physical Position NCBI36, Chr 14
4225320793_B, nprobe = 15
Log R Ratio
19600000
20200000
Physical Position NCBI36, Chr 15
4225320793_B, nprobe = 10
Log R Ratio
20300000
20600000
Physical Position NCBI36, Chr 19
4225320793_B, nprobe = 26
Log R Ratio
153600000
154400000
Physical Position NCBI36, Chr X
4225320293_A, nprobe = 15
Log R Ratio
103800000
104400000
Physical Position NCBI36, Chr 1
4225320293_A, nprobe = 11
Log R Ratio
143600000
144000000
Physical Position NCBI36, Chr 1

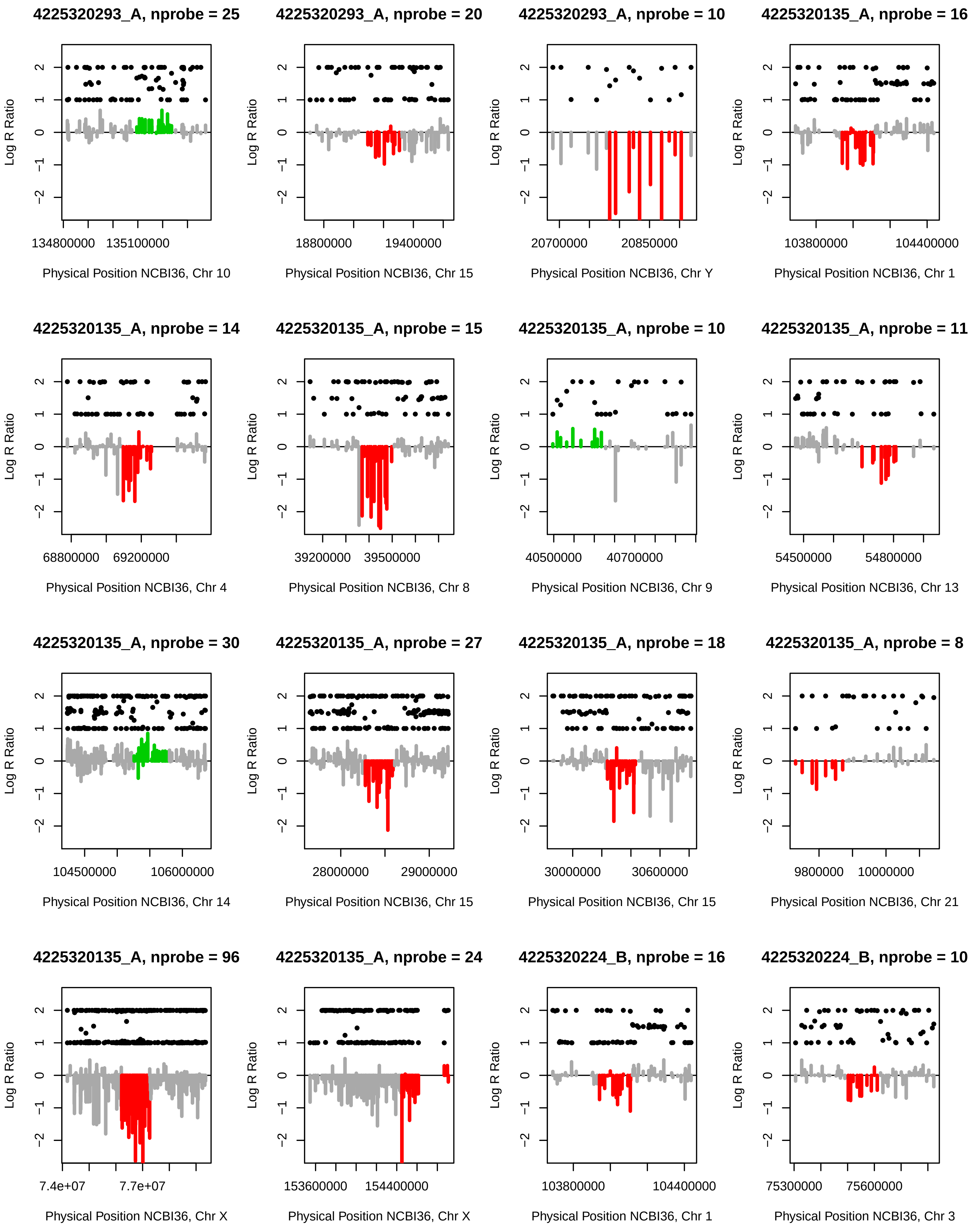

4225320293_A, nprobe = 25
Log R Ratio
134800000
135100000
Physical Position NCBI36, Chr 10
4225320293_A, nprobe = 20
Log R Ratio
18800000
19400000
Physical Position NCBI36, Chr 15
4225320293_A, nprobe = 10
Log R Ratio
20700000
20850000
Physical Position NCBI36, Chr Y
4225320135_A, nprobe = 16
Log R Ratio
103800000
104400000
Physical Position NCBI36, Chr 1
4225320135_A, nprobe = 14
Log R Ratio
68800000
69200000
Physical Position NCBI36, Chr 4
4225320135_A, nprobe = 15
Log R Ratio
39200000
39500000
Physical Position NCBI36, Chr 8
4225320135_A, nprobe = 10
Log R Ratio
40500000
40700000
Physical Position NCBI36, Chr 9
4225320135_A, nprobe = 11
Log R Ratio
54500000
54800000
Physical Position NCBI36, Chr 13
4225320135_A, nprobe = 30
Log R Ratio
104500000
106000000
Physical Position NCBI36, Chr 14
4225320135_A, nprobe = 27
Log R Ratio
28000000
29000000
Physical Position NCBI36, Chr 15
4225320135_A, nprobe = 18
Log R Ratio
30000000
30600000
Physical Position NCBI36, Chr 15
4225320135_A, nprobe = 8
Log R Ratio
9800000
10000000
Physical Position NCBI36, Chr 21
4225320135_A, nprobe = 96
Log R Ratio
7.4e+07
7.7e+07
Physical Position NCBI36, Chr X
4225320135_A, nprobe = 24
Log R Ratio
153600000
154400000
Physical Position NCBI36, Chr X
4225320224_B, nprobe = 16
Log R Ratio
103800000
104400000
Physical Position NCBI36, Chr 1
4225320224_B, nprobe = 10
Log R Ratio
75300000
75600000
Physical Position NCBI36, Chr 3

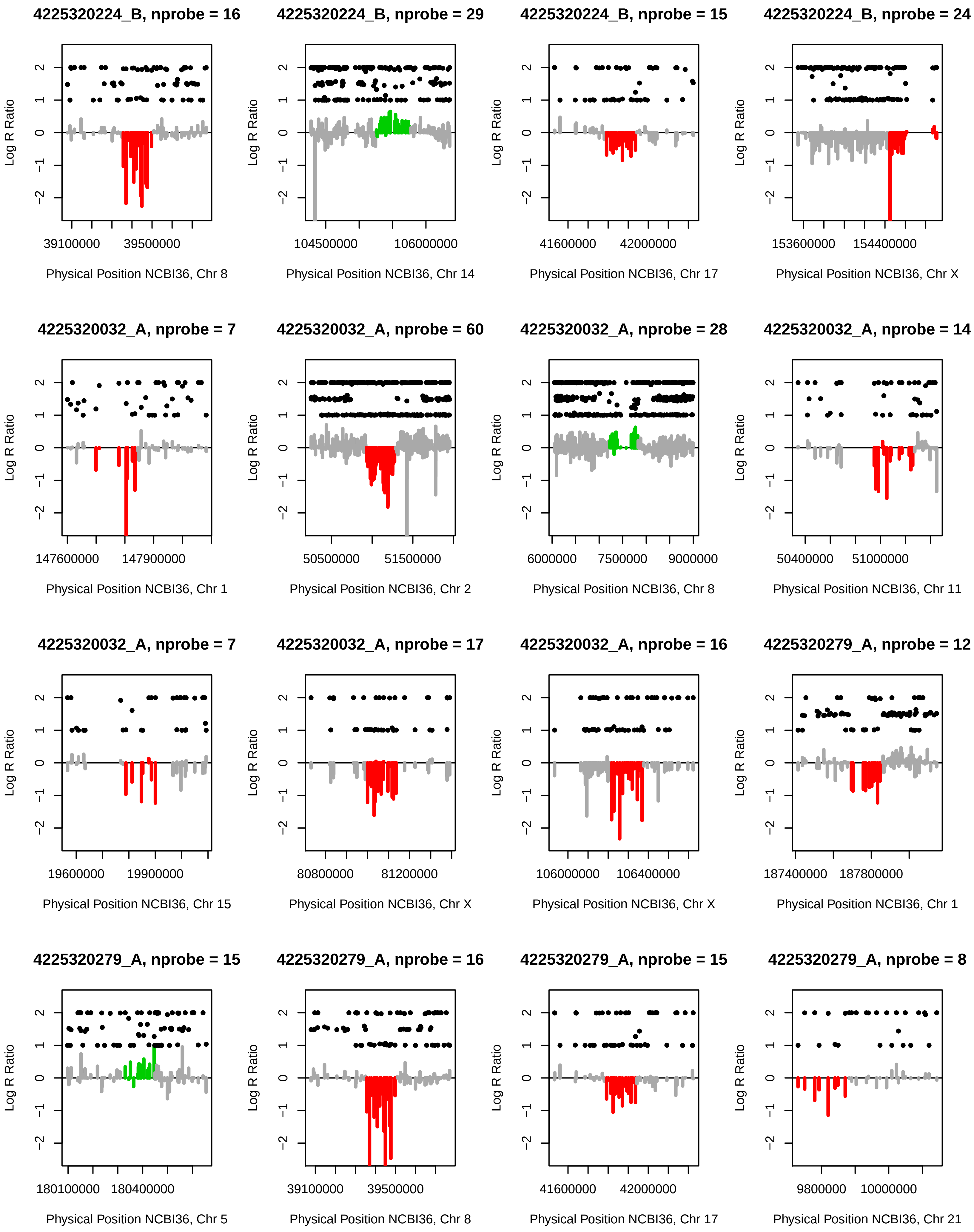
4225320224_B, nprobe = 16
Log R Ratio
Physical Position NCBI36, Chr 8
39100000
39500000
4225320224_B, nprobe = 29
Log R Ratio
Physical Position NCBI36, Chr 14
104500000
106000000
4225320224_B, nprobe = 15
Log R Ratio
Physical Position NCBI36, Chr 17
41600000
42000000
4225320224_B, nprobe = 24
Log R Ratio
Physical Position NCBI36, Chr X
153600000
154400000
4225320032_A, nprobe = 7
Log R Ratio
Physical Position NCBI36, Chr 1
147600000
147900000
4225320032_A, nprobe = 60
Log R Ratio
Physical Position NCBI36, Chr 2
50500000
51500000
4225320032_A, nprobe = 28
Log R Ratio
Physical Position NCBI36, Chr 8
6000000
7500000
9000000
4225320032_A, nprobe = 14
Log R Ratio
Physical Position NCBI36, Chr 11
50400000
51000000
4225320032_A, nprobe = 7
Log R Ratio
Physical Position NCBI36, Chr 15
19600000
19900000
4225320032_A, nprobe = 17
Log R Ratio
Physical Position NCBI36, Chr X
80800000
81200000
4225320032_A, nprobe = 16
Log R Ratio
Physical Position NCBI36, Chr X
106000000
106400000
4225320279_A, nprobe = 12
Log R Ratio
Physical Position NCBI36, Chr 1
187400000
187800000
4225320279_A, nprobe = 15
Log R Ratio
Physical Position NCBI36, Chr 5
180100000
180400000
4225320279_A, nprobe = 16
Log R Ratio
Physical Position NCBI36, Chr 8
39100000
39500000
4225320279_A, nprobe = 15
Log R Ratio
Physical Position NCBI36, Chr 17
41600000
42000000
4225320279_A, nprobe = 8
Log R Ratio
Physical Position NCBI36, Chr 21
9800000
10000000
2
1
0
−1
−2

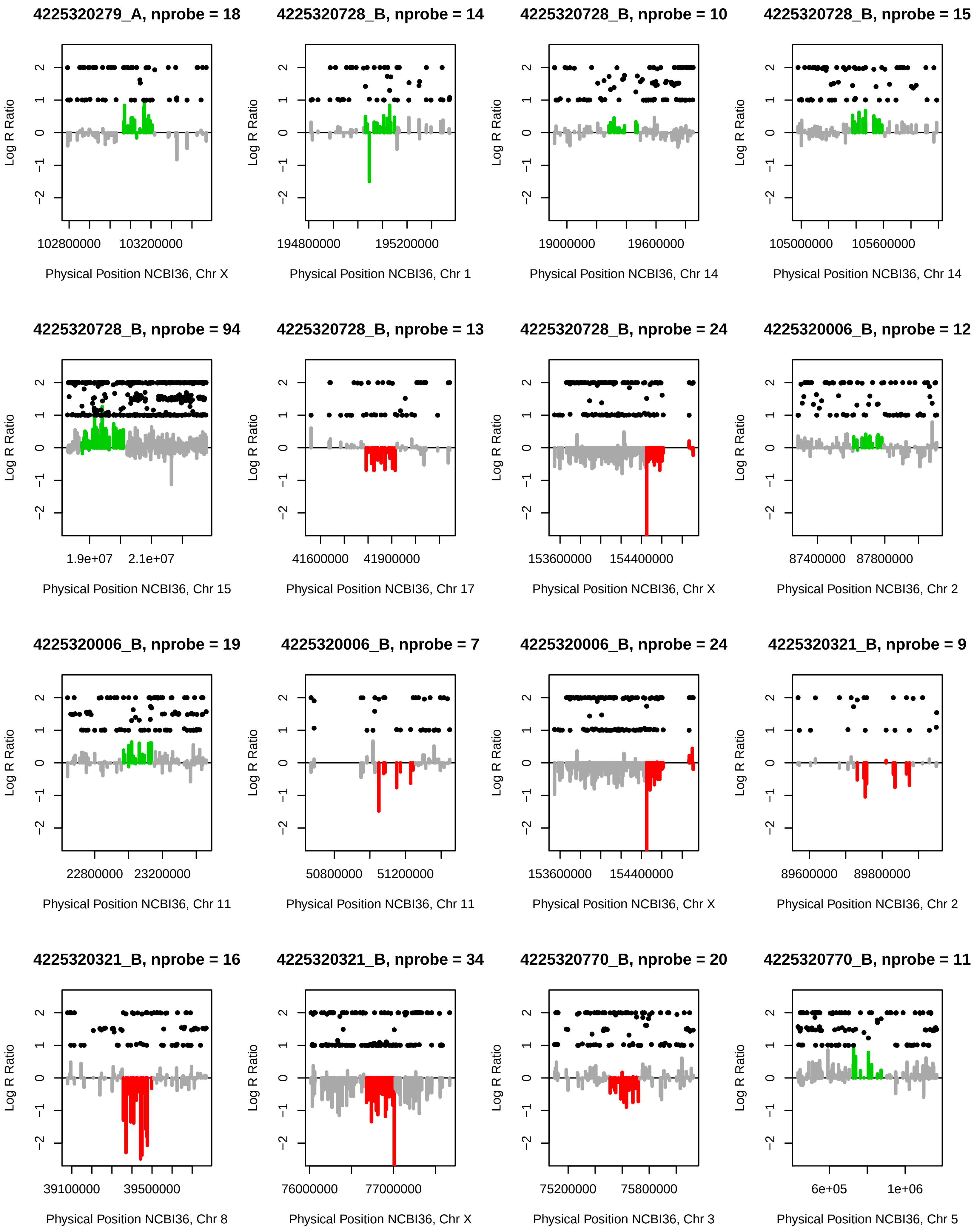
4225320279_A, nprobe = 18
Log R Ratio
102800000
103200000
Physical Position NCBI36, Chr X
4225320728_B, nprobe = 14
194800000
195200000
Physical Position NCBI36, Chr 1
4225320728_B, nprobe = 10
19000000
19600000
Physical Position NCBI36, Chr 14
4225320728_B, nprobe = 15
105000000
105600000
Physical Position NCBI36, Chr 14
4225320728_B, nprobe = 94
1.9e+07
2.1e+07
Physical Position NCBI36, Chr 15
4225320728_B, nprobe = 13
41600000
41900000
Physical Position NCBI36, Chr 17
4225320728_B, nprobe = 24
153600000
154400000
Physical Position NCBI36, Chr X
4225320006_B, nprobe = 12
87400000
87800000
Physical Position NCBI36, Chr 2
4225320006_B, nprobe = 19
22800000
23200000
Physical Position NCBI36, Chr 11
4225320006_B, nprobe = 7
50800000
51200000
Physical Position NCBI36, Chr 11
4225320006_B, nprobe = 24
153600000
154400000
Physical Position NCBI36, Chr X
4225320321_B, nprobe = 9
89600000
89800000
Physical Position NCBI36, Chr 2
4225320321_B, nprobe = 16
39100000
39500000
Physical Position NCBI36, Chr 8
4225320321_B, nprobe = 34
76000000
77000000
Physical Position NCBI36, Chr X
4225320770_B, nprobe = 20
75200000
75800000
Physical Position NCBI36, Chr 3
4225320770_B, nprobe = 11
6e+05
1e+06
Physical Position NCBI36, Chr 5

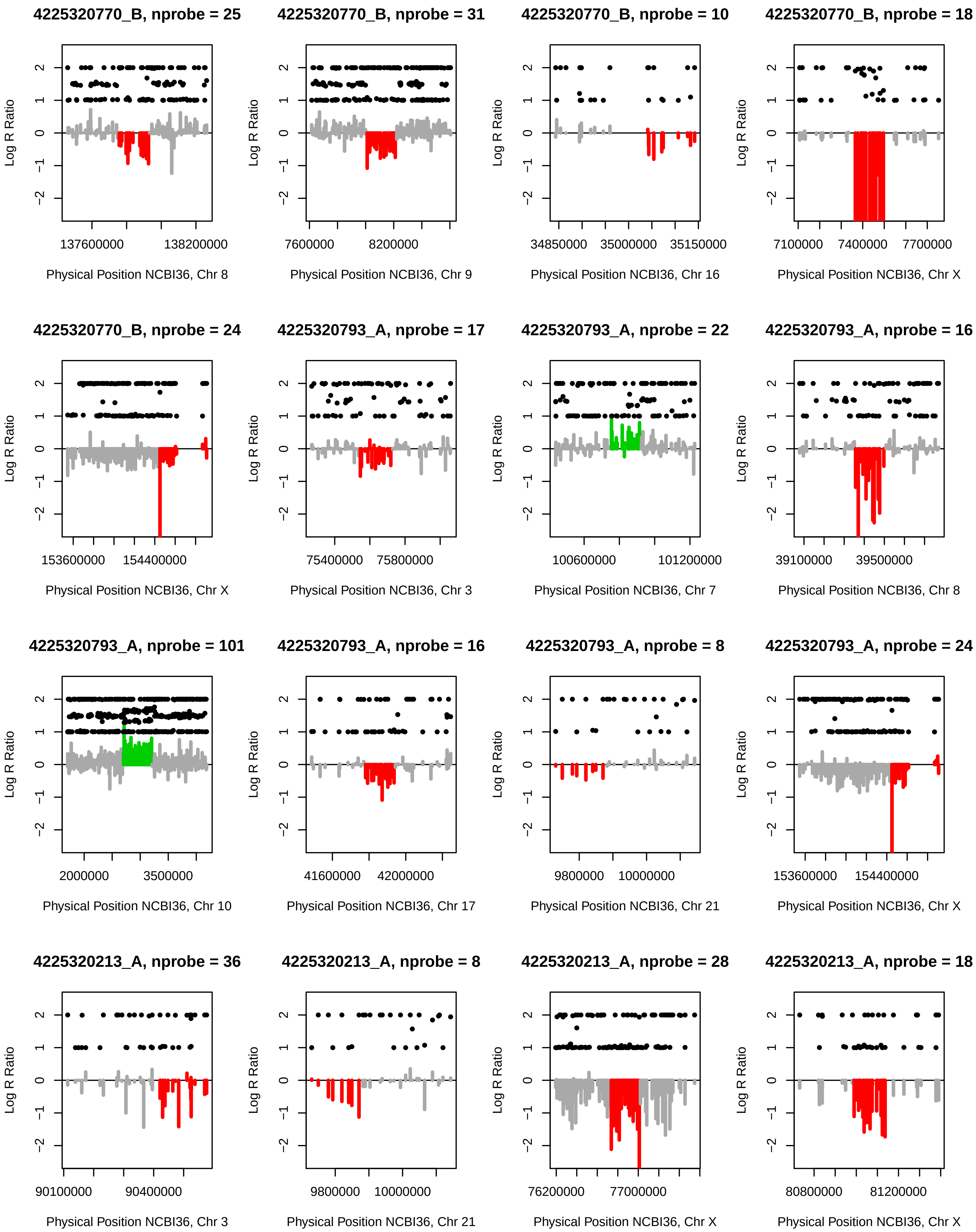
4225320770_B, nprobe = 25
Log R Ratio
137600000
138200000
Physical Position NCBI36, Chr 8
4225320770_B, nprobe = 31
7600000
8200000
Physical Position NCBI36, Chr 9
4225320770_B, nprobe = 10
34850000
35000000
35150000
Physical Position NCBI36, Chr 16
4225320770_B, nprobe = 18
7100000
7400000
7700000
Physical Position NCBI36, Chr X
4225320770_B, nprobe = 24
153600000
154400000
Physical Position NCBI36, Chr X
4225320793_A, nprobe = 17
75400000
75800000
Physical Position NCBI36, Chr 3
4225320793_A, nprobe = 22
100600000
101200000
Physical Position NCBI36, Chr 7
4225320793_A, nprobe = 16
39100000
39500000
Physical Position NCBI36, Chr 8
4225320793_A, nprobe = 101
2000000
3500000
Physical Position NCBI36, Chr 10
4225320793_A, nprobe = 16
41600000
42000000
Physical Position NCBI36, Chr 17
4225320793_A, nprobe = 8
9800000
10000000
Physical Position NCBI36, Chr 21
4225320793_A, nprobe = 24
153600000
154400000
Physical Position NCBI36, Chr X
4225320213_A, nprobe = 36
90100000
90400000
Physical Position NCBI36, Chr 3
4225320213_A, nprobe = 8
9800000
10000000
Physical Position NCBI36, Chr 21
4225320213_A, nprobe = 28
76200000
77000000
Physical Position NCBI36, Chr X
4225320213_A, nprobe = 18
80800000
81200000
Physical Position NCBI36, Chr X

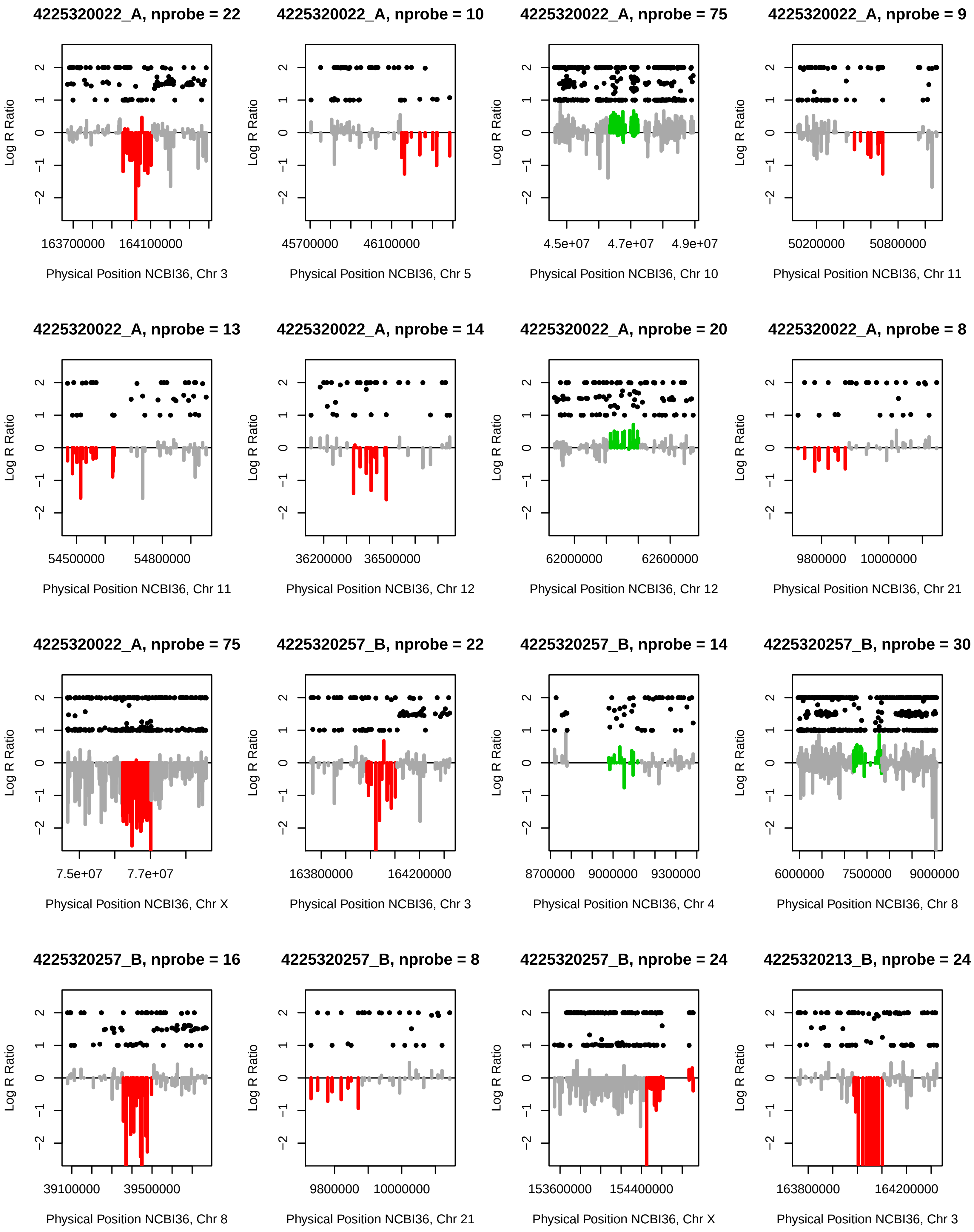
4225320022_A, nprobe = 22
Log R Ratio
163700000
164100000
Physical Position NCBI36, Chr 3
4225320022_A, nprobe = 10
Log R Ratio
45700000
46100000
Physical Position NCBI36, Chr 5
4225320022_A, nprobe = 75
Log R Ratio
4.5e+07
4.7e+07
4.9e+07
Physical Position NCBI36, Chr 10
4225320022_A, nprobe = 9
Log R Ratio
50200000
50800000
Physical Position NCBI36, Chr 11
4225320022_A, nprobe = 13
Log R Ratio
54500000
54800000
Physical Position NCBI36, Chr 11
4225320022_A, nprobe = 14
Log R Ratio
36200000
36500000
Physical Position NCBI36, Chr 12
4225320022_A, nprobe = 20
Log R Ratio
62000000
62600000
Physical Position NCBI36, Chr 12
4225320022_A, nprobe = 8
Log R Ratio
9800000
10000000
Physical Position NCBI36, Chr 21
4225320022_A, nprobe = 75
Log R Ratio
7.5e+07
7.7e+07
Physical Position NCBI36, Chr X
4225320257_B, nprobe = 22
Log R Ratio
163800000
164200000
Physical Position NCBI36, Chr 3
4225320257_B, nprobe = 14
Log R Ratio
8700000
9000000
9300000
Physical Position NCBI36, Chr 4
4225320257_B, nprobe = 30
Log R Ratio
6000000
7500000
9000000
Physical Position NCBI36, Chr 8
4225320257_B, nprobe = 16
Log R Ratio
39100000
39500000
Physical Position NCBI36, Chr 8
4225320257_B, nprobe = 8
Log R Ratio
9800000
10000000
Physical Position NCBI36, Chr 21
4225320257_B, nprobe = 24
Log R Ratio
153600000
154400000
Physical Position NCBI36, Chr X
4225320213_B, nprobe = 24
Log R Ratio
163800000
164200000
Physical Position NCBI36, Chr 3

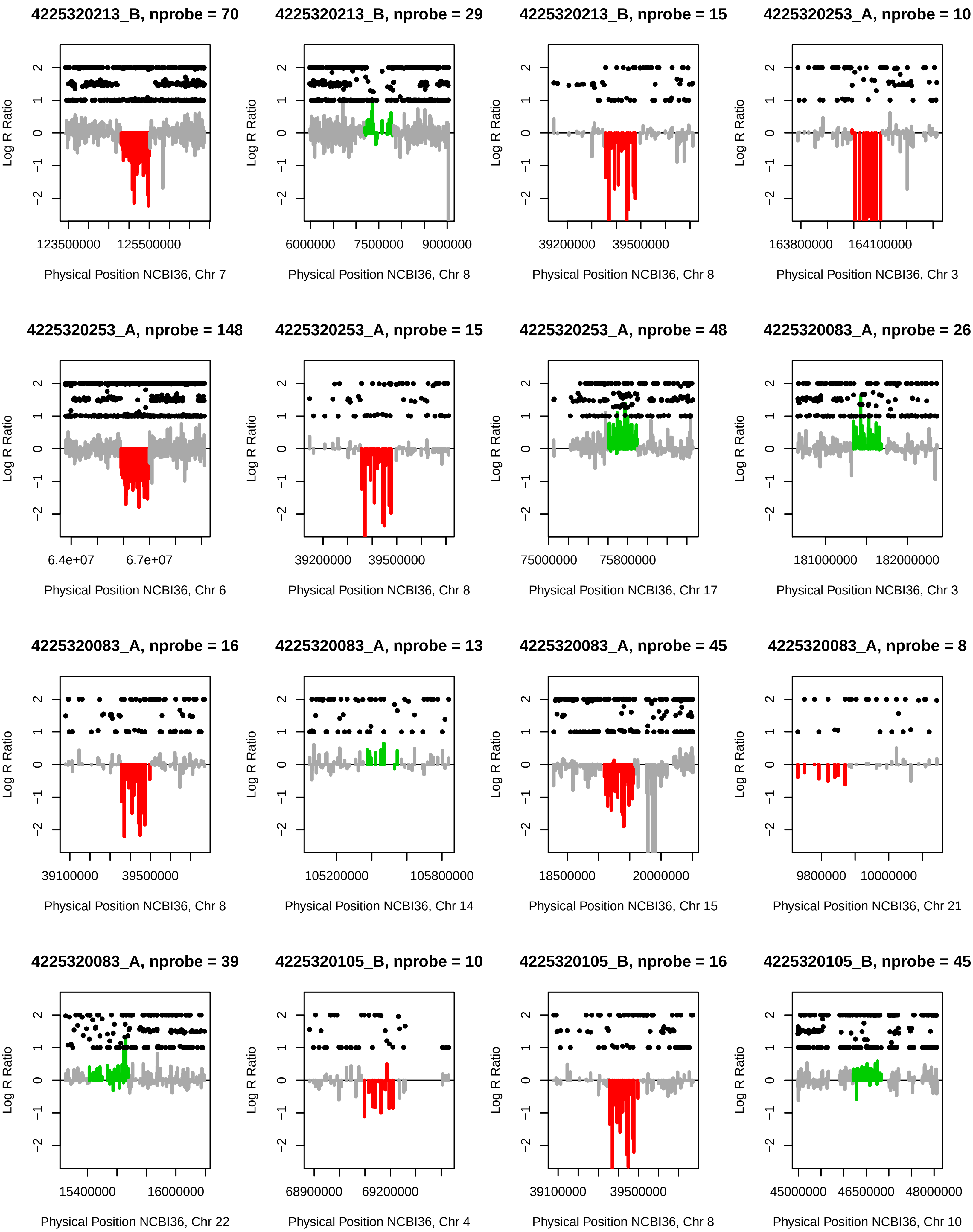
4225320213_B, nprobe = 70
Log R Ratio
2
1
0
−1
−2
123500000
125500000
Physical Position NCBI36, Chr 7
4225320213_B, nprobe = 29
6000000
7500000
9000000
Physical Position NCBI36, Chr 8
4225320213_B, nprobe = 15
39200000
39500000
Physical Position NCBI36, Chr 8
4225320253_A, nprobe = 10
163800000
164100000
Physical Position NCBI36, Chr 3
4225320253_A, nprobe = 148
6.4e+07
6.7e+07
Physical Position NCBI36, Chr 6
4225320253_A, nprobe = 15
39200000
39500000
Physical Position NCBI36, Chr 8
4225320253_A, nprobe = 48
75000000
75800000
Physical Position NCBI36, Chr 17
4225320083_A, nprobe = 26
181000000
182000000
Physical Position NCBI36, Chr 3
4225320083_A, nprobe = 16
39100000
39500000
Physical Position NCBI36, Chr 8
4225320083_A, nprobe = 13
105200000
105800000
Physical Position NCBI36, Chr 14
4225320083_A, nprobe = 45
18500000
20000000
Physical Position NCBI36, Chr 15
4225320083_A, nprobe = 8
9800000
10000000
Physical Position NCBI36, Chr 21
4225320083_A, nprobe = 39
15400000
16000000
Physical Position NCBI36, Chr 22
4225320105_B, nprobe = 10
68900000
69200000
Physical Position NCBI36, Chr 4
4225320105_B, nprobe = 16
39100000
39500000
Physical Position NCBI36, Chr 8
4225320105_B, nprobe = 45
45000000
46500000
48000000
Physical Position NCBI36, Chr 10

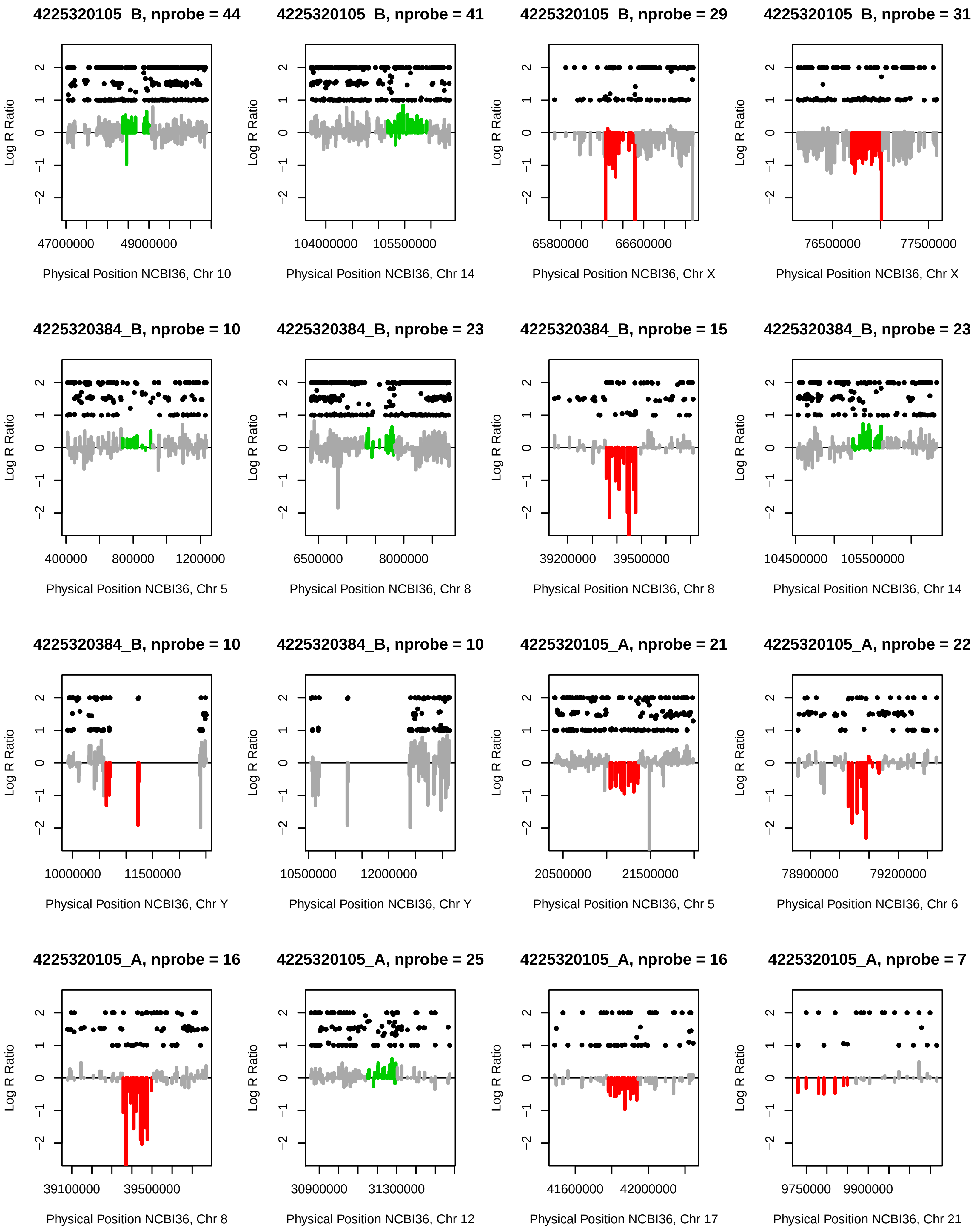

4225320105_B, nprobe = 44
Log R Ratio
47000000
49000000
Physical Position NCBI36, Chr 10
4225320105_B, nprobe = 41
Log R Ratio
104000000
105500000
Physical Position NCBI36, Chr 14
4225320105_B, nprobe = 29
Log R Ratio
65800000
66600000
Physical Position NCBI36, Chr X
4225320105_B, nprobe = 31
Log R Ratio
76500000
77500000
Physical Position NCBI36, Chr X
4225320384_B, nprobe = 10
Log R Ratio
400000
800000
1200000
Physical Position NCBI36, Chr 5
4225320384_B, nprobe = 23
Log R Ratio
6500000
8000000
Physical Position NCBI36, Chr 8
4225320384_B, nprobe = 15
Log R Ratio
39200000
39500000
Physical Position NCBI36, Chr 8
4225320384_B, nprobe = 23
Log R Ratio
104500000
105500000
Physical Position NCBI36, Chr 14
4225320384_B, nprobe = 10
Log R Ratio
10000000
11500000
Physical Position NCBI36, Chr Y
4225320384_B, nprobe = 10
Log R Ratio
10500000
12000000
Physical Position NCBI36, Chr Y
4225320105_A, nprobe = 21
Log R Ratio
20500000
21500000
Physical Position NCBI36, Chr 5
4225320105_A, nprobe = 22
Log R Ratio
78900000
79200000
Physical Position NCBI36, Chr 6
4225320105_A, nprobe = 16
Log R Ratio
39100000
39500000
Physical Position NCBI36, Chr 8
4225320105_A, nprobe = 25
Log R Ratio
30900000
31300000
Physical Position NCBI36, Chr 12
4225320105_A, nprobe = 16
Log R Ratio
41600000
42000000
Physical Position NCBI36, Chr 17
4225320105_A, nprobe = 7
Log R Ratio
9750000
9900000
Physical Position NCBI36, Chr 21

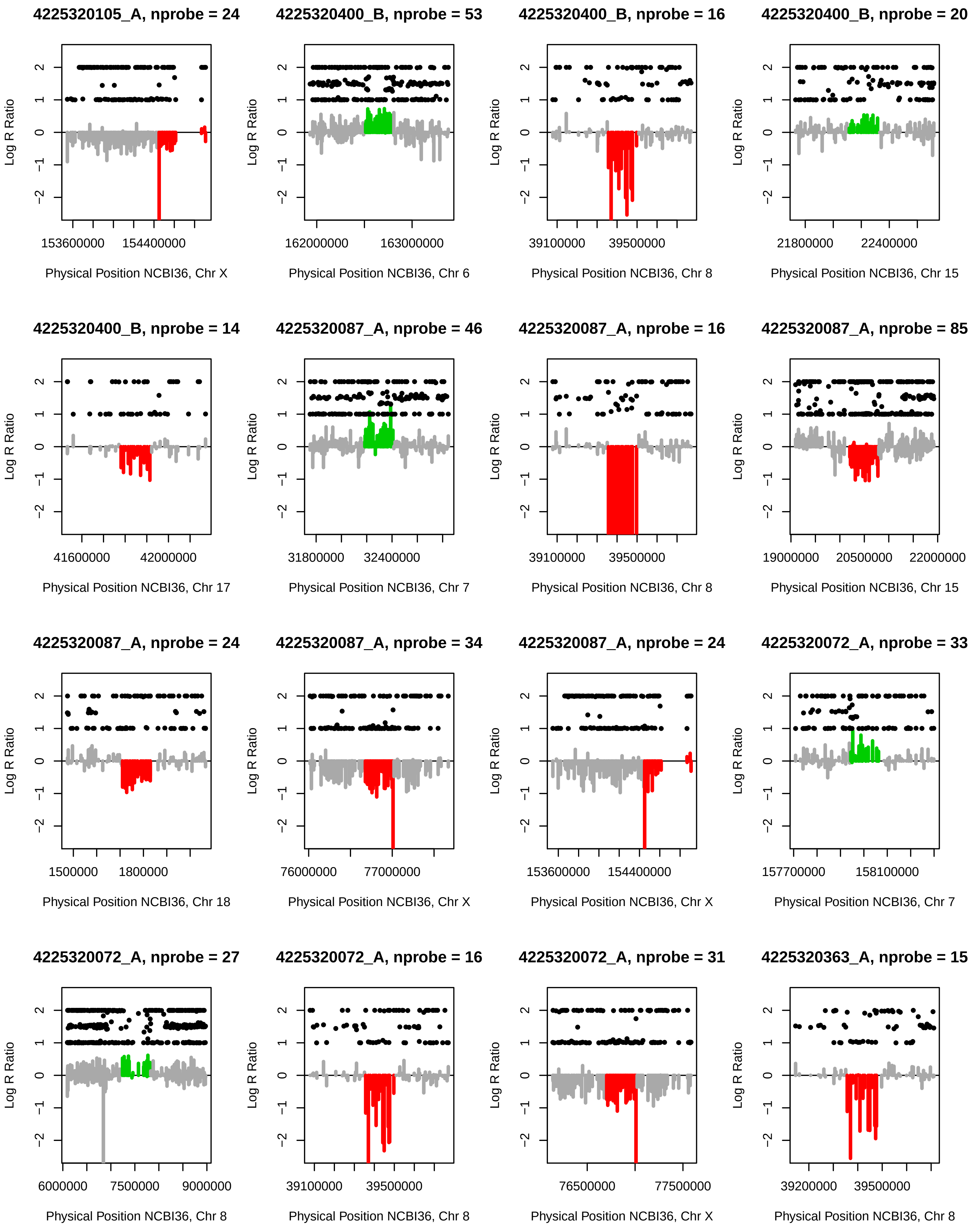
4225320105_A, nprobe = 24
Log R Ratio
153600000
154400000
Physical Position NCBI36, Chr X
4225320400_B, nprobe = 53
162000000
163000000
Physical Position NCBI36, Chr 6
4225320400_B, nprobe = 16
39100000
39500000
Physical Position NCBI36, Chr 8
4225320400_B, nprobe = 20
21800000
22400000
Physical Position NCBI36, Chr 15
4225320400_B, nprobe = 14
41600000
42000000
Physical Position NCBI36, Chr 17
4225320087_A, nprobe = 46
31800000
32400000
Physical Position NCBI36, Chr 7
4225320087_A, nprobe = 16
39100000
39500000
Physical Position NCBI36, Chr 8
4225320087_A, nprobe = 85
19000000
20500000
22000000
Physical Position NCBI36, Chr 15
4225320087_A, nprobe = 24
1500000
1800000
Physical Position NCBI36, Chr 18
4225320087_A, nprobe = 34
76000000
77000000
Physical Position NCBI36, Chr X
4225320087_A, nprobe = 24
153600000
154400000
Physical Position NCBI36, Chr X
4225320072_A, nprobe = 33
157700000
158100000
Physical Position NCBI36, Chr 7
4225320072_A, nprobe = 27
6000000
7500000
9000000
Physical Position NCBI36, Chr 8
4225320072_A, nprobe = 16
39100000
39500000
Physical Position NCBI36, Chr 8
4225320072_A, nprobe = 31
76500000
77500000
Physical Position NCBI36, Chr X
4225320363_A, nprobe = 15
39200000
39500000
Physical Position NCBI36, Chr 8

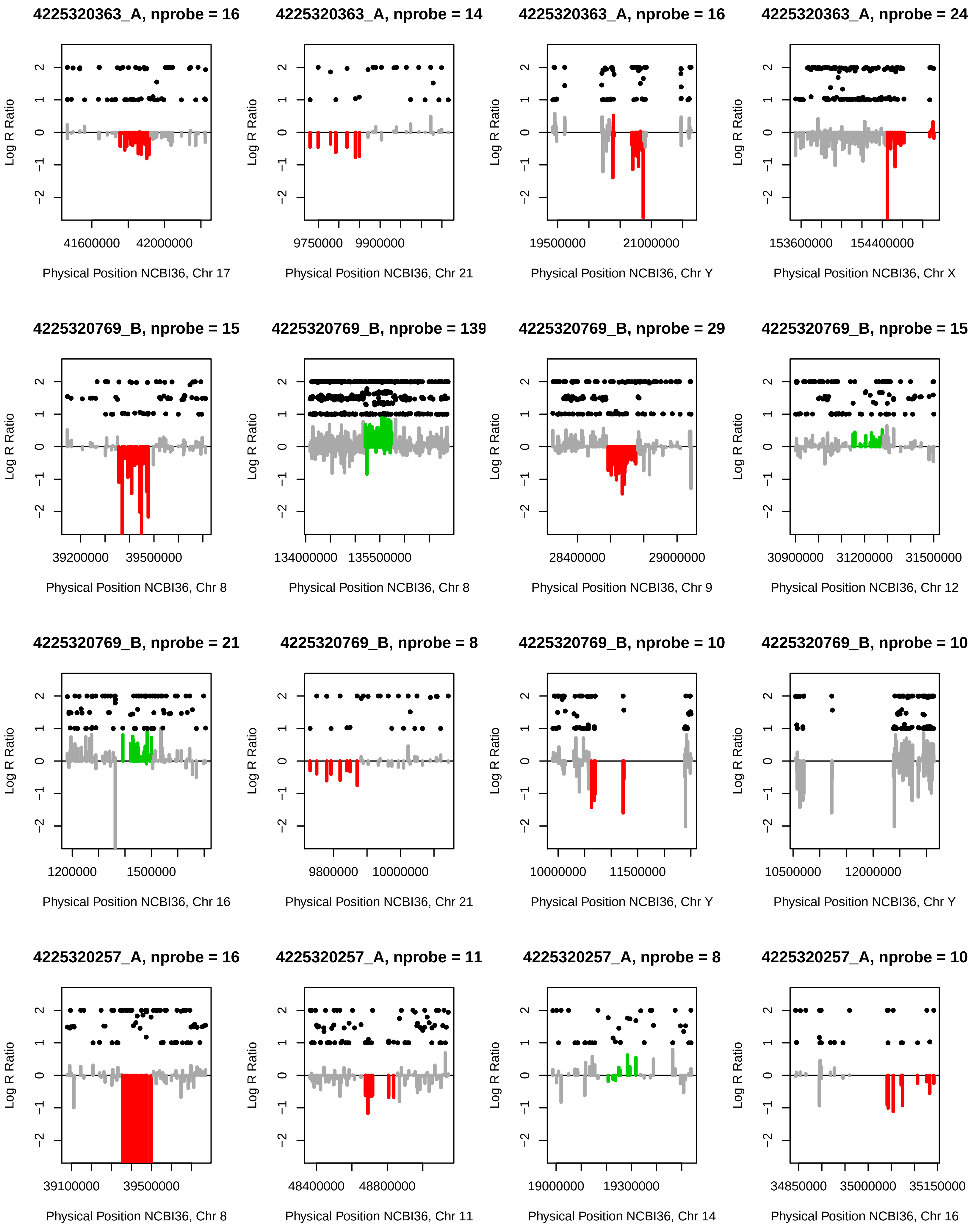
4225320363_A, nprobe = 16
Log R Ratio
41600000 42000000
Physical Position NCBI36, Chr 17
4225320363_A, nprobe = 14
Log R Ratio
9750000 9900000
Physical Position NCBI36, Chr 21
4225320363_A, nprobe = 16
Log R Ratio
19500000 21000000
Physical Position NCBI36, Chr Y
4225320363_A, nprobe = 24
Log R Ratio
153600000 154400000
Physical Position NCBI36, Chr X
4225320769_B, nprobe = 15
Log R Ratio
39200000 39500000
Physical Position NCBI36, Chr 8
4225320769_B, nprobe = 139
Log R Ratio
134000000 135500000
Physical Position NCBI36, Chr 8
4225320769_B, nprobe = 29
Log R Ratio
28400000 29000000
Physical Position NCBI36, Chr 9
4225320769_B, nprobe = 15
Log R Ratio
30900000 31200000 31500000
Physical Position NCBI36, Chr 12
4225320769_B, nprobe = 21
Log R Ratio
1200000 1500000
Physical Position NCBI36, Chr 16
4225320769_B, nprobe = 8
Log R Ratio
9800000 10000000
Physical Position NCBI36, Chr 21
4225320769_B, nprobe = 10
Log R Ratio
10000000 11500000
Physical Position NCBI36, Chr Y
4225320769_B, nprobe = 10
Log R Ratio
10500000 12000000
Physical Position NCBI36, Chr Y
4225320257_A, nprobe = 16
Log R Ratio
39100000 39500000
Physical Position NCBI36, Chr 8
4225320257_A, nprobe = 11
Log R Ratio
48400000 48800000
Physical Position NCBI36, Chr 11
4225320257_A, nprobe = 8
Log R Ratio
19000000 19300000
Physical Position NCBI36, Chr 14
4225320257_A, nprobe = 10
Log R Ratio
34850000 35000000 35150000
Physical Position NCBI36, Chr 16

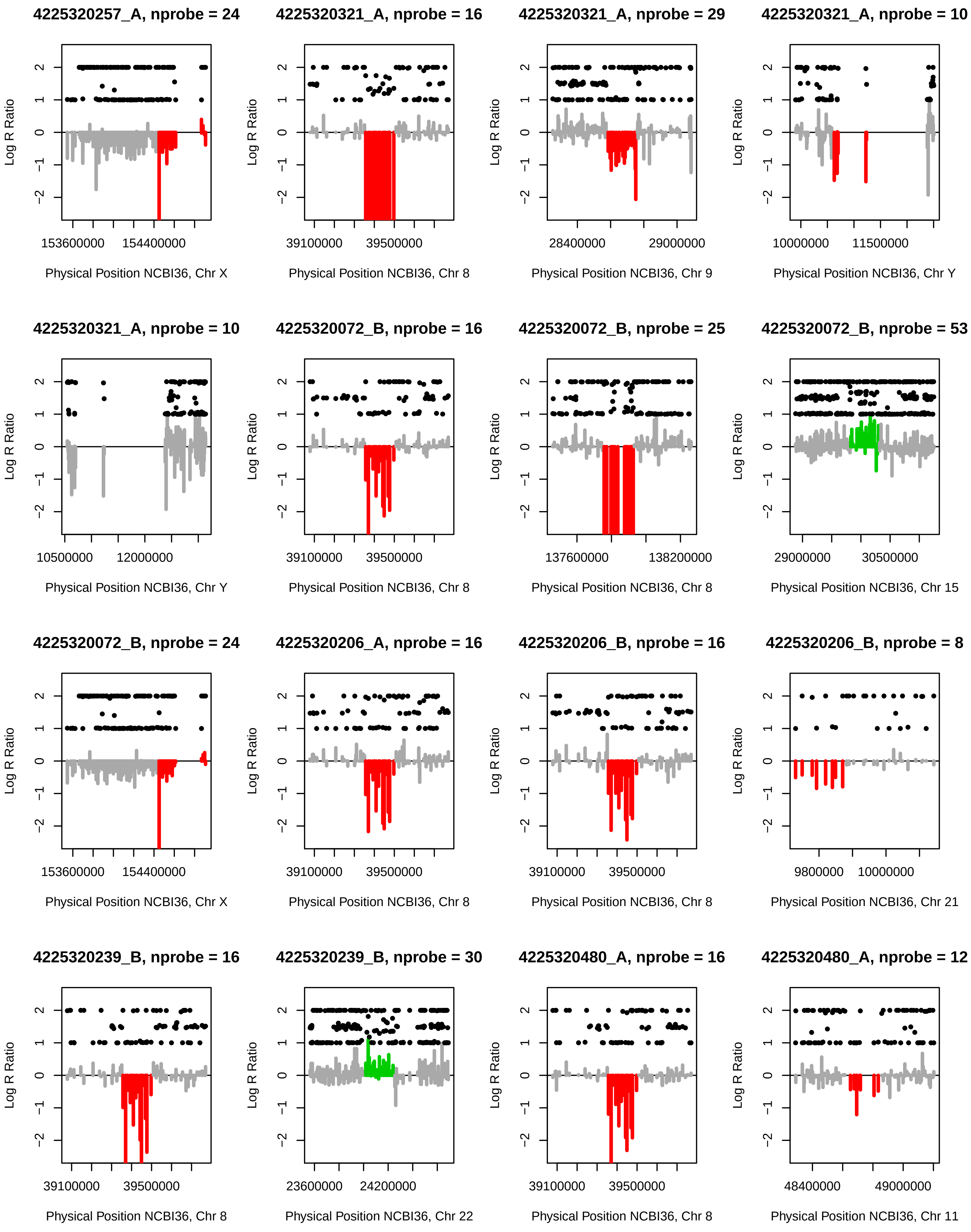
4225320257_A, nprobe = 24
Log R Ratio
153600000
154400000
Physical Position NCBI36, Chr X
4225320321_A, nprobe = 16
39100000
39500000
Physical Position NCBI36, Chr 8
4225320321_A, nprobe = 29
28400000
29000000
Physical Position NCBI36, Chr 9
4225320321_A, nprobe = 10
10000000
11500000
Physical Position NCBI36, Chr Y
4225320321_A, nprobe = 10
10500000
12000000
Physical Position NCBI36, Chr Y
4225320072_B, nprobe = 16
39100000
39500000
Physical Position NCBI36, Chr 8
4225320072_B, nprobe = 25
137600000
138200000
Physical Position NCBI36, Chr 8
4225320072_B, nprobe = 53
29000000
30500000
Physical Position NCBI36, Chr 15
4225320072_B, nprobe = 24
153600000
154400000
Physical Position NCBI36, Chr X
4225320206_A, nprobe = 16
39100000
39500000
Physical Position NCBI36, Chr 8
4225320206_B, nprobe = 16
39100000
39500000
Physical Position NCBI36, Chr 8
4225320206_B, nprobe = 8
9800000
10000000
Physical Position NCBI36, Chr 21
4225320239_B, nprobe = 16
39100000
39500000
Physical Position NCBI36, Chr 8
4225320239_B, nprobe = 30
23600000
24200000
Physical Position NCBI36, Chr 22
4225320480_A, nprobe = 16
39100000
39500000
Physical Position NCBI36, Chr 8
4225320480_A, nprobe = 12
48400000
49000000
Physical Position NCBI36, Chr 11

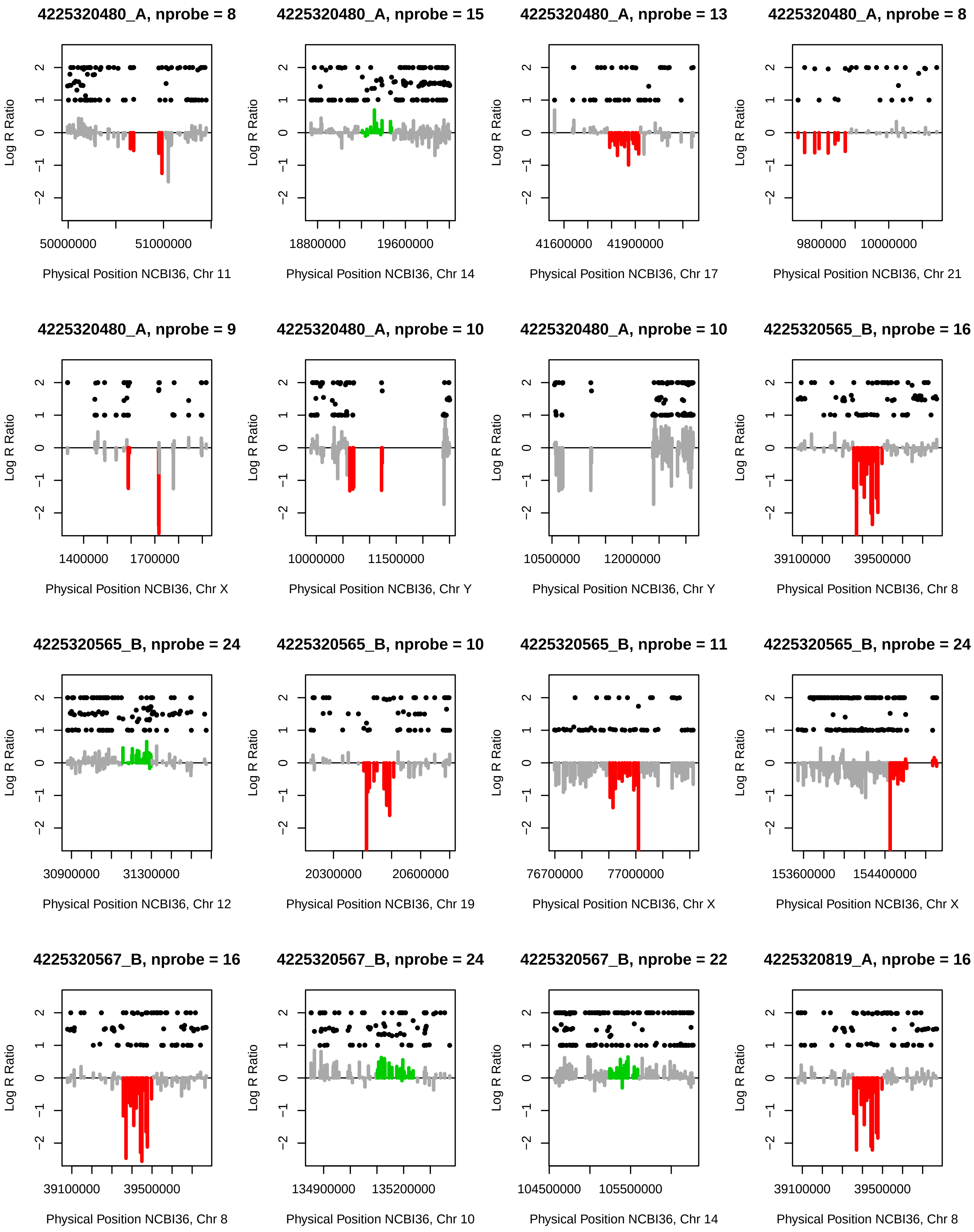
4225320480_A, nprobe = 8
Log R Ratio
50000000
51000000
Physical Position NCBI36, Chr 11
4225320480_A, nprobe = 15
18800000
19600000
Physical Position NCBI36, Chr 14
4225320480_A, nprobe = 13
41600000
41900000
Physical Position NCBI36, Chr 17
4225320480_A, nprobe = 8
9800000
10000000
Physical Position NCBI36, Chr 21
4225320480_A, nprobe = 9
1400000
1700000
Physical Position NCBI36, Chr X
4225320480_A, nprobe = 10
10000000
11500000
Physical Position NCBI36, Chr Y
4225320480_A, nprobe = 10
10500000
12000000
Physical Position NCBI36, Chr Y
4225320565_B, nprobe = 16
39100000
39500000
Physical Position NCBI36, Chr 8
4225320565_B, nprobe = 24
30900000
31300000
Physical Position NCBI36, Chr 12
4225320565_B, nprobe = 10
20300000
20600000
Physical Position NCBI36, Chr 19
4225320565_B, nprobe = 11
76700000
77000000
Physical Position NCBI36, Chr X
4225320565_B, nprobe = 24
153600000
154400000
Physical Position NCBI36, Chr X
4225320567_B, nprobe = 16
39100000
39500000
Physical Position NCBI36, Chr 8
4225320567_B, nprobe = 24
134900000
135200000
Physical Position NCBI36, Chr 10
4225320567_B, nprobe = 22
104500000
105500000
Physical Position NCBI36, Chr 14
4225320819_A, nprobe = 16
39100000
39500000
Physical Position NCBI36, Chr 8

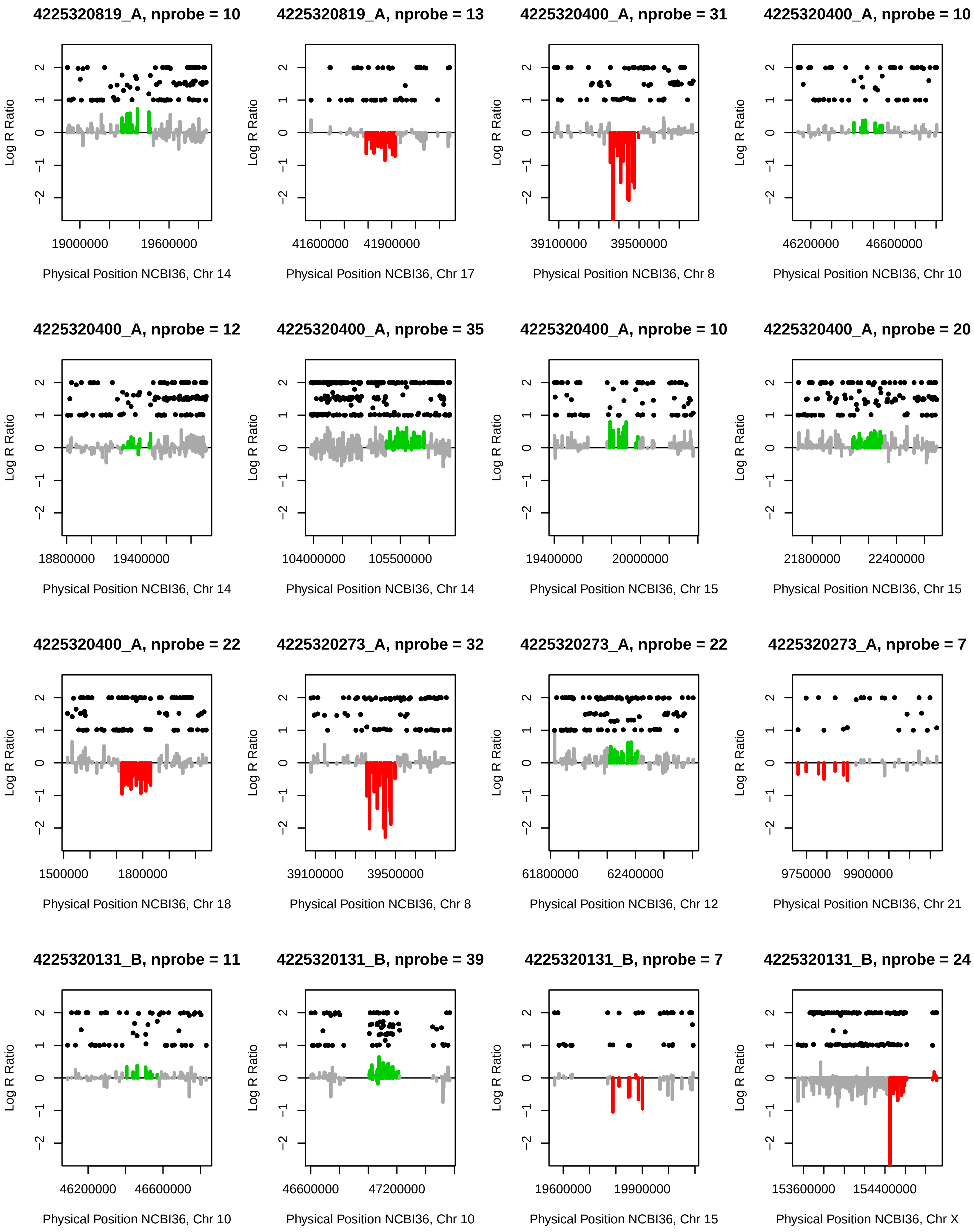
4225320819_A, nprobe = 10
Log R Ratio
19000000
19600000
Physical Position NCBI36, Chr 14
4225320819_A, nprobe = 13
Log R Ratio
41600000
41900000
Physical Position NCBI36, Chr 17
4225320400_A, nprobe = 31
Log R Ratio
39100000
39500000
Physical Position NCBI36, Chr 8
4225320400_A, nprobe = 10
Log R Ratio
46200000
46600000
Physical Position NCBI36, Chr 10
4225320400_A, nprobe = 12
Log R Ratio
18800000
19400000
Physical Position NCBI36, Chr 14
4225320400_A, nprobe = 35
Log R Ratio
104000000
105500000
Physical Position NCBI36, Chr 14
4225320400_A, nprobe = 10
Log R Ratio
19400000
20000000
Physical Position NCBI36, Chr 15
4225320400_A, nprobe = 20
Log R Ratio
21800000
22400000
Physical Position NCBI36, Chr 15
4225320400_A, nprobe = 22
Log R Ratio
1500000
1800000
Physical Position NCBI36, Chr 18
4225320273_A, nprobe = 32
Log R Ratio
39100000
39500000
Physical Position NCBI36, Chr 8
4225320273_A, nprobe = 22
Log R Ratio
61800000
62400000
Physical Position NCBI36, Chr 12
4225320273_A, nprobe = 7
Log R Ratio
9750000
9900000
Physical Position NCBI36, Chr 21
4225320131_B, nprobe = 11
Log R Ratio
46200000
46600000
Physical Position NCBI36, Chr 10
4225320131_B, nprobe = 39
Log R Ratio
46600000
47200000
Physical Position NCBI36, Chr 10
4225320131_B, nprobe = 7
Log R Ratio
19600000
19900000
Physical Position NCBI36, Chr 15
4225320131_B, nprobe = 24
Log R Ratio
153600000
154400000
Physical Position NCBI36, Chr X

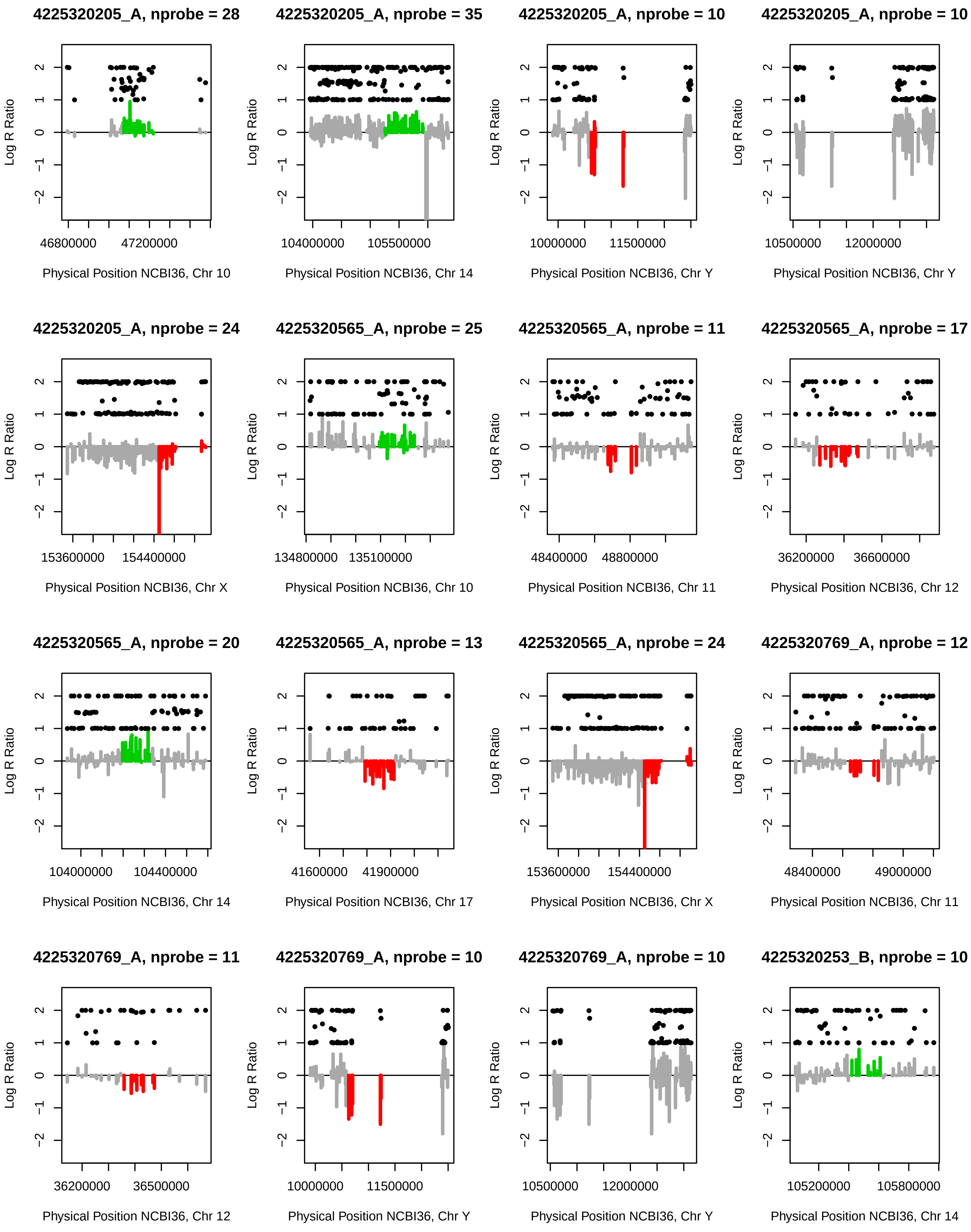

4225320205_A, nprobe = 28
4225320205_A, nprobe = 35
4225320205_A, nprobe = 10
4225320205_A, nprobe = 10
Log R Ratio
46800000
47200000
Physical Position NCBI36, Chr 10
104000000
105500000
Physical Position NCBI36, Chr 14
10000000
11500000
Physical Position NCBI36, Chr Y
10500000
12000000
Physical Position NCBI36, Chr Y
4225320205_A, nprobe = 24
4225320565_A, nprobe = 25
4225320565_A, nprobe = 11
4225320565_A, nprobe = 17
153600000
154400000
Physical Position NCBI36, Chr X
134800000
135100000
Physical Position NCBI36, Chr 10
48400000
48800000
Physical Position NCBI36, Chr 11
36200000
36600000
Physical Position NCBI36, Chr 12
4225320565_A, nprobe = 20
4225320565_A, nprobe = 13
4225320565_A, nprobe = 24
4225320769_A, nprobe = 12
104000000
104400000
Physical Position NCBI36, Chr 14
41600000
41900000
Physical Position NCBI36, Chr 17
153600000
154400000
Physical Position NCBI36, Chr X
48400000
49000000
Physical Position NCBI36, Chr 11
4225320769_A, nprobe = 11
4225320769_A, nprobe = 10
4225320769_A, nprobe = 10
4225320253_B, nprobe = 10
36200000
36500000
Physical Position NCBI36, Chr 12
10000000
11500000
Physical Position NCBI36, Chr Y
10500000
12000000
Physical Position NCBI36, Chr Y
105200000
105800000
Physical Position NCBI36, Chr 14

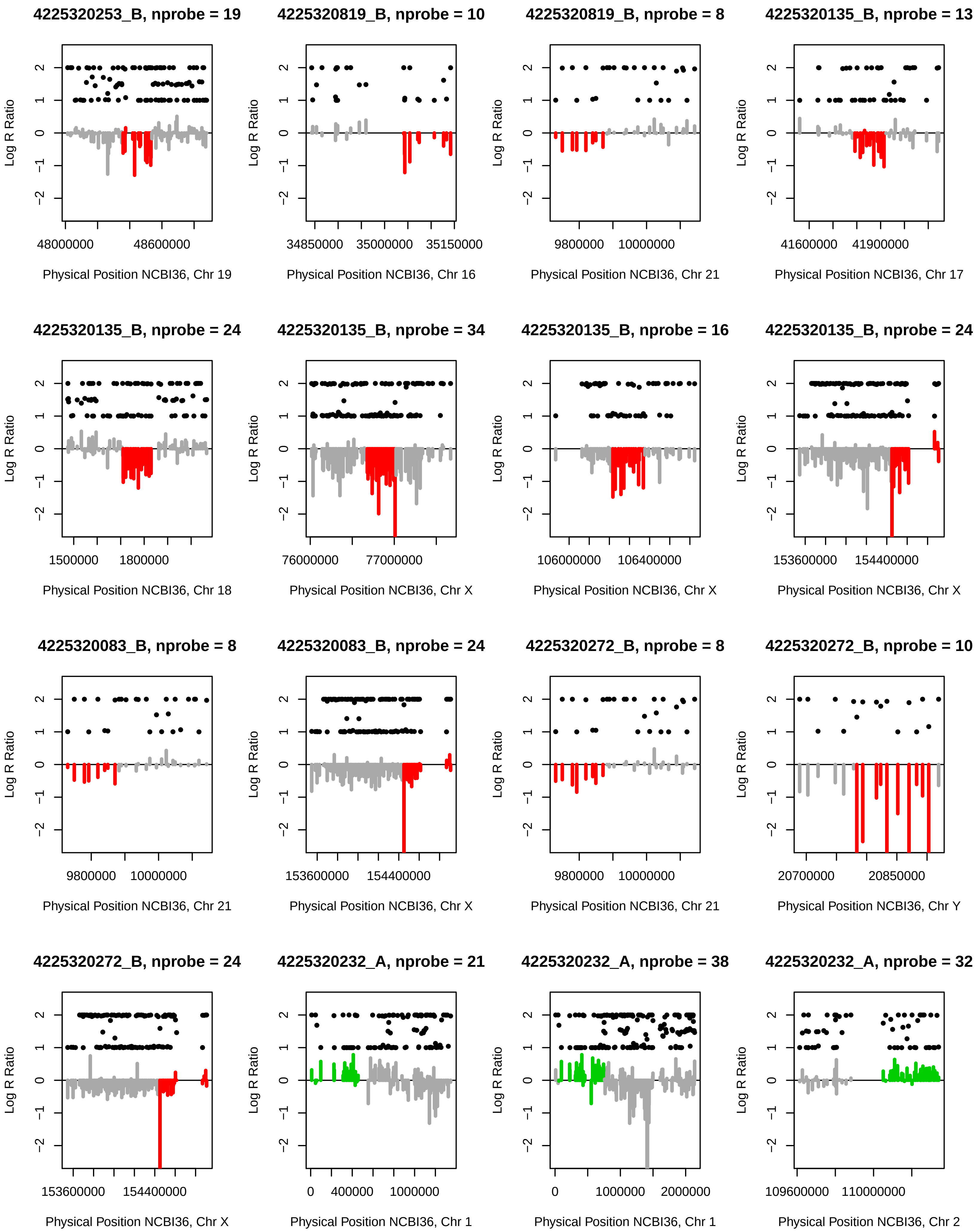

4225320253_B, nprobe = 19
Log R Ratio
2 1 0 −1 −2
48000000 48600000
Physical Position NCBI36, Chr 19
4225320819_B, nprobe = 10
34850000 35000000 35150000
Physical Position NCBI36, Chr 16
4225320819_B, nprobe = 8
9800000 10000000
Physical Position NCBI36, Chr 21
4225320135_B, nprobe = 13
41600000 41900000
Physical Position NCBI36, Chr 17
4225320135_B, nprobe = 24
1500000 1800000
Physical Position NCBI36, Chr 18
4225320135_B, nprobe = 34
76000000 77000000
Physical Position NCBI36, Chr X
4225320135_B, nprobe = 16
106000000 106400000
Physical Position NCBI36, Chr X
4225320135_B, nprobe = 24
153600000 154400000
Physical Position NCBI36, Chr X
4225320083_B, nprobe = 8
9800000 10000000
Physical Position NCBI36, Chr 21
4225320083_B, nprobe = 24
153600000 154400000
Physical Position NCBI36, Chr X
4225320272_B, nprobe = 8
9800000 10000000
Physical Position NCBI36, Chr 21
4225320272_B, nprobe = 10
20700000 20850000
Physical Position NCBI36, Chr Y
4225320272_B, nprobe = 24
153600000 154400000
Physical Position NCBI36, Chr X
4225320232_A, nprobe = 21
0 400000 1000000
Physical Position NCBI36, Chr 1
4225320232_A, nprobe = 38
0 1000000 2000000
Physical Position NCBI36, Chr 1
4225320232_A, nprobe = 32
109600000 110000000
Physical Position NCBI36, Chr 2

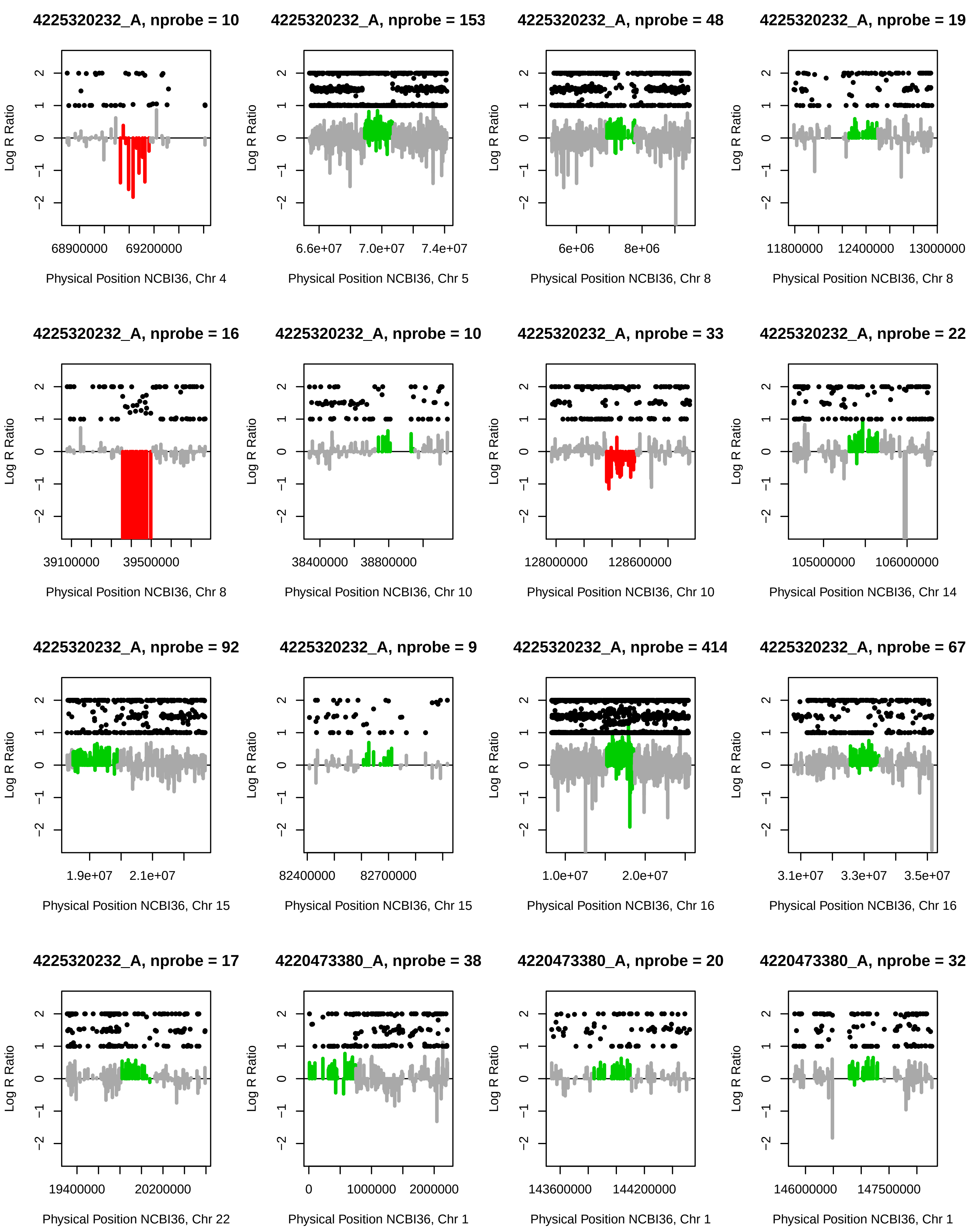
4225320232_A, nprobe = 10
Log R Ratio
68900000
69200000
Physical Position NCBI36, Chr 4
4225320232_A, nprobe = 153
Log R Ratio
6.6e+07
7.0e+07
7.4e+07
Physical Position NCBI36, Chr 5
4225320232_A, nprobe = 48
Log R Ratio
6e+06
8e+06
Physical Position NCBI36, Chr 8
4225320232_A, nprobe = 19
Log R Ratio
11800000
12400000
13000000
Physical Position NCBI36, Chr 8
4225320232_A, nprobe = 16
Log R Ratio
39100000
39500000
Physical Position NCBI36, Chr 8
4225320232_A, nprobe = 10
Log R Ratio
38400000
38800000
Physical Position NCBI36, Chr 10
4225320232_A, nprobe = 33
Log R Ratio
128000000
128600000
Physical Position NCBI36, Chr 10
4225320232_A, nprobe = 22
Log R Ratio
105000000
106000000
Physical Position NCBI36, Chr 14
4225320232_A, nprobe = 92
Log R Ratio
1.9e+07
2.1e+07
Physical Position NCBI36, Chr 15
4225320232_A, nprobe = 9
Log R Ratio
82400000
82700000
Physical Position NCBI36, Chr 15
4225320232_A, nprobe = 414
Log R Ratio
1.0e+07
2.0e+07
Physical Position NCBI36, Chr 16
4225320232_A, nprobe = 67
Log R Ratio
3.1e+07
3.3e+07
3.5e+07
Physical Position NCBI36, Chr 16
4225320232_A, nprobe = 17
Log R Ratio
19400000
20200000
Physical Position NCBI36, Chr 22
4220473380_A, nprobe = 38
Log R Ratio
0
1000000
2000000
Physical Position NCBI36, Chr 1
4220473380_A, nprobe = 20
Log R Ratio
143600000
144200000
Physical Position NCBI36, Chr 1
4220473380_A, nprobe = 32
Log R Ratio
146000000
147500000
Physical Position NCBI36, Chr 1

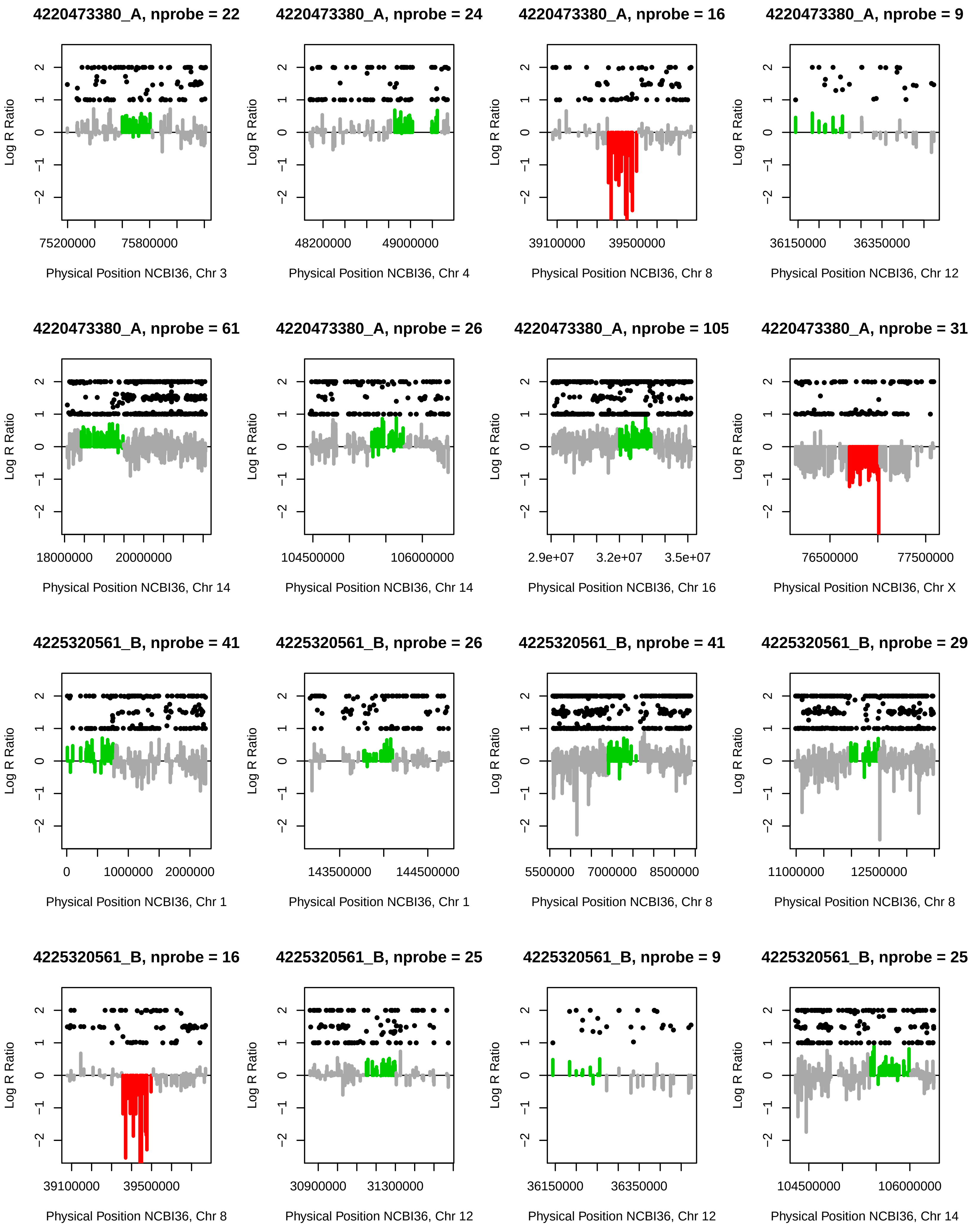
4220473380_A, nprobe = 22
Log R Ratio
75200000
75800000
Physical Position NCBI36, Chr 3
4220473380_A, nprobe = 24
48200000
49000000
Physical Position NCBI36, Chr 4
4220473380_A, nprobe = 16
39100000
39500000
Physical Position NCBI36, Chr 8
4220473380_A, nprobe = 9
36150000
36350000
Physical Position NCBI36, Chr 12
4220473380_A, nprobe = 61
18000000
20000000
Physical Position NCBI36, Chr 14
4220473380_A, nprobe = 26
104500000
106000000
Physical Position NCBI36, Chr 14
4220473380_A, nprobe = 105
2.9e+07
3.2e+07
3.5e+07
Physical Position NCBI36, Chr 16
4220473380_A, nprobe = 31
76500000
77500000
Physical Position NCBI36, Chr X
4225320561_B, nprobe = 41
0
1000000
2000000
Physical Position NCBI36, Chr 1
4225320561_B, nprobe = 26
143500000
144500000
Physical Position NCBI36, Chr 1
4225320561_B, nprobe = 41
5500000
7000000
8500000
Physical Position NCBI36, Chr 8
4225320561_B, nprobe = 29
11000000
12500000
Physical Position NCBI36, Chr 8
4225320561_B, nprobe = 16
39100000
39500000
Physical Position NCBI36, Chr 8
4225320561_B, nprobe = 25
30900000
31300000
Physical Position NCBI36, Chr 12
4225320561_B, nprobe = 9
36150000
36350000
Physical Position NCBI36, Chr 12
4225320561_B, nprobe = 25
104500000
106000000
Physical Position NCBI36, Chr 14

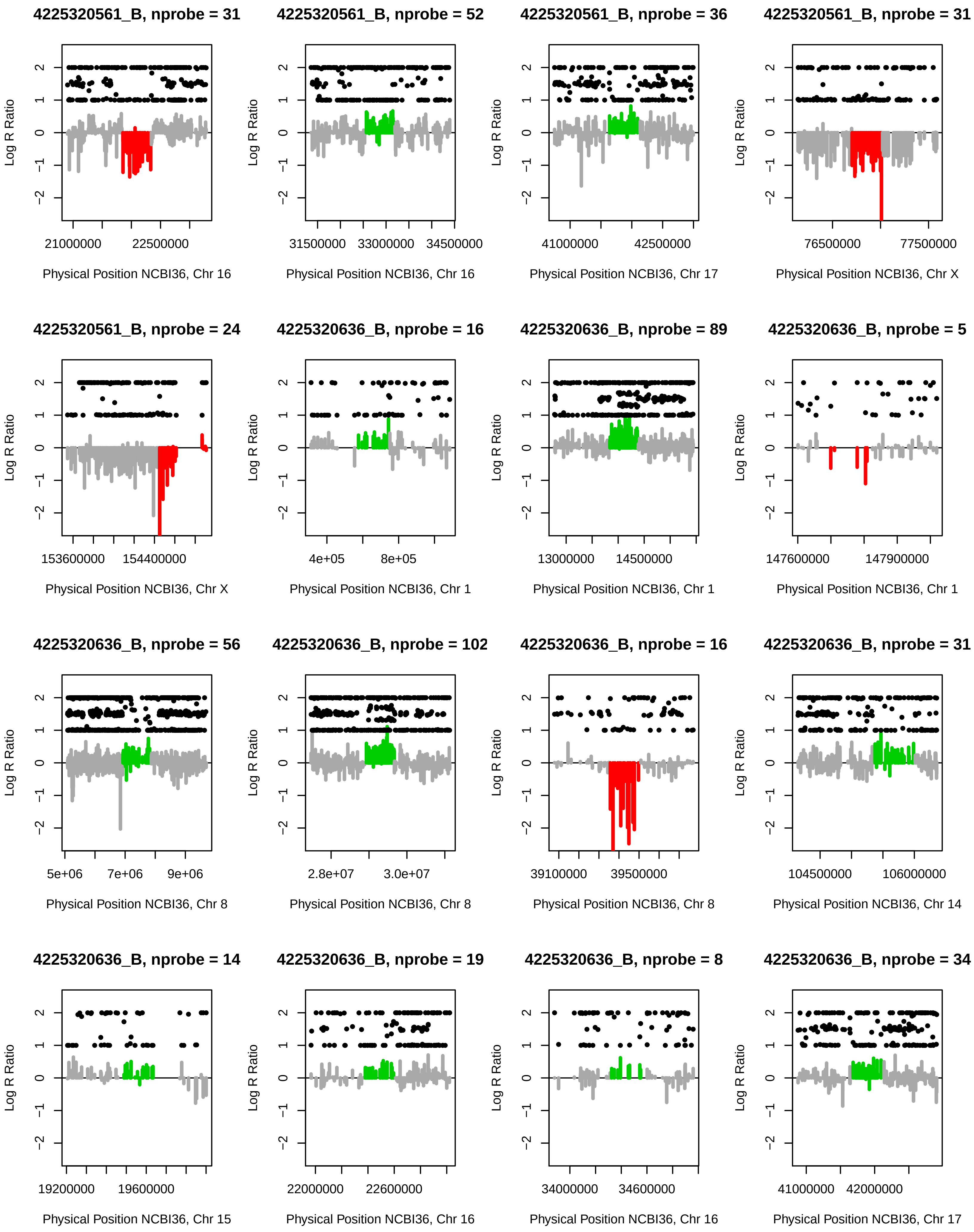
4225320561_B, nprobe = 31
Log R Ratio
Physical Position NCBI36, Chr 16
21000000
22500000
4225320561_B, nprobe = 52
Log R Ratio
Physical Position NCBI36, Chr 16
31500000
33000000
34500000
4225320561_B, nprobe = 36
Log R Ratio
Physical Position NCBI36, Chr 17
41000000
42500000
4225320561_B, nprobe = 31
Log R Ratio
Physical Position NCBI36, Chr X
76500000
77500000
4225320561_B, nprobe = 24
Log R Ratio
Physical Position NCBI36, Chr X
153600000
154400000
4225320636_B, nprobe = 16
Log R Ratio
Physical Position NCBI36, Chr 1
4e+05
8e+05
4225320636_B, nprobe = 89
Log R Ratio
Physical Position NCBI36, Chr 1
13000000
14500000
4225320636_B, nprobe = 5
Log R Ratio
Physical Position NCBI36, Chr 1
147600000
147900000
4225320636_B, nprobe = 56
Log R Ratio
Physical Position NCBI36, Chr 8
5e+06
7e+06
9e+06
4225320636_B, nprobe = 102
Log R Ratio
Physical Position NCBI36, Chr 8
2.8e+07
3.0e+07
4225320636_B, nprobe = 16
Log R Ratio
Physical Position NCBI36, Chr 8
39100000
39500000
4225320636_B, nprobe = 31
Log R Ratio
Physical Position NCBI36, Chr 14
104500000
106000000
4225320636_B, nprobe = 14
Log R Ratio
Physical Position NCBI36, Chr 15
19200000
19600000
4225320636_B, nprobe = 19
Log R Ratio
Physical Position NCBI36, Chr 16
22000000
22600000
4225320636_B, nprobe = 8
Log R Ratio
Physical Position NCBI36, Chr 16
34000000
34600000
4225320636_B, nprobe = 34
Log R Ratio
Physical Position NCBI36, Chr 17
41000000
42000000

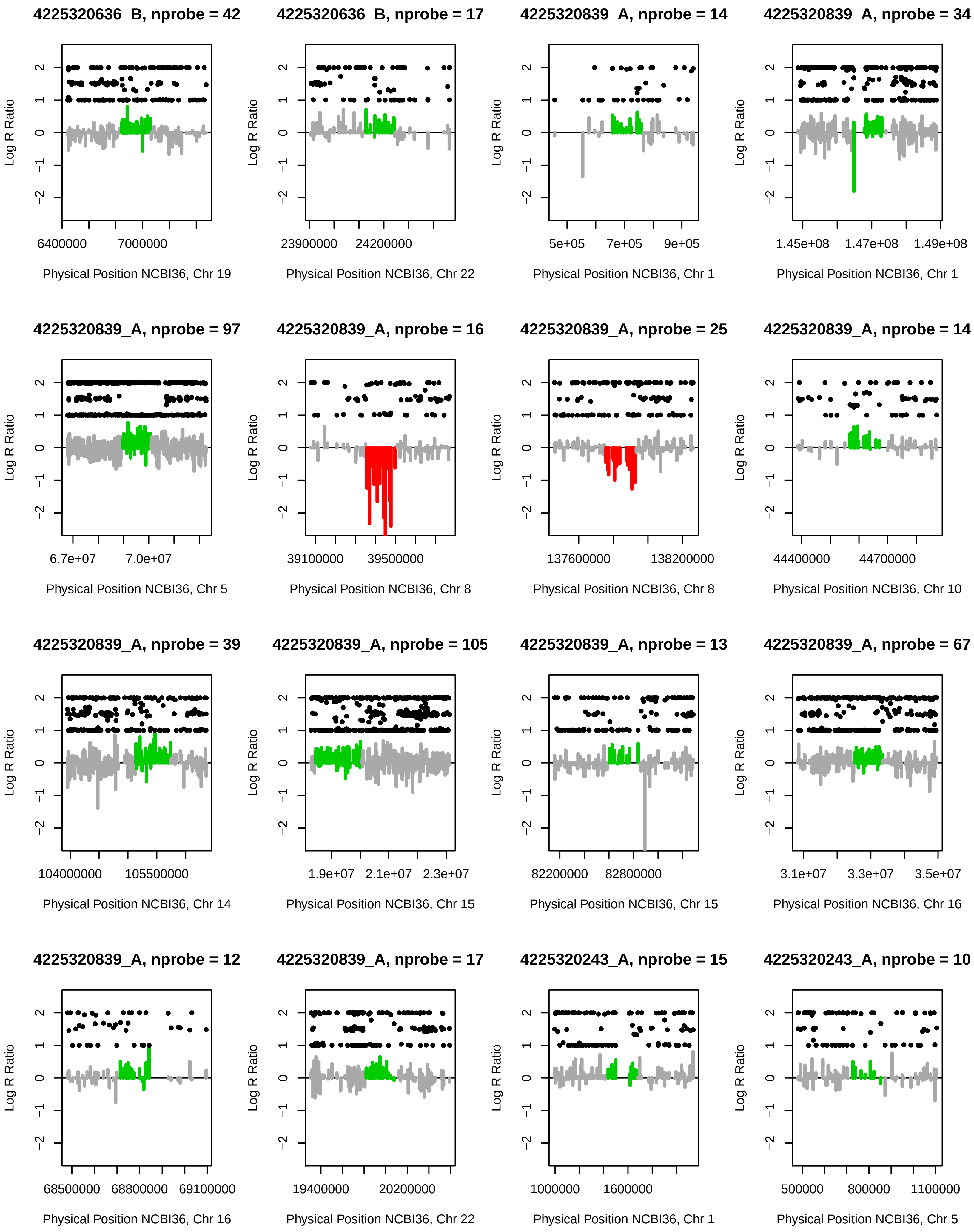
4225320636_B, nprobe = 42
Log R Ratio
Physical Position NCBI36, Chr 19
4225320636_B, nprobe = 17
Physical Position NCBI36, Chr 22
4225320839_A, nprobe = 14
Physical Position NCBI36, Chr 1
4225320839_A, nprobe = 34
Physical Position NCBI36, Chr 1
4225320839_A, nprobe = 97
Physical Position NCBI36, Chr 5
4225320839_A, nprobe = 16
Physical Position NCBI36, Chr 8
4225320839_A, nprobe = 25
Physical Position NCBI36, Chr 8
4225320839_A, nprobe = 14
Physical Position NCBI36, Chr 10
4225320839_A, nprobe = 39
Physical Position NCBI36, Chr 14
4225320839_A, nprobe = 105
Physical Position NCBI36, Chr 15
4225320839_A, nprobe = 13
Physical Position NCBI36, Chr 15
4225320839_A, nprobe = 67
Physical Position NCBI36, Chr 16
4225320839_A, nprobe = 12
Physical Position NCBI36, Chr 16
4225320839_A, nprobe = 17
Physical Position NCBI36, Chr 22
4225320243_A, nprobe = 15
Physical Position NCBI36, Chr 1
4225320243_A, nprobe = 10
Physical Position NCBI36, Chr 5

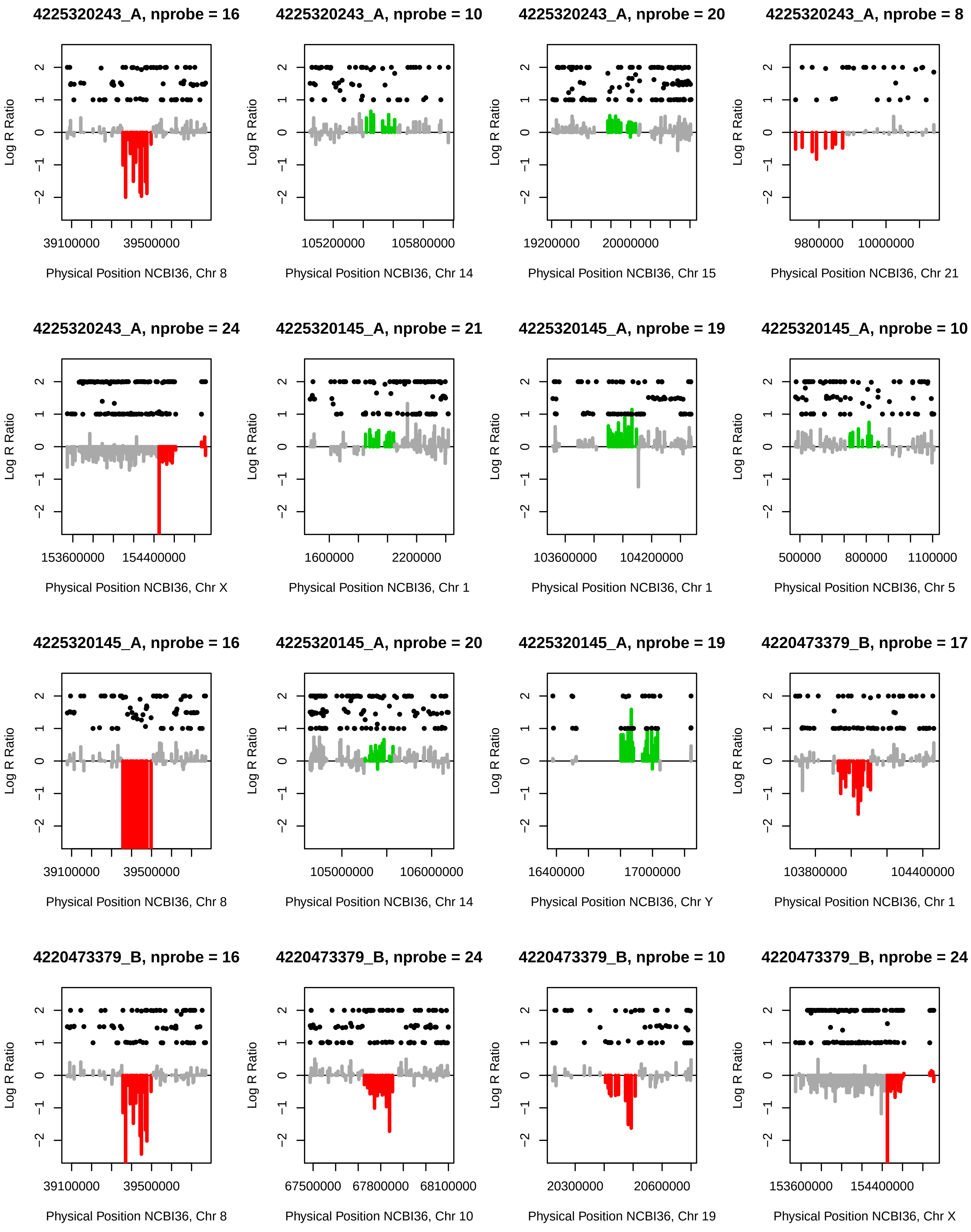
4225320243_A, nprobe = 16
Log R Ratio
39100000
39500000
Physical Position NCBI36, Chr 8
4225320243_A, nprobe = 10
Log R Ratio
105200000
105800000
Physical Position NCBI36, Chr 14
4225320243_A, nprobe = 20
Log R Ratio
19200000
20000000
Physical Position NCBI36, Chr 15
4225320243_A, nprobe = 8
Log R Ratio
9800000
10000000
Physical Position NCBI36, Chr 21
4225320243_A, nprobe = 24
Log R Ratio
153600000
154400000
Physical Position NCBI36, Chr X
4225320145_A, nprobe = 21
Log R Ratio
1600000
2200000
Physical Position NCBI36, Chr 1
4225320145_A, nprobe = 19
Log R Ratio
103600000
104200000
Physical Position NCBI36, Chr 1
4225320145_A, nprobe = 10
Log R Ratio
500000
800000
1100000
Physical Position NCBI36, Chr 5
4225320145_A, nprobe = 16
Log R Ratio
39100000
39500000
Physical Position NCBI36, Chr 8
4225320145_A, nprobe = 20
Log R Ratio
105000000
106000000
Physical Position NCBI36, Chr 14
4225320145_A, nprobe = 19
Log R Ratio
16400000
17000000
Physical Position NCBI36, Chr Y
4220473379_B, nprobe = 17
Log R Ratio
103800000
104400000
Physical Position NCBI36, Chr 1
4220473379_B, nprobe = 16
Log R Ratio
39100000
39500000
Physical Position NCBI36, Chr 8
4220473379_B, nprobe = 24
Log R Ratio
67500000
67800000
68100000
Physical Position NCBI36, Chr 10
4220473379_B, nprobe = 10
Log R Ratio
20300000
20600000
Physical Position NCBI36, Chr 19
4220473379_B, nprobe = 24
Log R Ratio
153600000
154400000
Physical Position NCBI36, Chr X

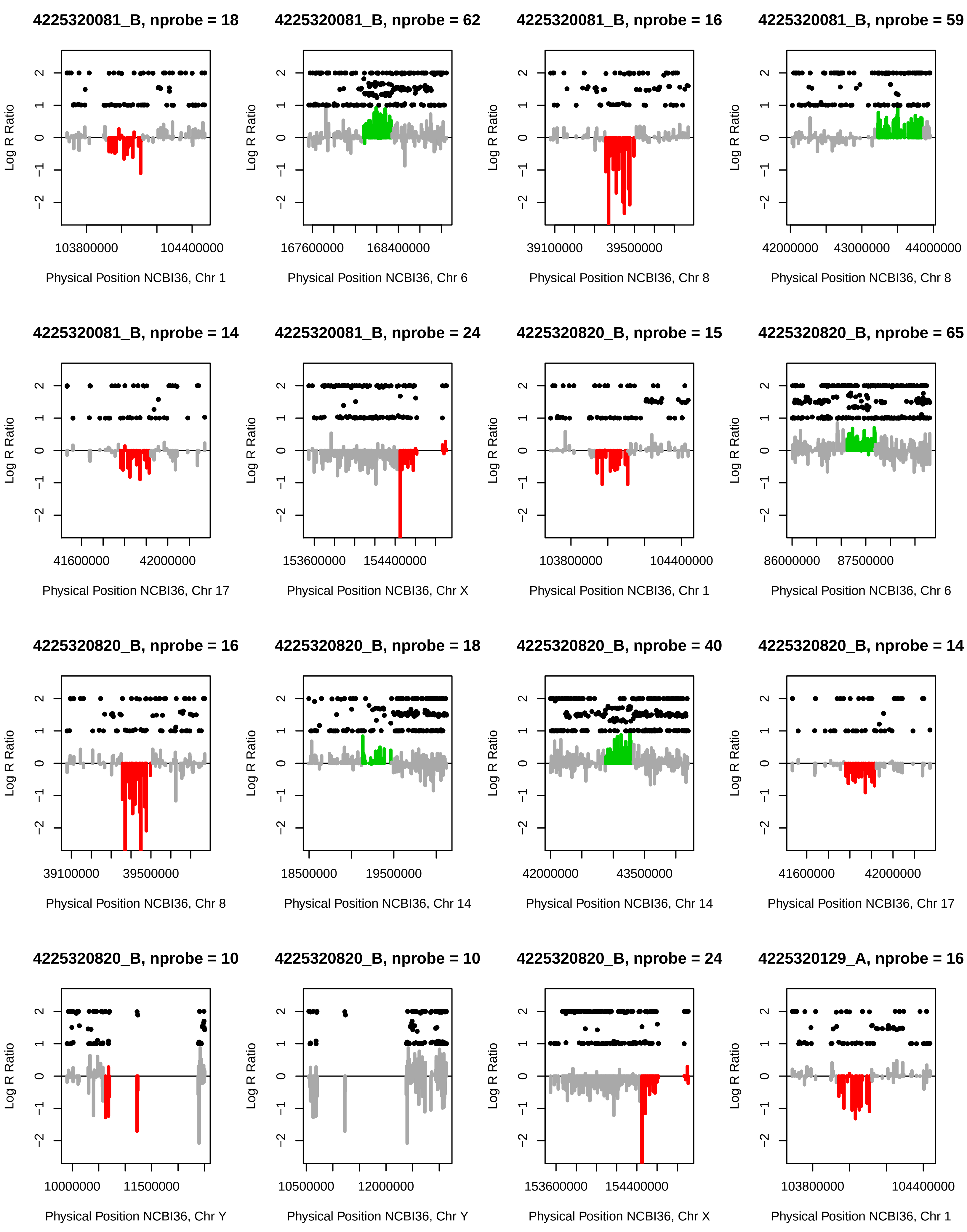
4225320081_B, nprobe = 18
Log R Ratio
Physical Position NCBI36, Chr 1
4225320081_B, nprobe = 62
Physical Position NCBI36, Chr 6
4225320081_B, nprobe = 16
Physical Position NCBI36, Chr 8
4225320081_B, nprobe = 59
Physical Position NCBI36, Chr 8
4225320081_B, nprobe = 14
Physical Position NCBI36, Chr 17
4225320081_B, nprobe = 24
Physical Position NCBI36, Chr X
4225320820_B, nprobe = 15
Physical Position NCBI36, Chr 1
4225320820_B, nprobe = 65
Physical Position NCBI36, Chr 6
4225320820_B, nprobe = 16
Physical Position NCBI36, Chr 8
4225320820_B, nprobe = 18
Physical Position NCBI36, Chr 14
4225320820_B, nprobe = 40
Physical Position NCBI36, Chr 14
4225320820_B, nprobe = 14
Physical Position NCBI36, Chr 17
4225320820_B, nprobe = 10
Physical Position NCBI36, Chr Y
4225320820_B, nprobe = 10
Physical Position NCBI36, Chr Y
4225320820_B, nprobe = 24
Physical Position NCBI36, Chr X
4225320129_A, nprobe = 16
Physical Position NCBI36, Chr 1

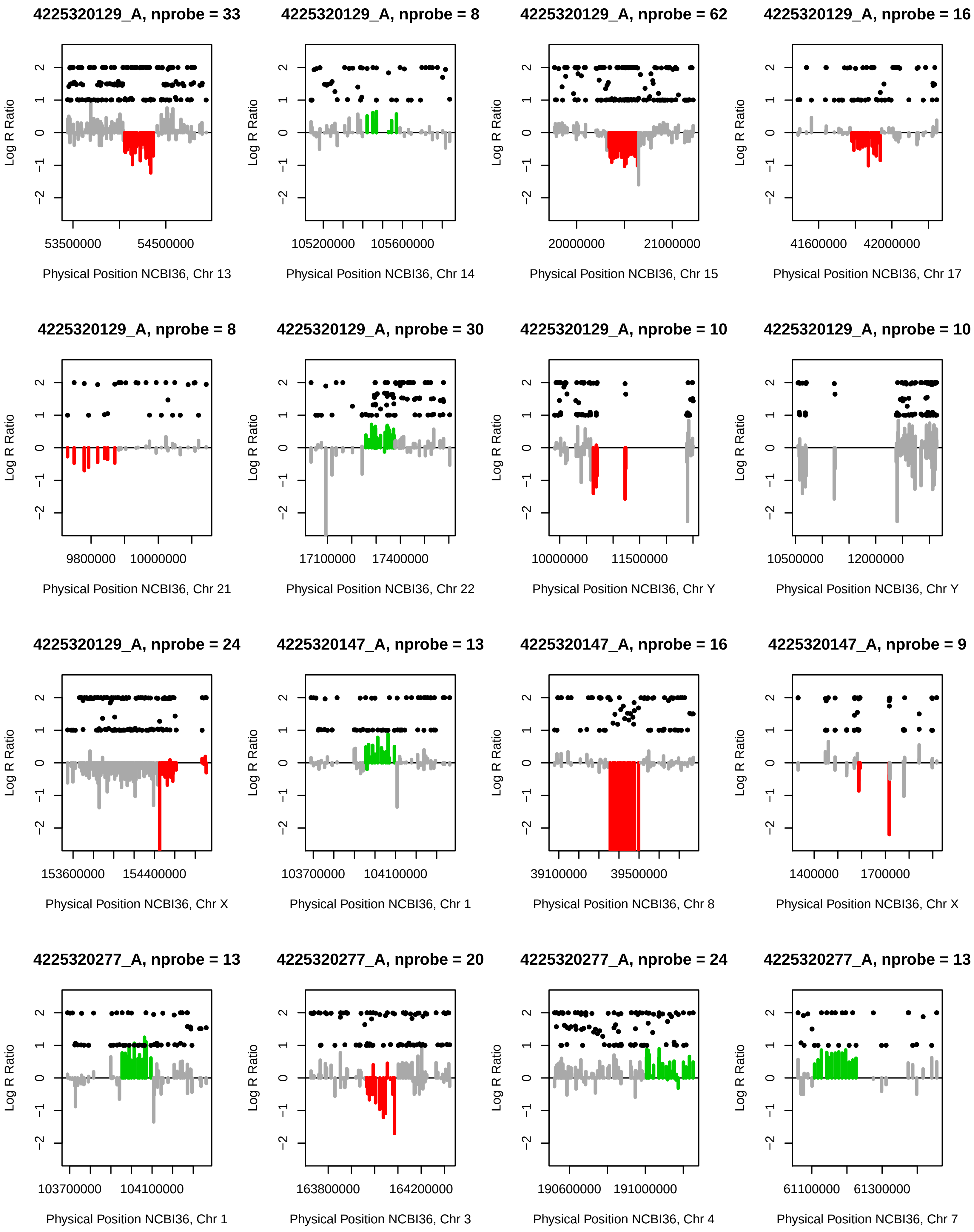

4225320129_A, nprobe = 33
Log R Ratio
-2
-1
0
1
2
53500000
54500000
Physical Position NCBI36, Chr 13
4225320129_A, nprobe = 8
Log R Ratio
105200000
105600000
Physical Position NCBI36, Chr 14
4225320129_A, nprobe = 62
Log R Ratio
20000000
21000000
Physical Position NCBI36, Chr 15
4225320129_A, nprobe = 16
Log R Ratio
41600000
42000000
Physical Position NCBI36, Chr 17
4225320129_A, nprobe = 8
Log R Ratio
9800000
10000000
Physical Position NCBI36, Chr 21
4225320129_A, nprobe = 30
Log R Ratio
17100000
17400000
Physical Position NCBI36, Chr 22
4225320129_A, nprobe = 10
Log R Ratio
10000000
11500000
Physical Position NCBI36, Chr Y
4225320129_A, nprobe = 10
Log R Ratio
10500000
12000000
Physical Position NCBI36, Chr Y
4225320129_A, nprobe = 24
Log R Ratio
153600000
154400000
Physical Position NCBI36, Chr X
4225320147_A, nprobe = 13
Log R Ratio
103700000
104100000
Physical Position NCBI36, Chr 1
4225320147_A, nprobe = 16
Log R Ratio
39100000
39500000
Physical Position NCBI36, Chr 8
4225320147_A, nprobe = 9
Log R Ratio
1400000
1700000
Physical Position NCBI36, Chr X
4225320277_A, nprobe = 13
Log R Ratio
103700000
104100000
Physical Position NCBI36, Chr 1
4225320277_A, nprobe = 20
Log R Ratio
163800000
164200000
Physical Position NCBI36, Chr 3
4225320277_A, nprobe = 24
Log R Ratio
190600000
191000000
Physical Position NCBI36, Chr 4
4225320277_A, nprobe = 13
Log R Ratio
61100000
61300000
Physical Position NCBI36, Chr 7

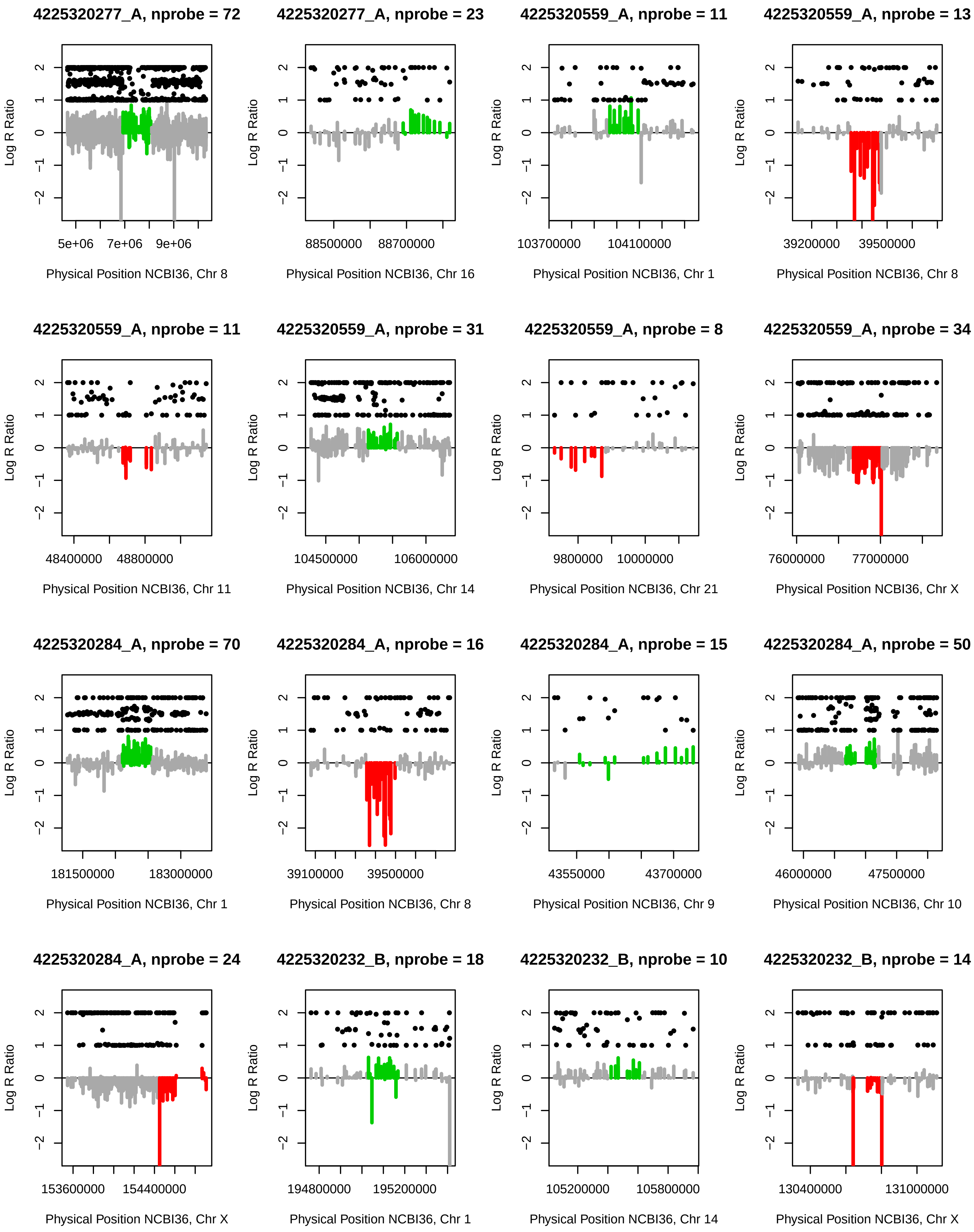
4225320277_A, nprobe = 72
Log R Ratio
−2
−1
0
1
2
5e+06
7e+06
9e+06
Physical Position NCBI36, Chr 8
4225320277_A, nprobe = 23
88500000
88700000
Physical Position NCBI36, Chr 16
4225320559_A, nprobe = 11
103700000
104100000
Physical Position NCBI36, Chr 1
4225320559_A, nprobe = 13
39200000
39500000
Physical Position NCBI36, Chr 8
4225320559_A, nprobe = 11
48400000
48800000
Physical Position NCBI36, Chr 11
4225320559_A, nprobe = 31
104500000
106000000
Physical Position NCBI36, Chr 14
4225320559_A, nprobe = 8
9800000
10000000
Physical Position NCBI36, Chr 21
4225320559_A, nprobe = 34
76000000
77000000
Physical Position NCBI36, Chr X
4225320284_A, nprobe = 70
181500000
183000000
Physical Position NCBI36, Chr 1
4225320284_A, nprobe = 16
39100000
39500000
Physical Position NCBI36, Chr 8
4225320284_A, nprobe = 15
43550000
43700000
Physical Position NCBI36, Chr 9
4225320284_A, nprobe = 50
46000000
47500000
Physical Position NCBI36, Chr 10
4225320284_A, nprobe = 24
153600000
154400000
Physical Position NCBI36, Chr X
4225320232_B, nprobe = 18
194800000
195200000
Physical Position NCBI36, Chr 1
4225320232_B, nprobe = 10
105200000
105800000
Physical Position NCBI36, Chr 14
4225320232_B, nprobe = 14
130400000
131000000
Physical Position NCBI36, Chr X

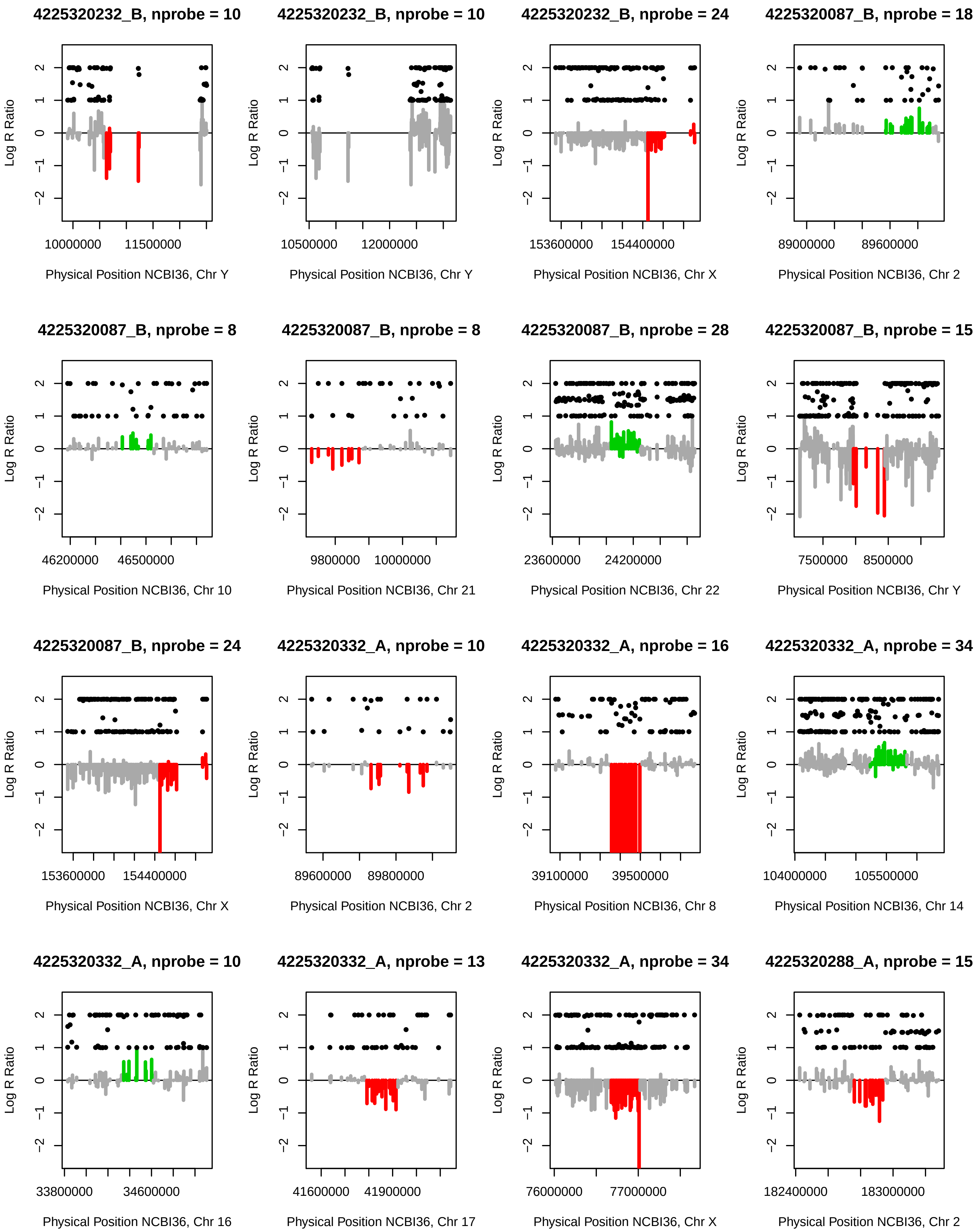
4225320232_B, nprobe = 10
Log R Ratio
Physical Position NCBI36, Chr Y
10000000
11500000
4225320232_B, nprobe = 10
Log R Ratio
Physical Position NCBI36, Chr Y
10500000
12000000
4225320232_B, nprobe = 24
Log R Ratio
Physical Position NCBI36, Chr X
153600000
154400000
4225320087_B, nprobe = 18
Log R Ratio
Physical Position NCBI36, Chr 2
89000000
89600000
4225320087_B, nprobe = 8
Log R Ratio
Physical Position NCBI36, Chr 10
46200000
46500000
4225320087_B, nprobe = 8
Log R Ratio
Physical Position NCBI36, Chr 21
9800000
10000000
4225320087_B, nprobe = 28
Log R Ratio
Physical Position NCBI36, Chr 22
23600000
24200000
4225320087_B, nprobe = 15
Log R Ratio
Physical Position NCBI36, Chr Y
7500000
8500000
4225320087_B, nprobe = 24
Log R Ratio
Physical Position NCBI36, Chr X
153600000
154400000
4225320332_A, nprobe = 10
Log R Ratio
Physical Position NCBI36, Chr 2
89600000
89800000
4225320332_A, nprobe = 16
Log R Ratio
Physical Position NCBI36, Chr 8
39100000
39500000
4225320332_A, nprobe = 34
Log R Ratio
Physical Position NCBI36, Chr 14
104000000
105500000
4225320332_A, nprobe = 10
Log R Ratio
Physical Position NCBI36, Chr 16
33800000
34600000
4225320332_A, nprobe = 13
Log R Ratio
Physical Position NCBI36, Chr 17
41600000
41900000
4225320332_A, nprobe = 34
Log R Ratio
Physical Position NCBI36, Chr X
76000000
77000000
4225320288_A, nprobe = 15
Log R Ratio
Physical Position NCBI36, Chr 2
182400000
183000000

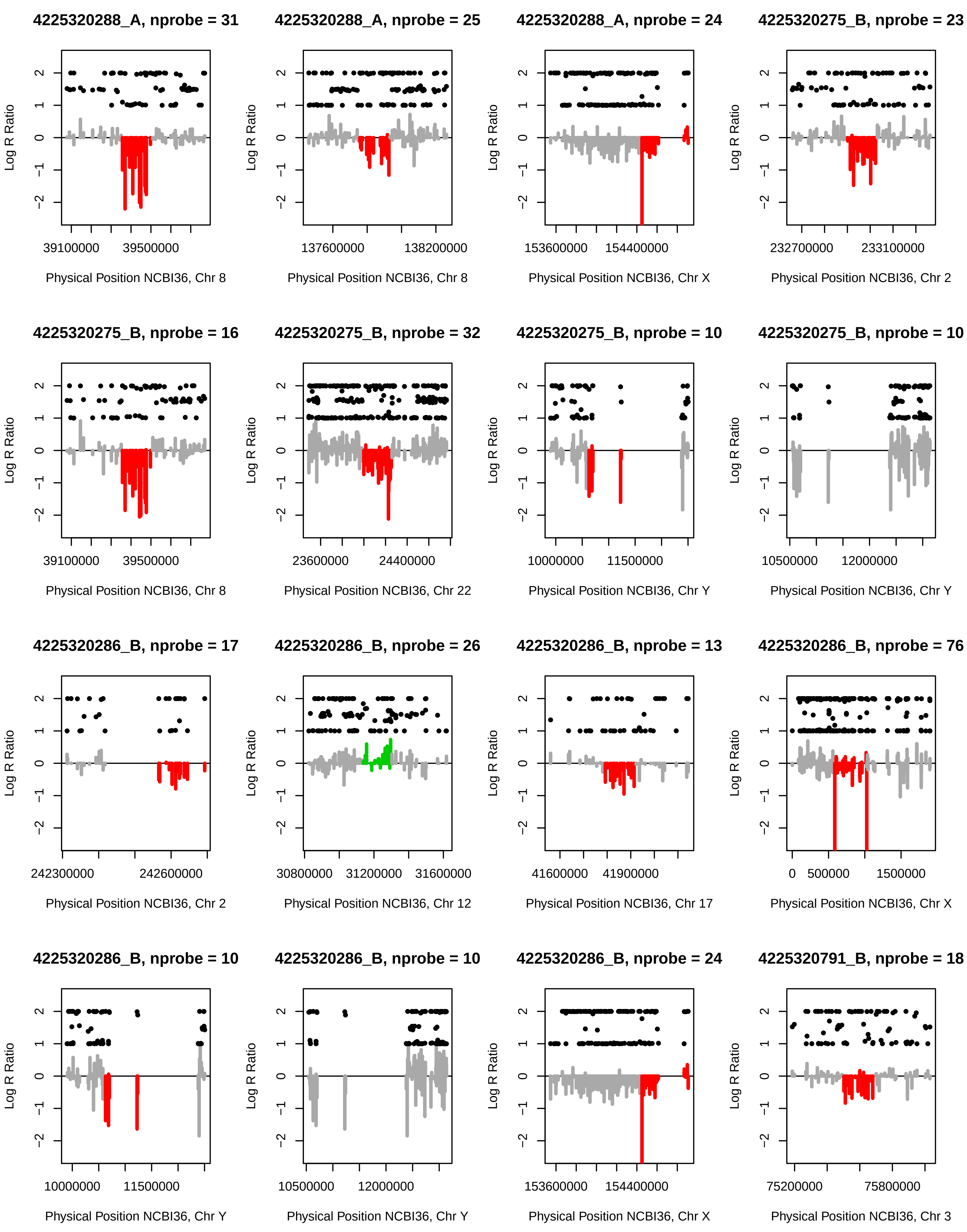

4225320288_A, nprobe = 31
Log R Ratio
-2 -1 0 1 2
39100000 39500000
Physical Position NCBI36, Chr 8
4225320288_A, nprobe = 25
Log R Ratio
-2 -1 0 1 2
137600000 138200000
Physical Position NCBI36, Chr 8
4225320288_A, nprobe = 24
Log R Ratio
-2 -1 0 1 2
153600000 154400000
Physical Position NCBI36, Chr X
4225320275_B, nprobe = 23
Log R Ratio
-2 -1 0 1 2
232700000 233100000
Physical Position NCBI36, Chr 2
4225320275_B, nprobe = 16
Log R Ratio
-2 -1 0 1 2
39100000 39500000
Physical Position NCBI36, Chr 8
4225320275_B, nprobe = 32
Log R Ratio
-2 -1 0 1 2
23600000 24400000
Physical Position NCBI36, Chr 22
4225320275_B, nprobe = 10
Log R Ratio
-2 -1 0 1 2
10000000 11500000
Physical Position NCBI36, Chr Y
4225320275_B, nprobe = 10
Log R Ratio
-2 -1 0 1 2
10500000 12000000
Physical Position NCBI36, Chr Y
4225320286_B, nprobe = 17
Log R Ratio
-2 -1 0 1 2
242300000 242600000
Physical Position NCBI36, Chr 2
4225320286_B, nprobe = 26
Log R Ratio
-2 -1 0 1 2
30800000 31200000 31600000
Physical Position NCBI36, Chr 12
4225320286_B, nprobe = 13
Log R Ratio
-2 -1 0 1 2
41600000 41900000
Physical Position NCBI36, Chr 17
4225320286_B, nprobe = 76
Log R Ratio
-2 -1 0 1 2
0 500000 1500000
Physical Position NCBI36, Chr X
4225320286_B, nprobe = 10
Log R Ratio
-2 -1 0 1 2
10000000 11500000
Physical Position NCBI36, Chr Y
4225320286_B, nprobe = 10
Log R Ratio
-2 -1 0 1 2
10500000 12000000
Physical Position NCBI36, Chr Y
4225320286_B, nprobe = 24
Log R Ratio
-2 -1 0 1 2
153600000 154400000
Physical Position NCBI36, Chr X
4225320791_B, nprobe = 18
Log R Ratio
-2 -1 0 1 2
75200000 75800000
Physical Position NCBI36, Chr 3

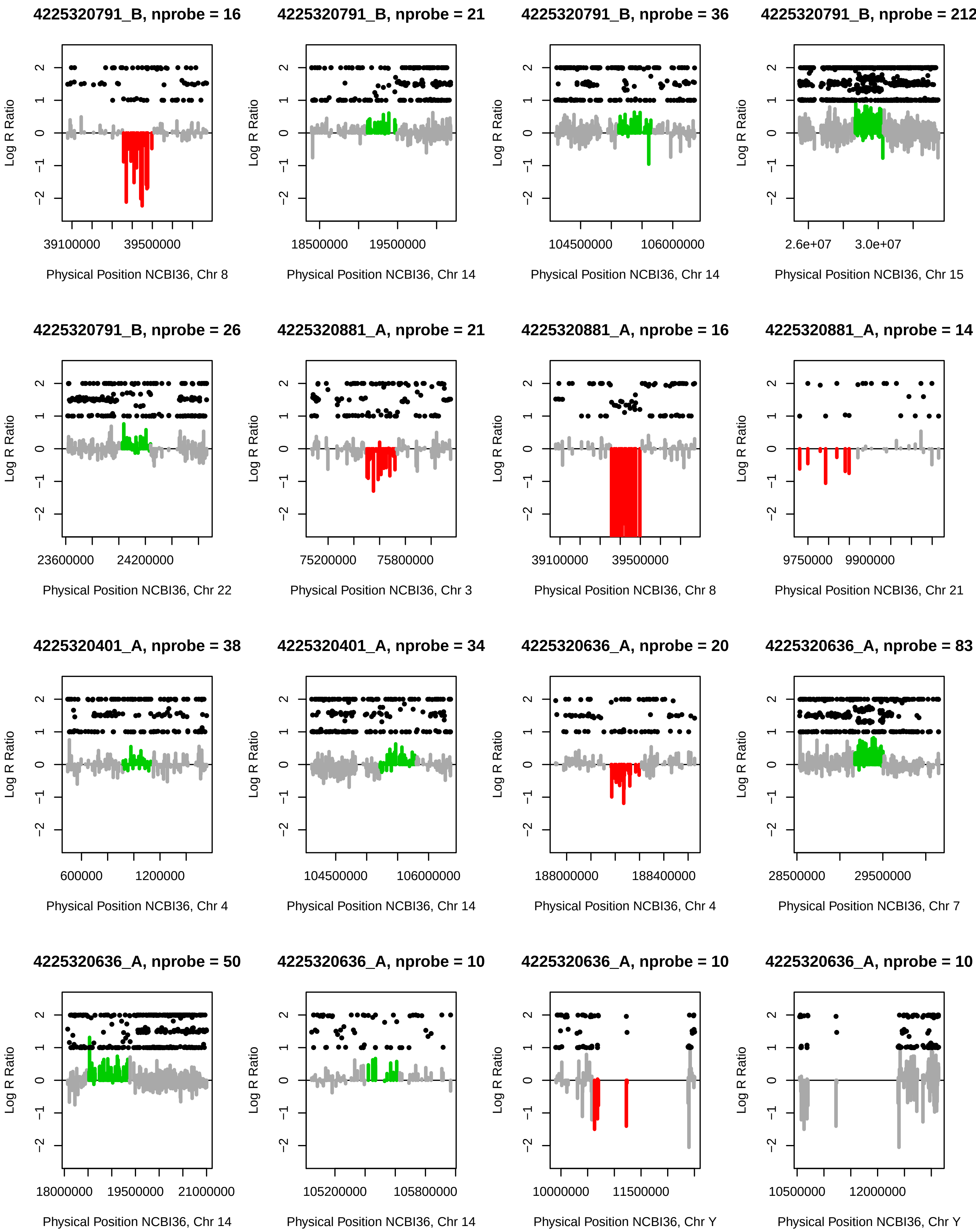

4225320791_B, nprobe = 16
Log R Ratio
Physical Position NCBI36, Chr 8
39100000
39500000
4225320791_B, nprobe = 21
Physical Position NCBI36, Chr 14
18500000
19500000
4225320791_B, nprobe = 36
Physical Position NCBI36, Chr 14
104500000
106000000
4225320791_B, nprobe = 212
Physical Position NCBI36, Chr 15
2.6e+07
3.0e+07
4225320791_B, nprobe = 26
Physical Position NCBI36, Chr 22
23600000
24200000
4225320881_A, nprobe = 21
Physical Position NCBI36, Chr 3
75200000
75800000
4225320881_A, nprobe = 16
Physical Position NCBI36, Chr 8
39100000
39500000
4225320881_A, nprobe = 14
Physical Position NCBI36, Chr 21
9750000
9900000
4225320401_A, nprobe = 38
Physical Position NCBI36, Chr 4
600000
1200000
4225320401_A, nprobe = 34
Physical Position NCBI36, Chr 14
104500000
106000000
4225320636_A, nprobe = 20
Physical Position NCBI36, Chr 4
188000000
188400000
4225320636_A, nprobe = 83
Physical Position NCBI36, Chr 7
28500000
29500000
4225320636_A, nprobe = 50
Physical Position NCBI36, Chr 14
18000000
19500000
21000000
4225320636_A, nprobe = 10
Physical Position NCBI36, Chr 14
105200000
105800000
4225320636_A, nprobe = 10
Physical Position NCBI36, Chr Y
10000000
11500000
4225320636_A, nprobe = 10
Physical Position NCBI36, Chr Y
10500000
12000000

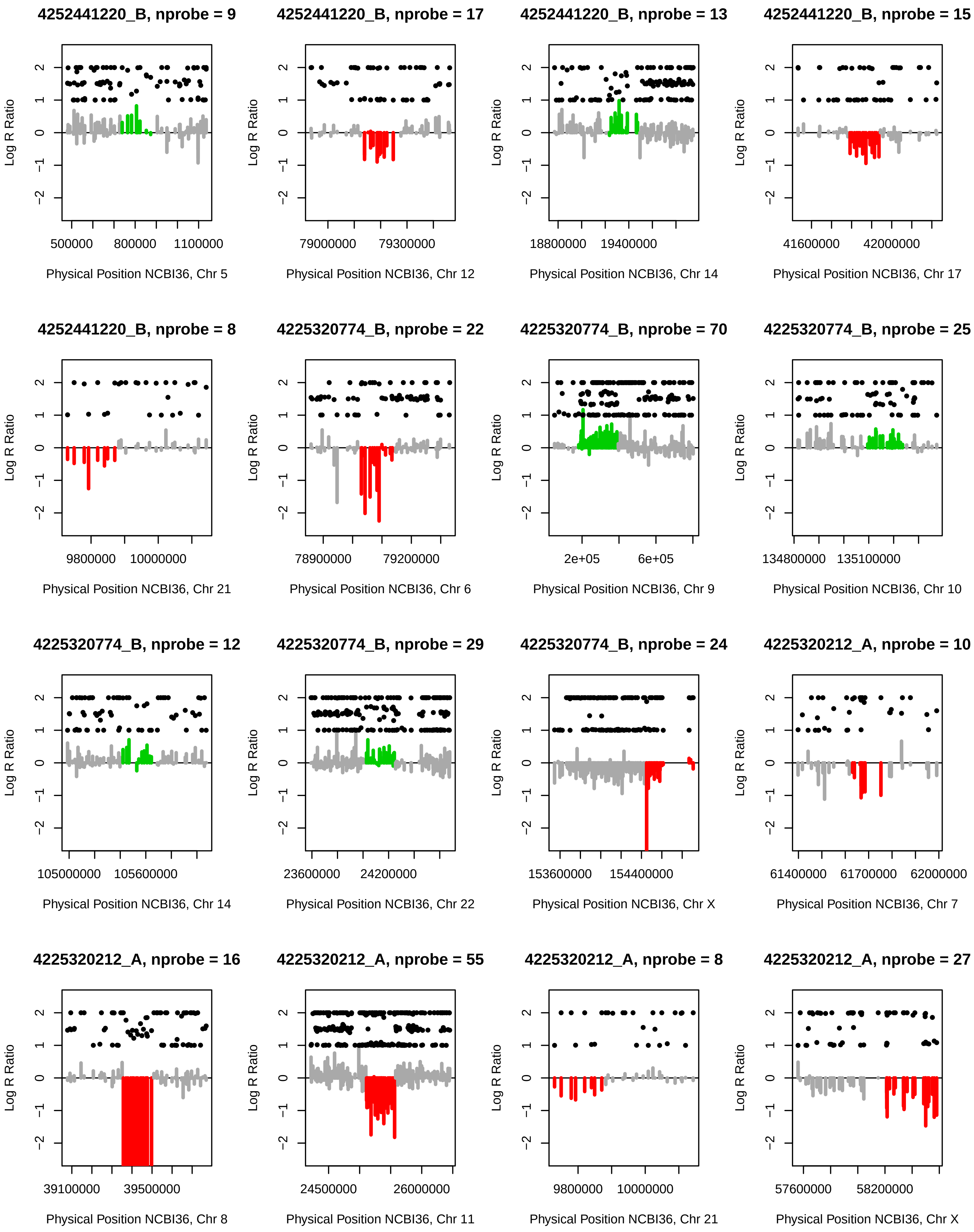
4252441220_B, nprobe = 9
Log R Ratio
Physical Position NCBI36, Chr 5
500000
800000
1100000
4252441220_B, nprobe = 17
Physical Position NCBI36, Chr 12
79000000
79300000
4252441220_B, nprobe = 13
Physical Position NCBI36, Chr 14
18800000
19400000
4252441220_B, nprobe = 15
Physical Position NCBI36, Chr 17
41600000
42000000
4252441220_B, nprobe = 8
Physical Position NCBI36, Chr 21
9800000
10000000
4225320774_B, nprobe = 22
Physical Position NCBI36, Chr 6
78900000
79200000
4225320774_B, nprobe = 70
Physical Position NCBI36, Chr 9
2e+05
6e+05
4225320774_B, nprobe = 25
Physical Position NCBI36, Chr 10
134800000
135100000
4225320774_B, nprobe = 12
Physical Position NCBI36, Chr 14
105000000
105600000
4225320774_B, nprobe = 29
Physical Position NCBI36, Chr 22
23600000
24200000
4225320774_B, nprobe = 24
Physical Position NCBI36, Chr X
153600000
154400000
4225320212_A, nprobe = 10
Physical Position NCBI36, Chr 7
61400000
61700000
62000000
4225320212_A, nprobe = 16
Physical Position NCBI36, Chr 8
39100000
39500000
4225320212_A, nprobe = 55
Physical Position NCBI36, Chr 11
24500000
26000000
4225320212_A, nprobe = 8
Physical Position NCBI36, Chr 21
9800000
10000000
4225320212_A, nprobe = 27
Physical Position NCBI36, Chr X
57600000
58200000

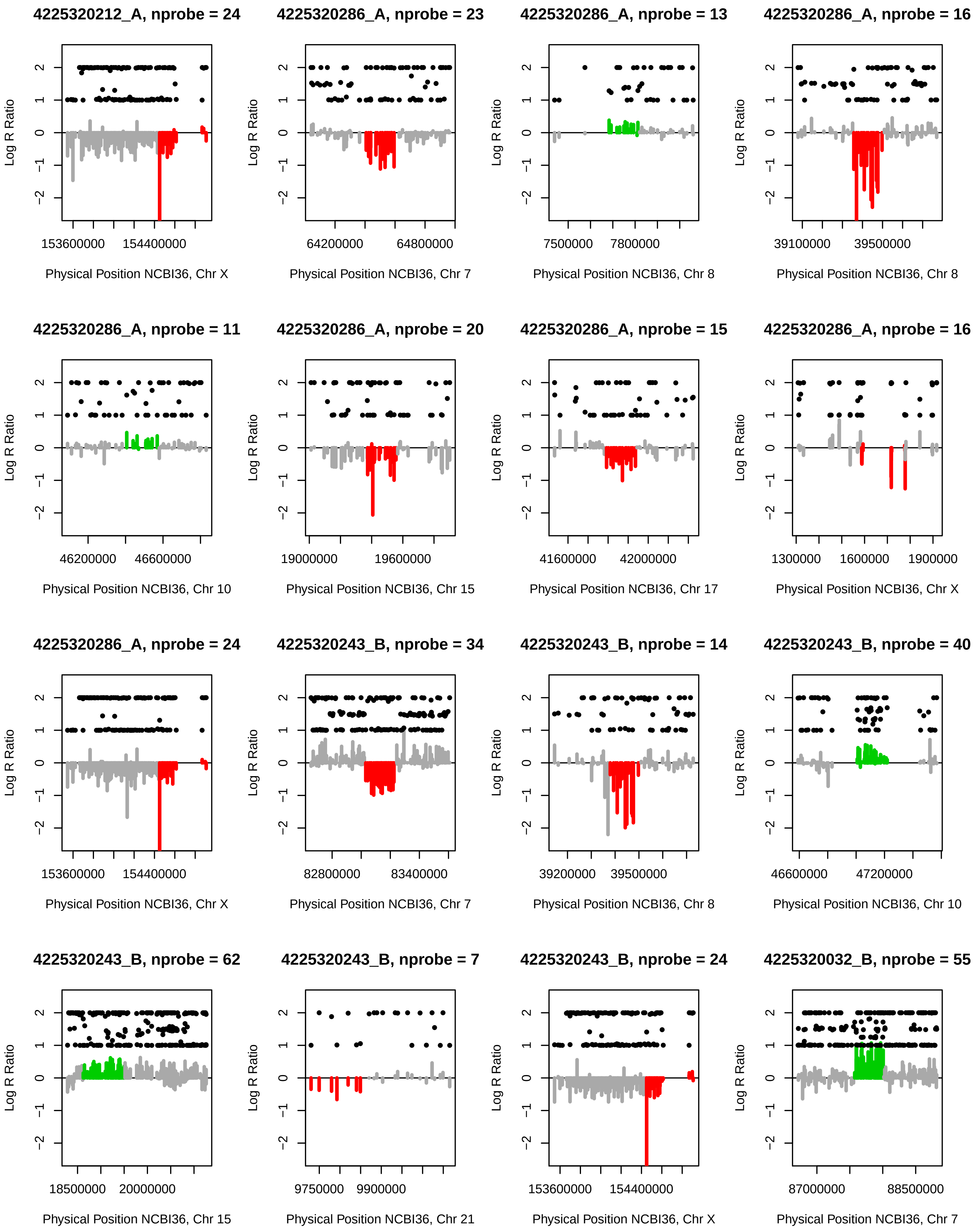
4225320212_A, nprobe = 24
Log R Ratio
153600000
154400000
Physical Position NCBI36, Chr X
4225320286_A, nprobe = 23
Log R Ratio
64200000
64800000
Physical Position NCBI36, Chr 7
4225320286_A, nprobe = 13
Log R Ratio
7500000
7800000
Physical Position NCBI36, Chr 8
4225320286_A, nprobe = 16
Log R Ratio
39100000
39500000
Physical Position NCBI36, Chr 8
4225320286_A, nprobe = 11
Log R Ratio
46200000
46600000
Physical Position NCBI36, Chr 10
4225320286_A, nprobe = 20
Log R Ratio
19000000
19600000
Physical Position NCBI36, Chr 15
4225320286_A, nprobe = 15
Log R Ratio
41600000
42000000
Physical Position NCBI36, Chr 17
4225320286_A, nprobe = 16
Log R Ratio
1300000
1600000
1900000
Physical Position NCBI36, Chr X
4225320286_A, nprobe = 24
Log R Ratio
153600000
154400000
Physical Position NCBI36, Chr X
4225320243_B, nprobe = 34
Log R Ratio
82800000
83400000
Physical Position NCBI36, Chr 7
4225320243_B, nprobe = 14
Log R Ratio
39200000
39500000
Physical Position NCBI36, Chr 8
4225320243_B, nprobe = 40
Log R Ratio
46600000
47200000
Physical Position NCBI36, Chr 10
4225320243_B, nprobe = 62
Log R Ratio
18500000
20000000
Physical Position NCBI36, Chr 15
4225320243_B, nprobe = 7
Log R Ratio
9750000
9900000
Physical Position NCBI36, Chr 21
4225320243_B, nprobe = 24
Log R Ratio
153600000
154400000
Physical Position NCBI36, Chr X
4225320032_B, nprobe = 55
Log R Ratio
87000000
88500000
Physical Position NCBI36, Chr 7

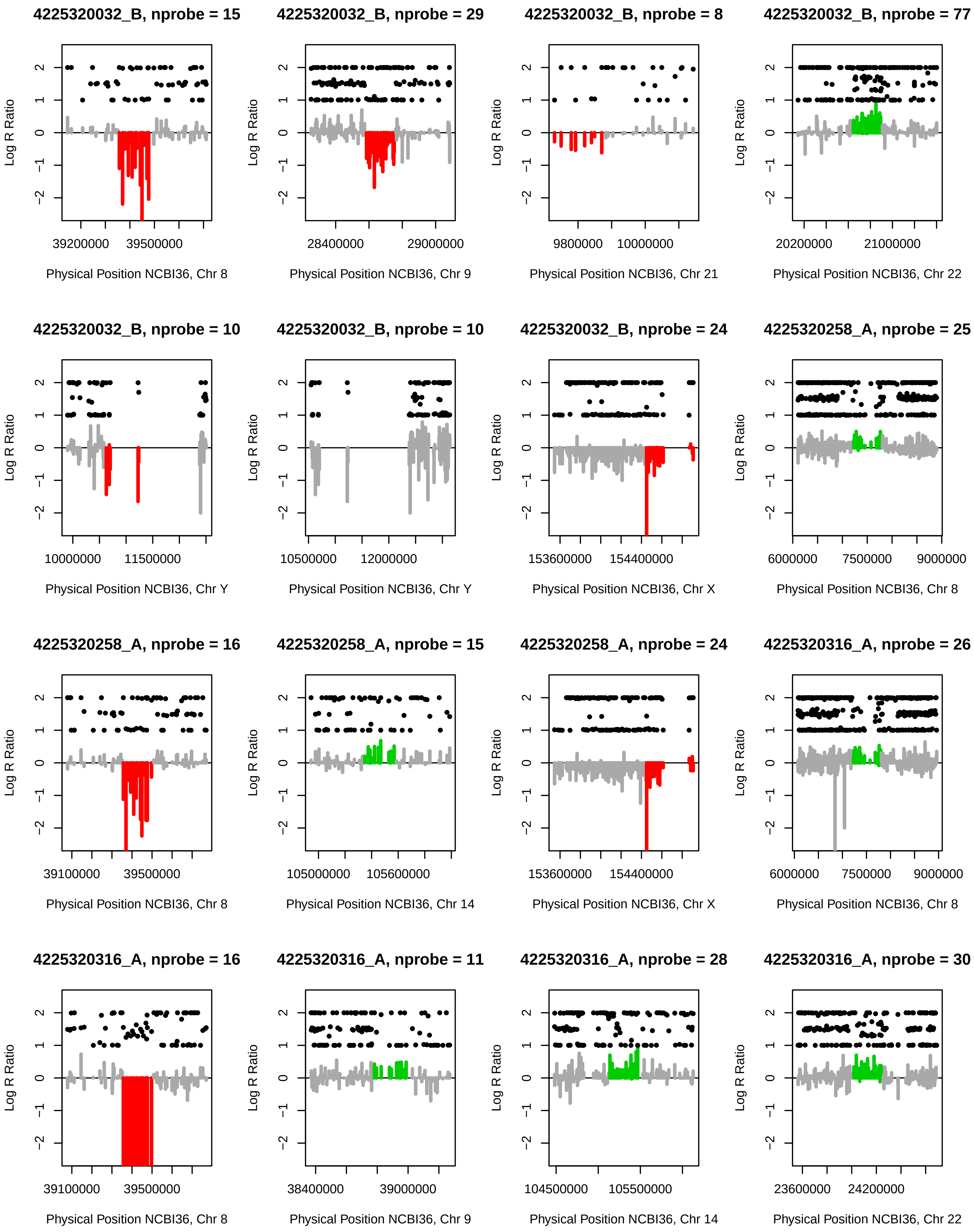
4225320032_B, nprobe = 15
Log R Ratio
39200000
39500000
Physical Position NCBI36, Chr 8
4225320032_B, nprobe = 29
28400000
29000000
Physical Position NCBI36, Chr 9
4225320032_B, nprobe = 8
9800000
10000000
Physical Position NCBI36, Chr 21
4225320032_B, nprobe = 77
20200000
21000000
Physical Position NCBI36, Chr 22
4225320032_B, nprobe = 10
10000000
11500000
Physical Position NCBI36, Chr Y
4225320032_B, nprobe = 10
10500000
12000000
Physical Position NCBI36, Chr Y
4225320032_B, nprobe = 24
153600000
154400000
Physical Position NCBI36, Chr X
4225320258_A, nprobe = 25
6000000
7500000
9000000
Physical Position NCBI36, Chr 8
4225320258_A, nprobe = 16
39100000
39500000
Physical Position NCBI36, Chr 8
4225320258_A, nprobe = 15
105000000
105600000
Physical Position NCBI36, Chr 14
4225320258_A, nprobe = 24
153600000
154400000
Physical Position NCBI36, Chr X
4225320316_A, nprobe = 26
6000000
7500000
9000000
Physical Position NCBI36, Chr 8
4225320316_A, nprobe = 16
39100000
39500000
Physical Position NCBI36, Chr 8
4225320316_A, nprobe = 11
38400000
39000000
Physical Position NCBI36, Chr 9
4225320316_A, nprobe = 28
104500000
105500000
Physical Position NCBI36, Chr 14
4225320316_A, nprobe = 30
23600000
24200000
Physical Position NCBI36, Chr 22

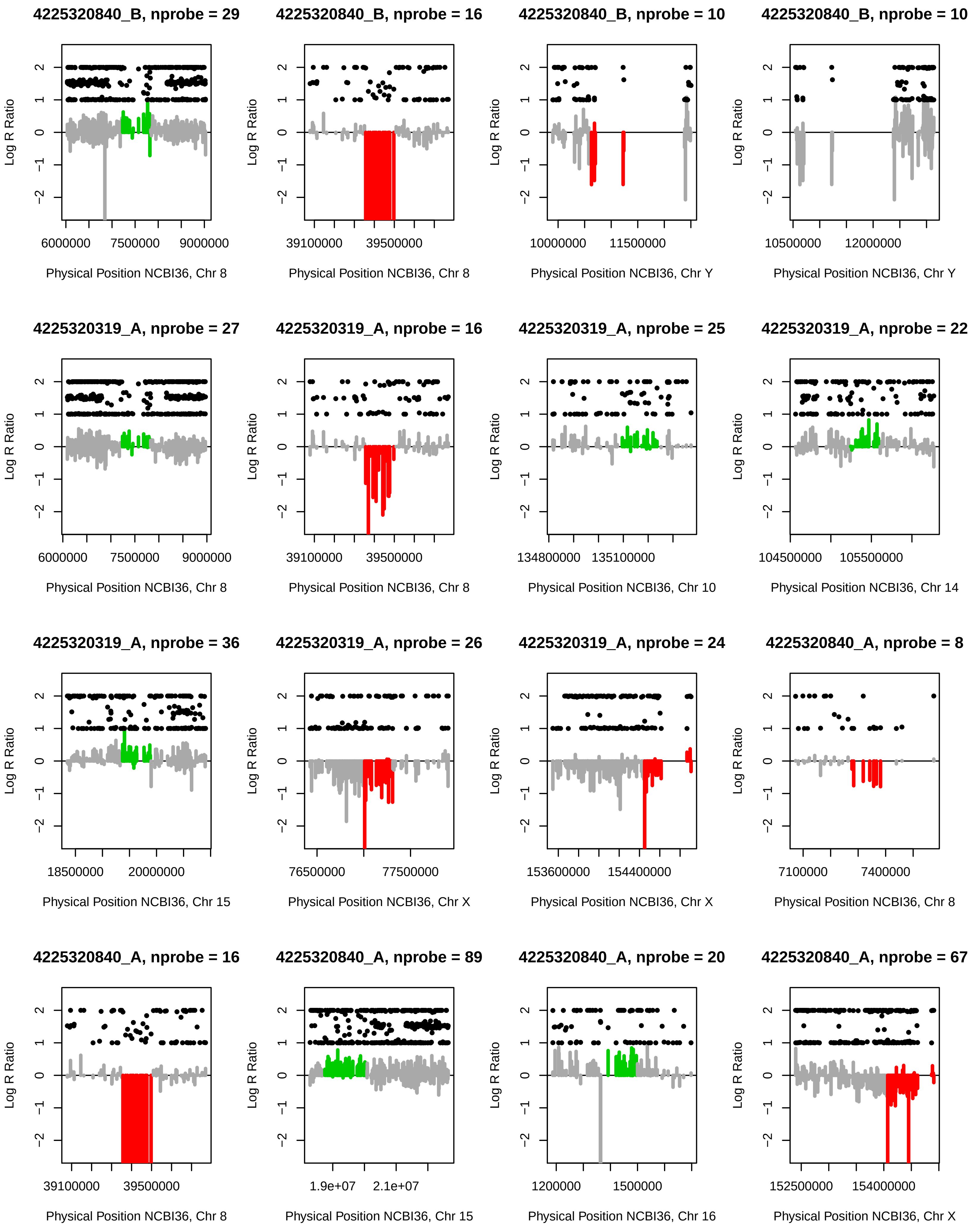
4225320840_B, nprobe = 29
Log R Ratio
6000000
7500000
9000000
Physical Position NCBI36, Chr 8
4225320840_B, nprobe = 16
Log R Ratio
39100000
39500000
Physical Position NCBI36, Chr 8
4225320840_B, nprobe = 10
Log R Ratio
10000000
11500000
Physical Position NCBI36, Chr Y
4225320840_B, nprobe = 10
Log R Ratio
10500000
12000000
Physical Position NCBI36, Chr Y
4225320319_A, nprobe = 27
Log R Ratio
6000000
7500000
9000000
Physical Position NCBI36, Chr 8
4225320319_A, nprobe = 16
Log R Ratio
39100000
39500000
Physical Position NCBI36, Chr 8
4225320319_A, nprobe = 25
Log R Ratio
134800000
135100000
Physical Position NCBI36, Chr 10
4225320319_A, nprobe = 22
Log R Ratio
104500000
105500000
Physical Position NCBI36, Chr 14
4225320319_A, nprobe = 36
Log R Ratio
18500000
20000000
Physical Position NCBI36, Chr 15
4225320319_A, nprobe = 26
Log R Ratio
76500000
77500000
Physical Position NCBI36, Chr X
4225320319_A, nprobe = 24
Log R Ratio
153600000
154400000
Physical Position NCBI36, Chr X
4225320840_A, nprobe = 8
Log R Ratio
7100000
7400000
Physical Position NCBI36, Chr 8
4225320840_A, nprobe = 16
Log R Ratio
39100000
39500000
Physical Position NCBI36, Chr 8
4225320840_A, nprobe = 89
Log R Ratio
1.9e+07
2.1e+07
Physical Position NCBI36, Chr 15
4225320840_A, nprobe = 20
Log R Ratio
1200000
1500000
Physical Position NCBI36, Chr 16
4225320840_A, nprobe = 67
Log R Ratio
152500000
154000000
Physical Position NCBI36, Chr X

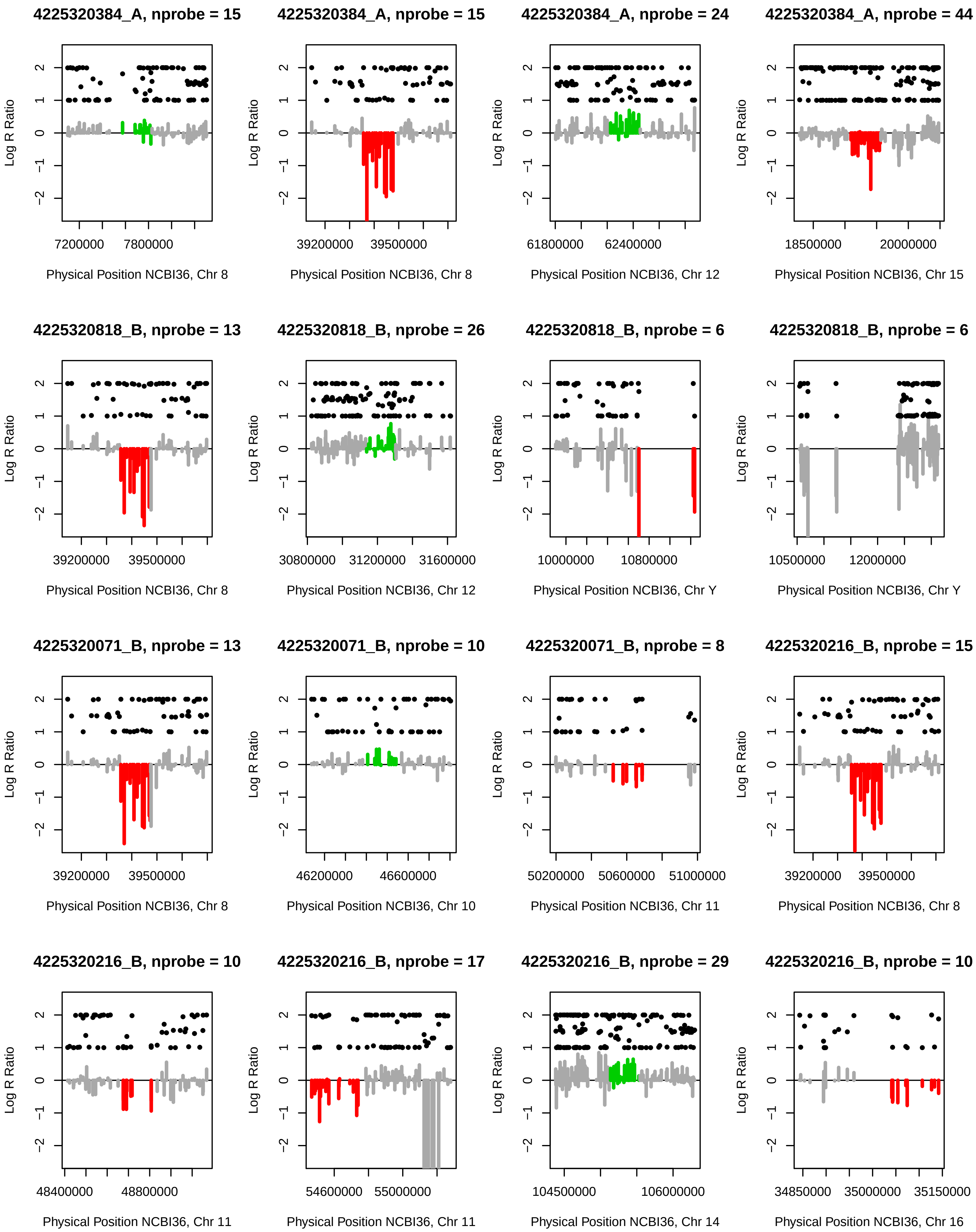
4225320384_A, nprobe = 15
Log R Ratio
7200000
7800000
Physical Position NCBI36, Chr 8
4225320384_A, nprobe = 15
Log R Ratio
39200000
39500000
Physical Position NCBI36, Chr 8
4225320384_A, nprobe = 24
Log R Ratio
61800000
62400000
Physical Position NCBI36, Chr 12
4225320384_A, nprobe = 44
Log R Ratio
18500000
20000000
Physical Position NCBI36, Chr 15
4225320818_B, nprobe = 13
Log R Ratio
39200000
39500000
Physical Position NCBI36, Chr 8
4225320818_B, nprobe = 26
Log R Ratio
30800000
31200000
31600000
Physical Position NCBI36, Chr 12
4225320818_B, nprobe = 6
Log R Ratio
10000000
10800000
Physical Position NCBI36, Chr Y
4225320818_B, nprobe = 6
Log R Ratio
10500000
12000000
Physical Position NCBI36, Chr Y
4225320071_B, nprobe = 13
Log R Ratio
39200000
39500000
Physical Position NCBI36, Chr 8
4225320071_B, nprobe = 10
Log R Ratio
46200000
46600000
Physical Position NCBI36, Chr 10
4225320071_B, nprobe = 8
Log R Ratio
50200000
50600000
51000000
Physical Position NCBI36, Chr 11
4225320216_B, nprobe = 15
Log R Ratio
39200000
39500000
Physical Position NCBI36, Chr 8
4225320216_B, nprobe = 10
Log R Ratio
48400000
48800000
Physical Position NCBI36, Chr 11
4225320216_B, nprobe = 17
Log R Ratio
54600000
55000000
Physical Position NCBI36, Chr 11
4225320216_B, nprobe = 29
Log R Ratio
104500000
106000000
Physical Position NCBI36, Chr 14
4225320216_B, nprobe = 10
Log R Ratio
34850000
35000000
35150000
Physical Position NCBI36, Chr 16

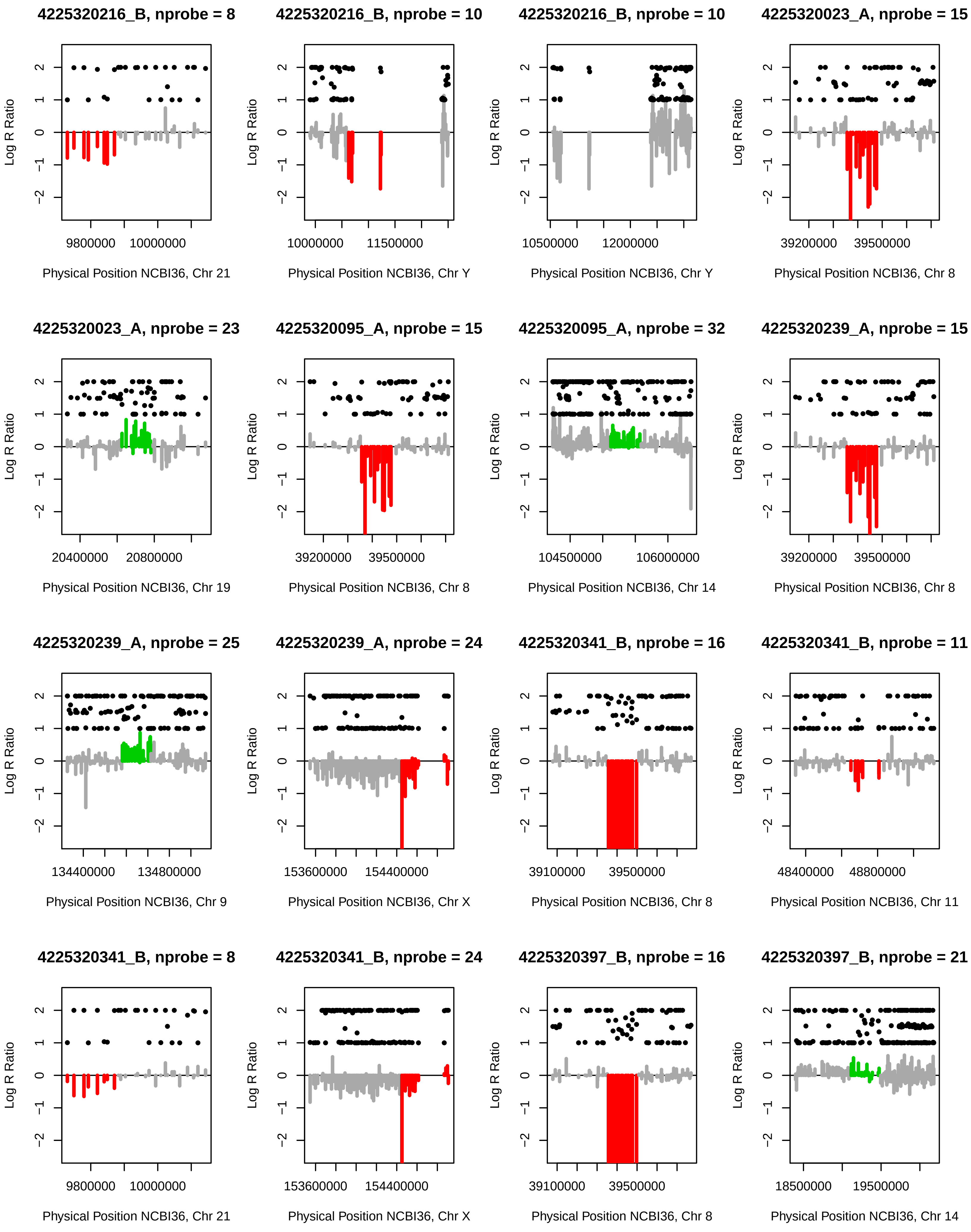

4225320216_B, nprobe = 8
Log R Ratio
2
1
0
−1
−2
9800000
10000000
Physical Position NCBI36, Chr 21
4225320216_B, nprobe = 10
10000000
11500000
Physical Position NCBI36, Chr Y
4225320216_B, nprobe = 10
10500000
12000000
Physical Position NCBI36, Chr Y
4225320023_A, nprobe = 15
39200000
39500000
Physical Position NCBI36, Chr 8
4225320023_A, nprobe = 23
20400000
20800000
Physical Position NCBI36, Chr 19
4225320095_A, nprobe = 15
39200000
39500000
Physical Position NCBI36, Chr 8
4225320095_A, nprobe = 32
104500000
106000000
Physical Position NCBI36, Chr 14
4225320239_A, nprobe = 15
39200000
39500000
Physical Position NCBI36, Chr 8
4225320239_A, nprobe = 25
134400000
134800000
Physical Position NCBI36, Chr 9
4225320239_A, nprobe = 24
153600000
154400000
Physical Position NCBI36, Chr X
4225320341_B, nprobe = 16
39100000
39500000
Physical Position NCBI36, Chr 8
4225320341_B, nprobe = 11
48400000
48800000
Physical Position NCBI36, Chr 11
4225320341_B, nprobe = 8
9800000
10000000
Physical Position NCBI36, Chr 21
4225320341_B, nprobe = 24
153600000
154400000
Physical Position NCBI36, Chr X
4225320397_B, nprobe = 16
39100000
39500000
Physical Position NCBI36, Chr 8
4225320397_B, nprobe = 21
18500000
19500000
Physical Position NCBI36, Chr 14

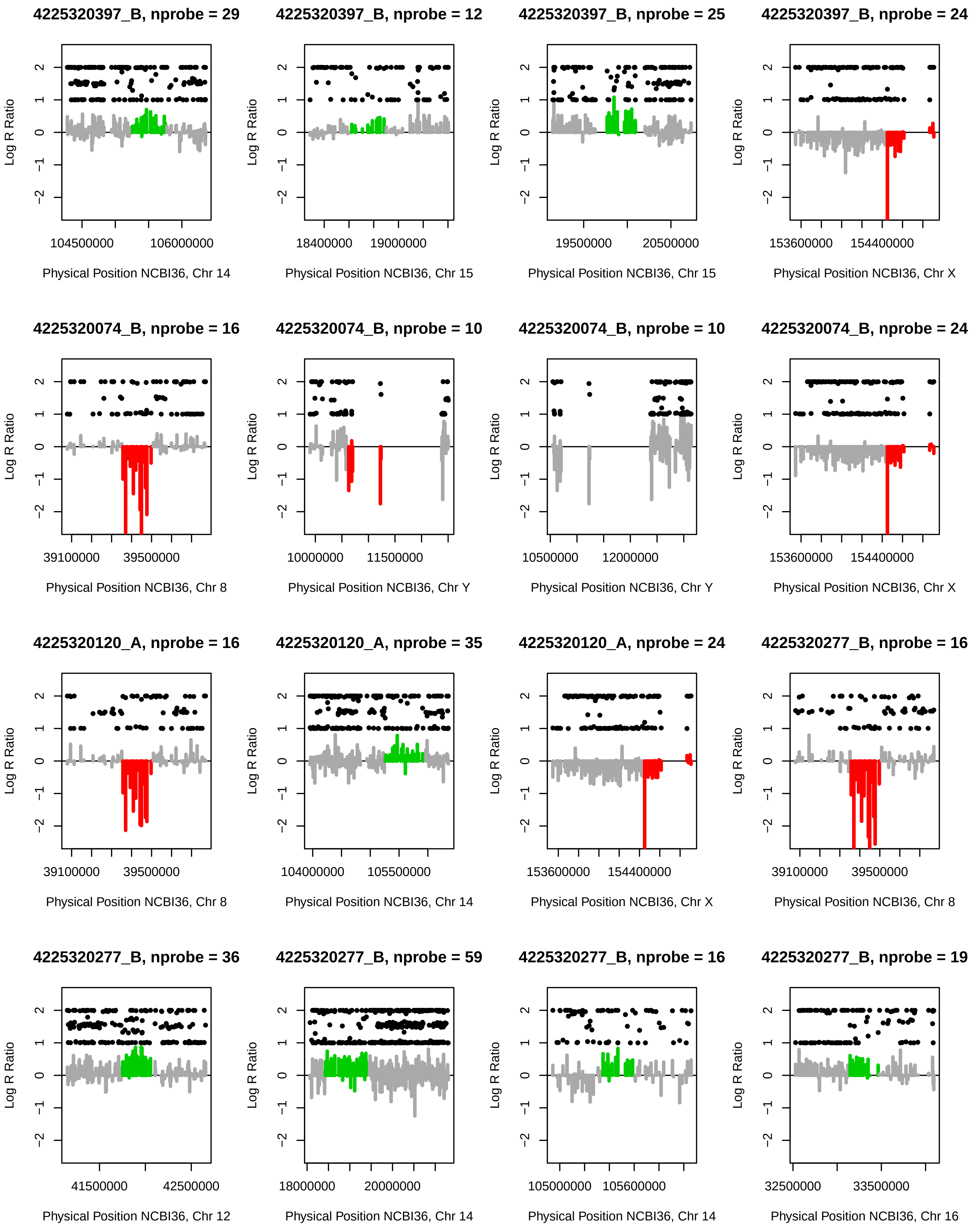

4225320397_B, nprobe = 29
Log R Ratio
104500000
106000000
Physical Position NCBI36, Chr 14
4225320397_B, nprobe = 12
18400000
19000000
Physical Position NCBI36, Chr 15
4225320397_B, nprobe = 25
19500000
20500000
Physical Position NCBI36, Chr 15
4225320397_B, nprobe = 24
153600000
154400000
Physical Position NCBI36, Chr X
4225320074_B, nprobe = 16
39100000
39500000
Physical Position NCBI36, Chr 8
4225320074_B, nprobe = 10
10000000
11500000
Physical Position NCBI36, Chr Y
4225320074_B, nprobe = 10
10500000
12000000
Physical Position NCBI36, Chr Y
4225320074_B, nprobe = 24
153600000
154400000
Physical Position NCBI36, Chr X
4225320120_A, nprobe = 16
39100000
39500000
Physical Position NCBI36, Chr 8
4225320120_A, nprobe = 35
104000000
105500000
Physical Position NCBI36, Chr 14
4225320120_A, nprobe = 24
153600000
154400000
Physical Position NCBI36, Chr X
4225320277_B, nprobe = 16
39100000
39500000
Physical Position NCBI36, Chr 8
4225320277_B, nprobe = 36
41500000
42500000
Physical Position NCBI36, Chr 12
4225320277_B, nprobe = 59
18000000
20000000
Physical Position NCBI36, Chr 14
4225320277_B, nprobe = 16
105000000
105600000
Physical Position NCBI36, Chr 14
4225320277_B, nprobe = 19
32500000
33500000
Physical Position NCBI36, Chr 16

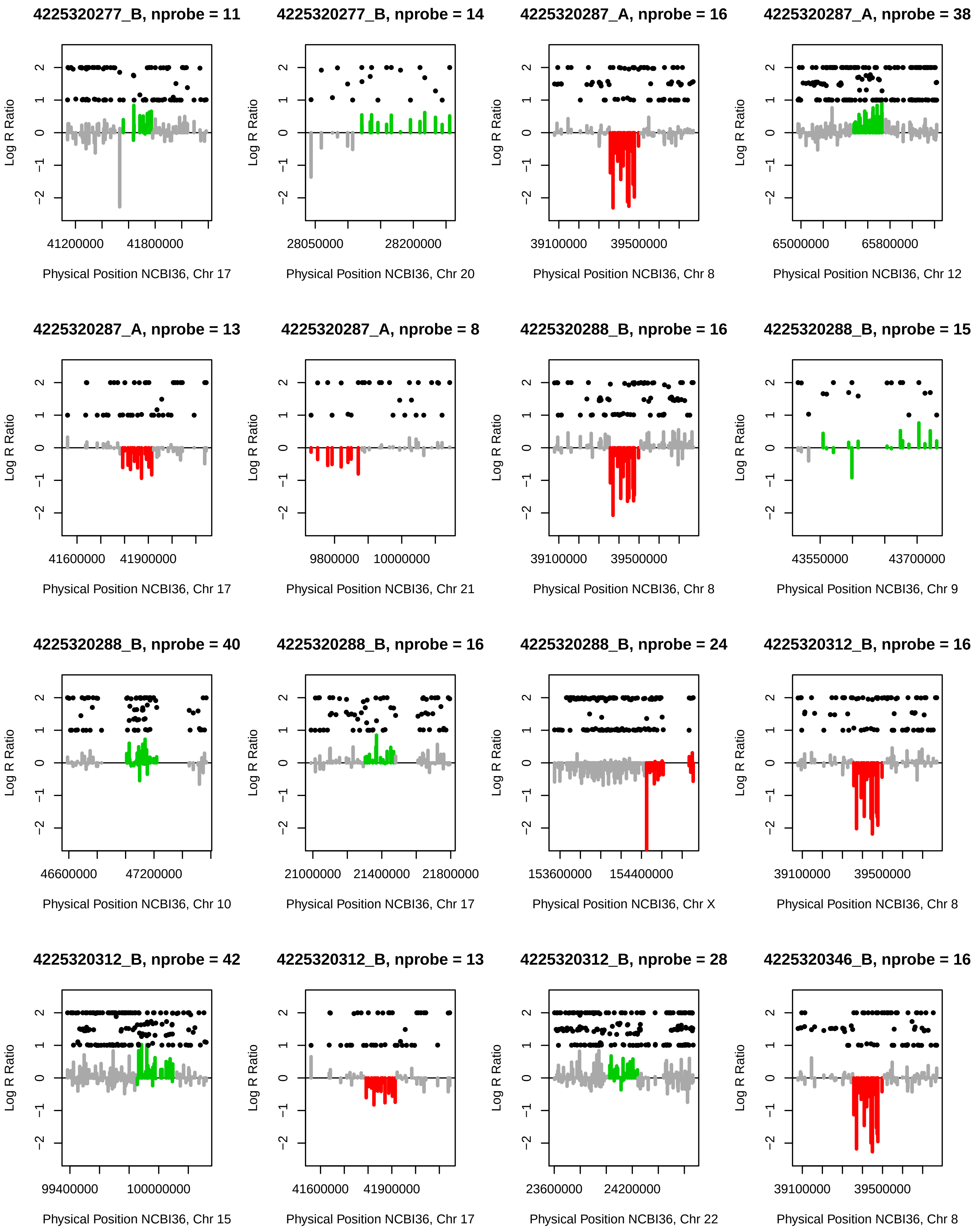
4225320277_B, nprobe = 11
Log R Ratio
41200000
41800000
Physical Position NCBI36, Chr 17
4225320277_B, nprobe = 14
Log R Ratio
28050000
28200000
Physical Position NCBI36, Chr 20
4225320287_A, nprobe = 16
Log R Ratio
39100000
39500000
Physical Position NCBI36, Chr 8
4225320287_A, nprobe = 38
Log R Ratio
65000000
65800000
Physical Position NCBI36, Chr 12
4225320287_A, nprobe = 13
Log R Ratio
41600000
41900000
Physical Position NCBI36, Chr 17
4225320287_A, nprobe = 8
Log R Ratio
9800000
10000000
Physical Position NCBI36, Chr 21
4225320288_B, nprobe = 16
Log R Ratio
39100000
39500000
Physical Position NCBI36, Chr 8
4225320288_B, nprobe = 15
Log R Ratio
43550000
43700000
Physical Position NCBI36, Chr 9
4225320288_B, nprobe = 40
Log R Ratio
46600000
47200000
Physical Position NCBI36, Chr 10
4225320288_B, nprobe = 16
Log R Ratio
21000000
21400000
21800000
Physical Position NCBI36, Chr 17
4225320288_B, nprobe = 24
Log R Ratio
153600000
154400000
Physical Position NCBI36, Chr X
4225320312_B, nprobe = 16
Log R Ratio
39100000
39500000
Physical Position NCBI36, Chr 8
4225320312_B, nprobe = 42
Log R Ratio
99400000
100000000
Physical Position NCBI36, Chr 15
4225320312_B, nprobe = 13
Log R Ratio
41600000
41900000
Physical Position NCBI36, Chr 17
4225320312_B, nprobe = 28
Log R Ratio
23600000
24200000
Physical Position NCBI36, Chr 22
4225320346_B, nprobe = 16
Log R Ratio
39100000
39500000
Physical Position NCBI36, Chr 8

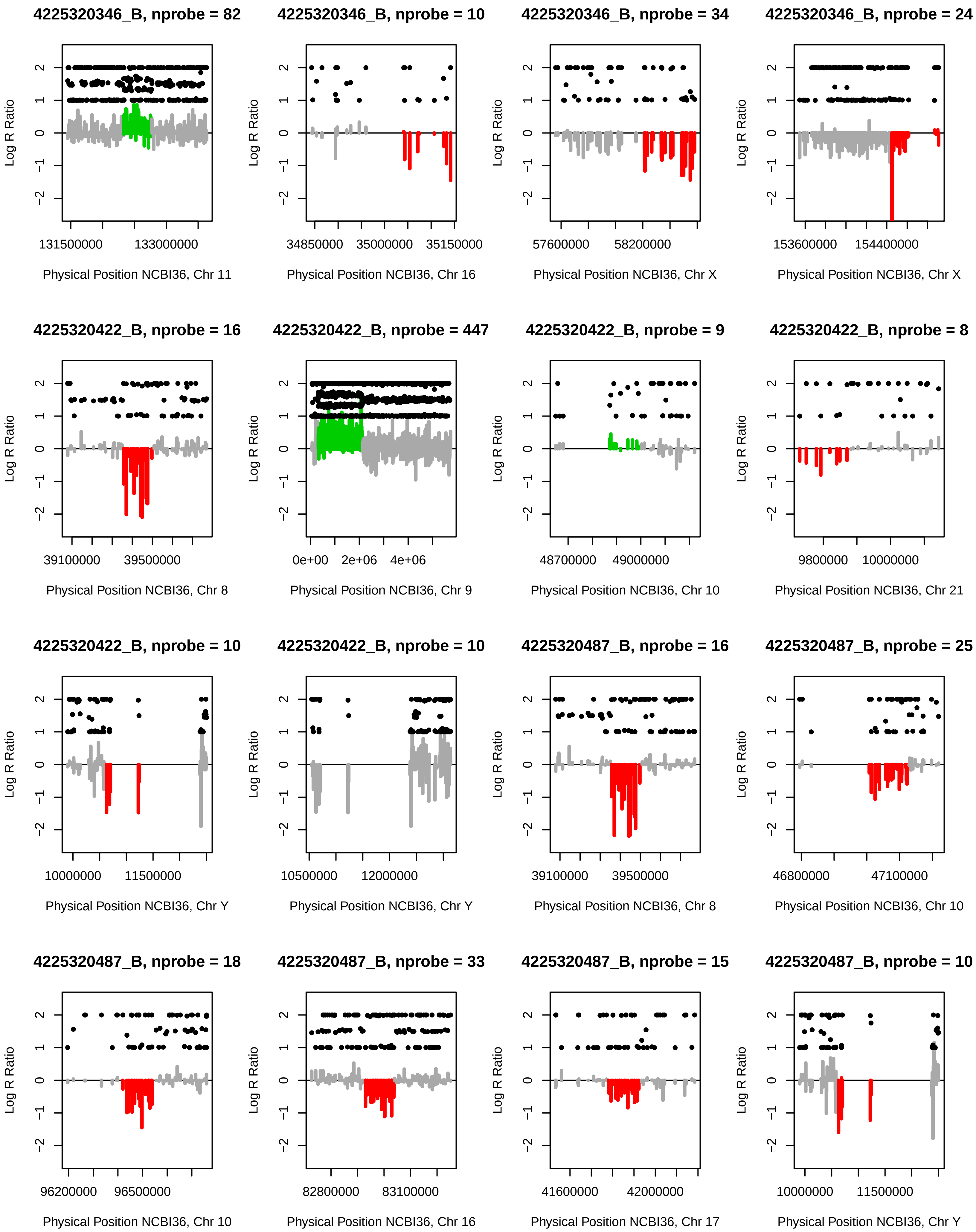
4225320346_B, nprobe = 82
4225320346_B, nprobe = 10
4225320346_B, nprobe = 34
4225320346_B, nprobe = 24
Log R Ratio
131500000
133000000
Physical Position NCBI36, Chr 11
34850000
35000000
35150000
Physical Position NCBI36, Chr 16
57600000
58200000
Physical Position NCBI36, Chr X
153600000
154400000
Physical Position NCBI36, Chr X
4225320422_B, nprobe = 16
4225320422_B, nprobe = 447
4225320422_B, nprobe = 9
4225320422_B, nprobe = 8
39100000
39500000
Physical Position NCBI36, Chr 8
0e+00
2e+06
4e+06
Physical Position NCBI36, Chr 9
48700000
49000000
Physical Position NCBI36, Chr 10
9800000
10000000
Physical Position NCBI36, Chr 21
4225320422_B, nprobe = 10
4225320422_B, nprobe = 10
4225320487_B, nprobe = 16
4225320487_B, nprobe = 25
10000000
11500000
Physical Position NCBI36, Chr Y
10500000
12000000
Physical Position NCBI36, Chr Y
39100000
39500000
Physical Position NCBI36, Chr 8
46800000
47100000
Physical Position NCBI36, Chr 10
4225320487_B, nprobe = 18
4225320487_B, nprobe = 33
4225320487_B, nprobe = 15
4225320487_B, nprobe = 10
96200000
96500000
Physical Position NCBI36, Chr 10
82800000
83100000
Physical Position NCBI36, Chr 16
41600000
42000000
Physical Position NCBI36, Chr 17
10000000
11500000
Physical Position NCBI36, Chr Y

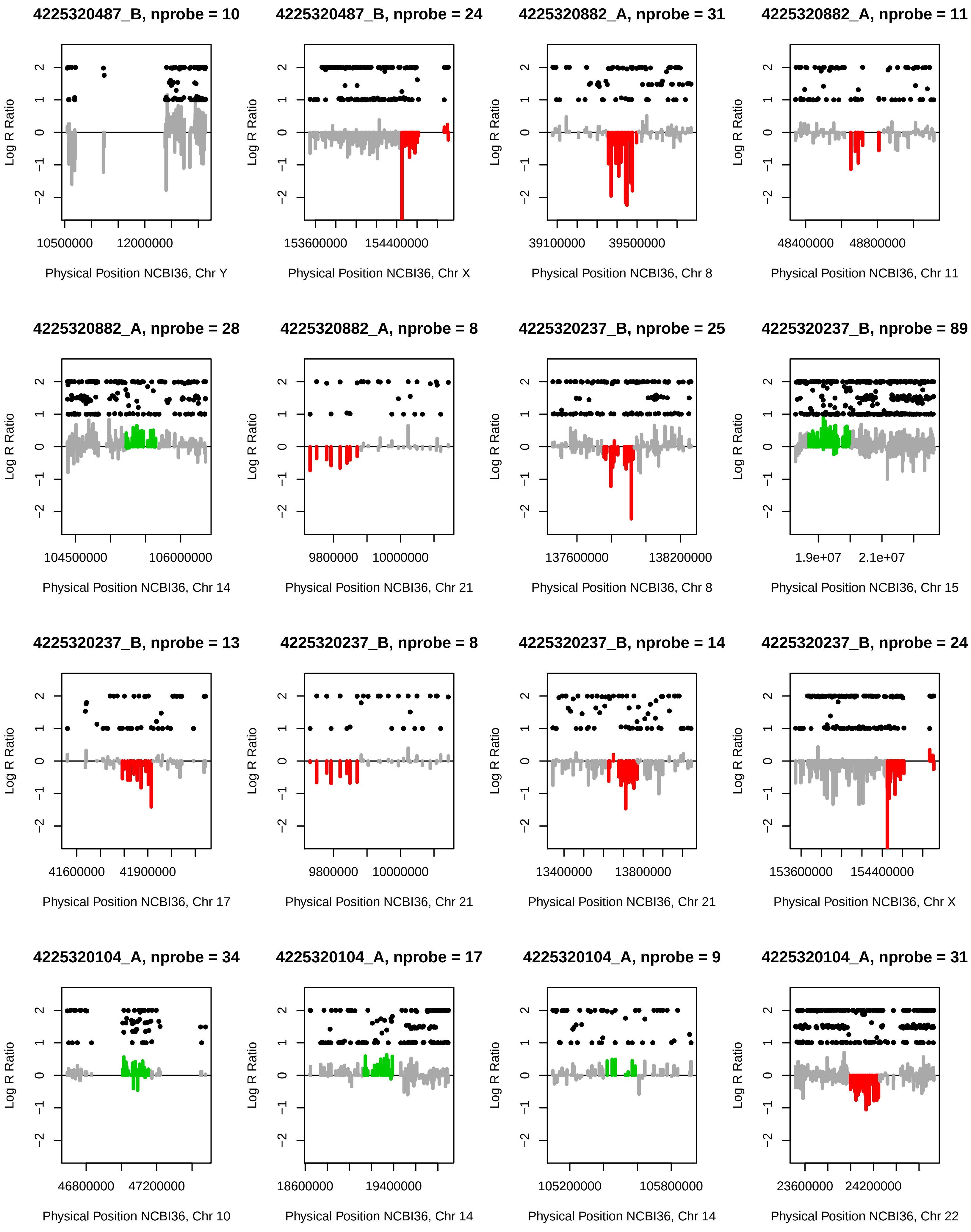
4225320487_B, nprobe = 10
Log R Ratio
2
1
0
−1
−2
10500000
12000000
Physical Position NCBI36, Chr Y
4225320487_B, nprobe = 24
153600000
154400000
Physical Position NCBI36, Chr X
4225320882_A, nprobe = 31
39100000
39500000
Physical Position NCBI36, Chr 8
4225320882_A, nprobe = 11
48400000
48800000
Physical Position NCBI36, Chr 11
4225320882_A, nprobe = 28
104500000
106000000
Physical Position NCBI36, Chr 14
4225320882_A, nprobe = 8
9800000
10000000
Physical Position NCBI36, Chr 21
4225320237_B, nprobe = 25
137600000
138200000
Physical Position NCBI36, Chr 8
4225320237_B, nprobe = 89
1.9e+07
2.1e+07
Physical Position NCBI36, Chr 15
4225320237_B, nprobe = 13
41600000
41900000
Physical Position NCBI36, Chr 17
4225320237_B, nprobe = 8
9800000
10000000
Physical Position NCBI36, Chr 21
4225320237_B, nprobe = 14
13400000
13800000
Physical Position NCBI36, Chr 21
4225320237_B, nprobe = 24
153600000
154400000
Physical Position NCBI36, Chr X
4225320104_A, nprobe = 34
46800000
47200000
Physical Position NCBI36, Chr 10
4225320104_A, nprobe = 17
18600000
19400000
Physical Position NCBI36, Chr 14
4225320104_A, nprobe = 9
105200000
105800000
Physical Position NCBI36, Chr 14
4225320104_A, nprobe = 31
23600000
24200000
Physical Position NCBI36, Chr 22

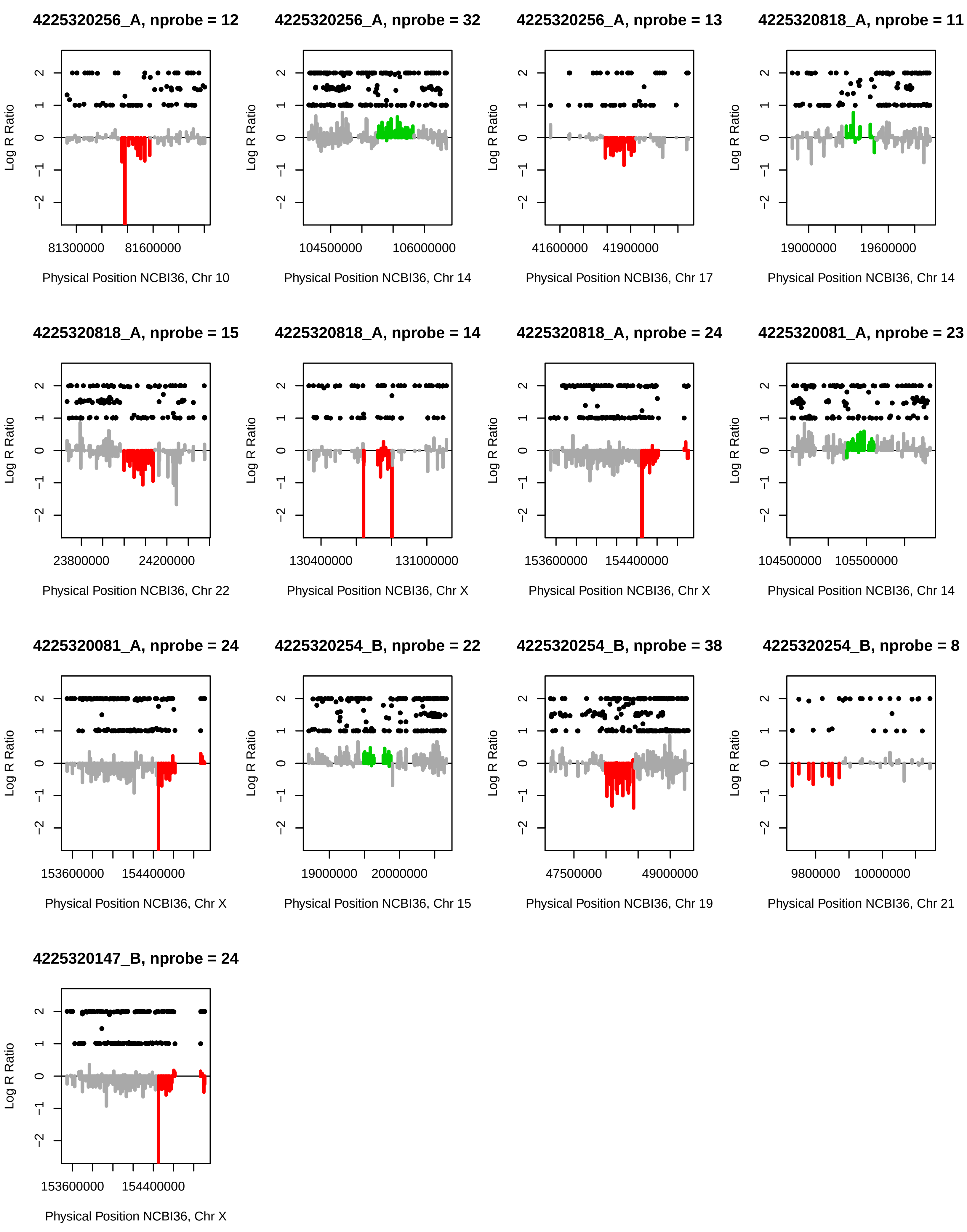
4225320256_A, nprobe = 12
Log R Ratio
81300000
81600000
Physical Position NCBI36, Chr 10
4225320256_A, nprobe = 32
Log R Ratio
104500000
106000000
Physical Position NCBI36, Chr 14
4225320256_A, nprobe = 13
Log R Ratio
41600000
41900000
Physical Position NCBI36, Chr 17
4225320818_A, nprobe = 11
Log R Ratio
19000000
19600000
Physical Position NCBI36, Chr 14
4225320818_A, nprobe = 15
Log R Ratio
23800000
24200000
Physical Position NCBI36, Chr 22
4225320818_A, nprobe = 14
Log R Ratio
130400000
131000000
Physical Position NCBI36, Chr X
4225320818_A, nprobe = 24
Log R Ratio
153600000
154400000
Physical Position NCBI36, Chr X
4225320081_A, nprobe = 23
Log R Ratio
104500000
105500000
Physical Position NCBI36, Chr 14
4225320081_A, nprobe = 24
Log R Ratio
153600000
154400000
Physical Position NCBI36, Chr X
4225320254_B, nprobe = 22
Log R Ratio
19000000
20000000
Physical Position NCBI36, Chr 15
4225320254_B, nprobe = 38
Log R Ratio
47500000
49000000
Physical Position NCBI36, Chr 19
4225320254_B, nprobe = 8
Log R Ratio
9800000
10000000
Physical Position NCBI36, Chr 21
4225320147_B, nprobe = 24
Log R Ratio
153600000
154400000
Physical Position NCBI36, Chr X
