# Supplementary figures and images for: Human Spermatogenic Failure Purges Deleterious Mutation Load from the Autosomes and Both Sex Chromosomes, including the Gene DMRT1
Source: PLoS Genet. 2013 Mar 21;9(3):e1003349. doi: 10.1371/journal.pgen.1003349 (PMC3605256; doi:10.1371/journal.pgen.1003349)

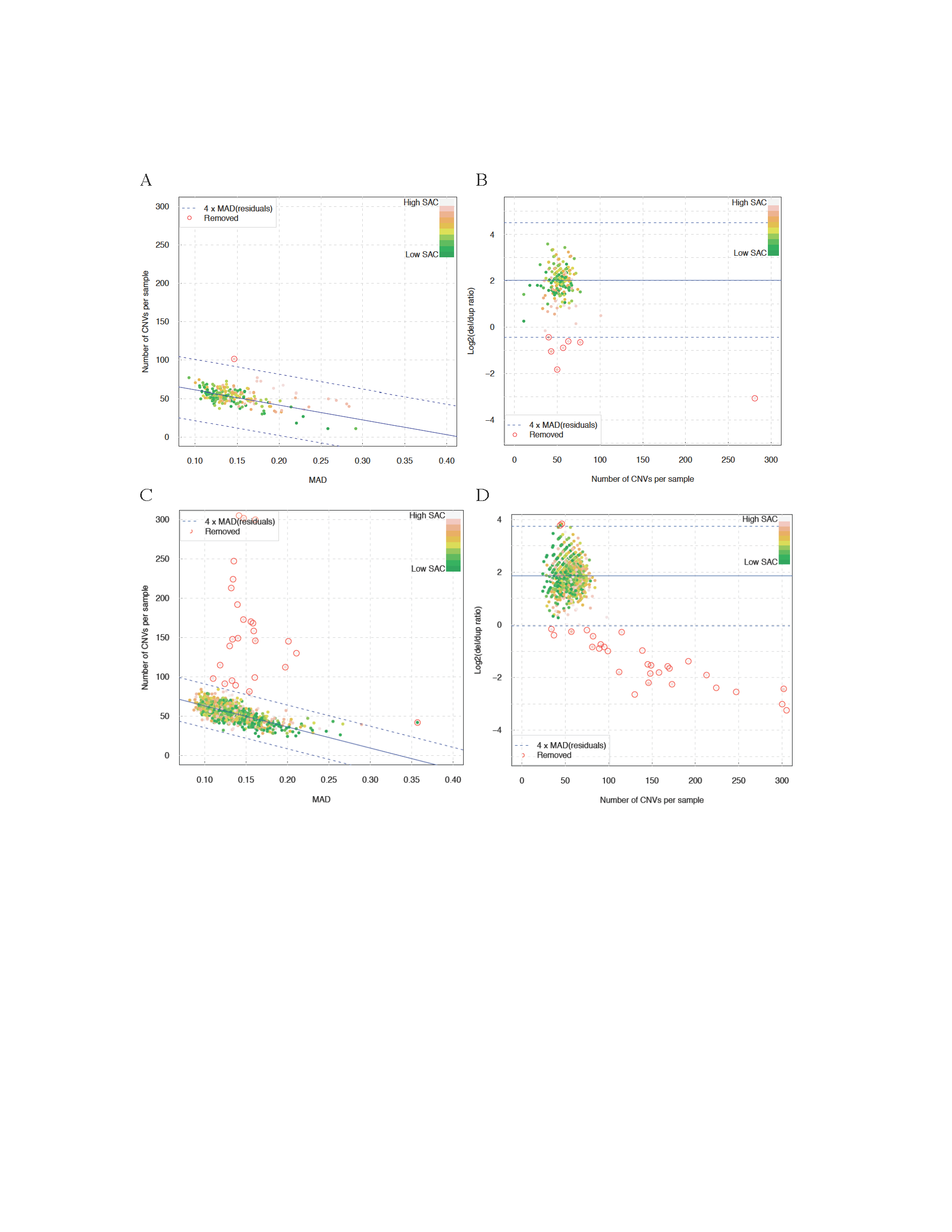

Supplement: Figure S1 — QC of Affymetrix callsets. Summary plots of array QC for the case samples and NBS control samples. There is an expected inverse correlation between the noise in the data (measured by the median absolute deviation (MAD) of the probe intensities) and the number of calls made in a particular experiment. We fit a linear model to these parameters separately for cases (A) and NBS controls (C), and samples >4 MADs from the fitted model were removed (circled dots). We also implemented an analogous QC step using the ratio of deletions/duplication calls per sample and number of calls per sample, separately for cases (B) and NBS controls (D), . In all plots, arrays are colored by their spatial autocorrelation function (a measure of “waviness”). Distributions of post-QC statistics are highly similar between cases and controls. (TIF) [file pgen.1003349.s002.tif]

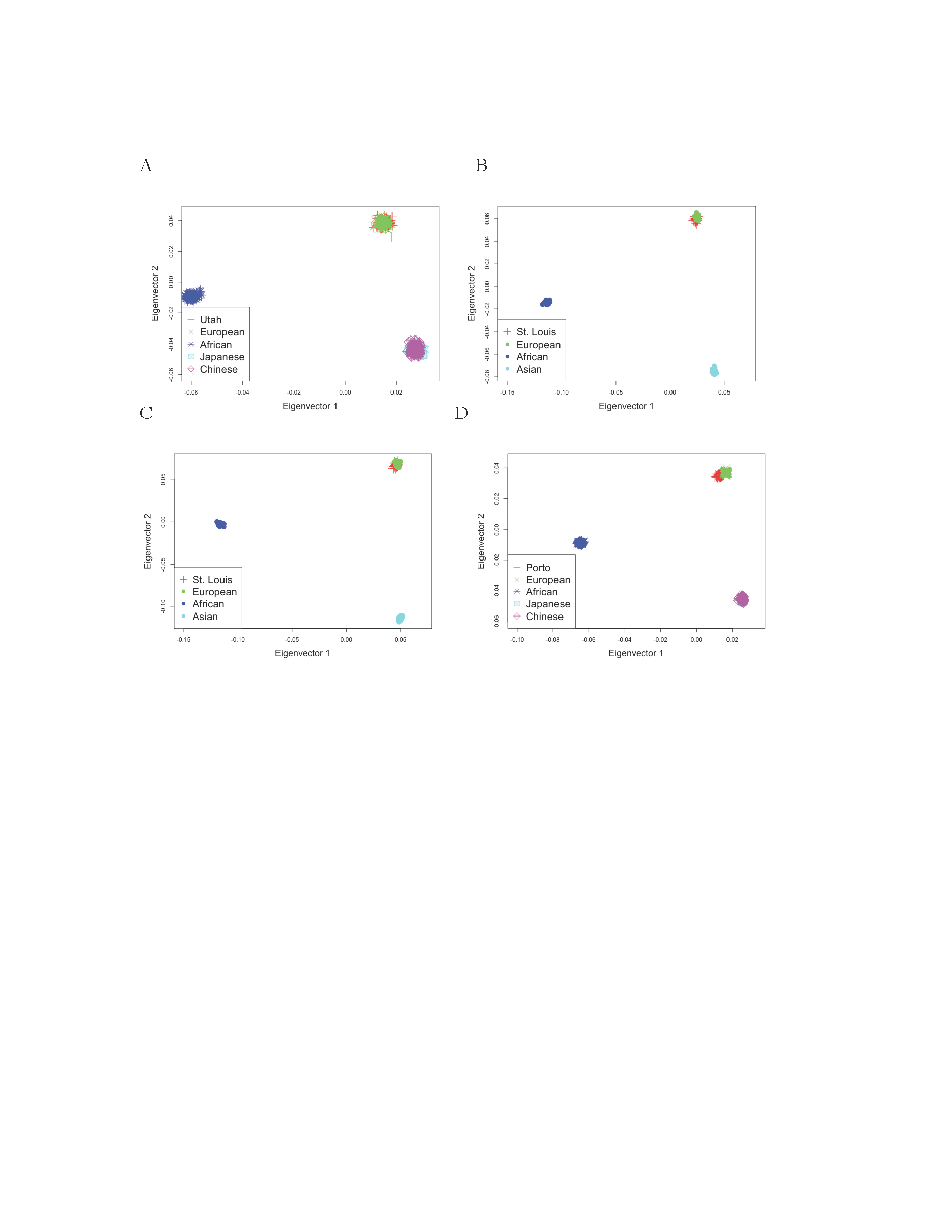

Supplement: Figure S2 — Principal components analysis (PCA) of population structure in all case cohorts post-QC. For each cohort, samples were analyzed together with HapMap samples using the EIGENSOFT package [69]. (A) Utah (B) WUSTL batch 1, (C) WUSTL batch 2, (D) Porto. Eigenvector loadings for cases and controls (A,B,C) or cases (D) are plotted as red crosses, while HapMap samples are plotted as other colored symbols described in each legend. (TIF) [file pgen.1003349.s003.tif]

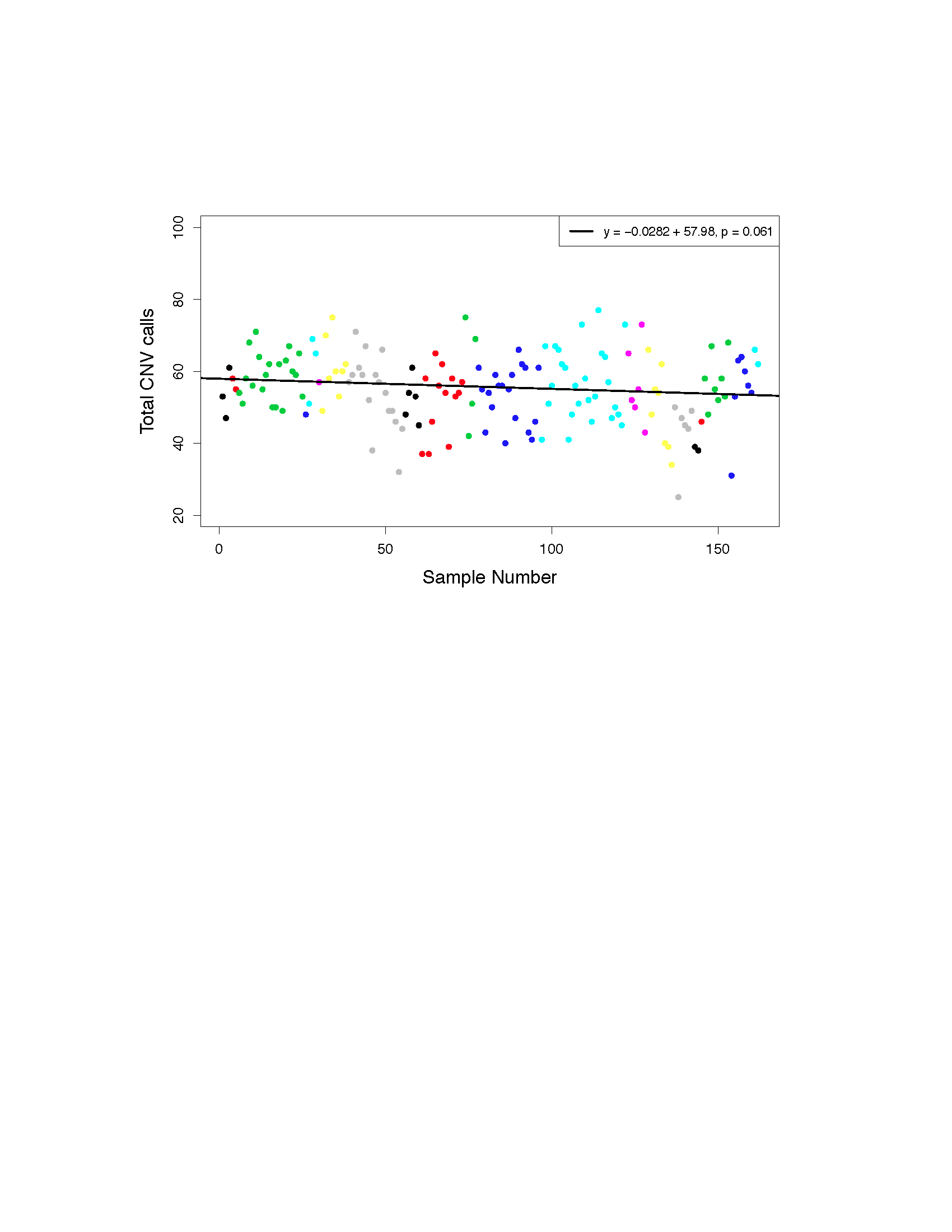

Supplement: Figure S4 — Analysis of batch effects in Porto cases. The Porto arrays were run over a period of several months. Here we plot the total number of CNV calls per array, as a function of run order (sample number 1 = first array run, sample number 162 = last array run). Each dot is colored based on run date. No obvious outlier batches are visible. There was a small but insignificant trend for fewer CNV calls on later run dates (least-squares regression line is plotted). (TIF) [file pgen.1003349.s005.tif]

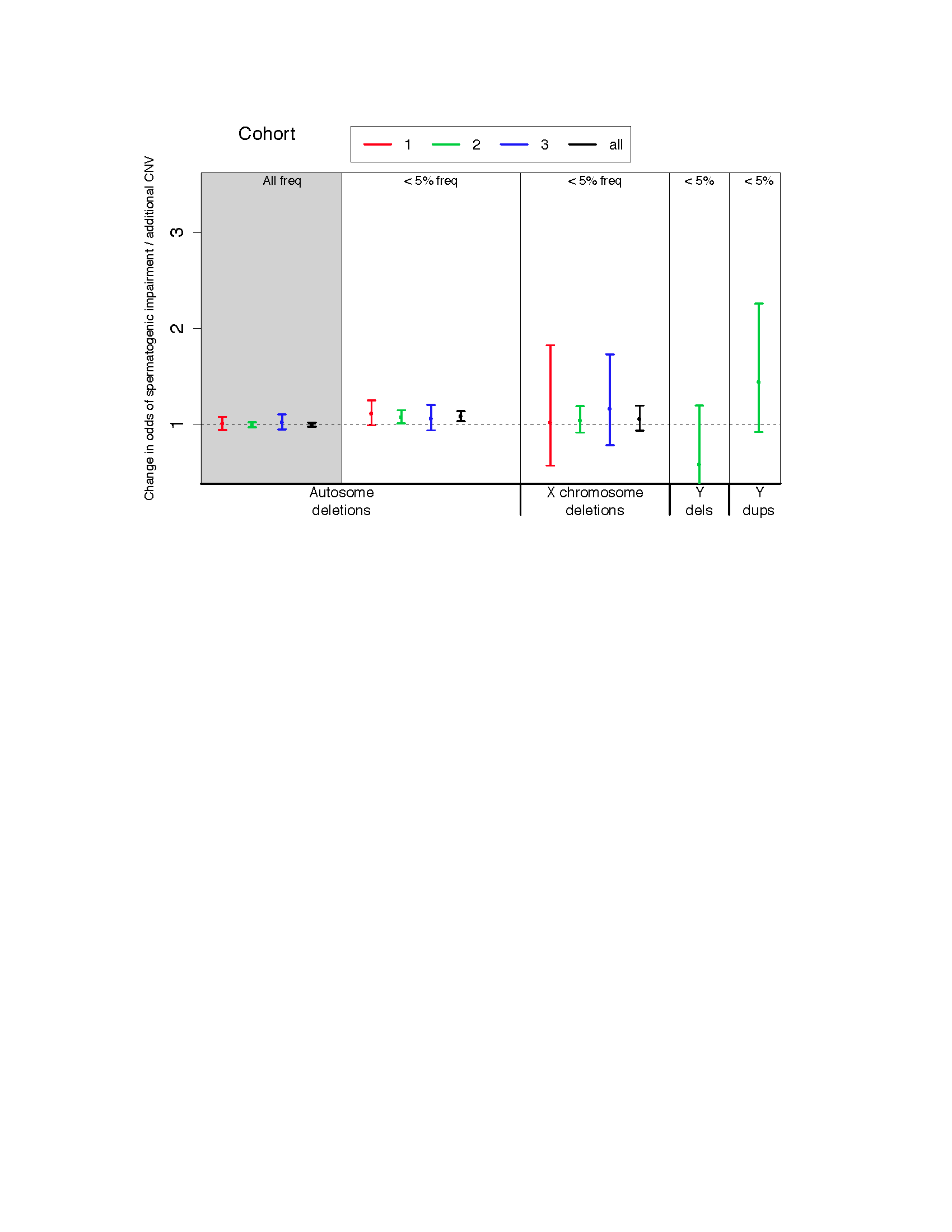

Supplement: Figure S5 — CNV burden statistics using a Spanish control cohort. In the primary CNV analyses described in the main text, we use a Caucasian population from the United Kingdom as a control group for the Porto azoospermia cohort. Here, we address the effect of using a control group that is more closely matched on genetic ancestry. We performed the same burden analyses depicted in Figure 1 of the main text, this time using a much smaller control cohort of 368 Caucasian men ascertained in Spain. We used logistic regression to estimate the influence of copy number variants (CNVs) on the odds of being diagnosed with impaired spermatogenesis in three case-control cohorts. Eigenvectors from a principal components analysis were used as covariates as before. The odds ratio estimated from fitting a logistic regression model of total CNV count to disease status is plotted separately for each cohort, as well as the combined set of all cohorts (black points). Cohort 1 = Utah (Illumina 370K), 2 = Porto and Weill Cornell (Affymetrix 6.0), 3 = WUSTL (Illumina OmniExpress). Sample sizes used in CNV analysis are n = 83 cases and n = 62 controls for cohort 1, n = 179 cases and 368 controls for cohort 2, and n = 61 cases and 100 controls for cohort 3. Conclusion: While the direction of burden effect for rare autosomal deletions, X-linked deletions, and Y duplications was the same as seen with the analysis using UK controls, only the rare autosomal deletion burden model shows statistical evidence for an odds ratio greater than 1. (TIF) [file pgen.1003349.s006.tif]

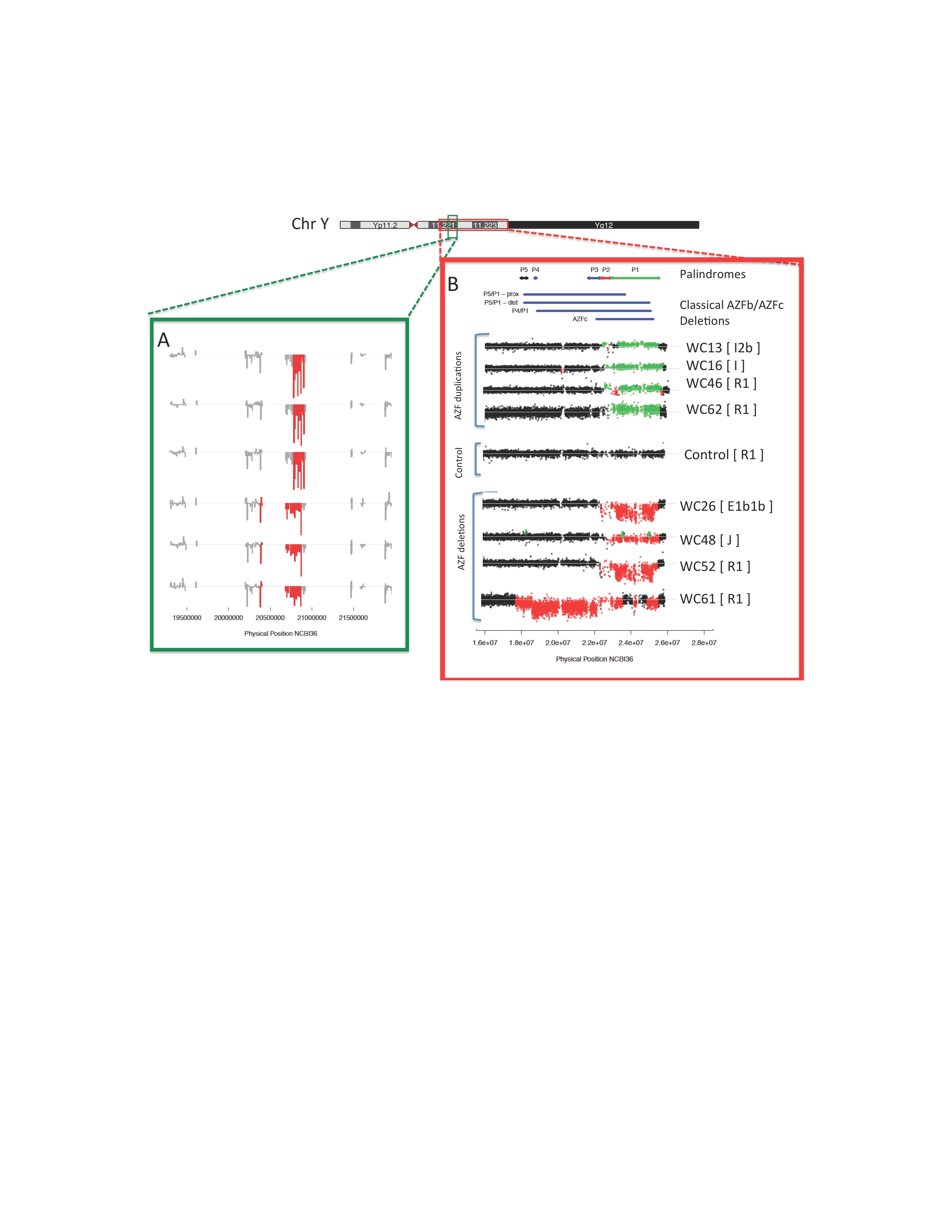

Supplement: Figure S6 — CNVs on the Y chromosome. (A) Our strongest statistical association involved gains and losses of a 200 kb tandem repeat termed DYZ19, approximately 500 kb distal to palindrome P4. Here are plotted 6 deletions from the UT cohort, which were evenly distributed between azoospermic and oligozoospermic men. (B) In the Weill-Cornell cohort, a small group of azoospermic individuals ascertained at a tertiary care clinic, we identified a number of classical AZF deletions, as well as duplications of AZFc. Next to each CNV is listed the sample ID and Y haplogroup of the sample inferred from SNP data (Methods). These data demonstrate that existing SNP platforms can cleanly identify Y chromosome rearrangements involving both gain and loss of sequence, and will facilitate investigation of the full spectrum of Y chromosome variation in future studies of male infertility. Notably, we observed complex patterns of copy number change in some samples that highlight the challenge of interpreting array data mapped to a single reference Y chromosome (haplogroup R1). In both panels, for each individual, deviations of probe log2 ratios from 0 are depicted by grey lines or black dots, and probes spanning CNV calls are colored as either red (losses) or green (gains). (TIF) [file pgen.1003349.s007.tif]

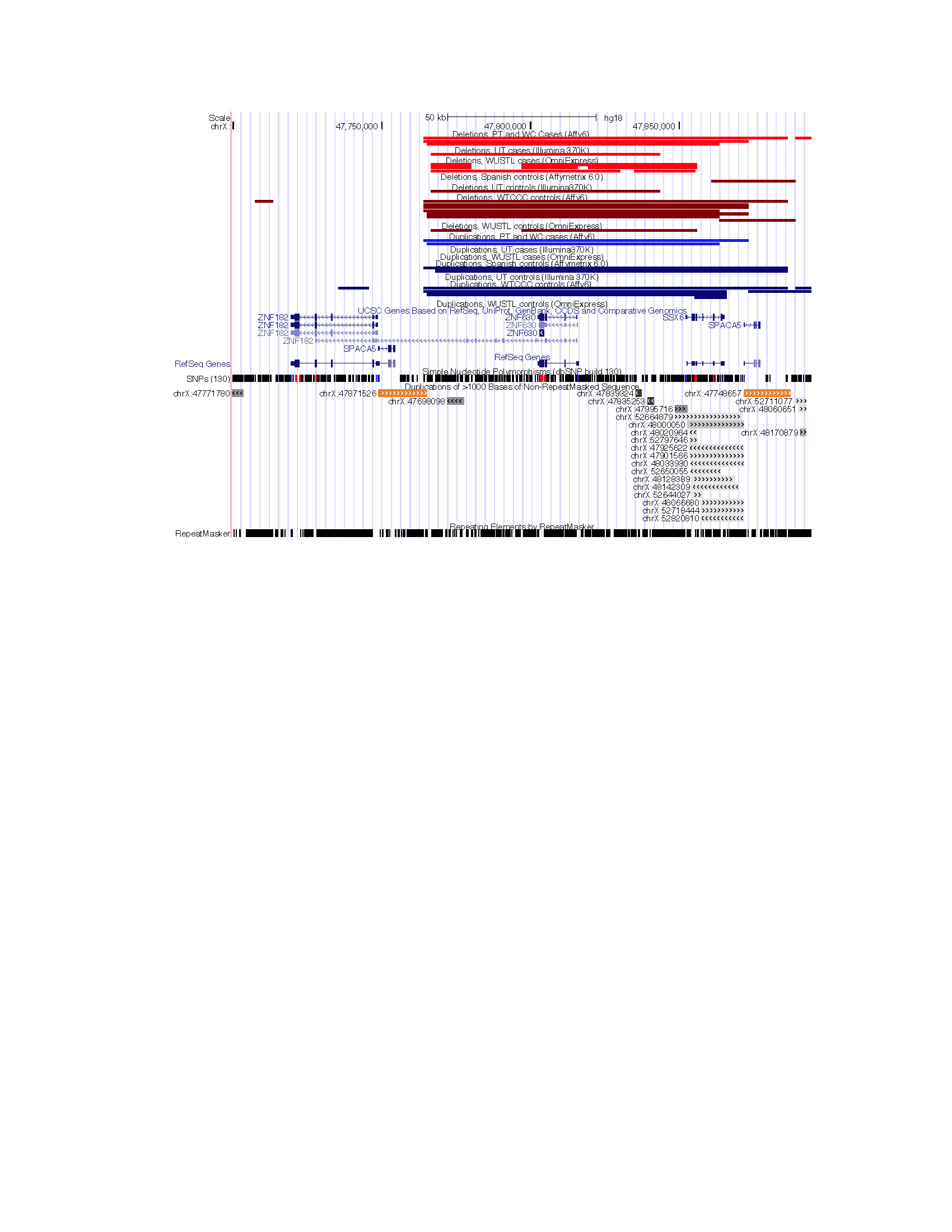

Supplement: Figure S7 — CNV calls made in all array datasets at the Xp11 rearrangement hotspot. This plot is the same as Figure 2a, with the addition of tracks containing deletion and duplication calls from a Spanish male control cohort assayed on the Affymetrix 6.0 platform. (TIF) [file pgen.1003349.s008.tif]

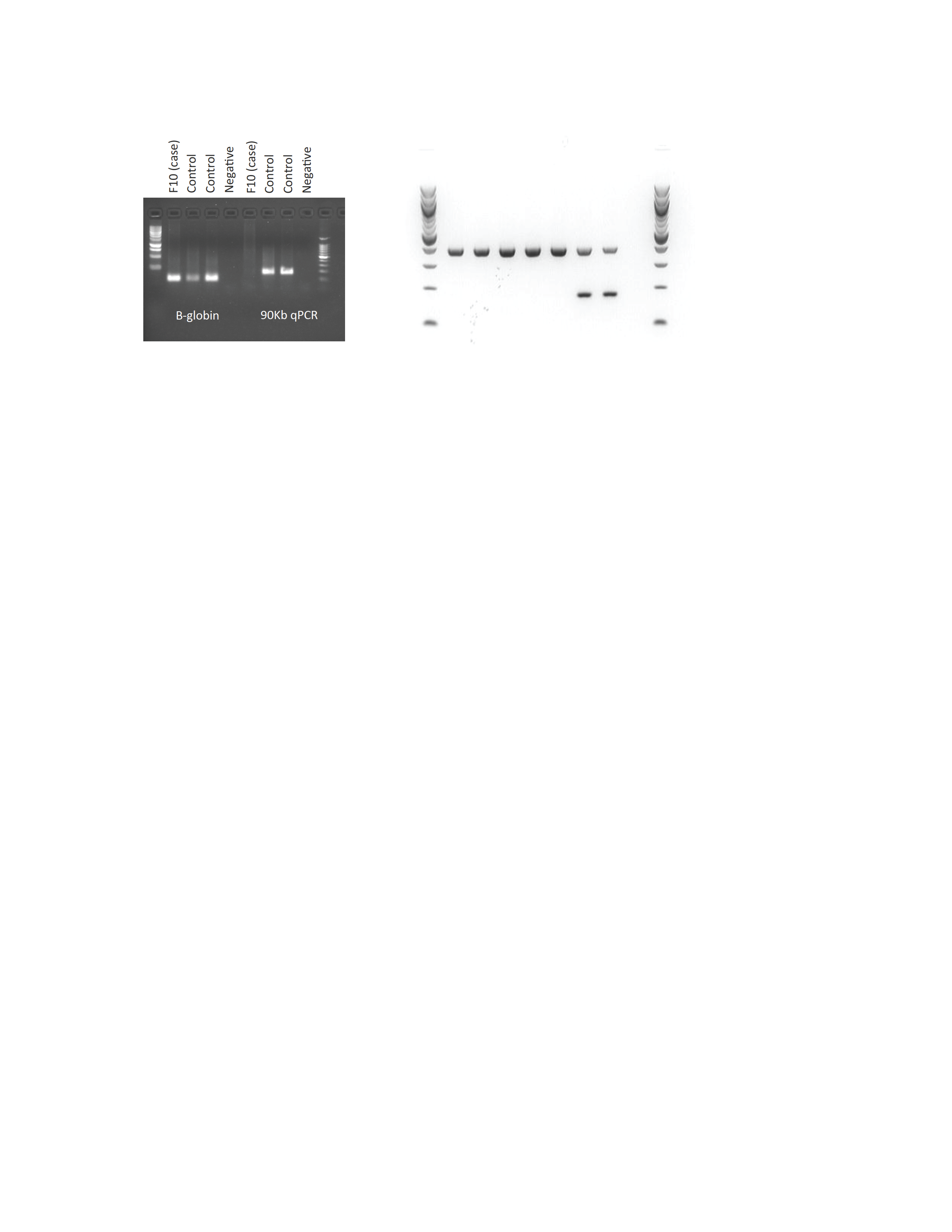

Supplement: Figure S8 — Left, example STS PCR validation of Xp11 in one case from Cornell (F10) and two controls. Right, the same assay, run in multiplex (Xp11 and B-globin reactions in the same tube) for 5 WUSTL case carriers and two controls. Note the presence of the smaller beta-globin band in the two control individuals in lanes 7 and 8. The primer sequences for the Xp11 deletion assay and a control locus are given in the Text S1. (TIF) [file pgen.1003349.s009.tif]

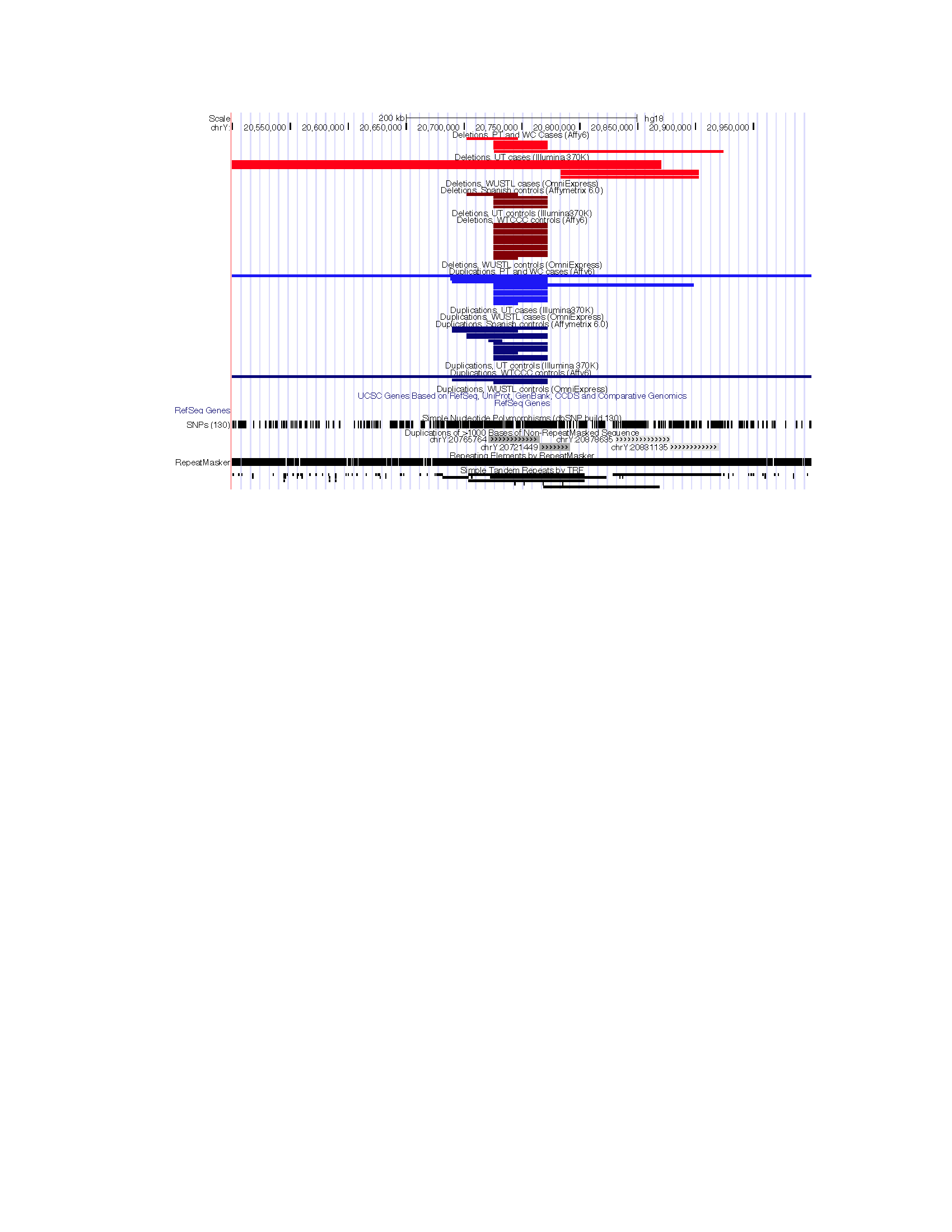

Supplement: Figure S9 — CNV calls made at the DYZ19 tandem repeat locus. CNV calls made on the Utah, Porto, and WUSTL case cohorts, and the Utah, NBS (WTCCC), WUSTL and Spanish control cohorts. (TIF) [file pgen.1003349.s010.tif]

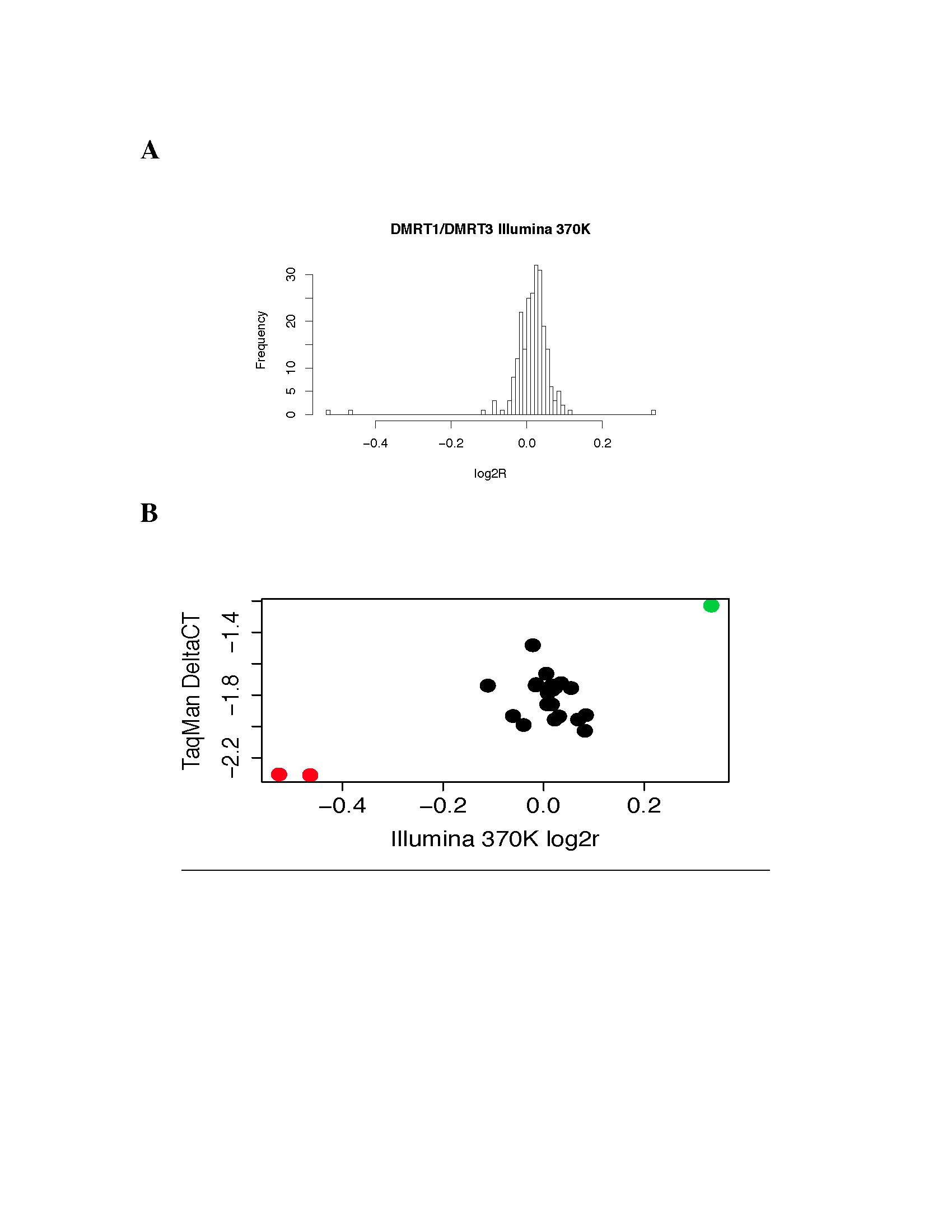

Supplement: Figure S10 — qPCR validation of DMRT1 deletions in the Utah Cohort. (A) Histogram of mean probe intensities from Illumina 370K array spanning the DMRT1/DMRT3 deletion locus (chr9:845901–994958 bp). N = 148 samples are plotted. (B) Taqman validation results for the DMRT1 locus from 30 of the samples screened by 370K array in panel A (y-axis), including the two deletion carriers (red points) and one duplication carrier (green point) identified from Illumina 370K intensity data (x-axis). (TIF) [file pgen.1003349.s011.tif]

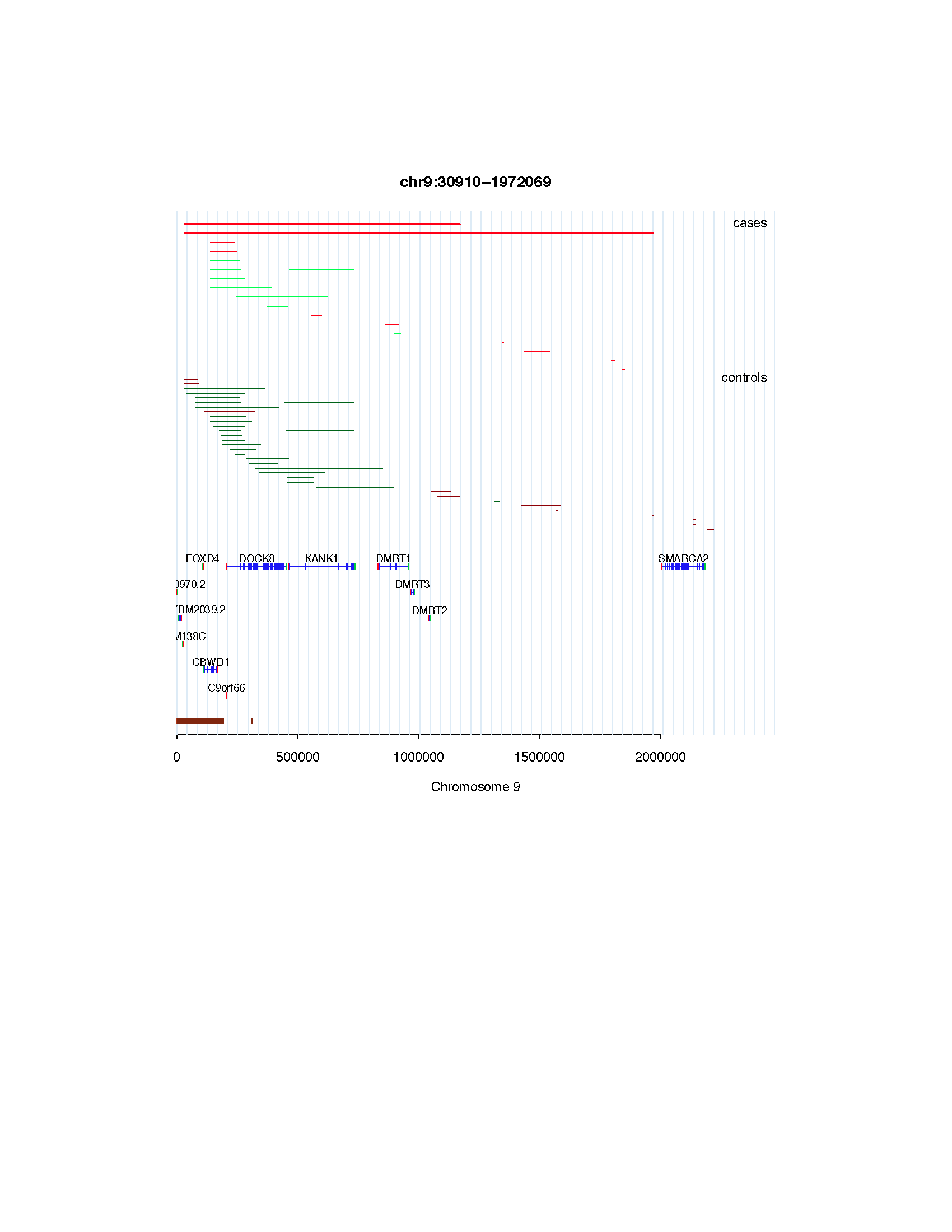

Supplement: Figure S11 — CNVs on chromosome 9p in the Nanjing cohort. Affymetrix 6.0 data was generated on a cohort of 979 idiopathic NOA cases and ethnicity-matched controls 1734 controls recruited primarily from the cities of Nanjing and Wuhan, China. CNVs were called using the identical pipeline as the other cohorts. Plotted above are all of the deletions (red) and duplications (green) observed in these cases (top) and controls (bottom). (TIF) [file pgen.1003349.s012.tif]

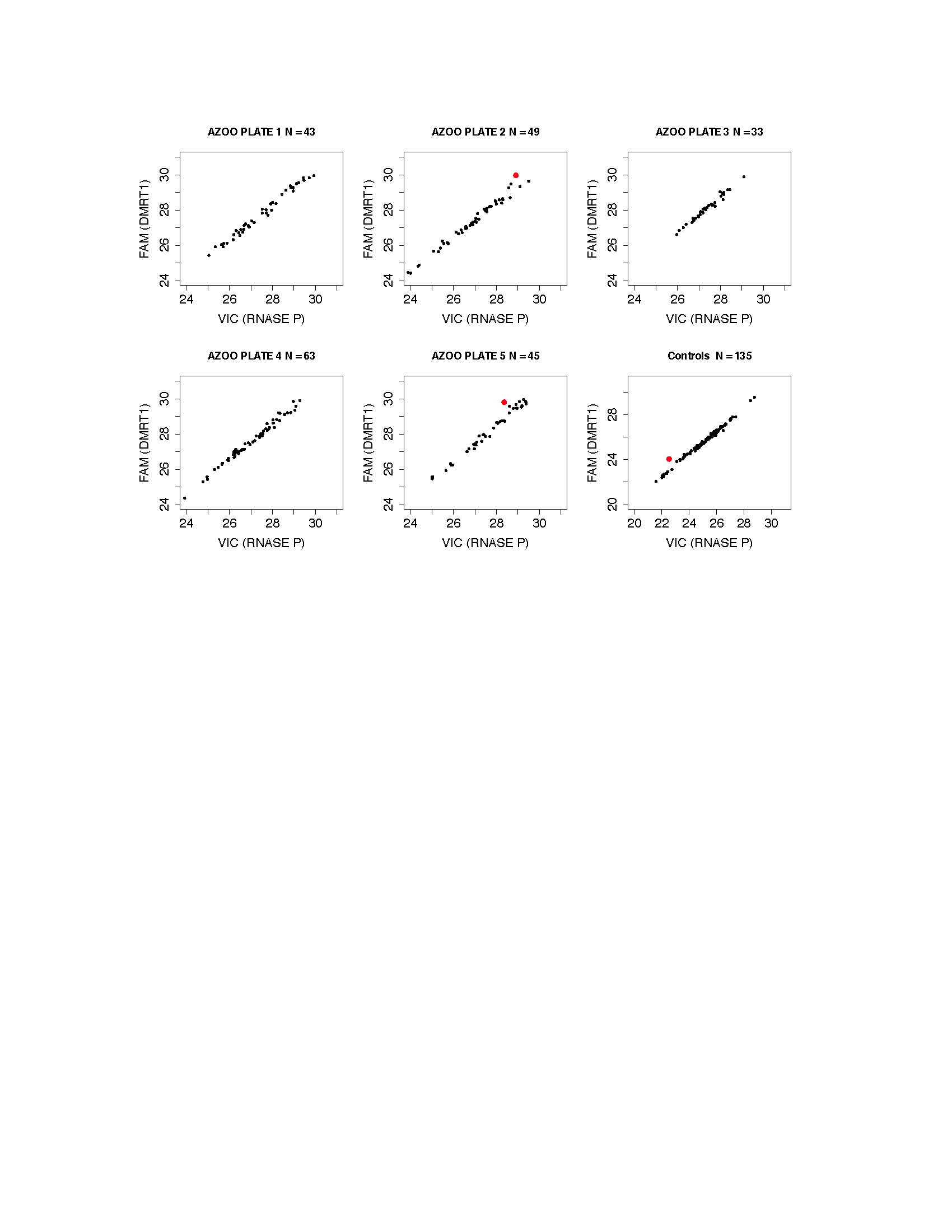

Supplement: Figure S12 — Detection of additional intronic DMRT1 deletions using the TaqMan validation assay. As described in the supplemental methods, we screened 5 plates of DNA from Weill Cornell cases and 2 plates of Caucasian male controls using the DMRT1 TaqMan validation assay. Each sample was assayed in quadruplicate (although some samples were assayed in duplicate or triplicate if insufficient DNA was available). We applied extremely stringent calling criteria to these data, excluding samples with (1) low DNA content as defined by picogreen assay (2) high standard deviation (>0.3) of delta CT measurements across replicates (3) low copy number confidence scores generated by CopyCaller software. Each point in the panels above represents the average delta CT for the control locus (VIC) and DMRT1 (FAM) for a single sample. Red dots indicate deletion carrier calls. We detected 2 deletions in 233 case samples, a frequency of 0.86%, and 1 deletion in 135 controls (0.74%). As this assay is targeting intronic sequence, and we have not cloned the breakpoints the functional consequences of each deletion is unclear. (TIF) [file pgen.1003349.s013.tif]

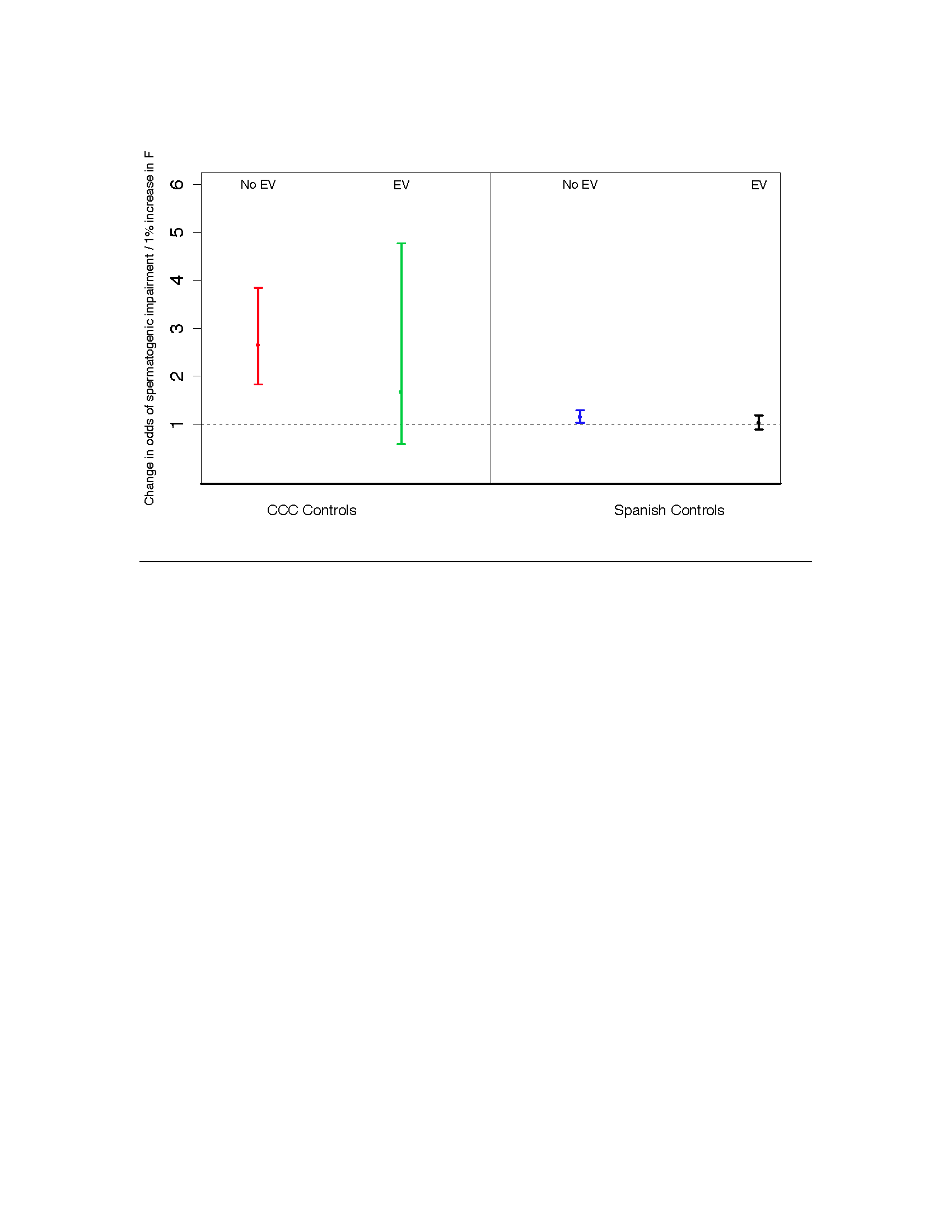

Supplement: Figure S13 — Controlling for population structure while testing for association between inbreeding and infertility. As described in Figure S2 and in Text S1, we used principal components analysis to assess population structure in our case and control cohorts. We use the BEAGLE software package to define homozygous-by-descent regions (HBDRs) of each sample in our study, and used these HBDRs to estimate corresponding inbreeding coefficients. We tested for association between inbreeding coefficient and the probability of azoospermia using a linear model. On the left, we show that there is strong association when analyzing data from the Porto cases and NBS Wellcome Trust case-control consortium controls (CCC controls) in a simple model that does not account for population structure (“No EV”). When including the first 10 eigenvectors from a PCA analysis (“EV”), the point estimate of association remains somewhat inflated but is no longer significant. On the right, we show the same analysis performed on the Porto cohort with a more closely matched control population from Spain, with clearly smaller confidence intervals and smaller point estimates of effect size. In order to be as conservative as possible, we report results of inbreeding analysis using the Spanish control cohort in the main text. (TIF) [file pgen.1003349.s014.tif]
