# Supplementary material for: Human Spermatogenic Failure Purges Deleterious Mutation Load from the Autosomes and Both Sex Chromosomes, including the Gene DMRT1
Source: PLoS Genet. 2013 Mar 21;9(3):e1003349. doi: 10.1371/journal.pgen.1003349 (PMC3605256; doi:10.1371/journal.pgen.1003349)
Supplement: Text S1 — A description of patient cohorts, supplementary methods and analyses. (DOCX) [file pgen.1003349.s018.docx]

Supplementary material

for

“Human spermatogenic failure purges deleterious mutation load from the autosomes and both sex chromosomes, including the gene *DMRT1*”

Alexandra M. Lopes^1,*^, Kenneth I. Aston^2,*^, Emma Thompson^3^, Filipa Carvalho^4^, João Gonçalves^5^, Ni Huang^6^ , Rune Matthiesen^1^, Michiel J. Noordam^6^, Inés Quintela^7^, Avinash Ramu^6^, Catarina Seabra^1^, Amy B. Wilfert^6^, Juncheng Dai^8^, Jonathan M. Downie^9^, Susana Fernandes^4^, Xuejiang Guo^10,11^, Jiahao Sha^10,11^, António Amorim^1,12^, Alberto Barros^4,13^, Angel Carracedo^7,14^, Zhibin Hu^8,10^, Matthew E. Hurles^15^, Sergey Moskovtsev^16,17^, Carole Ober^3,18^, Joshua D. Schiffman^9,19,20^, Peter N. Schlegel^21^, Mário Sousa^22^, Douglas T. Carrell^2,23,24^, Donald F. Conrad^6,25^

^1^IPATIMUP, Institute of Molecular Pathology and Immunology of the University of Porto, R. Dr. Roberto Frias S/N, 4200-465 Porto, Portugal;

^2^Andrology and IVF Laboratories, Department of Surgery, ^23^ Department of Physiology, ^24^Department of Obstetrics and Gynecology, University of Utah School of Medicine, Salt Lake City, Utah, 84108, USA;

^3^Department of Human Genetics, ^18^Department of Obstetrics & Gynecology, The University of Chicago, Chicago, IL 60637, USA;

^4^Department of Genetics, Faculty of Medicine, University of Porto, Porto, Portugal;

^5^Department of Human Genetics, National Institute of Health Dr. Ricardo Jorge, Lisbon, Portugal;

^6^Department of Genetics, ^25^Department of Pathology & Immunology, Washington University School of Medicine, St. Louis, MO 63110, USA;

^7^Genomics Medicine Group, National Genotyping Center, University of Santiago de Compostela, Santiago de Compostela, Spain;

^8^Department of Epidemiology and Biostatistics and Key Laboratory of Modern Toxicology of Ministry of Education, School of Public Health, ^10^State Key Laboratory of Reproductive Medicine, ^11^Department of Histology and Embryology, Nanjing Medical University, Nanjing, China.

^9^Department of Oncological Sciences, ^19^Center for Children's Cancer Research (C3R),^20^ and Division of Pediatric Hematology/Oncology, Huntsman Cancer Institute,University of Utah School of Medicine, Salt Lake City, UT 84112, USA;

^12^Faculty of Sciences, University of Porto, 4099-002 Porto, Portugal;

^13^Centre for Reproductive Genetics Alberto Barros, Porto, Portugal;

^14^Galician Foundation of Genomic Medicine and University of Santiago de Compostela, CIBERER Santiago de Compostela, Spain;

^15^Genome Mutation and Genetic Disease Group, Wellcome Trust Sanger Institute, Cambridge, UK;

^16^ CReATe Fertility Center, ^17^ Department of Obstetrics & Gynaecology, University of Toronto

^21^Department of Urology, Weill Cornell Medical College, New York-Presbyterian Hospital, New York, USA;

^22^Laboratory of Cell Biology, UMIB, ICBAS, University of Porto, Porto, Portugal;

*Joint First Authors

**Supplementary Methods**

**Patient Cohorts**

*Utah Cohort*

Following a complete reproductive history, physical examination, hormonal testing (FSH, testosterone), and standard clinical genetic screening for karyotypic anomalies and Y chromosome microdeletions, and complete semen analysis, only idiopathic azoospermic (n = 66) and severe oligozoospermic men (< 5 million sperm/ml; n = 59) were included in the patient group. Controls (n = 99) displayed normal semen parameters including sperm concentration > 20 million/ml, progressive sperm motility > 40%, viability > 50% based on membrane structure test, and morphologically normal heads > 30% and tails > 65% according to World Health Organization criteria. The study was approved by the University of Utah Institutional Review Board.

*Porto Cohort*

Following physical examination, hormonal testing (FSH, testosterone),standard clinical genetic screening for karyotypic anomalies and Y chromosome microdeletions, and multiple complete semen analyses only idiopathic azoospermic (n=183) and severe oligozoospermic men (< 5 million sperm/ml; n = 5) were included in the patient group.Histological evaluation following testicular biopsy was available for 36 cases: 24 SCOS (Sertoli-cell-only) and 12 MA (maturation arrest). The diagnosis of SCOS was established if only Sertoli cells were present in at least one (bilateral and multilocus) testicular biopsy (4 cases with rare germ cells in one of the biopsies – either BTD or BTT). The samples were collected at Departamento de Genética, INSA-IP (Lisbon, Portugal) and at Serviço de Genética, FMUP (Porto, Portugal) where the routine for male infertility diagnosis is well established.

The study was approved by the INSA Ethics Committee and Hospital Authority. All samples of genomic DNA to be analysed in this study i) belong to DNA banks that have been established throughout the years; ii) are coded; ii) each individual has signed a declaration of informed consent before donating his genomic DNA for analysis, authorizing molecular studies to be performed with this material.

*Washington University School of Medicine*

71 cases of idiopathic non-obstructive azoospermia or severe oligozoospermia from Washington University School of Medicine in St. Louis, using the same phenotypic criteria described above for Utah. This study was approved by the Washington University School of Medicine Institutional Review Board, IRB ID #201107177.

*Weill Cornell Medical College*

A cohort of 500 cases non-obstructive azoospermia was recruited at New York-Presbyterian Hospital. These cases were not karyotyped or screened for Y chromosome microdeletions. Forty cases were screened by Affymetrix 6.0 microarray; the remaining cases were used as a replication cohort for locus-specific assays described below.

*Controls*

Controls for the Affymetrix 6.0 experiments were obtained from two sources. A cohort of healthy unrelated individuals, all of them of Caucasian European origin and from Spain. DNA was obtained from peripheral blood by standard extraction methods. All samples were obtained with written informed consent reviewed by the ethical board of the corresponding hospital, in accordance with the Declaration of Helsinki. Studied subjects were from the control group of EPICOLON, a prospective, multicenter, nationwide Spanish initiative. A second male-only control dataset from the UK National Blood Service (NBS) was generated as part of the Wellcome Trust Case Control Consortium (<http://www.wtccc.org.uk/>).

Male control data for the OmniExpress platform were recruited and genotyped in St. Louis by the COGA project. These samples were unrelated Caucasians participating in a genetic study of alcoholism. Results of semen analysis were not available from Spanish, WTCCC or COGA control individuals.

*Nanjing Replication Cohort*

A large case-control cohort including 1,000 cases of non-obstructive azoospermia recruited from the Center of Reproductive Medicine at Nanjing Medical University, and 1,703 ethnicity-matched male controls from Nanjing, was screened on Affymetrix 6.0 arrays as previously described([3](#_ENREF_3)). All infertile male subjects were genetically unrelated Han Chinese men determined to have idiopathic NOA and selected on the basis of comprehensive andrological testing, including examination of medical history, physical examination, semen analysis, scrotal ultrasound, hormone analysis, karyotyping and Y chromosome microdeletion screening. Those with a history of cryptorchidism, vascular trauma, orchitis, obstruc­tion of the vas deferens, vasectomy, abnormalities in chromosome number or microdeletions of the azoospermia factor region on the Y chromosome were excluded from the study. Semen analysis for sperm concentration, motility and morphology was performed following World Health Organization (WHO) cri­teria (1999). Subjects with NOA had no detectable sperm in the ejaculate after evaluation of the centrifuged pellet. To ensure the reliability of the diagnosis, each individual was examined twice, and the absence of spermatozoa from both replicate samples was taken to indicate azoospermia.

**Microarray Analysis and Quality Control**

*Utah*

DNA was extracted from whole blood using the Gentra Puregene Blood Kit (Qiagen, Valencia, California) according to manufacturer’s recommendations. Precipitated DNA was re-suspended in Tris-EDTA, and concentration was determined using the NanoDrop 1000 spectrophotometer (Thermo Scientific, Waltham, Massachusets) and subsequently adjusted to approximately 100 ng/µl.

DNA was processed, and microarrays were run by deCODE Genetics (Reykjavik, Iceland). Prior to microarray hybridization the PicoGreen assay (Invitrogen Life Sciences, San Diego, California) was used to precisely quantify DNA, and concentrations were standardized to ensure the same quantity of DNA was loaded onto each microarray chip. Following quality control measures, DNA samples were hybridized to Illumina Human 370-Duo genomic microarray chips (Illumina Inc., San Diego, California) and subsequently processed and scanned according to established Illumina protocols. Between-sample variability was minimized by running and scanning all chips on the same instruments during the same time period. Two reference samples were included with each plate to enable determination of data quality and reproducibility. Raw intensity data were normalized and log ratios were calculated using BeadStudio Software (Illumina).

Array data were rigorously QCed to remove poorly performing samples. In addition we identified and removed ethnic outliers from our primarily Caucasian sample collection using well-established methods([1](#_ENREF_1)). After these QCs steps there were 35 azoospermic, 48 oligozoospermic and 62 control individual samples carried forward for analysis.

*Porto*

Samples were genotyped with Affymetrix Genome-Wide Human SNP Array 6.0 at CeGen (Spanish national genotyping centre, Santiago de Compostela node at the University Hospital) by outsourcing and 15 (out of 207) did not pass Affymetrix QC (7% failure). An additional 23 samples were removed that failed our own sample QC for CNV analysis or appeared as ethnic outliers. Note that, as described in the main text, a small number of Porto samples excluded from CNV analysis for technical reasons could be included for inbreeding analysis, as SNP genotyping accuracy is more robust to experimental noise.

*New York*

Array data from the New York cohort were generated by screening genomic DNA, isolated from peripheral blood, on the Affymetrix 6.0 platform at the Genome Sciences Resource at Vanderbilt University (Nashville, Tennessee). After removing samples with known genetic causes of azoospermia, and ethnic outliers, 17 samples remained for analysis.

*St. Louis*

Case samples were screened in two batches on the Illumina OmniExpress 12-plex array at the Genome Technology Access Center of Washington University (gtac.wustl.edu). One hundred male control samples were genotyped on the same platform in the same center by the COGA group in a single batch. Ten case samples were removed that failed array QC, displayed a whole-chromosome aneuploidy, or appeared as ethnic outliers, leaving 61 samples for the primary analysis.

**CNV Methods**

*Illumina Platforms*

Three CNV calling algorithms were used to generate CNV maps for each individual: GADA, a sparse Bayesian learning approach([4](#_ENREF_4)); PennCNV, a Hidden Markov Model (HMM) based method originally designed for the Illumina platform([5](#_ENREF_5)); and QuantiSNP 2.0, another HMM-based method for Illumina([6](#_ENREF_6)). QuantiSNP and PennCNV were run with default settings, and GADA was run using the options “-a 2 –T 5 –M 3”. A criterion of 50% reciprocal overlap between two calls was used to define two calls as identifying the same events, and all CNVs called by at least two algorithms were retained for analysis.

*Affymetrix Platform*

Calling was performed using the Birdsuite package by a pipeline previously described ([7](#_ENREF_7), [8](#_ENREF_8)).

*Sex Chromosome CNV Calling*

Preliminary analyses of our sex chromosome data for these samples suggested that Birdsuite, QuantiSNP and PennCNV made unreliable CNV calls and/or calls that were difficult to interpret for the X and Y. We attribute these issues to normalization decisions hard-coded in both packages and thus we only use GADA calls for analyses of the X and Y. Prior to segmentation of the sex chromosomes by GADA, for each probe intensity we subtract the population median intensity for that probe (recall all samples are male in our collection). This normalization removes spurious CNV calls due to fixed duplications between the X and Y. Overlapping CNV segments from different individuals were clustered into CNV regions (CNVRs) using the criterion of 50% minimum reciprocal overlap. For frequency filtering the minor allele frequency of a CNVR was defined as the number of calls at that CNVR divided by twice the number of samples in the study.

One potential concern about our analysis approach is whether our use of a single calling algorithm on the sex chromosomes could have led to inflated false positive rates. We compared our calls from the Utah cohort to a recent high-resolution CNV discovery project using 42 million probe CGH arrays on 20 male HapMap samples of European ancestry ([9](#_ENREF_9)). In that study, a single calling algorithm (GADA) was used for all chromosomes, identifying an average of 30 X-linked CNVs/ individual, spanning a total of 624kb of sequence on average, and accounting for about 3% of the total aneuploid sequence in the entire genome. These numbers are very similar to the ones that we have produced for normospermic controls in the present study: an average of 133kb aneuploid sequence on the X, accounting for 6% of total genomic aneuploidy per sample. The differences between our study and Conrad, et al. are likely to be explained by (1) the lower probe density used in our study and (2) our calling strategy lowers sensitivity to real CNVs on the autosomes compared to the X.

**Assignment of haplogroups**

SNP genotypes were used to assign each man to a Y haplogroup based on the Y chromosome haplogroup tree defined by Karafet, et al.([10](#_ENREF_10)), using a maximum likelihood approach implemented in the Yfitter program (http://sourceforge.net/projects/**yfitter**/). Haplogroups for some Y chromosomes bearing CNVs are shown in Figure S5.

We experimentally confirmed the Yfitter haplogroup assignments using standard PCR-based approaches. Y STR haplotypes were determined through amplification of 20ng DNA using AmpFlSTR Yfiler PCR Amplification Kit (Applied Biosystems, Foster City, CA) and the products detected by capillary electrophoresis in an ABI 3130 Sequencer and analyzed with GeneMapper v4.0 Software (Applied Biosystems, Foster City, CA). The corresponding Y chromosome haplogoups were predicted with Haplogroup Predictor (http://www.hprg.com/hapest5/).

**Functional Enrichment**

As described in the main text, we searched for significant case-specific aggregation of CNVs in several classes of functional sequence, including 195 genes previously shown to result in spermatogenic defects when mutated in the mouse ([11](#_ENREF_11)), all protein and non-protein coding genes, and testis genes that are differentially expressed during spermatogenesis (described below). Evidence for functional enrichment was assessed jointly across all cohorts, using the same linear mixed-effects modeling framework that we used for estimating rare CNV odds ratios described in the online methods.

**Identification of differentially expressed spermatogenesis genes**

Transcriptional data for 1181 genes that exhibit exceptional statistical significance in testicular expression variance (ANOVA and extensive permutation analysis) were extracted from ([12](#_ENREF_12)). Using the same sample grouping as described in ([12](#_ENREF_12)) (Score 2: Sertoli-cell-only syndrome (SCOS); Score 5: meiotic arrest; Score 8: hypospermatogenesis; Score 10: full spermatogenesis) gene lists of differentially expressed genes between all pairwise comparisons, including a specific group against all others, was created by using the R function “pairwise.t.test” with Bonferroni correction specified as parameter and p-value<0.001 threshold.

**Validation Assays**

**Xp11 deletion**

In the main text we describe a recurrent deletion of ~90kb on Xp11, detected in 9 cases and 8 controls. We validated the presence of these deletions in 8 cases using STS +/- PCR, such as the example shown in Figure S8. The primer sequences for the Xp11 deletion assay and a control locus are given below.

| Primer Name | Sequence | Primer Name | Sequence |
| --- | --- | --- | --- |
| Xp11 90kb qPCR F | CCTAGCACAGAGGGTTCCTG | B-globin F | GTGCATCTGACTCCTGAGGAGA |
| Xp11 90kb qPCR R | GAACCTTGTTGATGGGCTGT | B-globin R | CCTTGATACCAACCTGCCCAG |

Using the same PCR assay, we screened a set of 404 NOA samples with high quality and high quantity of DNA from Weill Cornell. This set of individuals was not pre-screened for known genetic causes of azoospermia; therefore the estimate of the deletion frequency in *bona fide* idiopathic cases is probably an underestimate. We detected 4 deletions in this panel, for a case allele frequency estimate of 0.99%. One of these deletion carriers was screened by Affy6 and included in the PT array results presented, so we report these replication findings as a 3/403 in the main text.

In the Portuguese NOA cohort, we identified two deletion carriers; only one sample had enough DNA available to attempt validation. This patient, Y2502, was validated by amplification of two STS products, one within (DXS6616) and one outside (DXS6950) the deletion. Polymerase reaction was performed in duplex using 200ng of DNA and Qiagen Master Mix (Qiagen, Hilden, Germany). P - patient, C1 and C2 – fertile controls, B - no DNA control.

**DXS6950**

**Left Primer:** TCGGTCTGTGTATCCATGCA

**Right Primer:** GGATGCATGGCAATGGAGAA

**DXS6616**

**Left Primer:** CAAGGCCTTTCACTCTGTCT

**Right Primer:** TACCTCTGTTCATTGCCAAC

P C1 C2 B


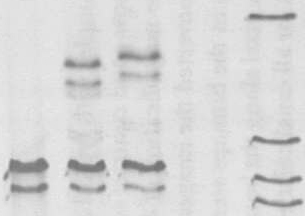


**DXS6950 (out)**

**DXS6616 (in)**

227 bp

117 bp

**DMRT1 deletion qPCR assay**

The hemizygous deletion of DMRT1 identified by array analysis was confirmed in both samples by Taqman PCR using a pre-designed assay #Hs06833797_cn within the DMRT1 gene against an RNase P reference (assay # 4403326; both assays from Applied Biosystems, Carlsbad, CA, USA) according to manufacturer’s recommendations. The DMRT1 assay is located within intron 3 of the gene. As described in the main text, we screened 30 case samples from the Utah cohort with this assay, including 2 putative deletion carriers and 1 putative duplication carrier. These samples had all been screened by Illumina 370K array in the discovery phase of the project, and the DMRT1 TaqMan assay unambiguously confirmed DMRT1 copy number status assigned by the array for 30/30 cases. We subsequently screened the Cornell replication cohort with the assay, running both DMRT1 and RNase P probes in quadruplicate on each sample. Data were summarized into copy number calls using CopyCaller software (Life Technologies). We applied extremely stringent calling criteria to these data, excluding samples with (1) low DNA content as defined by picogreen assay (2) high standard deviation (>0.3) of delta CT measurements across the 4 replicates (3) low copy number confidence scores generated by CopyCaller software. With the criteria of STD dev < 0.3 (when >2 reps available) and low DNA content by VIC and FAM CT (either greater than 30), we detected 2 deletions in 233 samples, a frequency of 0.86%.

**Exome Sequencing and Analysis**

In the main text we described whole exome sequencing of a case of UPD2. This sample was sequenced at the Washington University Genome Technology Access Center (gtac.wustl.edu) on the Illumina HiSeq platform, using a paired-end protocol (2x101bp + a 9bp indexing run). The sample was sequenced to an average sequencing coverage of 140x per coding position, with greater than 95% of the target at 8x coverage. Raw sequence reads were aligned to the reference human genome (hg19) using Novoalign a fast, highly accurate short read alignment tool. Picard tools were used to remove PCR or optical duplicates, and SNPs are called using SAMtools pileup and GATK’s Unified Genotyper. The specificity of SNP calls was improved by requiring detection by both software tools.

We hypothesized that the highly unusual observation of UPD2 in a case of azoospermia was related to the pathology of the disease in this case, and attempted to develop a simple scoring algorithm that would allow us to prioritize all variants in the exome data in terms of their pathogenicity. The program ANNOVAR ([13](#_ENREF_13)) was used to annotate each exome variant with a number of functional prediction programs such as PhyloP, PolyPhen2, SIFT, GERP, and LRT. This score was then multiplied by the ploidy of the mutant allele (e.g. 1x for a heterozygous genotype and 2x for a homozygous genotype) creating a final Individual Score ranging from 0-10. Only sites with minor allele frequencies less than 10% in both the 1000 genomes data and the Exome Variant Server (<http://evs.gs.washington.edu/EVS/>) were considered in the analysis.

**Single Locus CNV Associations**

For sake of completeness we interrogated our CNV call sets for significant single-locus associations, by testing each gene in the whole-genome RefSeq gene set for either case or control-specific enrichment of CNVs; we also partitioned the genome into non-overlapping 500kb windows and tested each window for significant clustering of case or control CNVs. Single locus association testing was done using the software package PLINK, and both nominal and genome-wide p-values were calculated via permutation ([14](#_ENREF_14)). Prior to association testing we removed all CNVRs with minor allele frequency greater than 5%, to avoid spurious results introduced by differential bias. For each cohort, we tested gains, losses, and all CNVs combined, both with and without size thresholds of 100kb. On the following pages we report all nominally significant results for each cohort.

| **Utah** | | | |
| --- | --- | --- | --- |
|  |  |  |  |
| Gene/Window^1^ | p_nominal | p_corr |  |
|  | | | |
| *All CNVs* | | | |
| MKNK2 | 0.0180 | 0.2000 |  |
| chr4:162000001-162500001 | 0.0102 | 0.2119 |  |
| chr19:1500001-2000001 | 0.0205 | 0.4144 |  |
|  | | | |
| *All Deletions* | | | |
| None significant |  |  |  |
|  | | | |
| *All Duplications* | | | |
| MKNK2 | 0.0187 | 0.1387 |  |
| chr4:162000001-162500001 | 0.0207 | 0.1680 |  |
| chr19:1500001-2000001 | 0.0208 | 0.1680 |  |
|  |  |  |  |
| *CNVs > 100kb* |  |  |  |
| chrY:20500001-21000001 | 0.0354 | 0.3243 |  |
|  |  |  |  |
| *Deletions > 100kb* |  |  |  |
| chrY:20500001-21000001 | 0.0360 | 0.1970 |  |
|  |  |  |  |
| **WUSTL** | | | |
|  |  |  |  |
| Gene/Window^2^ | p_nominal | p_corr |  |
|  | | | |
| *All CNVs* | | | |
| UBE2K | 0.0195 | 0.2044 |  |
| chr9:94000001-94500001 | 0.0012 | 0.0179 |  |
| chrX:31000001-31500001 | 0.0059 | 0.3190 |  |
|  | | | |
| *All Deletions* | | | |
| UBE2K | 0.0250 | 0.1455 |  |
| chr9:94000001-94500001 | 0.0008 | 0.0142 |  |
|  | | | |
| *All Duplications* | | | |
| DMD | 0.0069 | 0.0190 |  |
| chrX:31000001-31500001 | 0.0065 | 0.0870 |  |
|  |  |  |  |
| *CNVs > 100kb* |  |  |  |
| None significant |  |  |  |
|  | | | |
| *Deletions > 100kb* | | | |
| None significant |  |  |  |
|  | | | |
| *All Duplications* | | | |
| None significant |  |  |  |
|  | | | |
| *Duplications > 100kb* | | | |
| None significant |  |  |  |
|  |  |  |  |
| **Porto + Weill Cornell** | | | |
|  |  |  |  |
| Gene/Window^1^ | p_nominal | p_corr |  |
|  | | | |
| *All CNVs* | | | |
| EPHA3 | 0.0244 | 0.2510 |  |
| CSMD1 | 0.0006 | 0.0598 |  |
| DHRSX | 0.0002 | 0.0328 |  |
| DMD | 0.0017 | 0.3910 |  |
| EDA | 0.0045 | 0.2510 |  |
| TAF7L | 0.0024 | 0.2774 |  |
| chr1:16500001-17000001 | 0.0004 | 0.1762 |  |
| chr2:97000001-97500001 | 0.0051 | 0.2046 |  |
| chr3:127000001-127500001 | 0.1000 | 0.0007 |  |
| chr4:57500001-58000001 | 0.0012 | 0.2906 |  |
| chr8:3500001-4000001 | 0.0003 | 0.1609 |  |
| chr8:13500001-14000001 | 0.0015 | 0.2864 |  |
| chr21:22758742-23258742 | 0.0020 | 0.2864 |  |
| chrX:2000001-2500001 | 0.0003 | 0.1609 |  |
| chrX:17000001-17500001 | 0.0006 | 0.0991 |  |
| chrX:55500001-56000001 | 0.0011 | 0.2046 |  |
| chrX:90500001-91000001 | 0.0005 | 0.0991 |  |
| chrY:7000001-7500001 | 0.1000 | 0.0121 |  |
| chrY:20500001-21000001 | 0.1000 | 0.0042 |  |
|  | | | |
| *All Deletions* | | | |
| CSMD1 | 0.0009 | 0.0313 |  |
| EDA | 0.0027 | 0.1173 |  |
| TAF7L | 0.0030 | 0.1340 |  |
| chr2:97000001-97500001 | 0.0111 | 0.1756 |  |
| chr3:76000001-76500001 | 0.0041 | 0.4756 |  |
| chr3:127000001-127500001 | 0.1000 | 0.0005 |  |
| chr4:168500001-169000001 | 0.0037 | 0.4756 |  |
| chr8:3500001-4000001 | 0.0006 | 0.1012 |  |
| chr8:96500001-97000001 | 0.0037 | 0.4756 |  |
| chr17:6000001-6500001 | 0.0039 | 0.4756 |  |
| chr21:22758742-23258742 | 0.0020 | 0.1756 |  |
| chrX:4500001-5000001 | 0.0048 | 0.4756 |  |
| chrX:55500001-56000001 | 0.0007 | 0.1211 |  |
| chrX:68500001-69000001 | 0.0035 | 0.4756 |  |
| chrX:90500001-91000001 | 0.0005 | 0.0671 |  |
| chrX:150500001-151000001 | 0.0041 | 0.4756 |  |
| chrY:7000001-7500001 | 0.1000 | 0.0065 |  |
|  | | | |
| *All Duplications* | | | |
| EPHA3 | 0.0270 | 0.0943 |  |
| DHRSX | 0.1000 | 0.0009 |  |
| chr4:9500001-10000001 | 0.0238 | 0.4187 |  |
| chr4:57500001-58000001 | 0.0009 | 0.0690 |  |
| chr8:54000001-54500001 | 0.0038 | 0.4187 |  |
| chr12:34000001-34500001 | 0.0002 | 0.0019 |  |
| chr12:110500001-111000001 | 0.0021 | 0.4838 |  |
| chr14:103452209-103952209 | 0.0029 | 0.4187 |  |
| chrX:2000001-2500001 | 0.0003 | 0.0107 |  |
| chrX:114500001-115000001 | 0.0034 | 0.4187 |  |
| chrY:20500001-21000001 | 0.1000 | 0.1000 |  |
|  | | | |
| *CNVs > 100kb* | | | |
| EPHA3 | 0.0241 | 0.0574 |  |
| PARD3 | 0.0253 | 0.3610 |  |
| chr1:16500001-17000001 | 0.0012 | 0.2766 |  |
| chr2:97000001-97500001 | 0.0013 | 0.0572 |  |
| chr3:89000001-89500001 | 0.0235 | 0.2288 |  |
| chr11:134000002-134449982 | 0.0072 | 0.4212 |  |
| chr12:110500001-111000001 | 0.0026 | 0.2615 |  |
| chrX:148500001-149000001 | 0.0041 | 0.2288 |  |
|  | | | |
| *Deletions > 100kb* | | | |
| CNTNAP3 | 0.0232 | 0.0965 |  |
| chr2:97000001-97500001 | 0.0018 | 0.1043 |  |
| *Duplications > 100kb* | | | |
| EPHA3 | 0.0252 | 0.0436 |  |
| PARD3 | 0.0255 | 0.2867 |  |
| chr1:16500001-17000001 | 0.0018 | 0.2232 |  |
| chr3:89000001-89500001 | 0.0247 | 0.1570 |  |
| chr11:134000002-134449982 | 0.0077 | 0.3041 |  |
| chr12:110500001-111000001 | 0.0031 | 0.1897 |  |

^1^ Coordinates in hg18

^2^ Coordinates in hg19

**Acknowledgments**

The Collaborative Study on the Genetics of Alcoholism (COGA), Principal Investigators B. Porjesz, V. Hesselbrock, H. Edenberg, L. Bierut, includes ten different centers: University of Connecticut (V. Hesselbrock); Indiana University (H.J. Edenberg, J. Nurnberger Jr., T. Foroud); University of Iowa (S. Kuperman, J. Kramer); SUNY Downstate (B. Porjesz); Washington University in St. Louis (L. Bierut, A. Goate, J. Rice, K. Bucholz); University of California at San Diego (M. Schuckit); Rutgers University (J. Tischfield); Southwest Foundation (L. Almasy), Howard University (R. Taylor) and Virginia Commonwealth University (D. Dick). Other COGA collaborators include: L. Bauer (University of Connecticut); D. Koller, S. O’Connor, L. Wetherill, X. Xuei (Indiana University); Grace Chan (University of Iowa); N. Manz, M. Rangaswamy (SUNY Downstate); A. Hinrichs, J. Rohrbaugh, J-C Wang (Washington University in St. Louis); A. Brooks (Rutgers University); and F. Aliev (Virginia Commonwealth University). A. Parsian and M. Reilly are the NIAAA Staff Collaborators. We continue to be inspired by our memories of Henri Begleiter and Theodore Reich, founding PI and Co-PI of COGA, and also owe a debt of gratitude to other past organizers of COGA, including Ting-Kai Li, currently a consultant with COGA, P. Michael Conneally, Raymond Crowe, and Wendy Reich, for their critical contributions. This national collaborative study is supported by NIH Grant U10AA008401 from the National Institute on Alcohol Abuse and Alcoholism (NIAAA) and the National Institute on Drug Abuse (NIDA).

1. Price AL*, et al.* (2006) Principal components analysis corrects for stratification in genome-wide association studies. *Nature genetics* 38(8):904-909.

2. Price AL*, et al.* (2009) Sensitive detection of chromosomal segments of distinct ancestry in admixed populations. *PLoS genetics* 5(6):e1000519.

3. Hu Z*, et al.* (2012) A genome-wide association study in Chinese men identifies three risk loci for non-obstructive azoospermia. *Nature genetics* 44(2):183-186.

4. Pique-Regi R, Ortega A, & Asgharzadeh S (2009) Joint estimation of copy number variation and reference intensities on multiple DNA arrays using GADA. *Bioinformatics* 25(10):1223-1230.

5. Wang K*, et al.* (2007) PennCNV: an integrated hidden Markov model designed for high-resolution copy number variation detection in whole-genome SNP genotyping data. *Genome Res* 17(11):1665-1674.

6. Colella S*, et al.* (2007) QuantiSNP: an Objective Bayes Hidden-Markov Model to detect and accurately map copy number variation using SNP genotyping data. *Nucleic Acids Res* 35(6):2013-2025.

7. Bochukova EG*, et al.* (2010) Large, rare chromosomal deletions associated with severe early-onset obesity. *Nature* 463(7281):666-670.

8. Korn JM*, et al.* (2008) Integrated genotype calling and association analysis of SNPs, common copy number polymorphisms and rare CNVs. *Nature genetics* 40(10):1253-1260.

9. Conrad DF*, et al.* (2010) Origins and functional impact of copy number variation in the human genome. *Nature* 464(7289):704-712.

10. Karafet TM*, et al.* (2008) New binary polymorphisms reshape and increase resolution of the human Y chromosomal haplogroup tree. *Genome Res* 18(5):830-838.

11. Matzuk MM & Lamb DJ (2008) The biology of infertility: research advances and clinical challenges. *Nat Med* 14(11):1197-1213.

12. Feig C*, et al.* (2007) A new paradigm for profiling testicular gene expression during normal and disturbed human spermatogenesis. *Mol Hum Reprod* 13(1):33-43.

13. Wang K, Li M, & Hakonarson H (2010) ANNOVAR: functional annotation of genetic variants from high-throughput sequencing data. *Nucleic Acids Res* 38(16):e164.

14. Purcell S*, et al.* (2007) PLINK: a tool set for whole-genome association and population-based linkage analyses. *Am J Hum Genet* 81(3):559-575.
